# Protein biomarker signature in patients with spinal and bulbar muscular atrophy

**DOI:** 10.1172/jci.insight.176383

**Published:** 2024-05-30

**Authors:** Andrew T.N. Tebbenkamp, Spencer B. Huggett, Vittoria Lombardi, Luca Zampedri, Abdullah AlQahtani, Angela Kokkinis, Andrea Malaspina, Carlo Rinaldi, Christopher Grunseich, Pietro Fratta, Vissia Viglietta

**Affiliations:** 1 Nido Biosciences, Inc., Boston, Massachusetts, USA.; 2 University College London (UCL), London, United Kingdom.; 3 Neurogenetics Branch, National Institute of Neurological Disease and Stroke, National Institutes of Health, Bethesda, Maryland, USA.; 4 Institute of Developmental and Regenerative Medicine, University of Oxford, Oxford, United Kingdom.

**Keywords:** Muscle biology, Neuroscience, Genetic diseases, Neuromuscular disease, Proteomics

## Abstract

Spinal and bulbar muscular atrophy (SBMA) is a slowly progressing disease with limited sensitive biomarkers that support clinical research. We analyzed plasma and serum samples from patients with SBMA and matched healthy controls in multiple cohorts, identifying 40 highly reproducible SBMA-associated proteins out of nearly 3,000 measured. These proteins were robustly enriched in gene sets of skeletal muscle expression and processes related to mitochondria and calcium signaling. Many proteins outperformed currently used clinical laboratory tests (e.g., creatine kinase [CK]) in distinguishing patients from controls and in their correlations with clinical and functional traits in patients. Two of the 40 proteins, Ectodysplasin A2 receptor (EDA2R) and Repulsive guidance molecule A (RGMA), were found to be associated with decreased survival and body weight in a mouse model of SBMA. In summary, we identified what we believe to be a robust and novel set of fluid protein biomarkers in SBMA that are linked with relevant disease features in patients and in a mouse model of disease. Changes in these SBMA-associated proteins could be used as an early predictor of treatment effects in clinical trials.

## Abstract 2

A spinal and bulbar muscular atrophy-associated protein signature correlates with relevant disease features and has potential as an important clinical biomarker of treatment effect.

## Introduction

Spinal and bulbar muscular atrophy (SBMA) is an inherited, slowly progressive neuromuscular disorder with no FDA-approved disease-modifying therapy. Some of its characteristics include muscle weakness and atrophy, loss of motor neurons, metabolic disease, and testicular atrophy (1–3). SBMA is caused by a cytosine-adenine-guanine (CAG) repeat expansion in exon 1 of the androgen receptor gene (*AR*), which is on the X-chromosome. Therefore, SBMA only affects males, while females are carriers (4, 5). Healthy individuals have between 9 and 36 CAG repeats, while patients have between 38 and 68 repeats (5, 6). Expanded CAG repeats lead to abnormal AR function, involving both gain of toxic functions and loss of function. Mutant AR triggers transcriptional dysregulation, protein aggregation, and other cascading events (7). With AR expressed in diverse tissues and eliciting multiple toxic mechanisms, disease complexity poses a challenge for identifying fluid biomarkers suitable for tracking disease progression or informing pharmacodynamic effects in clinical settings (2).

The use of biomarkers increases clinical trial success (8). Sometimes, biomarkers may be the sole reason for FDA approval. For example, the FDA recently granted accelerated approval to the amyotrophic lateral sclerosis therapeutic tofersen (QALSODY), based on its ability to reduce neurofilament light chain (NfL) protein levels in the blood (9). In SBMA, fluid biomarkers used in clinical studies — such as CK, creatinine, myoglobin, and liver transaminases (10–14) — exhibited variability across patient cohorts and inconsistent associations with clinical characteristics and generally failed to track disease progression.

Extensive efforts have been made to identify biomarkers to measure disease progression or therapeutic effects in similar complex diseases such as ALS, where the heterogeneity of disease manifestation together with the multiple disease mechanisms involved has hindered the assessment of potential treatments. This has led to the discovery of several promising biomarkers reflecting the different sites of pathology in ALS, such as NfL, which indicates motor neuron degeneration and is currently the biomarker of choice in ALS studies given its abundance and correlation with clinical outcomes (15). Another biomarker found to be associated with ALS severity was the cardiac troponin T (cTnT), which also prompted investigations in SBMA where this marker was found to be increased in patients with SBMA and correlated with lower limb impairment (16). The work in ALS and SBMA also highlights the need for more than 1 marker to address the multiple pathophysiological mechanisms of these diseases.

The lack of robust biomarkers in SBMA may limit the evaluation of efficacy in interventional trials, and additional studies are needed to identify potential pharmacodynamic biomarkers for interventional trials.

This study aimed to uncover novel blood protein biomarkers in SBMA. Protein biomarkers were compared against current clinical laboratory markers and were characterized through gene set analyses, correlations with clinical disease features, and evaluation in a SBMA mouse model. We employed a targeted proteomic approach, harnessing proximity extension assay technology for enhanced multiplexing and sensitivity in protein detection. Our research unveiled proteins with known alterations in SBMA and other neuromuscular diseases, as well as additional proteins that were associated with SBMA, disease characteristics in patients, and a SBMA mouse model.

## Results

### Discovery of SBMA proteomic signature from patient cohorts.

**Figure 1:**
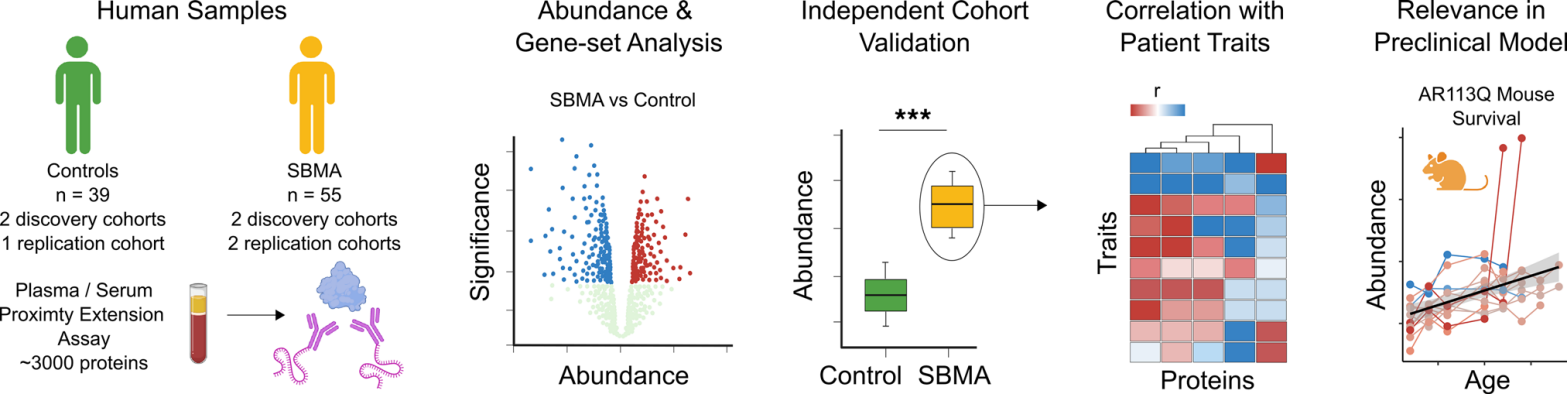
Study design. Illustration outlining the experimental design and analyses. Multiplexed proximity extension assay was used to discover protein biomarkers in the plasma/serum from patients with SBMA. These proteins were then measured against clinical outcomes from these patients and in the AR113Q mouse model of SBMA.

The outline of our study is shown in Figure 1. Briefly, we investigated plasma and serum proteomes from patients with SBMA and age-matched healthy controls. We examined reproducibility across multiple cohorts, conducted gene set enrichment analyses (GSEA) to characterize findings, investigated correlations between protein abundance and clinical features, and characterized protein abundance in a SBMA mouse model.

**Figure 2:**
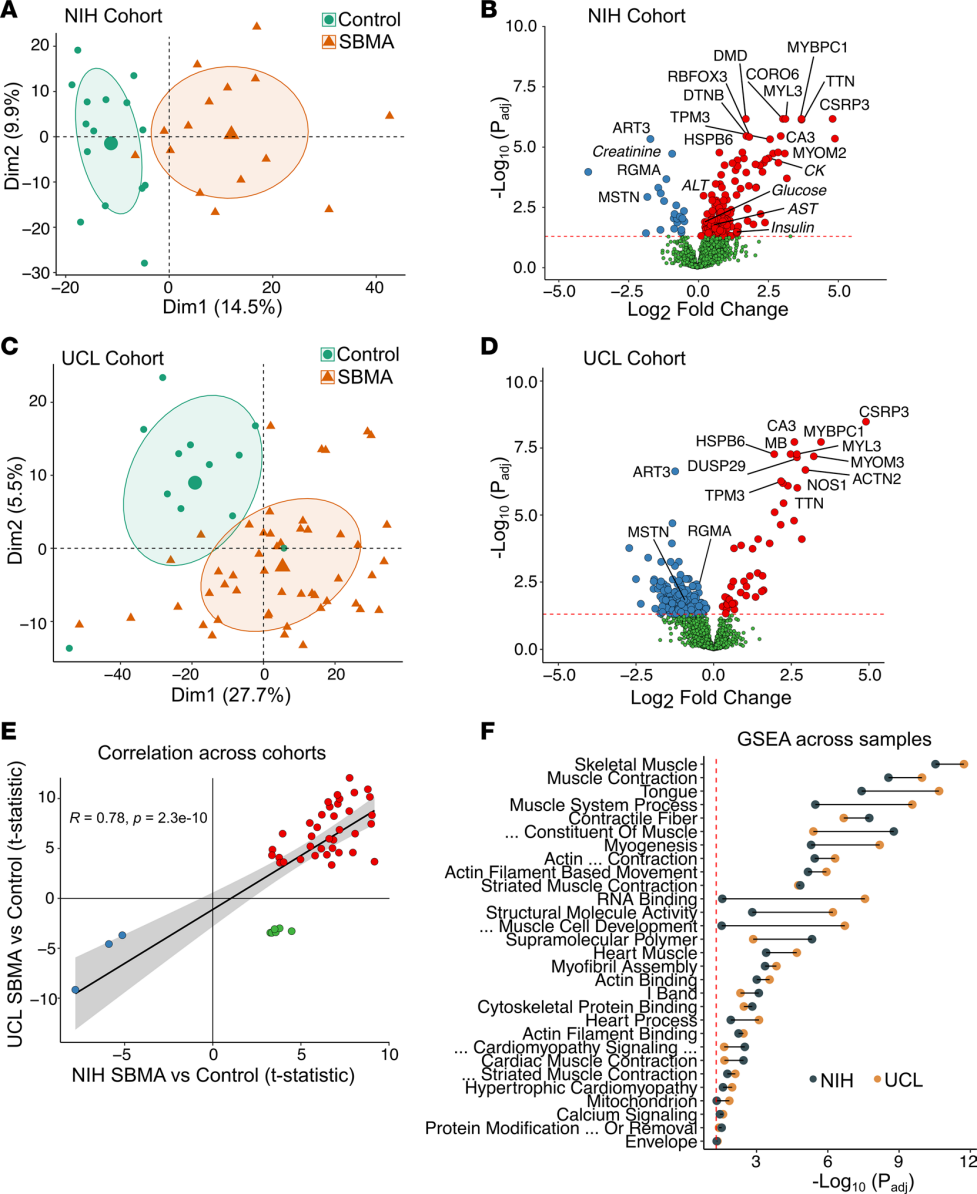
Discovery of protein biomarker signature in SBMA. (**A**) PCA of control and patients with SBMA from the NIH cohort (*n* = 15 SBMA; *n* = 15 control). Differential abundance in protein expression was calculated using a linear regression framework, adjusting for age. (**B**) Volcano plot of all measured proteins from the NIH cohort. Proteins in red were increased, and proteins in blue were decreased. Proteins in green were not significantly different (*P*_adj_ < 0.05 cut-off, dotted red line). Top proteins are labeled, and clinical labs measured in those patients are in bold and italicized. (**C**) PCA of samples from the UCL cohort (*n* = 19 SBMA; *n* = 12 control). (**D**) Volcano plot as described in **B**. (**E**) Correlation of proteins consistent across both cohorts; red indicates increased in both, blue indicates decreased in both, and green indicates inconsistent across cohorts. (**F**) Gene set enrichment analysis (GSEA) of SBMA proteomic associations showing the tissues and biological functions that were significantly enriched across cohorts.

For a summary of the data used in our study, see Table 1. We leveraged 2 independent discovery cohorts to investigate protein differences between SBMA and control samples, with all samples from male donors and patients with SBMA having a confirmed genetic diagnosis. One discovery cohort used samples obtained from research conducted at the NIH, with average age of patients with SBMA of 57.6 years (y) (±7.8 y) and of control donors 58.7 y (±7.3 y) (17). The other discovery cohort came from the UCL, from a longitudinal nonintervention study (UCL Discovery). The average ages from this cohort were 60.8 y (±9.3 y) and 59.4 y (±10.7 y) from patients with SBMA and control donors, respectively. To determine the proteomic profile of patients with SBMA, we utilized the Olink Explore 3072 platform, which assesses the abundance of 2,925 unique proteins. Using principal component analyses (PCA) on all assessed proteins, we found distinct separation of patients with SBMA from matched control samples across cohorts (Figure 2, A and C). We identified 185 and 280 proteins that exhibited differential abundance between SBMA versus controls in NIH and UCL cohorts, respectively (*P*_adj_ < 0.05 and limit of detection > 50%; Figure 2, B and D). Applying a more conservative log_2_ fold change > |0.5| cut-off did not substantially change the number of differentially abundant proteins, with 95% (NIH cohort) and 97.5% (UCL cohort) of proteins retaining their differential abundance. Across cohorts, 46 proteins were significantly associated with SBMA (*P*_adj_ <0.05 and limit of detection > 50%). The differential abundance of these proteins was highly correlated across cohorts (*r* = 0.78, *P* < 0.0001) with 40 of 46 of these proteins (87%) being estimated in the same direction in SBMA across cohorts (Figure 2E and Supplemental Table 1; supplemental material available online with this article; https://doi.org/10.1172/jci.insight.176383DS1). Herein, these 40 proteins are referred to as the “SBMA-associated proteins” (Table 2).

The NIH cohort included clinical laboratory tests from both patients and controls, revealing differences in 6 of 14 measures between SBMA and controls (including creatinine, CK, glucose, insulin, alanine transaminase, and aspartate transaminase; *P*_adj_ < 0.05). CK is widely used across several neuromuscular disorders, including prior SBMA interventional trials (18, 19). In the NIH cohort, half of the SBMA-associated proteins (20 of 40) exhibited a more significant association with SBMA than CK (*P*_adj_ < 4.6 × 10^–5^) and 77.5% of these proteins (31 of 40) showed less variability than CK in patients with SBMA (coefficient of variation < 49.8%). This suggests that these proteins may offer enhanced sensitivity and reliability to currently available biomarkers.

To gain more insight into the biological systems in which these proteomes were enriched, we performed GSEA on thousands of gene sets using all detected proteins in each cohort. We found that 29 gene sets were significantly enriched across cohorts (*P*_adj_ < 0.05). The most significant enrichment was observed in gene sets associated with skeletal muscle expression and function, but significant enrichment was also detected in RNA Binding, Mitochondrion, and Calcium Signaling, among other gene sets (Figure 2F and Supplemental Table 2).

### Replication of SBMA-associated proteins in independent patient cohorts.

**Figure 3:**
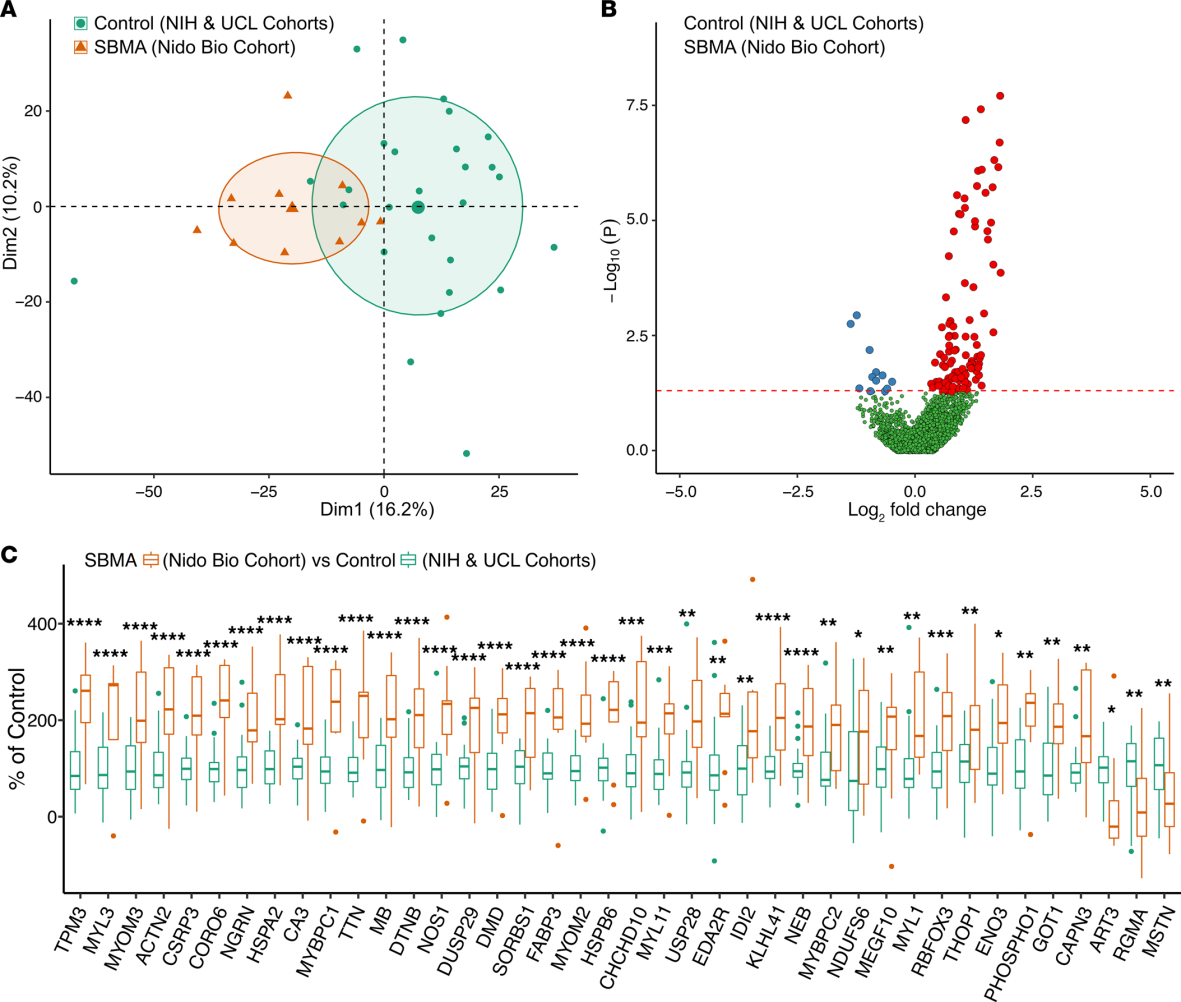
Replication of SBMA proteomic signature. (**A**) PCA of Nido Biosciences SBMA samples (*n* = 9) compared with control samples from NIH and UCL. (**B**) Volcano plot of increased (red), decreased (blue), or unchanged (green) proteins. (**C**) Log_2_ fold change of 40 SBMA-associated proteins from Nido Bio compared with control samples from NIH (*n* = 15) and UCL cohorts (*n* = 12). Data are plotted as mean ± SEM. *P < 0.05; **P < 0.01; ***P < 0.001; ****P < 0.0001.

Plasma samples were also collected in a separate, multisite observational study composed of only patients with SBMA (Table 1). Plasma proteomes from these patients were analyzed on the same Olink Explore 3072 platform and compared with the control samples from the 2 discovery cohorts. Once again, PCA indicated that the SBMA samples clustered separately from the control samples, suggesting proteome-level differences between cases and controls (Figure 3A). In this data set, the abundance of 126 proteins were significantly different in SBMA than controls (*P* < 0.05; Figure 3B). Furthermore, all 40 SBMA-associated proteins demonstrated differential abundance (*P* < 0.05) in this independent SBMA cohort, building further evidence for the robustness of these biomarkers (Figure 3C).

Most of the SBMA-associated proteins had increased abundance in patients, while Repulsive Guidance Molecule A (RGMA), MSTN, and ART3 were the only 3 proteins that were decreased in patients as compared with healthy controls. Some of the proteins with the highest fold change have also been identified in samples from patients with other skeletal muscle–related disorders such as Duchenne muscular dystrophy (DMD) or facioscapulohumeral muscular dystrophy (FSHD) (20–25) (Table 2). It is noteworthy that CK is also elevated in cases of DMD and FSHD, but the creatine kinase – muscle isoform (CKM) is not measured in the 3072 platform. By comparison, biomarker papers from more neural-related disorders such as amyotrophic lateral sclerosis, Parkinson’s disease, or Huntington’s disease did not report differential abundance of the 40 SBMA-associated proteins (26–28).

A separate replication cohort of patients with SBMA and healthy controls from UCL (UCL Replication) was analyzed on a more limited panel of 1,536 antibody pairs. Similarly in this cohort, we found that patients with SBMA showed modest clustering from healthy controls and that 113 proteins showed differential abundance between patients with SBMA and healthy controls (*P* < 0.05; Supplemental Figure 1, A and B). However, within this limited panel, only 13 of the 40 SBMA-associated proteins were assessed, and 10 of the 13 assessed proteins (76.9%) exhibited differential abundance between SBMA and controls (Supplemental Figure 1C).

To further establish that these proteins could reliably distinguish patients with SBMA from healthy controls, we generated ROC curves for each of the 40 proteins and confirmed substantial consistency across cohorts with an average AUC of 0.94 (SD = 0.06) (Supplemental Figure 2). While these data illustrate the utility of these markers in separating patients from healthy controls, they are not intended to be diagnostic, which is done by a genetic test.

Using the Human Protein Atlas, we mapped the human tissue expression of these 40 SBMA-associated proteins and found that many of them were enriched in skeletal muscle and tongue and slightly less in cardiac muscle (Supplemental Figure 3). These expression profiles align with our GSEA and further support that muscle tissue is a major site of pathology of SBMA. A few of the SBMA-associated proteins have ubiquitous expression patterns across tissues, such as NDUFS6 and CHCHD10, which are localized to mitochondria, with the latter being associated with a subtype of ALS (29). Others are expressed in tissues relevant to other sites of SBMA pathology, such as ART3 and PHOSPHO1 (testis), MEGF10 and NGRN (spinal cord), and GOT1 (liver).

### Overlap of SBMA-associated proteins and AR gene regulation data sets.

Given that these proteins were detected in circulating biofluids, identifying their tissue expression patterns is insufficient to fully explain their relevance to SBMA pathogenesis. To gain further insight into potential associations with AR dysfunction, we intersected the 40 SBMA-associated proteins with a published data set that used primary human skeletal muscle myoblasts to perform AR ChIP coupled with microarrays (30). Proteins whose genes contain androgen response elements (AREs) and are bound by AR may be more likely to have altered levels in the context of SBMA. Using this data set, we found 19 proteins whose encoding genes have evidence of being regulated by AR (Supplemental Figure 4A). Dystrobrevin β (DTNB) followed by CAPN3 and DMD have the highest peak values, suggesting that their genes contain AREs that are more likely to be occupied by AR.

To further investigate whether these 40 proteins may be regulated by AR, we also interrogated a data set derived from RNA-Seq on skeletal muscle from WT and *Ar*-KO mice (31). Genes that were differentially expressed between *Ar*-KO and WT muscle after DHT administration include *Mstn*, *Art3*, *Mb*, *Dmd*, and *Nos1* (Supplemental Figure 4B). In total, 12 genes had altered expression with the 5 most affected genes being downregulated. Although these data sets have some limitations, their information is complementary, and therefore proteins that were identified in both analyses — MSTN, ART3, MB, DMD, TTN, and MYL1 — suggest an increased likelihood of being directly regulated by AR. It should be noted that gene regulation by AR is one of many mechanisms by which fluid biomarkers can be altered. Proteolysis of extracellular domains or passive release from tissue damage are also likely mechanisms.

### Correlations between patient functional assessments and SBMA-associated proteins.

**Figure 4:**
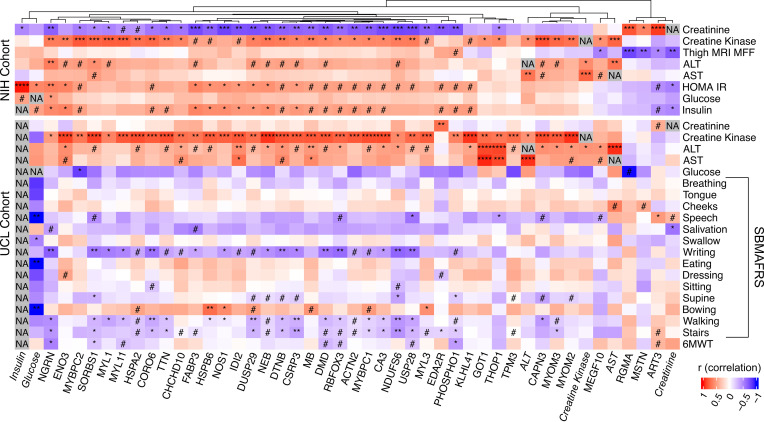
A heatmap showing the correlations (*r*) of the 40 SBMA-associated proteins with clinical labs and functional readouts in the same patients using linear regression. The different cohorts are labeled on the left of the heatmap, proteins and clinical labs (italicized) are labeled on the bottom, and traits are labeled on the right. The dendrogram at the top clusters the proteins via a hierarchical clustering algorithm. ^#^*P* < 0.1; *P < 0.05; **P < 0.01; ***P < 0.001; ****P < 0.0001 as assessed by linear regression. NA, not applicable; MFF, muscle fat fraction.

For some SBMA cohort blood measures, muscle MRI and clinical functional assessments were also performed at the time of sample collection. Muscle MRI has recently been shown to be an informative biomarker, as it quantifies muscle atrophy and fat infiltration into the muscles, 2 major features of SBMA (32). Furthermore, muscle MRI measurements were shown to correlate with clinical assessments. These additional measurements allowed us to correlate the abundance of the 40 SBMA-associated proteins with other meaningful aspects of the disease within patients and investigate the connection of these biomarkers with relevant metrics of disease. In the NIH cohort, we found that nearly every protein correlated with at least 1 clinical parameter measured in those patients. Only 3 proteins, including RGMA, MSTN, and ART3, and 1 metabolite (creatinine) were found to have decreased abundances in plasma, all of which significantly correlated with higher thigh MRI muscle fat fraction (MFF) (Figure 4). In the UCL cohort, the SBMA Functional Rating Scale (SBMAFRS; a rater-based measurement of 5 domains and multiple subdomains relevant to SBMA phenotypes) (33) and the 6-minute walk test (6MWT) functional assessments were collected. Many proteins that were increased in plasma had an inverse correlation with “walking” and “stairs” subscales of the SBMAFRS scores, indicating that higher protein levels correlated with lower scores and increased disease severity. Many of the proteins that correlated with lower-limb items on the SBMAFRS were also associated with the 6MWT in the same direction, suggesting consistency across functional assessments. Examples of individual proteins significantly associated with lower limb impairment are reported in Supplemental Figure 5.

Some patients in the UCL cohort also had longitudinal follow-up assessments and blood collections every 13 months on average (Table 1), and this allowed us to assess the dynamics of the SBMA-associated proteins over time. Results show that, of the 40 SBMA-associated proteins, only RGMA and MEGF10 change over time (Supplemental Figure 6) while most proteins in the SBMA proteomic signature were stable over approximately 1 y.

### Correlations between SBMA-associated proteins and phenotypes in SBMA preclinical mouse model.

**Figure 5:**
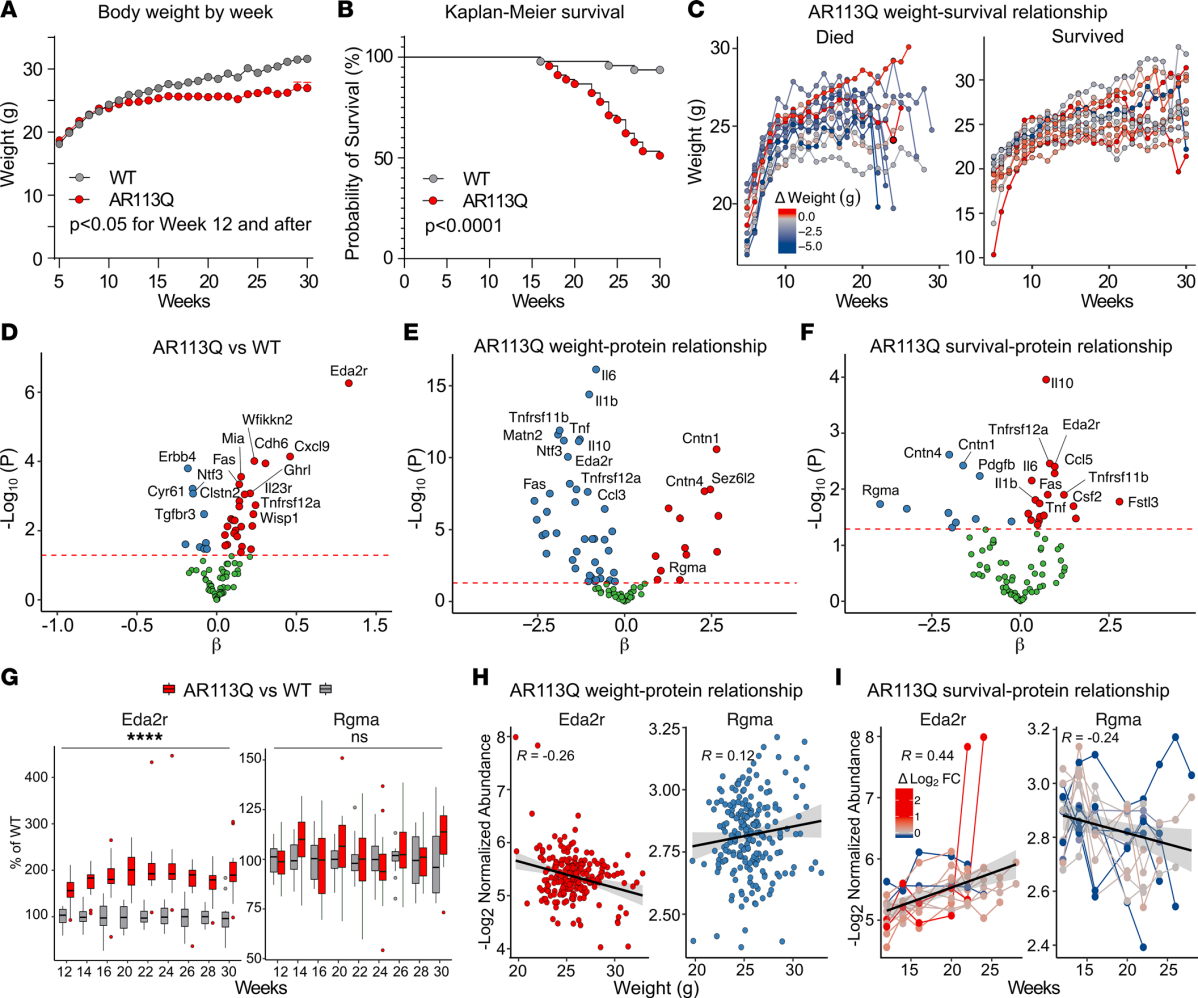
Phenotypes and protein signature in AR113Q mice. (**A**) Weekly body weight of WT and AR113Q mice from 5–30 weeks of age (2-way ANOVA). At study start, *n* = 47 for WT and *n* = 45 for AR113Q, with the number of mice that died each week during the study subtracted. (**B**) Kaplan-Meier survival plot comparing WT and AR113Q mice. The significance was measured at 30 weeks (Mantel-Cox). (**C**) Relationship between body weight and survival, categorized by AR113Q mice that died or survived. (**D**) Volcano plot of 92 proteins, as described previously. The red dashed line represents a *P* < 0.05 from multilevel metaregression models. (**E** and **F**) Volcano plots showing the correlation of protein levels with body weight or rate of death, respectively, across time in AR113Q mice. Eda2r: hazard ratio = 2.63, *P* = 0.003; Rgma: hazard ratio = 0.019, *P* = 0.0185 via time-varying Cox proportional hazard ratio models. All proteins below the red dashed line (*P* < 0.05) were not associated with weight or survival. The β represents the regression coefficient/slope of the model. (**G**) Differential abundance (log_2_ fold change) of Eda2r and Rgma between AR113Q and WT at different ages. *n* = 12 for WT and *n* = 15–30 for AR113Q mice, depending on each week. Week 18 samples were not available for analysis. ****P < 0.0001. (**H**) Change in levels of Eda2r and Rgma and association with body weight in AR113Q mice. (**I**) Change in levels of Eda2r and Rgma over time in AR113Q mice that died.

Finally, we utilized the AR113Q preclinical mouse model of SBMA, which was generated by knocking in a human *AR* exon 1 with 113 CAG repeats to the mouse *Ar* locus (34). These mice mimic the genetic cause of SBMA and have been shown to recapitulate the pathological features of the disease such as muscle atrophy and polyQ AR protein accumulation. Consistent with previous studies, our data confirm that AR113Q mice exhibit body weight loss of approximately 15% from WT and exhibit decreased survival to 50% by 30 weeks of age (Figure 5, A and B). Currently, the cause of either of these phenotypes is not well known. To determine if these 2 phenotypes were related to each other, we used time-varying Cox proportional hazard ratio modeling and found that decreases in body weight were significantly associated with premature death in AR113Q mice (*P* = 0.0374; Figure 5C).

To measure the plasma proteomic profile of AR113Q mice, we again used the Olink platform; however, this rodent panel was limited and only measured 92 proteins. Of these 92 proteins, only 2 of the 40 SBMA-associated proteins were measured. We found that 36 proteins exhibited differential abundance between AR113Q versus WT (*P* < 0.05; Figure 5D and Supplemental Table 3). Interestingly, Ectodysplasin A2 receptor (Eda2r; Xedar, Tnfrsf27) which was one of the 2 SBMA-associated proteins measured in this panel, was significantly increased in AR113Q mice (*P* = 5.47 × 10^–7^). Eda2r is a single-pass transmembrane receptor of the TNF family, located near *AR* on the X-chromosome, and is linked to several overlapping features of patients with SBMA or AR biology, including aging, metabolic dysfunction, dysregulation of hair growth (androgenic alopecia), and muscle atrophy (35, 36).

We also tested whether plasma protein levels in AR113Q mice correlated with body weight or survival phenotypes. Our analyses identified several proteins that were significantly associated with body weight (Figure 5E) and survival (Figure 5F) using mixed-effects regression and time-varying Cox proportional hazard ratios models. Increases in Eda2r were associated with decreases in body weight and premature death in AR113Q mice (Figure 5, G–I). Decreases in Rgma, the other SBMA-associated protein measured in this panel, was associated with decreased body weight and premature death in AR113Q mice (Figure 5, G–I). Rgma is a GPI-anchored glycoprotein that functions as a guidance molecule, with tissue-dependent roles of cellular adhesion or repulsion (37–39). Rgma has an important role in nervous system development and maintenance as well as myoblast fusion and myotube hypertrophy. Together, both Eda2r and Rgma were constituents of the SBMA proteomic signature, correlated with other SBMA-relevant endpoints, and are reported to function in biology relevant to the pathophysiology of SBMA, making them interesting targets for further investigation in the context of SBMA.

## Discussion

**Figure 6:**
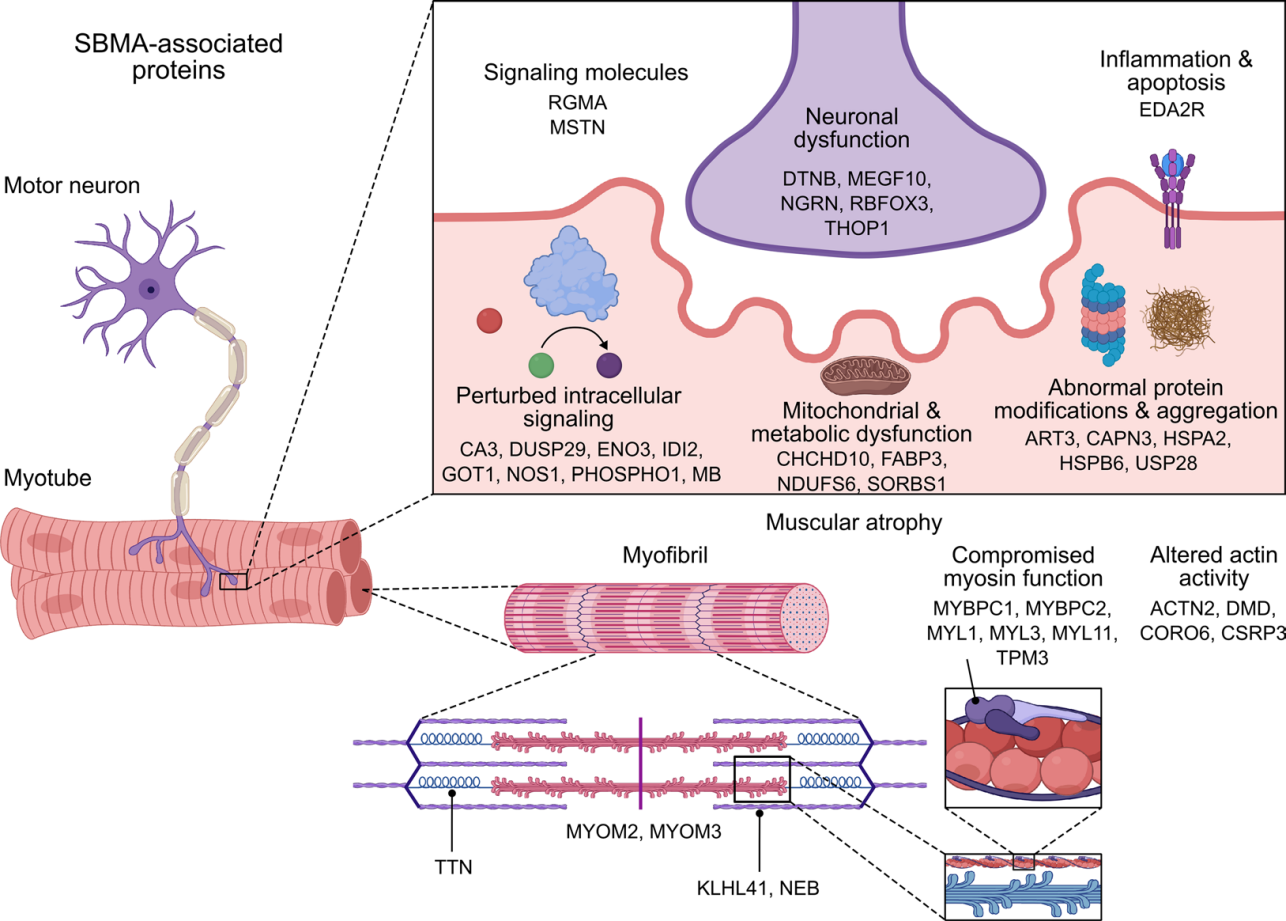
Contextual summary of 40 SBMA-associated proteins. All 40 proteins are labeled next to their specific or general molecular functions as they may relate to neuromuscular biology in SBMA. The listed molecular functions or localization of proteins is not meant to rigidly define these proteins but, rather, to link the constellation of potential mechanisms that may contribute to SBMA pathophysiology.

The objective of this study was to identify protein biomarkers from SBMA blood samples. We found a panel of 40 proteins that were significantly different in patients with SBMA compared with matched controls. These proteins were reproducible across independent samples and were enriched for skeletal muscle processes aligning with the clinical manifestations and areas of pathology implicated in SBMA. We have illustrated the connections of these proteins with potential pathophysiological mechanisms in Figure 6. Acknowledging that this illustration does not capture the full expression patterns and molecular complexities of these proteins, it integrates our discovery with known biology. Additionally, our investigation revealed correlations between these SBMA-associated proteins and various clinical outcomes in patients, suggesting a potential link to pathogenic processes. Of note, many of these SBMA-associated proteins demonstrated more favorable characteristics than currently assessed biomarkers, including lower variability, stronger correlations with functional assessments, and more significant differences between disease and control groups.

While our findings offer insight into potentially new disease biomarkers, we acknowledge that the mechanisms underlying the altered levels of these proteins remain unclear. We have yet to determine whether increased protein levels result from transcriptional regulation or posttranslational processing or whether they are released into circulation due to muscle degeneration, a key component of SBMA. To bridge this gap, we integrated publicly available data sets that assessed AR gene regulation activity, such as ChIP-on-chip from myoblasts and RNA-Seq from *Ar*-KO mice, providing insights into potential mechanistic pathways. However, further analysis is necessary to determine whether these protein-encoding genes are direct targets of AR. Indeed, analysis of AR promoter/enhancer occupancy in myotubes could be informative, but analysis of AR in myofibers in adult intact skeletal muscle is missing. Additionally, myotubes do not reproduce the molecular architecture and physiology of the intact myofiber in the intact muscle. There are similar concerns in the *Ar*-KO mice, where the effect can be indirect, as loss of a transcription factor can result in network changes of coordinated control of gene expression by other transcription factors and coregulators of transcription.

Despite the elusive mechanisms underlying altered protein levels, our discoveries shed light on various sites of pathology and distinguishing features of SBMA. The majority of SBMA-associated proteins are comprised of genes highly expressed in skeletal muscle. Several of these proteins, including MB (myoglobin), CA3, and FABP3 are also increased in muscle degenerative disorders such as DMD and FSHD. Of note, MB levels are reported to be abnormally high in 100% of patients with SBMA but do not display abnormalities in patients with ALS (10). Interestingly, evidence suggests that MB has an ARE and is differentially expressed by *Ar* KO in mouse skeletal muscle tissue. Other SBMA-associated proteins highly expressed in muscle, such as KLHL41 and NEB, may also be altered in other diseases but are not detected due to differences in the detection method or other sample postprocessing techniques. Interestingly, mutations in KLHL41 and NEB are associated with subtypes of nemaline myopathy, further connecting these proteins to myodegeneration in SBMA (40, 41).

As previously mentioned, the proteins EDA2R and RGMA are intriguing biomarkers, given their known biology and their clinical and preclinical connections with SBMA. Increased EDA2R levels may reflect heighted inflammation, muscle atrophy, and aging as recently elucidated (35, 42, 43). RGMA may also be a valuable biomarker as it correlates with muscular fat content in the lower limbs of patients with SBMA, decreases over time, and is associated with premature death and low body weight in the AR113Q mouse model. While we have yet to determine whether altered levels of RGMA reflect a neuronal or muscle contribution to SBMA pathology, our data align with the previous conclusion that muscle biomarkers may be more revealing of disease status than neuronal biomarkers (11). Consistent with these observations, the NfL (gene name *NEFL*) marker of neurodegeneration did not exhibit differences between SBMA versus control (*P*_adj_ > 0.05). The SBMA proteomic signature appeared stable over time based on the limited longitudinal patient samples analyzed. Future studies with additional mouse models and patients that include presymptomatic and more longitudinal samples, extended follow-up assessments, and differing disease states will be needed to fully characterize longitudinal changes and identify proteomic biomarkers with wider dynamic ranges. It may be that the largest biomarker abnormalities precede the onset of symptoms by years, as is the case with Alzheimer’s disease (44).

A major advance in our work is identifying biomarkers that correlate with clinical outcomes. We found several muscle-enriched proteins that correlate with the SBMAFRS and the subscores of lower- or upper-limb test items that involve recruitment of large muscle groups. Consistent with previous studies, CK did not correlate with any functional readout in patients with SBMA (11, 45). Creatinine, on the other hand, showed correlations with SBMA muscle MRI and the SBMAFRS, aligning with published research (11, 46). While clinical labs like CK and creatinine remain useful in SBMA, our findings suggest that other protein biomarkers may hold greater relevance to the disease.

It should be noted that, while the proteomic technology platform used in this study detects upward of 3,000 proteins, it is well short of an entire proteome. Such is a limitation of this technology that, while it is highly sensitive, it is not unbiased and will only measure proteins for which there are antibodies. Whereas mass spectrometry offers unbiased discovery and higher specificity, it is not as sensitive as ligand-binding approaches. Additionally, aptamer-based approaches offer a complementary technology that can be highly sensitive and multiplexed. Ultimately, all technologies will advance and give a more complete picture of disease-specific proteomes.

In summary, our study reveals several promising protein biomarkers that merit further exploration in both clinical and preclinical settings. These discoveries offer potential keys to a deeper understanding of SBMA and may ultimately assist the research and development of effective therapies for patients.

## Methods

### Sex as a biological variable.

SBMA only affects males; therefore, only male samples, humans and mice, were collected and used.

### SBMA human samples.

The current study utilized 4 SBMA patient cohorts and 2 cohorts from a preclinical mouse model (Table 1). Demographic information on the human samples was unavailable. Two patient cohorts were used for discovery, and 2 were used for replication. One of the human discovery samples came from a NIH study published in 2017 (17) that also contained data on clinical labs and thigh muscle MRI data. Another human discovery sample came from an observational study at UCL that contained data on the SBMAFRS and 6MWT. One human replication sample came from a longitudinal observational study conducted by Nido Biosciences (study no. NDO-000-001) and consisted of only patients with SBMA. The other human replication sample came from UCL but used independent participants and was analyzed on a previous version of the proteomics platform including a reduced number of proteins (~1,500 versus ~3,000 in the other data sets).

### SBMA mouse model samples.

Longitudinal plasma samples were collected in 2 cohorts from WT and AR113Q male mice. C57BL/6J-hAR*113Q hemizygous (gift from A. Lieberman, University of Michigan, Ann Arbor, Michigan, USA) and C57BL/6J (The Jackson Laboratory) WT littermate male mice (approximately 3–5 weeks of age) were bred and genotyped at the Charles River Laboratory and were then shipped to Psychogenics. Mice were assigned unique identification numbers (ear notched) and were single-housed in polycarbonate cages with filter tops. All animals were examined, manipulated, and weighed prior to initiation of the study to assure adequate health and suitability and to minimize nonspecific stress associated with manipulation. Animals were body weighed weekly. In total, the study included 47 WT mice and 45 AR113Q mice at study start.

During the course of the study, 12:12 light/dark cycles were maintained. The room temperature was maintained between 20°C and 23°C with relative humidity maintained around 50%. Chow and water were provided ad libitum for the duration of the study. This line of mice does not show rapid or overt signs of morbidity or other clinically relevant changes immediately before death, although in rare cases extreme lethargy or immobility necessitated euthanasia. Euthanasia was deemed necessary when mice lost more than 10% of their body weight within 1 week. Most animals that died were found dead. The cause of death in this line of mice is yet to be fully understood.

Plasma was collected via submandibular bleeds every other week from 12–28 weeks of age. Briefly, animals were scruffed and a lancet was used to pierce the cheek. The animal was then held to a plasma K-EDTA tube and allowed to bleed into the tube. After collection, the bleeding was stopped by applying pressure with a sterile gauze pad to the cheek until blood stopped flowing. Animals were then given 1 mL Ringer’s solution and allowed to recover on heating pad before being returned to their home cage. Collection tubes were then left on wet ice for at no more than 15 minutes prior to centrifugation at 5,000*g* for 10 minutes at 4°C. The supernatant containing the plasma was then pipetted out and placed in a separate 1.5 mL Eppendorf tube and frozen on dry ice. At each time point, 50 μL of whole blood was collected to generate 25 μL of plasma.

### Public data set analyses.

To determine whether genes had a mechanistic link to AR, we used muscle (gastrocnemius) RNA-Seq data from female mice treated with DHT with a conditional KO of AR in muscle tissue (31). Briefly, 13-week-old female mice (*n* = 3 per group) were used that were treated with a biodegradable pellet of 10 mg DHT at 9 weeks of age. RNA-Seq data were aligned to reference genome mm10 using salmon (47) and STAR (48). Differential expression analyses explored differences between *Ar* KO DHT versus WT DHT by using a negative binomial regression model from DESeq2 (49). RNA-Seq data are available on the Gene Expression Omnibus (GEO; GSE152756).

To investigate possible ARE for proteins of interest, we leveraged published ChIP-on-chip data from primary human skeletal muscle myoblasts (30). Cells were treated with 30 nM of DHT or DMSO for 8 hours. IgG and AR ChIPs were performed on lysates, and probes were mapped to the hg18 reference genome. Active regions were defined as 10,000 bp up- or downstream from a gene, and enrichment of AR binding sites was performed using Tiling Analysis Software and the Microarray Analysis Tool. ChIP-on-chip data are available on GEO (GSE22076).

### Proximity extension assay.

Olink Proteomics has developed targeted immunoassays for protein biomarker research that can measure numerous proteins simultaneously in multiple matrices. Three platforms were used: Olink Explore 3072, Olink Explore 1536, and Olink Mouse Target 96, with the numbers indicating the approximate number of proteins measured in that platform (i.e*.,* Explore measures ~3,000 proteins and Target measures ~96 proteins). To identify a single protein (or antigen of the protein), antibodies are coupled to 1 of 2 complementary oligonucleotides, which will then hybridize to each other when brought into close proximity of the antibodies binding a specific protein. Then, either quantitative PCR (qPCR) or DNA sequencing is used to quantify the number of hybridized oligonucleotides, and those values are then converted to normalized protein values. The antibodies are multiplexed, allowing detection of several proteins at once.

The biosamples analyzed were kept frozen at Nido Biosciences from the time of receipt and storage, through shipment and storage at Olink. No additional freeze-thaw cycles were done since sample ascertainment by Nido Biosciences. Samples were thawed at 4°C at Olink before plating for analysis.

Each study was done with a set of technical controls predetermined by Olink. These controls were implemented on every 96-well plate, in which test samples were analyzed. After each sample set analysis, the data are scrutinized for quality control and are accompanied by an Analysis Report (available upon request).

### Statistics.

To determine protein abundance differences between SBMA and controls, linear regression analyses adjusted for age were performed. The UCL cohort had longitudinally assessed SBMA sample; thus, a linear mixed-effects regression was conducted using sample IDs as a random effect to account for repeated participants across time points using the lmerTest R package (50). The Nido Biosciences Observational Study did not have control samples. To determine proteomic associations with disease, Nido Biosciences patients with SBMA were compared with control samples from NIH and UCL discovery data sets using linear regression and adjusting for age and cohort. GSEA were also conducted (51) using the fgsea package in R (52) on over 3,000 gene sets with > 15 genes to test for enrichment from 7 categories of gene sets (Hallmark pathways, Kyoto Encyclopedia of Genes and Genomes [KEGG] pathways, Ingenuity pathways, Human Protein Atlas tissue expression,and Gene Ontology gene sets of biological processes, molecular functions, and cellular components).

Linear regression analyses were also conducted in mice to investigate protein abundance differences between AR113Q versus WT mice controlling for cohort. Analyses were conducted within time point, and results were metaanalyzed across time points using multilevel metaregression via the metafor package in R (53). Linear mixed-effects regression models adjusting for cohort were performed to identify protein correlations with body weight in AR113Q mice. Kaplan-Meier survival analysis was determined using GraphPad software (version 9), utilizing the Mantel-Cox method. Time-varying Cox proportional hazard ratio models adjusting for cohort were also fit to determine protein and body weight associations with rates of survival over time in AR113Q mice via the survival R package (54).

For human discovery data sets, correction for multiple testing was performed via a Benjamini-Hochberg adjustment using a threshold of *P*_adj_ < 0.05 to determine significance for differential protein abundance and significantly enriched gene sets. Replication and preclinical follow-up samples employed a nominal *P* value cut-off of *P* < 0.05.

### Study approval.

Participants were recruited under NIH protocols NCT02124057 and NCT04944940. The study was approved by the NIH Combined Neuroscience IRB and the NIH IRB, and informed consent was obtained from all participants. The UCL samples were collected on this study: *Characterisation of a panel of disease biomarkers in peripheral blood from individuals with amyotrophic lateral sclerosis/motor neuron disease* (Research Ethics Committee reference no. 09/H0703/27).

### Data availability.

The data supporting this study are available from the corresponding author upon reasonable request. A Supporting Data Values file has been provided with this manuscript.

## Author contributions

ANTN and SBH are co–first authors and equally contributed to study implementation, data analysis and interpretation, and manuscript organization. Co-first authorship order was decided by each author’s overall level of project management. ATNT oversaw the mouse studies and managing the service provider. VL, LZ, AA, AK, CG, AM, CR, and PF gathered participant data and materials and contributed to data discussions. VV oversaw study progress and implementation. ATNT, SBH, CG, PF, and VV edited the manuscript.

## Supplementary Material

Supplemental data

Supporting data values

## Figures and Tables

**Table 2:**
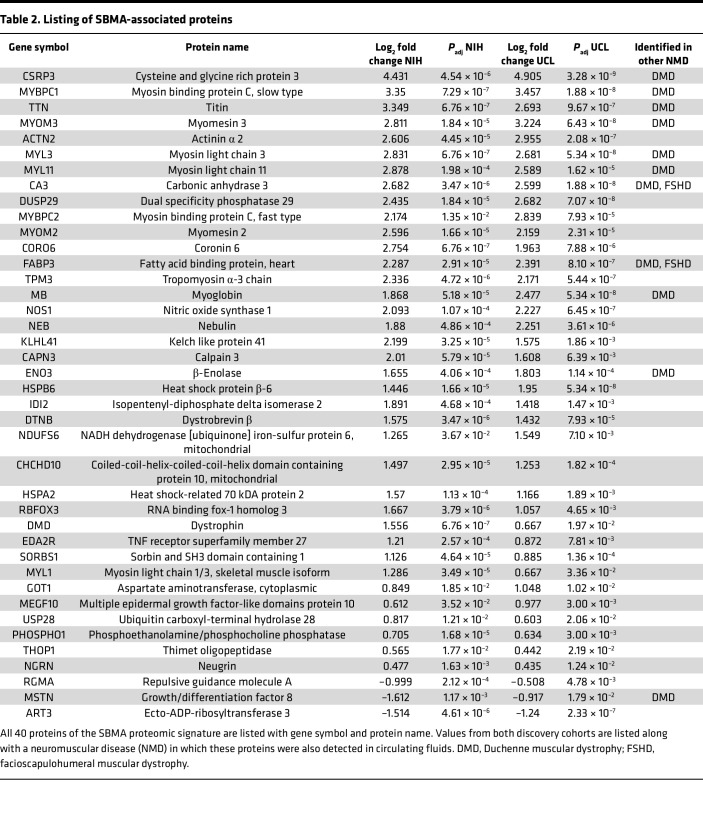
Listing of SBMA-associated proteins

**Table 1:**
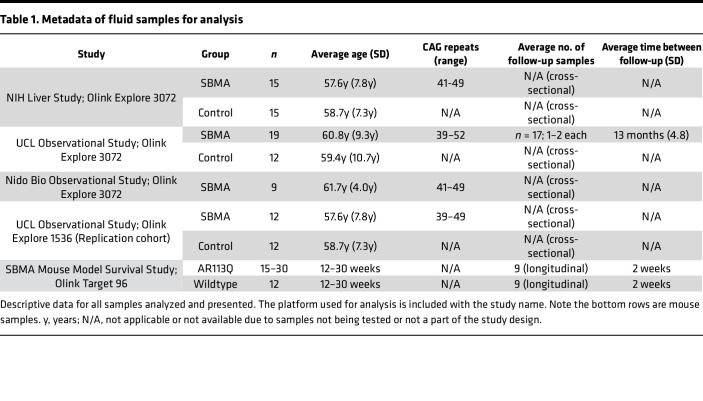
Metadata of fluid samples for analysis

## Version 1 07/08/2024

Electronic publication
