# Supplementary material for: Protein biomarker signature in patients with spinal and bulbar muscular atrophy
Source: JCI Insight. 2024 May 30;9(13):e176383. doi: 10.1172/jci.insight.176383 (PMC11383357; doi:10.1172/jci.insight.176383)
Supplement: Supplemental data [file jciinsight-9-176383-s089.pdf]

Supplemental Figures

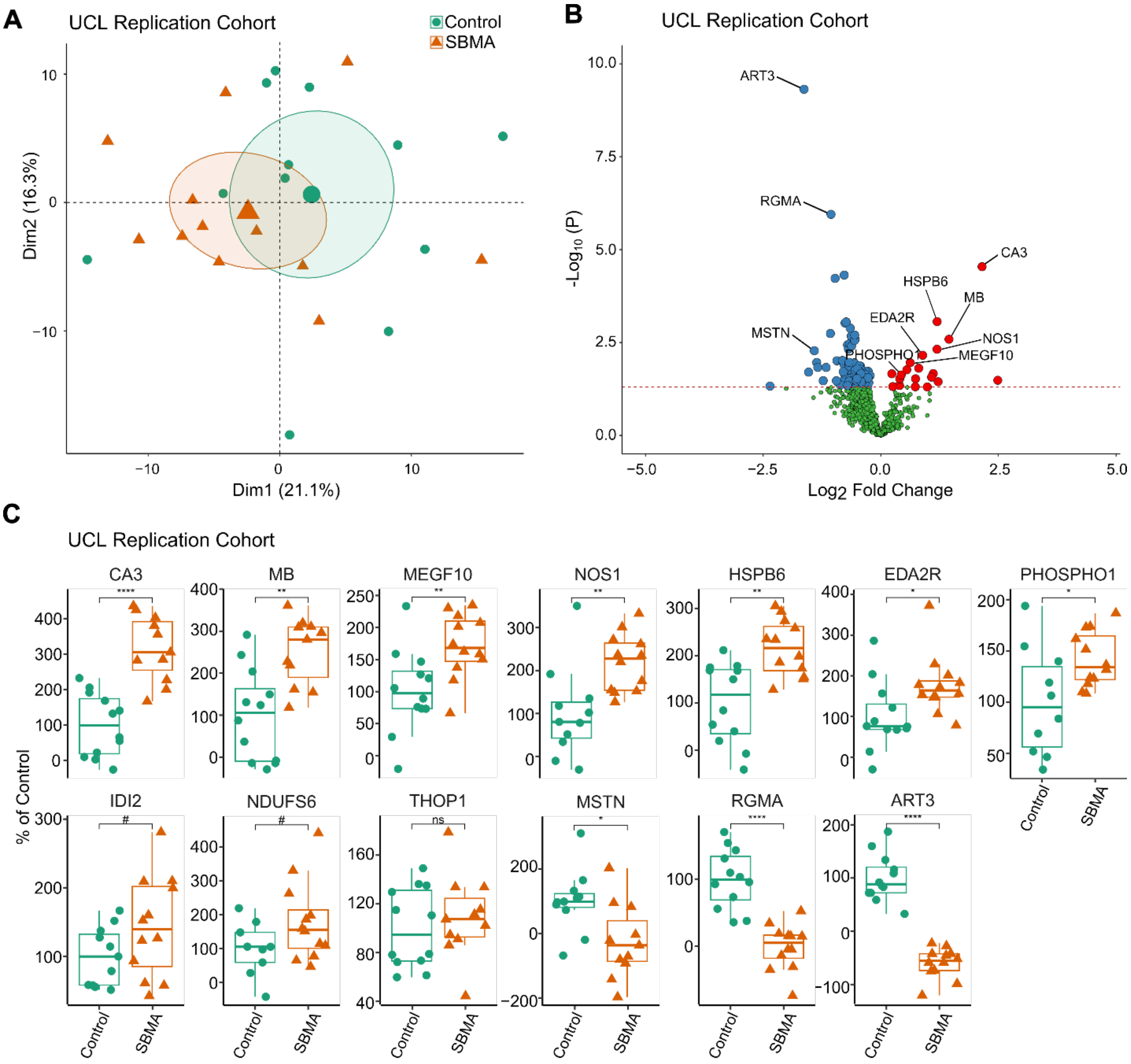

Supplemental Figure 1. A) PCA of SBMA and control samples analyzed on the Olink 1536 platform. B) Volcano plot of all proteins measured in these samples, with labeled proteins also altered in the two discovery cohorts (P <0.05 cut-off, dotted red line). C) Box and whisker plots showing differential abundance of the SBMA associated. The arbitrary protein abundance metric from Olink (NPX values) were transformed to % control for each protein, where 100% indicates the mean protein abundance for the healthy control group. The middle line in the boxes represents the median, the upper and lower hinges on boxes represent the 25th and 75th percentile. The upper and lower end of the whiskers show values that are 1.5* inter-quartile range. ns = not significant, # = p<0.1; * = p<0.05; ** = p<0.01; *** = p<0.001; **** = p < .0001 via t-test.

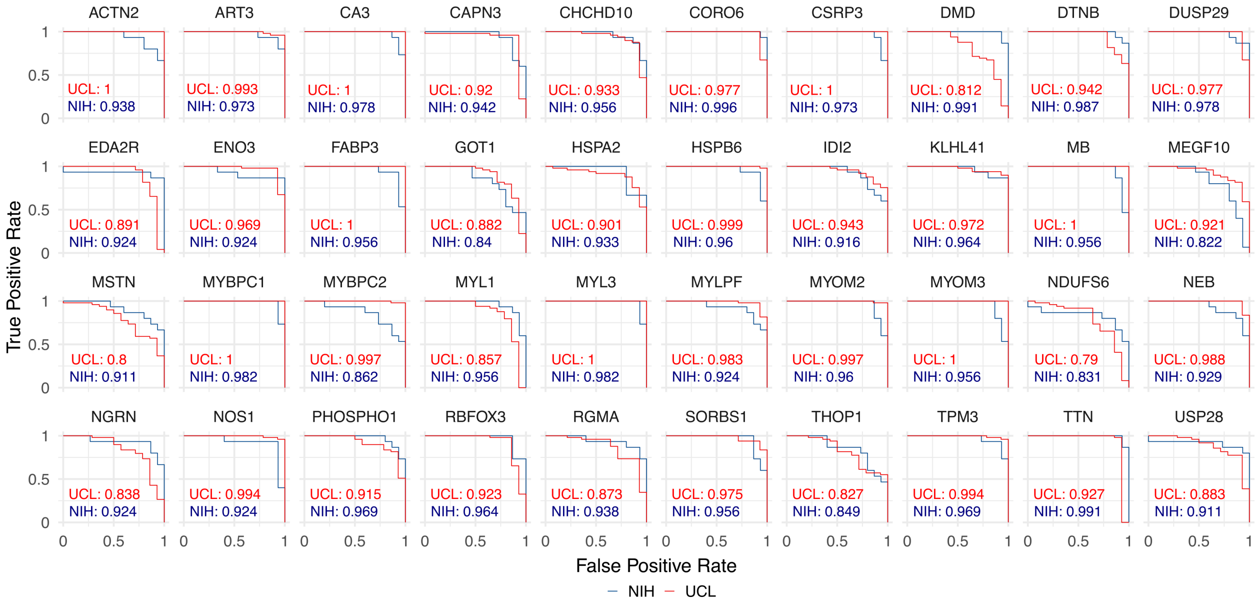

Supplemental Figure 2. ROC Curves of SBMA associated proteins. Each curve shows how the abundance of a specific protein distinguishes between SBMA versus control plotted as the true positive rate (sensitivity) against the false positive rate (1 – specificity). The Area Under the Curve (AUC) values are reported in the bottom left on the plot with 1 representing perfect distinguishing ability. Curves and AUC values in red represent samples in the UCL cohort, whereas blue represents those in the NIH cohort.

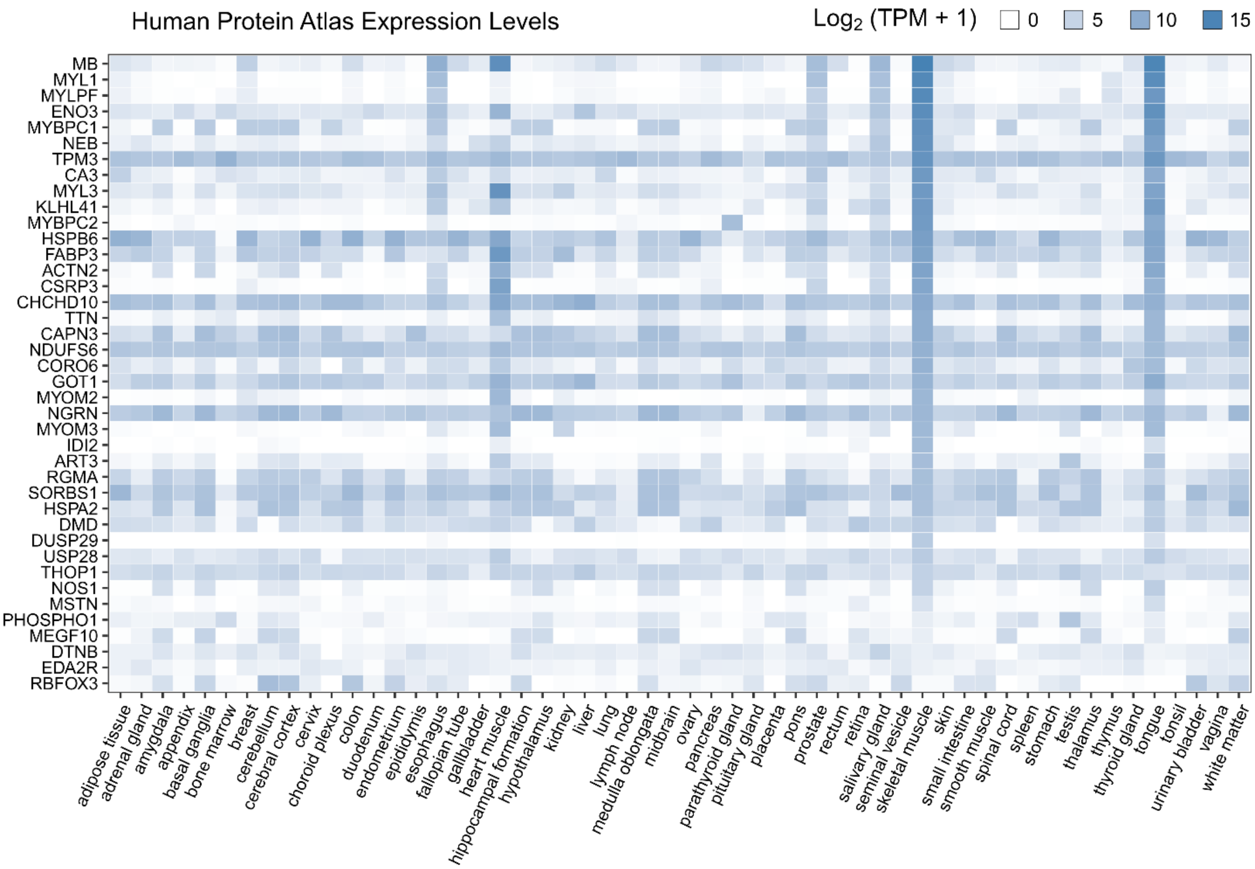

Supplemental Figure 3. Tissue-level expression of SBMA-associated proteins. Publicly available data from the Human Protein Atlas (https://www.proteinatlas.org/) shows the expression profile across 54 different tissues (x-axis) of the 40 SBMA-associated proteins (y-axis). Proteins were sorted based on expression in skeletal muscle. TPM = transcripts per million.

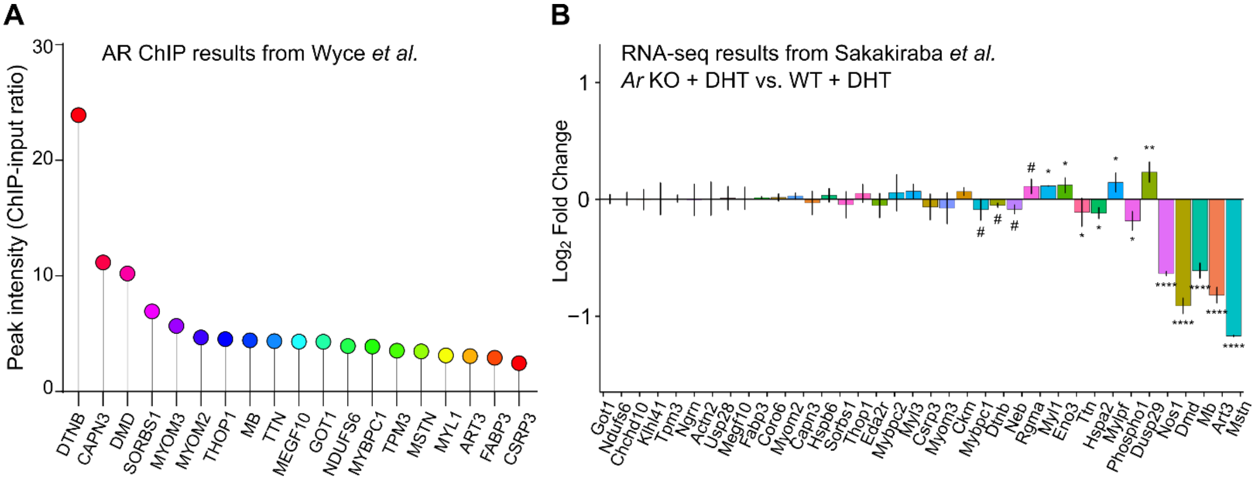

Supplemental Figure 4. Relationship between SBMA-associated proteins and AR function. A) Peak intensities from Chromatin immunoprecipitation of AR were plotted using data published in Wyce *et al.*, providing evidence of AR binding and regulating gene expression. Proteins not plotted did not have evidence of an ARE in that dataset. B) Log_2 fold change of genes measured in RNA-seq data from Sakakiraba *et al.* Differential expression results from DESeq2 differential expression are plotted # = p<0.1; * = p<0.05; ** = p<0.01; *** = p<0.001; **** = p < .0001.

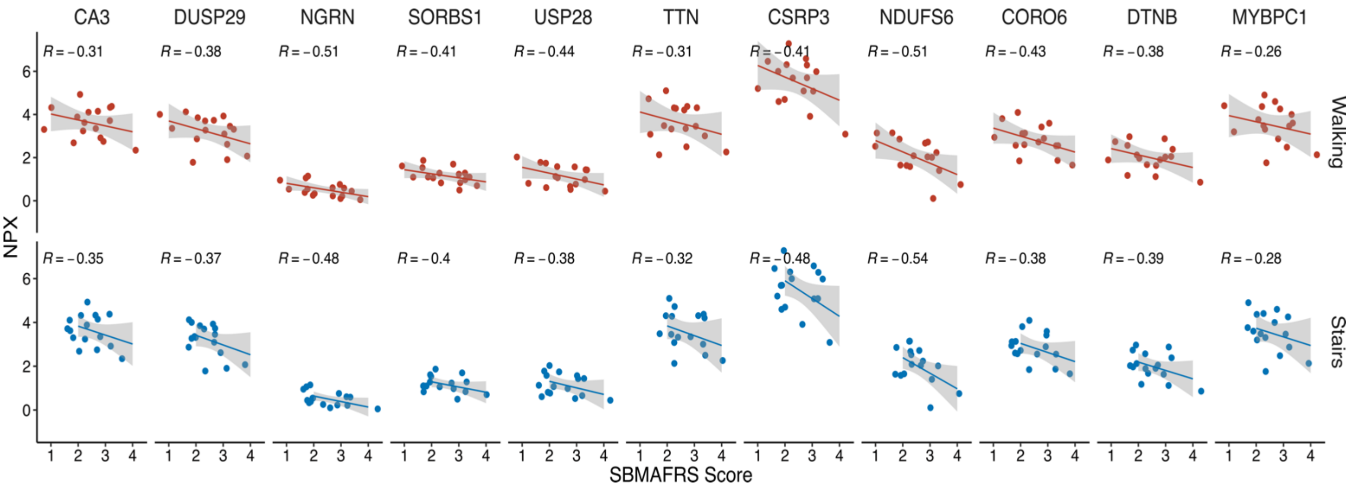

Supplemental Figure 5. SBMA Associated Proteins Correlate with SBMAFRS Lower-limb Impairment. Scatter plots show the 11 SBMA associated proteins that are significantly associated with both 'Walking' and '**S**tairs' items from the SBMAFRS in the UCL SBMA patients. Such that higher abundance of these proteins is correlated with lower scores in walking and stair SBMAFRS items. Y-axis shows NPX values, which are log2 normalized protein abundance metrics from the Olink platform and the x-axis shows SBMAFRS scores from lower-limb items that range from zero to four. Pearson product moment correlation coefficients are reported. Each point is a SBMA patient. Note, only baseline observations that had data on both protein abundance and SBMAFRS functional assessments were used.

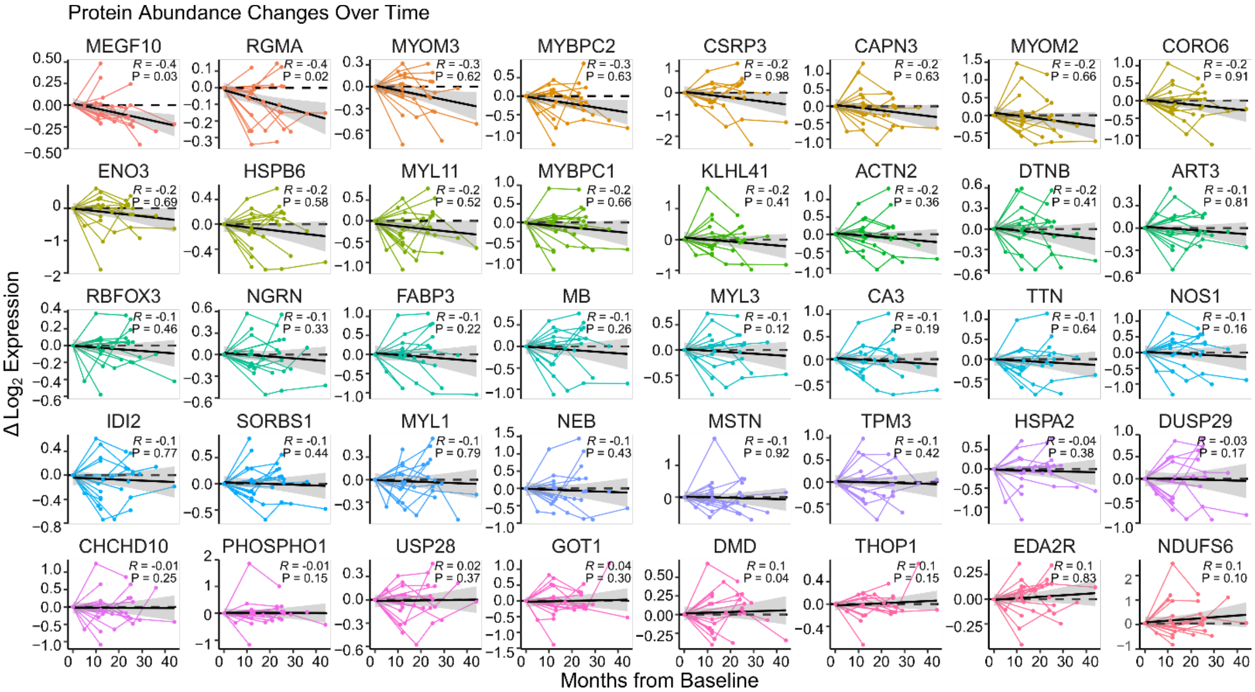

Supplemental Figure 6. Proteomic signature is stable over time. The 40 SBMA-associated proteins were plotted to show their correlation across time. Changes in log2 protein abundance are shown on the y-axis centered at 0 at baseline. Pearson product moment correlation coefficient reported in plot. Mixed-effects regression models covarying for age and log-transforming months from baseline were used to assess changes over time. Only RGMA and MEGF10 showed significant changes over time in these models (|*R*| > 0.3 and nominal P < 0.05).

Supplemental Table 1. SBMA vs Control - Differential Abundance Results

Protein	Study	Log2FoldChange	se	t	P	padj
CSRP3	UCL	4.90462181	0.410666158	11.94308739	1.12E-12	3.28E-09
MYBPC1	UCL	3.456529231	0.316300471	10.92799267	1.72E-11	1.88E-08
CA3	UCL	2.599117101	0.245262004	10.59730842	1.93E-11	1.88E-08
HSPB6	UCL	1.950376496	0.195658093	9.968289419	8.94E-11	5.34E-08
MYL3	UCL	2.681251823	0.263867829	10.16134418	1.02E-10	5.34E-08
MB	UCL	2.477151876	0.256542381	9.655916768	1.10E-10	5.34E-08
MYOM3	UCL	3.224362518	0.310990262	10.36804979	1.54E-10	6.43E-08
DUSP29	UCL	2.682369787	0.283515425	9.461107039	1.93E-10	7.07E-08
DMD	NIH	1.5563	0.169590397	9.176816759	6.19E-10	6.76E-07
ACTN2	UCL	2.955486338	0.322210717	9.172526497	6.40E-10	2.08E-07
ART3	UCL	-1.23966185	0.135258263	-9.165146914	7.97E-10	2.33E-07
TTN	NIH	3.34902	0.370783039	9.032290182	8.66E-10	6.76E-07
CORO6	NIH	2.753626667	0.306330312	8.989076698	9.58E-10	6.76E-07
MYL3	NIH	2.830873333	0.317527814	8.915355465	1.14E-09	6.76E-07
TNNI3	NIH	4.349533333	0.4882234	8.908899765	1.16E-09	6.76E-07
MYBPC1	NIH	3.349766667	0.380652727	8.800059558	1.49E-09	7.29E-07
TPM3	UCL	2.170893109	0.2483675	8.740648886	2.05E-09	5.44E-07
NOS1	UCL	2.226962897	0.265828614	8.377438622	2.65E-09	6.45E-07
FABP3	UCL	2.390766634	0.290128397	8.240374455	3.60E-09	8.10E-07
TTN	UCL	2.693316963	0.323006215	8.338282179	4.63E-09	9.67E-07
DTNB	NIH	1.57468	0.194915484	8.078783515	8.51E-09	3.47E-06
CA3	NIH	2.68156	0.333757137	8.034464884	9.49E-09	3.47E-06
RBFOX3	NIH	1.666953333	0.209653959	7.950974734	1.17E-08	3.79E-06
CSRP3	NIH	4.431433333	0.565548277	7.835641122	1.55E-08	4.54E-06
ART3	NIH	-1.51412	0.194315991	-7.792050418	1.73E-08	4.61E-06
NEB	UCL	2.251403868	0.298192849	7.550160488	1.85E-08	3.61E-06
TPM3	NIH	2.336266667	0.301540266	7.747776766	1.93E-08	4.72E-06
CORO6	UCL	1.962751881	0.263113328	7.459720482	4.32E-08	7.88E-06
HSPB6	NIH	1.446346667	0.201002062	7.195680755	7.85E-08	1.66E-05
MYOM2	NIH	2.59592	0.360990299	7.191107371	7.94E-08	1.66E-05
PHOSPHO1	NIH	0.705146667	0.098497248	7.159049422	8.62E-08	1.68E-05

Creatinine	NIH	-0.814208775	0.112663145	-7.226931018	8.99E-08	1.89E-05
MYLPF	UCL	2.58917951	0.363830967	7.116435231	9.44E-08	1.62E-05
DUSP29	NIH	2.43484	0.343056976	7.097479927	1.01E-07	1.84E-05
MYOM3	NIH	2.811086667	0.397307379	7.075344721	1.07E-07	1.84E-05
NEXN	UCL	-1.327750478	0.189575157	-7.003821068	1.25E-07	2.02E-05
MYOM2	UCL	2.158610997	0.314293535	6.868136814	1.50E-07	2.31E-05
FABP3	NIH	2.28702	0.332547593	6.877271243	1.79E-07	2.91E-05
CHCHD10	NIH	1.4968	0.218497878	6.850409767	1.92E-07	2.95E-05
KLHL41	NIH	2.198826667	0.323655055	6.793734977	2.22E-07	3.25E-05
MYL1	NIH	1.28584	0.19054309	6.74828986	2.51E-07	3.49E-05
CK	NIH	2.138177615	0.317812793	6.727789639	3.19E-07	4.46E-05
ACTN2	NIH	2.606306667	0.392609986	6.638411549	3.34E-07	4.45E-05
SORBS1	NIH	1.12634	0.170518962	6.605365113	3.65E-07	4.64E-05
MB	NIH	1.86792	0.285297756	6.547264945	4.25E-07	5.18E-05
ENPP2	NIH	0.872893333	0.134283743	6.500364936	4.81E-07	5.63E-05
CAPN3	NIH	2.010493333	0.310489111	6.475245872	5.14E-07	5.79E-05
DTNB	UCL	1.431956137	0.220892376	6.482596462	5.48E-07	7.93E-05
MYBPC2	UCL	2.838985934	0.438301397	6.47724592	5.70E-07	7.93E-05
PTGES2	NIH	1.294033333	0.201133855	6.43369231	5.74E-07	6.22E-05
GPD1	NIH	1.22696	0.195456057	6.277421217	8.70E-07	9.09E-05
ENO3	UCL	1.803401255	0.289891853	6.22094493	8.72E-07	0.000114314
DCTN1	UCL	-1.345101956	0.233090992	-5.770716178	8.99E-07	0.000114314
SPINT3	NIH	-3.514486667	0.56657374	-6.203052519	1.06E-06	0.000107015
NOS1	NIH	2.092926667	0.338082566	6.19057851	1.10E-06	0.000107015
SORBS1	UCL	0.88496612	0.148303257	5.967273669	1.11E-06	0.000135671
HSPA2	NIH	1.570046667	0.254982422	6.157470201	1.20E-06	0.000113157
SERPINH1	UCL	-2.718674824	0.47747075	-5.693908633	1.46E-06	0.00017071
TRIAP1	NIH	0.762813333	0.125800101	6.063694105	1.54E-06	0.00014096
ENPP2	UCL	0.655984132	0.106578083	6.154962775	1.56E-06	0.000175295
CHCHD10	UCL	1.25275638	0.213999358	5.854019343	1.68E-06	0.000181566
MYLPF	NIH	2.878286667	0.48572372	5.92576922	2.24E-06	0.000198104
RGMA	NIH	-0.999486667	0.169712568	-5.889290823	2.47E-06	0.000212157
EDA2R	NIH	1.20992	0.208318147	5.808039377	3.07E-06	0.000256675

PPP1R9B	UCL	-2.109840051	0.38627878	-5.46196209	3.75E-06	0.000391546
NUDT16	NIH	0.58818	0.103214955	5.698592818	4.13E-06	0.000335613
IMMT	NIH	0.728253333	0.128179684	5.681503577	4.33E-06	0.000342028
ENO3	NIH	1.6551	0.295110061	5.608416045	5.28E-06	0.00040609
CEP85	UCL	-1.121645484	0.223286417	-5.023348481	5.51E-06	0.000555273
SCAMP3	UCL	-1.383166724	0.264561071	-5.228156657	5.86E-06	0.000560369
CALCOCO1	UCL	-1.690366552	0.328927019	-5.139032227	5.94E-06	0.000560369
NT5C1A	NIH	0.78672	0.141827349	5.547026063	6.23E-06	0.000467513
IDI2	NIH	1.890966667	0.341486595	5.537455033	6.40E-06	0.000467845
BPIFB1	NIH	-1.260246667	0.228449363	-5.516525199	6.77E-06	0.000483172
NEB	NIH	1.879813333	0.341430205	5.505703087	6.98E-06	0.000485763
PSMD1	NIH	1.38778	0.253841833	5.467105169	7.75E-06	0.000527026
PPP1CC	UCL	-1.247411987	0.24151514	-5.164943223	8.71E-06	0.000795265
EDDM3B	NIH	-1.1803	0.22294722	-5.294078129	1.24E-05	0.000825477
SLMAP	NIH	1.258953333	0.241116668	5.221345109	1.51E-05	0.000972605
AFM	NIH	0.459926667	0.088238313	5.21232391	1.55E-05	0.000972605
DDAH1	NIH	0.894733333	0.171738816	5.209849216	1.56E-05	0.000972605
IDI2	UCL	1.418227227	0.272219744	5.20986173	1.65E-05	0.001465784
NGF	NIH	0.19694	0.038032225	5.178240236	1.70E-05	0.001038231
MSTN	NIH	-1.611813333	0.314320543	-5.127928695	1.95E-05	0.001166934
LRCH4	UCL	-1.06715908	0.222576713	-4.794567513	1.97E-05	0.001693469
KLHL41	UCL	1.574609059	0.313466923	5.02320641	2.23E-05	0.00185887
HSPA2	UCL	1.165938373	0.234295175	4.976365271	2.33E-05	0.001893091
ALT	NIH	0.947610025	0.188222277	5.034526417	2.78E-05	0.001510721
CREG1	NIH	0.609193333	0.122028034	4.992240841	2.83E-05	0.001625748
MAD1L1	NIH	0.8401	0.168644069	4.981497453	2.92E-05	0.001625748
NGRN	NIH	0.476933333	0.095795542	4.978658967	2.94E-05	0.001625748
LMNB2	NIH	0.549113333	0.110309412	4.977937279	2.95E-05	0.001625748
TRIM21	UCL	-0.842749697	0.177565363	-4.746137888	3.12E-05	0.002421499
ADAMTS8	NIH	-1.07378	0.216910172	-4.950344143	3.18E-05	0.001720693
WASHC3	UCL	-0.898340094	0.191440029	-4.692540518	3.21E-05	0.002421499
TAX1BP1	UCL	-0.801110687	0.170887938	-4.687929986	3.29E-05	0.002421499
STIP1	UCL	-0.86670538	0.182960062	-4.737128806	3.31E-05	0.002421499

KIF22	UCL	-2.501245227	0.521979771	-4.791843221	3.45E-05	0.002461951
FYB1	UCL	-1.914664877	0.415220394	-4.611201434	3.67E-05	0.002521236
EEF1D	UCL	-1.318560884	0.285435685	-4.619467545	3.71E-05	0.002521236
NDUFA5	NIH	0.613873333	0.125987045	4.872511567	3.93E-05	0.002090065
VASP	UCL	-1.125816088	0.242176719	-4.648737881	4.27E-05	0.002838633
PRKG1	UCL	-1.92946637	0.429129918	-4.496228968	4.69E-05	0.002994812
MEGF10	UCL	0.97710049	0.200252788	4.879335263	4.78E-05	0.002994812
PHOSPHO1	UCL	0.633524392	0.138424315	4.576684318	4.82E-05	0.002994812
TRIM58	UCL	-1.696747601	0.369177709	-4.596018558	4.92E-05	0.002994812
CRHBP	NIH	0.570793333	0.119368423	4.781778298	5.04E-05	0.002599285
COX6B1	NIH	0.828886667	0.173474702	4.778141465	5.09E-05	0.002599285
TMPRSS5	NIH	-0.68136	0.142743572	-4.773314766	5.15E-05	0.002599285
CDC42BPB	UCL	-1.603277119	0.361220604	-4.43849853	6.45E-05	0.003789945
AP1G2	UCL	-1.15721199	0.256304288	-4.51499271	6.48E-05	0.003789945
CD38	UCL	-0.551643329	0.119258737	-4.625600981	6.67E-05	0.003821767
SPACA5_SPACA5B	NIH	0.70538	0.150951201	4.672900875	6.78E-05	0.003361641
EIF2AK3	UCL	-1.065293623	0.236441612	-4.505525121	6.80E-05	0.003821767
IGSF9	NIH	1.604386667	0.344710921	4.65429602	7.13E-05	0.003477843
ADAMTSL2	NIH	0.620926667	0.133855967	4.638767197	7.44E-05	0.003568936
AP3S2	UCL	-0.642642601	0.144732128	-4.440220776	7.69E-05	0.004239025
TIMP3	NIH	1.618546667	0.350694582	4.615259971	7.94E-05	0.00374399
SDCCAG8	UCL	-1.738130659	0.400722345	-4.337493727	8.06E-05	0.004362054
WWP2	UCL	-0.678390407	0.154869632	-4.380396597	8.39E-05	0.00444872
ELAVL4	NIH	1.08304	0.235959132	4.589947375	8.50E-05	0.003947965
CORO1A	UCL	-1.050842894	0.234309017	-4.48485895	8.52E-05	0.00444872
FARSA	UCL	-0.663812664	0.148509836	-4.469822877	9.06E-05	0.004645242
RBFOX3	UCL	1.057194266	0.230472648	4.587070417	9.22E-05	0.004645242
MYOM1	UCL	0.561261486	0.129225354	4.343276828	9.41E-05	0.004660523
CRACR2A	UCL	-1.495429369	0.345828458	-4.324194068	9.59E-05	0.004672052
PAXX	NIH	0.480873333	0.10581428	4.544503208	9.62E-05	0.004398883
L1CAM	NIH	-0.433866667	0.095713597	-4.532967952	9.93E-05	0.004469524
RGMA	UCL	-0.507900585	0.110965643	-4.577097655	0.000101165	0.004777091
OPHN1	UCL	-1.649881122	0.388233932	-4.249708705	0.000101327	0.004777091

SLC39A5	NIH	0.951233333	0.211070424	4.506710677	0.000106688	0.004728206
JUN	NIH	0.919506667	0.204949322	4.486507493	0.000112722	0.004921057
SSNA1	NIH	0.753953333	0.16831555	4.479403921	0.000114923	0.004943368
S100A13	NIH	0.7208	0.161893151	4.452319286	0.000123714	0.005244386
GCC1	UCL	-1.793779538	0.425585948	-4.214846721	0.000124503	0.005698819
CLIP2	UCL	-1.93466431	0.459455046	-4.210780416	0.000124777	0.005698819
BIN2	UCL	-1.566368325	0.370424843	-4.228572553	0.000128344	0.005767559
TBC1D5	UCL	-1.029366867	0.237671243	-4.331053485	0.000130229	0.005767559
CACNB3	NIH	1.025473333	0.231644816	4.42692114	0.000132562	0.005539211
CDKN1A	UCL	-1.513219616	0.358658377	-4.219111308	0.000133888	0.005833801
MINK1	UCL	-1.538465367	0.368643612	-4.17331351	0.000135716	0.005833801
RBP5	NIH	1.059826667	0.240024801	4.415488156	0.000136748	0.005633621
EHBP1	UCL	-0.950770278	0.228487164	-4.161154012	0.00014086	0.00596717
AAMDC	NIH	0.73758	0.167546354	4.402244396	0.00014176	0.005697134
CTSD	NIH	0.701253333	0.159334351	4.401143439	0.000142185	0.005697134
VAV3	UCL	-1.466257479	0.353531641	-4.147457569	0.000146677	0.006124809
ERP44	NIH	0.31734	0.072451995	4.380003625	0.000150591	0.005930879
LRRN1	NIH	-0.735786667	0.168136203	-4.376134662	0.000152181	0.005930879
SF3B4	UCL	-0.846374274	0.204058921	-4.147695521	0.000153554	0.006321663
FGF21	NIH	2.03222	0.464877405	4.371518125	0.000154101	0.005930879
COQ7	NIH	0.445966667	0.102187186	4.364213207	0.000157189	0.005971132
CAPN3	UCL	1.607882484	0.36824204	4.366374036	0.000157475	0.00639304
USO1	UCL	-1.60302772	0.39084341	-4.101457718	0.000165219	0.006615568
ASGR1	NIH	0.423133333	0.097636778	4.333749461	0.000170738	0.006402682
DNAJA2	UCL	-1.551489437	0.374181461	-4.146355715	0.000170781	0.006745843
MAP2K1	NIH	0.773846667	0.179127869	4.320079672	0.000177188	0.006560446
EIF4G1	UCL	-1.517947322	0.367383461	-4.131779143	0.000180157	0.007021335
TJAP1	UCL	-1.10834725	0.271108643	-4.088203304	0.000183322	0.007050645
NDUFS6	UCL	1.548995715	0.358974755	4.31505473	0.000186994	0.007098472
BCR	UCL	-1.23272284	0.299511126	-4.11578313	0.000193274	0.007242818
IRAG2	UCL	-0.847169891	0.206122864	-4.110023878	0.000203856	0.007542685
NAGK	NIH	-0.560673333	0.131632038	-4.25939871	0.000208856	0.007636283
TNIP1	UCL	-1.255086293	0.311921566	-4.023724009	0.000211594	0.007731109

GIPC3	UCL	-1.529113303	0.382422125	-3.998495914	0.000218933	0.007810667
NFE2	UCL	-1.081348981	0.267266636	-4.045955746	0.000219116	0.007810667
EDA2R	UCL	0.871517217	0.203782916	4.276694212	0.000221788	0.007810667
FIS1	NIH	0.871166667	0.20569979	4.235136397	0.00022303	0.008053854
GYS1	UCL	-1.458326257	0.359325989	-4.058504822	0.000226157	0.007869732
VPS53	UCL	-0.942772428	0.2331372	-4.043852406	0.000230645	0.007931483
CNTN3	UCL	-0.478926943	0.11359572	-4.216065021	0.000234918	0.00798449
MTIF3	UCL	-1.101114548	0.274122261	-4.016873879	0.000243273	0.008173405
PTPRZ1	NIH	-0.482253333	0.115100925	-4.189830204	0.000252089	0.008983236
OGA	UCL	-0.821161644	0.206645272	-3.973774167	0.0002528	0.008396975
CA14	NIH	-0.75946	0.181441046	-4.185712195	0.000254909	0.008983236
NUB1	UCL	-0.891241815	0.223872998	-3.981015241	0.00026071	0.008562436
ITGB6	NIH	-0.65574	0.157224993	-4.170710956	0.000265446	0.009243222
TNFRSF12A	NIH	0.657113333	0.158390123	4.148701441	0.000281689	0.009693425
TWF2	UCL	-1.105870367	0.278846246	-3.965878625	0.000283592	0.009210422
UBE2B	NIH	0.53986	0.130599482	4.133707056	0.000293313	0.009976042
DRG2	UCL	-0.611757762	0.154472352	-3.960305869	0.000297604	0.0095593
YAP1	NIH	0.464446667	0.112903193	4.113671667	0.000309585	0.010408459
FABP4	NIH	0.950533333	0.231430165	4.107214526	0.000315017	0.010470719
HDGF	UCL	-1.361484233	0.336426328	-4.046901569	0.000321152	0.010157719
GOT1	UCL	1.048404848	0.25699518	4.079472812	0.000323184	0.010157719
TOM1L2	UCL	-1.164952308	0.301552998	-3.863176006	0.00032957	0.010248215
GRHPR	NIH	0.86646	0.211874425	4.089497821	0.000330406	0.01085886
DAB2	UCL	-1.194403526	0.301882058	-3.956523732	0.000339043	0.010431817
CD8A	NIH	1.042473333	0.255849152	4.074562399	0.000343954	0.011178501
KAZN	UCL	-1.398727066	0.361168293	-3.872784777	0.000346295	0.010543966
GM2A	NIH	0.50902	0.125439186	4.057902623	0.000359712	0.011562158
PDLIM7	UCL	-1.715099905	0.435913696	-3.934494194	0.000363554	0.010955347
CES1	NIH	1.613786667	0.398152416	4.053188184	0.000364298	0.011582313
PLSCR3	UCL	-0.632316807	0.160935835	-3.928999461	0.000378026	0.011275214
DNER	NIH	-0.421413333	0.10441325	-4.036013956	0.0003815	0.0119862
DSG4	NIH	0.809133333	0.200656928	4.032421613	0.000385198	0.0119862
TNNI3	UCL	1.35085363	0.338205492	3.994180051	0.00038767	0.011446061

USP28	NIH	0.816933333	0.202971573	4.024865748	0.000393091	0.012103058
SDK2	UCL	0.366176953	0.094330114	3.881866958	0.000394701	0.011458447
EVI5	UCL	-1.150214371	0.300921986	-3.822300884	0.00039593	0.011458447
ARHGEF12	UCL	-1.105520251	0.290343896	-3.80762354	0.000402102	0.011522992
RGCC	UCL	-1.201648855	0.314997301	-3.814790961	0.000407793	0.011572618
EFCAB14	NIH	0.30942	0.077260953	4.004869079	0.000414756	0.0126225
Glucose	NIH	0.23980448	0.059606098	4.023153491	0.000416264	0.012612381
ATG16L1	UCL	-1.01054241	0.264322803	-3.823137463	0.000417982	0.011747697
HIP1R	NIH	0.598106667	0.149473014	4.00143577	0.000418592	0.0126225
APPL2	UCL	-1.507816095	0.398514262	-3.783593809	0.00043044	0.011982641
NAP1L4	UCL	-0.777764114	0.203177512	-3.828002951	0.000448151	0.012357984
COL18A1	NIH	0.3392	0.085316319	3.975792702	0.000448369	0.013382434
ADAMTSL5	NIH	-0.64914	0.163509217	-3.970051436	0.000455315	0.0134525
NGRN	UCL	0.434996369	0.111565604	3.899018626	0.000456182	0.012424034
MYBPC2	NIH	2.173746667	0.548058156	3.966270081	0.000459948	0.013453488
BACH1	UCL	-0.623919051	0.166257075	-3.752736854	0.000460765	0.012424034
NEDD4L	UCL	-0.568113399	0.148169754	-3.834206274	0.000463298	0.012424034
PCBP2	NIH	0.737733333	0.186409614	3.957592731	0.000470756	0.013633268
CD14	NIH	0.668373333	0.169364352	3.946363726	0.000485112	0.013911294
EPHA1	NIH	0.53882	0.136835322	3.937725964	0.000496447	0.014098126
FKBP5	UCL	-1.53830089	0.406737645	-3.782047001	0.000515276	0.013655199
DGCR6	NIH	0.760133333	0.193785072	3.922558765	0.000516983	0.014540139
AXIN1	UCL	-1.252132095	0.333381661	-3.75585175	0.00052171	0.013655199
FRMD4B	UCL	-0.841865727	0.226743291	-3.71285837	0.000523223	0.013655199
CLEC4G	NIH	0.539446667	0.138344119	3.899310428	0.000550089	0.015323896
RBPMS2	UCL	-1.647953652	0.441336998	-3.734002951	0.000558206	0.014323385
HARS1	UCL	-0.914401932	0.243846231	-3.749912099	0.000558627	0.014323385
TNPO1	UCL	0.51049033	0.13610047	3.750834446	0.000566077	0.014388209
LEP	NIH	1.801173333	0.46428394	3.879465082	0.000579987	0.015981911
PFKFB2	UCL	-1.102105753	0.291614434	-3.779325114	0.000582371	0.014454509
CRELD1	NIH	0.614986667	0.158646105	3.876468739	0.000584637	0.015981911
TRIM5	UCL	-0.954444872	0.255886605	-3.729952461	0.000585717	0.014454509
AZI2	UCL	-0.757971479	0.202937224	-3.735004672	0.000590367	0.014454509

CMIP	UCL	-1.066985551	0.288668132	-3.696236034	0.000593235	0.014454509
DYNLT1	UCL	-0.744483567	0.196788067	-3.783174349	0.000593411	0.014454509
SEC31A	UCL	-1.087903209	0.294425539	-3.695002867	0.000616958	0.014903873
NARS1	NIH	0.441353333	0.114790768	3.84485042	0.000635994	0.01713155
MAVS	UCL	-1.248000418	0.338283939	-3.689209787	0.000636945	0.015034462
AP3B1	UCL	-0.978234376	0.261827357	-3.736180918	0.000642282	0.015034462
FXN	UCL	-1.086270338	0.28867478	-3.76295546	0.00064317	0.015034462
GIT1	UCL	-1.259518798	0.345010094	-3.650672316	0.000643735	0.015034462
TBCA	NIH	0.944526667	0.24596975	3.840011489	0.000644234	0.01713155
SERPINA5	NIH	0.431886667	0.112470656	3.839994185	0.000644263	0.01713155
SKAP2	UCL	-1.544039457	0.420074128	-3.675635684	0.000648576	0.015034462
GHR	NIH	0.531586667	0.138631527	3.83452941	0.000653695	0.017224898
HPSE	UCL	-1.425325172	0.385765662	-3.694795345	0.00065499	0.015034462
NFU1	UCL	-1.368141017	0.368858366	-3.709122909	0.000658368	0.015034462
HS6ST2	NIH	0.53458	0.139534213	3.831175082	0.000659552	0.017224898
DDHD2	UCL	-0.761816909	0.20902747	-3.644577963	0.000666255	0.015096626
LYN	UCL	-1.426154442	0.386075624	-3.693976916	0.000678678	0.015259822
PCBD1	NIH	0.714926667	0.187165061	3.81976563	0.000679858	0.017598103
MESD	UCL	-1.444332036	0.393533101	-3.670166583	0.000686164	0.015310371
THOP1	NIH	0.564526667	0.14814064	3.81074813	0.00069634	0.017737556
KLRB1	NIH	0.502493333	0.131881466	3.810189177	0.000697374	0.017737556
B4GALT1	NIH	0.405006667	0.106509807	3.80252936	0.000711701	0.017945916
DGKA	UCL	-0.63425194	0.174357618	-3.637649728	0.000712429	0.015775991
OTUD7B	NIH	0.423606667	0.111629278	3.794763104	0.000726522	0.01816304
PDLIM5	UCL	-1.37397809	0.378728727	-3.627868687	0.000731589	0.016078463
CTSZ	NIH	0.691693333	0.182474978	3.790620173	0.000734551	0.018208139
GDNF	UCL	-0.753017624	0.203648261	-3.69763837	0.000739308	0.016126851
FKBPL	UCL	-0.654592069	0.180071225	-3.635184179	0.000745819	0.016148356
MDH1	NIH	0.46414	0.122654274	3.784132293	0.000747299	0.018261223
SEMA3F	NIH	0.457666667	0.12097391	3.78318487	0.000749178	0.018261223
NIT2	NIH	0.7256	0.19215002	3.77621611	0.000763147	0.018314669
PLIN1	NIH	0.832973333	0.220605698	3.775846864	0.000763894	0.018314669
CC2D1A	UCL	-0.813505564	0.223504095	-3.639779233	0.000768627	0.016519832

GOT1	NIH	0.849073333	0.225252744	3.769425034	0.000777004	0.018477528
SPAG1	NIH	0.5819	0.154824876	3.758439944	0.000799941	0.018869575
PLA2G15	NIH	0.41304	0.110078864	3.752218971	0.000813223	0.01902941
MYL4	NIH	1.30796	0.349045759	3.74724507	0.000823997	0.019128493
RAB39B	UCL	-0.603771903	0.162467911	-3.716253249	0.000831096	0.017715101
CALCOCO2	UCL	-0.895774895	0.250677593	-3.573414299	0.000841473	0.017715101
RAB33A	UCL	-0.88009339	0.244017522	-3.606681121	0.000842422	0.017715101
MSTN	UCL	-0.916914713	0.247950959	-3.697968009	0.000858448	0.017921472
ANXA11	UCL	-1.33562091	0.373685183	-3.574187501	0.000864498	0.017921472
RARRES2	NIH	0.570753333	0.153213208	3.725222775	0.000873398	0.019969839
CALB2	NIH	1.09046	0.292740298	3.725008167	0.000873894	0.019969839
EIF4E	UCL	-1.449267146	0.40397891	-3.58748219	0.000892735	0.018376512
TBCC	NIH	0.550826667	0.148278408	3.714813737	0.000897744	0.020355822
BAG6	UCL	-0.395769492	0.111466411	-3.550571749	0.000910138	0.018603724
GPI	NIH	0.43886	0.118352885	3.708063385	0.000913885	0.020562412
PALM2	NIH	0.707393333	0.190924876	3.705087296	0.000921091	0.020566342
MDM1	UCL	-0.399568164	0.113286615	-3.527055364	0.000939312	0.019066737
AHSG	NIH	0.24902	0.067404545	3.694409623	0.000947403	0.020993599
FAM13A	UCL	-0.761373153	0.215336875	-3.535730487	0.000968083	0.019515215
HSPA1A	NIH	0.497193333	0.135181095	3.677979786	0.000989329	0.021757797
ADAMTS8	UCL	-0.514948584	0.139836716	-3.682499129	0.000993092	0.019696795
DAAM1	UCL	-0.88340992	0.250537731	-3.526055406	0.000999495	0.019696795
DMD	UCL	0.666601757	0.181473279	3.673277757	0.001004005	0.019696795
TRIM25	UCL	-1.33401981	0.376127316	-3.546724084	0.001010588	0.019696795
SPRY2	UCL	-1.018851011	0.285983708	-3.562619074	0.001010783	0.019696795
ABHD14B	NIH	0.69776	0.190194227	3.668670759	0.001013881	0.022046922
ARHGEF1	UCL	-1.221231954	0.346109688	-3.528453541	0.001021807	0.019779743
ERN1	NIH	0.625446667	0.170644812	3.665195897	0.001023197	0.022046922
ACSL1	NIH	0.8848	0.241452134	3.664494434	0.001025088	0.022046922
INPPL1	UCL	-1.14303549	0.326308978	-3.502923818	0.001050557	0.020199541
ROBO1	UCL	0.42905961	0.116637756	3.678565363	0.001057314	0.020199541
MYH7B	NIH	0.707133333	0.193836756	3.648086907	0.001070296	0.022851219
PLEKHO1	UCL	-0.911902566	0.259896804	-3.508710194	0.001082719	0.02048011

EFHD1	NIH	0.42102	0.115582118	3.642604998	0.00108583	0.023014867
ABRAXAS2	UCL	-0.684768256	0.195829678	-3.496754244	0.001086013	0.02048011
RIDA	NIH	0.835913333	0.229811409	3.637388316	0.001100815	0.023164634
USP28	UCL	0.60276478	0.166238008	3.625914351	0.001102553	0.020604808
CEP20	UCL	-0.804853395	0.231104881	-3.482632613	0.001107556	0.020604808
LDLR	NIH	0.952013333	0.261981942	3.633889142	0.001110979	0.023211535
MEPE	UCL	-0.304072783	0.08410304	-3.615479083	0.001118251	0.020604808
YARS1	UCL	-2.345459278	0.670139269	-3.499957973	0.001120823	0.020604808
AMOT	NIH	0.855446667	0.235924743	3.625930275	0.00113444	0.02341718
MPI	NIH	0.504773333	0.139330544	3.622847644	0.001143656	0.02341718
EIF4G3	UCL	-0.773242133	0.221598041	-3.489390645	0.001144237	0.020903785
TP53I3	NIH	0.57104	0.157639026	3.622453232	0.00114484	0.02341718
PPP1R12A	UCL	-1.167044551	0.331834518	-3.516947424	0.001151982	0.02091456
PLIN3	NIH	0.450726667	0.124769439	3.612476513	0.001175199	0.023871224
MINDY1	UCL	-1.579409265	0.448487307	-3.521636486	0.001180149	0.021293669
SELPLG	NIH	0.420213333	0.116644485	3.602513526	0.001206297	0.024325251
VPS37A	UCL	-1.035364792	0.293451098	-3.52823622	0.001210627	0.021709593
LPL	NIH	-0.746893333	0.207468834	-3.600026666	0.001214183	0.024325251
THOP1	UCL	0.442237546	0.124069806	3.564425233	0.001235573	0.021940237
CETN3	UCL	-0.773582264	0.223713695	-3.457911967	0.001241509	0.021940237
HHEX	UCL	-0.859062549	0.248181215	-3.461432605	0.001246007	0.021940237
PREB	NIH	0.64122	0.178642	3.589413468	0.001248408	0.024727562
IMPA1	NIH	0.343326667	0.095735721	3.586191897	0.001258981	0.024727562
C1RL	NIH	0.28064	0.078259984	3.585996143	0.001259626	0.024727562
LARP1	NIH	0.769073333	0.214673431	3.582526864	0.001271115	0.024786748
BAG3	UCL	0.498113721	0.139263542	3.576770447	0.001276903	0.022349632
NUDT5	NIH	0.524686667	0.146724442	3.576000419	0.001293005	0.025046626
CAMSAP1	UCL	-1.599343974	0.46584999	-3.433173789	0.001329077	0.023064642
ABL1	UCL	-0.573352933	0.164452533	-3.486434191	0.001333536	0.023064642
CD4	NIH	0.422426667	0.119167959	3.544800707	0.001402815	0.026994954
SMTN	UCL	-1.256382609	0.359500067	-3.494804941	0.001403712	0.024135584
GAPDH	NIH	0.271686667	0.0767681	3.539056805	0.001423995	0.027052983
DCUN1D1	NIH	0.672233333	0.189991554	3.538227464	0.001427079	0.027052983

ENPP6	NIH	-0.517026667	0.146229695	-3.535715957	0.001436457	0.027052983
CTRL	NIH	-1.18972	0.336647843	-3.534019371	0.001442826	0.027052983
TP73	UCL	-0.469785445	0.140379769	-3.34653239	0.001466654	0.025070345
AST	NIH	0.593952591	0.168005917	3.535307579	0.001491551	0.027921447
MECR	UCL	-1.105604974	0.331425231	-3.335910694	0.001514102	0.025730934
METAP1D	UCL	-1.040548496	0.306818267	-3.391416378	0.001525204	0.025769779
UFD1	NIH	0.731086667	0.208312803	3.509561858	0.001537741	0.028485131
RABEP1	UCL	-0.782618103	0.232615922	-3.364421904	0.001544794	0.025950765
CLMP	NIH	0.319166667	0.090997326	3.507429088	0.001546299	0.028485131
SFRP4	NIH	0.6198	0.176737206	3.506901648	0.001548422	0.028485131
OSMR	UCL	0.287836261	0.081437567	3.5344408	0.001581269	0.025990428
NUDT5	UCL	-0.579971913	0.170534042	-3.400915777	0.001584507	0.025990428
GPC1	UCL	-0.401222878	0.114526959	-3.503305082	0.001584901	0.025990428
SULT1A1	UCL	-1.651837217	0.491071508	-3.363740698	0.001584935	0.025990428
GSTA1	NIH	1.284986667	0.367409088	3.497427549	0.001587053	0.028972534
WASF1	UCL	-0.731273105	0.217027176	-3.36950017	0.001591614	0.025990428
ADA2	NIH	0.56858	0.162678247	3.495120039	0.001596602	0.028972534
GATD3	NIH	0.712193333	0.203880512	3.493189838	0.001604633	0.028972534
CTSF	NIH	0.539106667	0.154462939	3.49020075	0.001617146	0.029019341
IST1	UCL	-1.430757972	0.423025932	-3.382199204	0.001629983	0.026469114
CSF2	NIH	0.477553333	0.13725156	3.479401868	0.001663148	0.029662847
GMPR	UCL	-0.812918681	0.239256089	-3.397692754	0.001678196	0.026917436
EIF4B	UCL	-1.150746142	0.343273202	-3.352274913	0.001679385	0.026917436
TBC1D23	UCL	-0.592488087	0.175149939	-3.382747891	0.001685218	0.026917436
GGT5	NIH	0.3592	0.103428814	3.472920043	0.001691366	0.029983298
UBAC1	NIH	0.796166667	0.230322095	3.456753321	0.001763781	0.031078678
FTCD	NIH	1.300486667	0.376948336	3.45003955	0.001794729	0.031434622
F9	UCL	-0.22960273	0.066460624	-3.454718249	0.001803448	0.028649342
LAT	UCL	-1.375638518	0.412367643	-3.335951649	0.001815402	0.028683353
TARBP2	UCL	-1.065843459	0.320920275	-3.32120948	0.001844484	0.028879979
IPCEF1	UCL	-1.121334235	0.33920178	-3.305802921	0.001847607	0.028879979
SLITRK2	NIH	0.66266	0.193035662	3.432837194	0.001876442	0.032670194
SNX9	UCL	-0.81403077	0.242557005	-3.356039001	0.001879937	0.02913889

APRT	UCL	-0.924912129	0.279685371	-3.30697357	0.001884109	0.02913889
UPB1	NIH	1.262533333	0.368054532	3.430288782	0.001888849	0.032691622
IL6R	NIH	0.463966667	0.135532336	3.423291307	0.001923327	0.033092539
AMFR	NIH	0.548693333	0.160477582	3.419127622	0.00194413	0.03325486
PSTPIP2	UCL	-1.517390961	0.459771286	-3.300316931	0.001950267	0.029829876
PAGR1	UCL	-0.385115367	0.118512649	-3.249571833	0.001957318	0.029829876
ACOT13	UCL	-1.390315285	0.4198929	-3.311118821	0.001959403	0.029829876
SRC	UCL	-1.500568737	0.455434665	-3.294805718	0.001976469	0.029859292
DARS1	UCL	-1.129147754	0.342461055	-3.297156679	0.001981766	0.029859292
APLP1	NIH	-0.5151	0.15129781	-3.404543664	0.002018726	0.034330085
PDGFC	NIH	0.376213333	0.110586317	3.401988079	0.00203208	0.034347468
ELAC1	UCL	-1.106074475	0.337229733	-3.279884204	0.002039605	0.030428019
NFX1	UCL	-0.847648584	0.260031749	-3.259788805	0.002040438	0.030428019
CDC37	NIH	0.546246667	0.160667126	3.399865802	0.002043234	0.034347468
RILP	UCL	-0.847326331	0.25607211	-3.308936419	0.002050742	0.030428019
TBL1X	UCL	-1.145618211	0.350378703	-3.269657091	0.002078633	0.03068608
MEGF10	NIH	0.611646667	0.180521694	3.388216969	0.002105519	0.035192245
NAA80	UCL	-1.404761456	0.431242846	-3.257471909	0.002115208	0.031069112
CD59	UCL	-0.25758063	0.07625184	-3.378025098	0.002134501	0.031195726
USP25	UCL	-0.913710296	0.277055503	-3.297932312	0.002159726	0.031305478
HGS	UCL	-1.882947196	0.546339318	-3.446479384	0.00216343	0.031305478
FGF2	UCL	-0.543029494	0.163413232	-3.323044819	0.002188914	0.031518202
SGSH	NIH	0.623733333	0.184994699	3.371628134	0.002197392	0.036519162
HMGCL	UCL	-0.631537362	0.197019385	-3.205457987	0.002228496	0.031930848
TNFRSF1A	NIH	0.354306667	0.105338638	3.3635015	0.002243801	0.036735053
PECR	UCL	-0.246857257	0.073001037	-3.381558223	0.002246687	0.032034475
ASGR2	NIH	0.556826667	0.165709252	3.360262981	0.002262557	0.036735053
NDUFS6	NIH	1.2646	0.376387012	3.359839634	0.00226502	0.036735053
BPIFB2	NIH	1.2662	0.376871805	3.359763146	0.002265465	0.036735053
PHLDB1	NIH	0.453153333	0.134929683	3.358440666	0.002273178	0.036735053
RTN4IP1	UCL	-1.066148058	0.332009923	-3.211193352	0.002284211	0.032275619
RRAS	UCL	-1.176677621	0.364303063	-3.229941609	0.002285684	0.032275619
MRPL24	NIH	0.548333333	0.163422584	3.355309411	0.002291539	0.036828312

PIBF1	UCL	-1.32578958	0.401666768	-3.300720116	0.002298773	0.032304395
ALPP	NIH	-1.655793333	0.494225149	-3.350281416	0.002321323	0.036982201
HHEX	NIH	0.709506667	0.211829056	3.349430333	0.002326402	0.036982201
TDRKH	UCL	-1.145363109	0.349597004	-3.276238347	0.00233329	0.032632562
SSNA1	UCL	-0.596653259	0.181268621	-3.291541893	0.002345181	0.032642687
BID	UCL	-0.632433344	0.194687773	-3.248449212	0.002361392	0.032712548
ENO1	UCL	-0.993475588	0.30765646	-3.229171872	0.002383835	0.032767051
DFFA	UCL	-0.755124107	0.233563431	-3.233057943	0.002387746	0.032767051
SAMD9L	UCL	-0.791119585	0.243708602	-3.246170139	0.00240712	0.032878555
C1R	NIH	0.284226667	0.085227117	3.334932333	0.002414574	0.038176365
SH2B3	UCL	-1.334176651	0.415628329	-3.210023378	0.002424682	0.032964393
MTHFSD	NIH	0.496813333	0.149165167	3.330625673	0.00244138	0.03839267
TRIM24	UCL	-0.738558282	0.216807535	-3.406515743	0.002491171	0.033585025
SETMAR	UCL	0.419720932	0.12604613	3.329899385	0.002493312	0.033585025
CD99L2	NIH	0.284586667	0.085681451	3.321450138	0.002499452	0.039095713
MYL1	UCL	0.667374615	0.199763327	3.340826487	0.002510035	0.033612596
GMFG	UCL	-0.891841957	0.276773109	-3.222285432	0.002518357	0.033612596
CTBS	NIH	0.383813333	0.115707069	3.317112213	0.002527369	0.039322092
NUP50	NIH	0.413106667	0.12468604	3.313174975	0.002552966	0.039380814
NAGPA	NIH	0.259606667	0.078422119	3.31037556	0.002571318	0.039380814
PCSK9	NIH	0.498853333	0.15069537	3.310342795	0.002571533	0.039380814
YTHDF3	UCL	-0.717012378	0.226024564	-3.172276351	0.002596583	0.03442358
REEP4	UCL	-0.715747805	0.227060034	-3.152240367	0.002602672	0.03442358
CMC1	UCL	-0.958223432	0.300340113	-3.190461047	0.002647045	0.034852757
PTPN1	UCL	-0.974380348	0.303928885	-3.205948485	0.002680997	0.034951807
ESYT2	UCL	-1.075092639	0.33838078	-3.177168153	0.002703269	0.034951807
RHOC	UCL	-1.429295138	0.448989921	-3.183356845	0.002704837	0.034951807
TXLNA	UCL	-1.133780348	0.355185148	-3.192082647	0.002713247	0.034951807
GGCT	UCL	-0.610431995	0.191991619	-3.179472093	0.002714355	0.034951807
BRAP	UCL	-1.285171861	0.404924701	-3.173853946	0.002741901	0.03503832
DNM1	UCL	-1.509636111	0.474034898	-3.184651841	0.002749059	0.03503832
DBNL	UCL	-1.166108418	0.36737004	-3.174206634	0.00275969	0.03503832
GIGYF2	UCL	-0.80722303	0.249933683	-3.229748864	0.002769022	0.03503832

TGFBR2	NIH	0.382193333	0.116672803	3.275770568	0.002808929	0.042792277
DOK2	UCL	-1.196386997	0.378876658	-3.157721578	0.002817912	0.03537341
DLG4	UCL	-0.668915085	0.211308481	-3.165585607	0.002819707	0.03537341
MAX	NIH	0.859106667	0.262535317	3.272347043	0.002833553	0.042943739
GBP4	UCL	-0.304558555	0.0969488	-3.141437066	0.002841399	0.035493196
CASP3	UCL	-1.17089687	0.37062978	-3.15920882	0.002859679	0.035569542
HGS	NIH	0.554006667	0.169552729	3.267459453	0.002869067	0.043257838
TACC3	UCL	-1.324317121	0.419130384	-3.159678163	0.002879699	0.03566678
IGFBP4	NIH	0.58608	0.179692846	3.261565578	0.002912462	0.043686934
PKD2	UCL	-0.478984177	0.148399232	-3.227672873	0.002915273	0.035955032
ITPRIP	UCL	-0.483924448	0.155642769	-3.109199679	0.002947627	0.036201324
CASP8	UCL	-0.680018918	0.215222231	-3.159612813	0.002960079	0.036202134
DNAJC6	NIH	0.8219	0.252504136	3.254996186	0.002961575	0.044196975
SH2D1A	NIH	0.819	0.252005989	3.249922758	0.003000048	0.044543856
ATP1B4	UCL	-0.423704089	0.136651508	-3.100617726	0.003021344	0.036797451
CHMP6	NIH	0.419566667	0.129315925	3.244508867	0.003041632	0.044875903
GLRX	NIH	0.709293333	0.218713234	3.243028882	0.003053096	0.044875903
AARSD1	UCL	-0.727373316	0.227941242	-3.191056207	0.003122114	0.037866963
NARS1	UCL	-0.50434681	0.160851616	-3.135478661	0.0031718	0.03831063
CERT	UCL	-1.263196521	0.404302953	-3.124381137	0.003211397	0.038601689
COL3A1	NIH	0.351646667	0.109184294	3.22067078	0.00323141	0.04725937
CIAPIN1	UCL	-0.779722676	0.249410076	-3.126267744	0.003233068	0.038601689
FGD3	UCL	-0.836430246	0.269260233	-3.106400961	0.003235516	0.038601689
CEP170	UCL	-0.846113005	0.269795867	-3.136122924	0.003254489	0.038670208
MAMDC4	NIH	1.09048	0.338918884	3.217525053	0.003257287	0.047400815
Insulin	NIH	1.005722579	0.311920732	3.224288981	0.003292703	0.04501048
CPA4	NIH	0.585673333	0.182359809	3.211636031	0.003306266	0.047642246
SERPINF2	NIH	0.123473333	0.03845213	3.211092167	0.003310824	0.047642246
STXBP1	UCL	-1.035463283	0.331179589	-3.126591486	0.003312964	0.039201329
ADAM22	NIH	0.507826667	0.158228225	3.209456882	0.003324568	0.047642246
SARG	UCL	-1.134688982	0.366257052	-3.09806726	0.003326011	0.039201329
COL6A3	NIH	0.518893333	0.161838709	3.206237467	0.003351786	0.047642246
SIGLEC8	NIH	0.83942	0.261842395	3.205821582	0.003355317	0.047642246

SIL1	NIH	0.4699	0.146687457	3.203409546	0.00337587	0.047682252
PRG3	NIH	0.678613333	0.211955829	3.201673365	0.003390738	0.047682252
PPP2R5A	UCL	-0.930546086	0.299407703	-3.1079564	0.003473071	0.04065168
EREG	UCL	-0.635174928	0.204048996	-3.112854955	0.00347688	0.04065168
ARG1	NIH	0.577446667	0.18097022	3.190838063	0.003484958	0.048772737
FST	NIH	0.439986667	0.138148895	3.184872852	0.003537895	0.049277829
BECN1	UCL	-0.561872674	0.182791125	-3.073850957	0.003544688	0.041221212
LAT2	UCL	-1.301251319	0.418638423	-3.108294052	0.003553796	0.041221212
RILPL2	UCL	-1.206357433	0.39248814	-3.073614994	0.00360046	0.041597406
IVD	NIH	2.9914	0.941739803	3.176461258	0.003613854	0.05006379
CDH3	NIH	0.524193333	0.165107913	3.174852884	0.003628555	0.05006379
ZFYVE19	UCL	-1.049300406	0.34025761	-3.08384111	0.003655255	0.042022929
UFD1	UCL	-1.648748142	0.534071284	-3.087131238	0.003669035	0.042022929
BAG3	NIH	0.418713333	0.132093434	3.169827005	0.003674863	0.050464664
NIT1	UCL	-0.72928798	0.238202203	-3.061634069	0.003680421	0.042022929
ABL1	NIH	0.50466	0.159513522	3.16374432	0.003731665	0.050567708
SUSD4	NIH	0.516806667	0.163377815	3.163260976	0.003736214	0.050567708
RNASE4	NIH	0.478873333	0.1514254	3.162437291	0.00374398	0.050567708
GSTA3	NIH	1.2378	0.39150578	3.161639143	0.003751519	0.050567708
HPCAL1	UCL	-1.087486787	0.354575188	-3.067013211	0.003790727	0.043113988
KCTD5	NIH	0.47848	0.151591272	3.156382249	0.003801539	0.051006893
CEACAM5	NIH	-0.931413333	0.295429635	-3.152741717	0.003836553	0.051241636
PNMA1	UCL	-0.762628631	0.246900336	-3.088811635	0.003864988	0.043788219
PAG1	UCL	-0.523417497	0.168934471	-3.09834633	0.003891307	0.04391618
PTS	NIH	0.900573333	0.286181188	3.146864192	0.003893734	0.051768969
MAP2	UCL	-0.432458785	0.139513422	-3.099764731	0.003931581	0.044200038
TIMP1	NIH	0.265513333	0.08457073	3.139541698	0.003966115	0.052492694
DECR1	UCL	-1.202666023	0.390793229	-3.077499643	0.004043854	0.045288063
ATXN3	UCL	-0.673574897	0.221816563	-3.036630297	0.004068115	0.045305902
PTPN6	UCL	-1.406695146	0.463981358	-3.03179238	0.004076446	0.045305902
HCLS1	UCL	-1.006340663	0.329364852	-3.055397861	0.004101942	0.045416577
FAM172A	UCL	-0.60073423	0.198000429	-3.034004697	0.004123795	0.045486238
BMPER	NIH	0.322026667	0.103093286	3.123643453	0.004127719	0.054188292

AK2	NIH	0.829686667	0.265830955	3.121106293	0.004154084	0.054188292
MATN2	NIH	0.290953333	0.093245077	3.120307706	0.004162416	0.054188292
TIMM8A	NIH	0.34212	0.109663174	3.119734606	0.004168406	0.054188292
ADH1B	NIH	0.908546667	0.291390067	3.117974045	0.004186856	0.054188292
AHCY	UCL	-0.596465387	0.197666016	-3.017541394	0.004224596	0.046373594
PDZK1	NIH	0.796706667	0.255835386	3.114137878	0.004227329	0.054471091
MIF	UCL	-0.945314176	0.310604442	-3.043466375	0.004251919	0.046373594
GALNT2	NIH	0.405133333	0.130267307	3.110015407	0.00427124	0.054795507
SPART	UCL	-0.765341551	0.254414344	-3.008248429	0.004276586	0.046373594
RANBP1	UCL	-0.597753051	0.197175397	-3.031580308	0.004284174	0.046373594
PGF	NIH	0.273913333	0.088128657	3.108107428	0.004291709	0.054817686
SUSD4	UCL	0.388693857	0.127760821	3.042355648	0.004296163	0.046373594
ARF6	UCL	-1.153789194	0.381403244	-3.025116365	0.004315536	0.046373594
CDNF	UCL	-0.332941935	0.108293611	-3.074437471	0.004319175	0.046373594
LATS1	UCL	-1.020421849	0.337604355	-3.022537574	0.004331164	0.046373594
ANXA4	UCL	-0.523305048	0.174388738	-3.000796121	0.004367422	0.046398951
TMED8	UCL	-1.210913471	0.402170893	-3.010942595	0.004375284	0.046398951
RBPMS	UCL	-0.623179036	0.202322916	-3.080120864	0.004407644	0.046398951
STK11	UCL	-0.75906202	0.24783488	-3.062773167	0.004418325	0.046398951
CASP7	UCL	-0.896477997	0.2981765	-3.00653471	0.004436738	0.046398951
GOPC	UCL	-1.26730643	0.420634164	-3.012847118	0.004446591	0.046398951
CLTA	NIH	1.341653333	0.433908352	3.092020074	0.004468069	0.056203209
PROC	NIH	0.468206667	0.151426076	3.091981771	0.004468497	0.056203209
HSD11B1	NIH	-0.46392	0.150043979	-3.091893483	0.004469484	0.056203209
CD63	UCL	-0.596157628	0.195327122	-3.052098559	0.004499212	0.046398951
UROD	UCL	-0.699411495	0.232816408	-3.004133181	0.004503068	0.046398951
PRSS8	UCL	-0.474734798	0.152994792	-3.102947436	0.004510258	0.046398951
VTI1A	UCL	-0.496535716	0.165805384	-2.994689938	0.004519749	0.046398951
NCK2	UCL	-0.939486319	0.313185925	-2.999771838	0.004529137	0.046398951
CLEC4A	NIH	-0.4697	0.152206705	-3.085935014	0.004536564	0.056203209
STAT5B	UCL	-1.715398917	0.573078752	-2.993303995	0.004536568	0.046398951
ERBB4	NIH	-0.310933333	0.100762079	-3.085816959	0.004537903	0.056203209
ARHGAP45	UCL	-0.786235969	0.264172206	-2.976225177	0.00453989	0.046398951

AMBP	NIH	0.243126667	0.078801966	3.085286831	0.004543919	0.056203209
RWDD1	UCL	-0.706215511	0.235589931	-2.997647263	0.004576772	0.046612906
IL31RA	NIH	0.510886667	0.165919289	3.079127633	0.004614377	0.056203209
PRKAB1	UCL	-0.439216874	0.146021354	-3.007894819	0.004630573	0.046997101
QDPR	NIH	0.483213333	0.157003481	3.077723699	0.004630582	0.056203209
BTN3A2	NIH	0.334106667	0.108567649	3.077405379	0.004634264	0.056203209
SIGLEC9	NIH	0.42788	0.139047272	3.077226855	0.00463633	0.056203209
MAG	NIH	-0.397026667	0.129033498	-3.076927088	0.004639802	0.056203209
UXS1	NIH	0.493573333	0.160447312	3.076233101	0.004647847	0.056203209
CFD	NIH	0.26266	0.085402051	3.07557017	0.004655546	0.056203209
REEP4	NIH	0.480266667	0.156214875	3.074397797	0.00466919	0.056203209
RICTOR	UCL	-0.367684815	0.122035392	-3.012935911	0.004682658	0.047326304
RPE	NIH	0.5126	0.166886158	3.071554923	0.004702433	0.056371382
OTUD7B	UCL	-0.639637353	0.213014105	-3.002793418	0.004709891	0.047326304
PLA2G4A	UCL	-0.936370709	0.31399972	-2.982074985	0.004711582	0.047326304
VSTM2L	NIH	1.022553333	0.333181177	3.069060935	0.004731783	0.056491693
NUDC	UCL	-0.686785285	0.229941401	-2.98678394	0.004734907	0.047370376
IKBKG	UCL	-0.920227003	0.309342636	-2.974782311	0.004748382	0.047370376
DDT	NIH	0.652053333	0.212607828	3.066930037	0.004756998	0.056513298
ERBIN	UCL	-1.212758891	0.405494589	-2.990813991	0.00477484	0.047472299
DUT	UCL	-1.198075179	0.401137329	-2.986695811	0.004796291	0.047523927
INHBC	NIH	0.598273333	0.195369686	3.062262852	0.004812673	0.056513298
CCL14	NIH	0.487453333	0.159195819	3.061973216	0.004816149	0.056513298
INPP5D	UCL	-0.559733007	0.188061498	-2.976329615	0.004823251	0.0476296
KHK	NIH	0.98774	0.322645298	3.061380422	0.004823269	0.056513298
OXCT1	NIH	0.61682	0.201522179	3.060804544	0.004830196	0.056513298
FKBP4	NIH	0.369773333	0.120885786	3.058865285	0.004853593	0.056528449
RAB11FIP3	UCL	-1.256750901	0.424673762	-2.959332583	0.004865266	0.047882736
SIGLEC7	NIH	0.318053333	0.104024023	3.057498884	0.004870143	0.056528449
SPON2	NIH	0.37254	0.12199595	3.05370792	0.004916342	0.056836998
MOCS2	NIH	0.47594	0.155936562	3.052138601	0.004935589	0.056836998
CDC37	UCL	-0.688931396	0.231465815	-2.976385064	0.00494172	0.048471969
ITIH4	NIH	0.35312	0.115896827	3.046847875	0.005001006	0.057364486

VTA1	UCL	-0.902972565	0.304185581	-2.968492334	0.005017032	0.049046106
GORASP2	UCL	-0.440744325	0.147195259	-2.994283432	0.005065766	0.04935745
CLSTN2	NIH	0.503733333	0.165654459	3.040867934	0.00507594	0.057996581
DCTD	UCL	-1.055460165	0.35859618	-2.943311235	0.005122239	0.049741872
ITGB1BP2	NIH	0.868813333	0.286234214	3.035323139	0.005146375	0.058303488
RNASE10	NIH	0.7629	0.25151173	3.033258131	0.005172844	0.058303488
ENPP5	NIH	-0.505733333	0.166733292	-3.033187484	0.005173752	0.058303488
PRCP	NIH	0.370253333	0.122128721	3.031664704	0.005193357	0.058303488
IL12A_IL12B	NIH	0.831793333	0.274432386	3.030959079	0.005202465	0.058303488
ITGB1BP2	UCL	-1.129874224	0.382973452	-2.950267745	0.005238192	0.050685104
GLRX5	UCL	-0.768618035	0.260526849	-2.950245001	0.005254049	0.050685104
ZFYVE19	NIH	0.73136	0.241989749	3.022276784	0.005315791	0.059346137
CACYBP	UCL	-1.103197367	0.376942819	-2.926696865	0.005343201	0.051375585
IGHMBP2	UCL	-0.579457351	0.199998581	-2.897307307	0.005362103	0.051388283
Triglycerides	NIH	0.919275619	0.303844113	3.025484384	0.005397227	0.060186304
PBXIP1	UCL	-0.328164001	0.112488243	-2.917318222	0.005419579	0.051545779
DNAJB14	UCL	-0.597671573	0.203776987	-2.932968943	0.005424722	0.051545779
STAT5B	NIH	0.927953333	0.307899587	3.01381805	0.005428454	0.060373494
MITD1	UCL	-1.224360907	0.417658765	-2.9314862	0.00543144	0.051545779
PADI2	NIH	1.266913333	0.421218628	3.007733393	0.005510898	0.061058241
AK2	UCL	-1.099803535	0.375628806	-2.927899875	0.005518752	0.052092621
AKR1B1	UCL	-1.122000462	0.371876997	-3.017127898	0.005524705	0.052092621
MCFD2	UCL	-0.468729762	0.158386525	-2.959404296	0.005549684	0.052159893
GMPR2	NIH	0.616993333	0.205431451	3.003402498	0.005570302	0.061483525
RNASE6	NIH	0.24948	0.083175949	2.999424742	0.005625399	0.061858241
CCN5	NIH	0.51748	0.172879124	2.993305314	0.005711173	0.062439032
YWHAQ	UCL	-0.74015065	0.254441507	-2.908922604	0.005712509	0.053518157
TMPRSS15	NIH	0.63648	0.212683472	2.992616182	0.00572091	0.062439032
SIRT2	NIH	0.725126667	0.2427801	2.98676319	0.005804245	0.063113075
ARHGAP1	UCL	-0.541338089	0.187451891	-2.887877455	0.005806222	0.053924054
SNX18	UCL	-0.412507625	0.136398442	-3.024284007	0.005808818	0.053924054
METAP2	UCL	-0.745401725	0.255255678	-2.920216039	0.005811179	0.053924054
CCL18	NIH	0.805426667	0.270004934	2.983007222	0.005858329	0.063465231

KIFBP	UCL	-1.210638547	0.417535374	-2.899487379	0.005868451	0.053969561
DCTN2	UCL	-0.828134496	0.280667922	-2.95058477	0.005870345	0.053969561
CRYZL1	UCL	-0.995642178	0.344279093	-2.891962361	0.005871475	0.053969561
PDZD2	UCL	-0.49082343	0.168357364	-2.915366567	0.005898745	0.05405026
ANXA2	UCL	-0.45322168	0.15597756	-2.905685155	0.005932885	0.054193196
PRDX5	NIH	0.575626667	0.193343493	2.977222863	0.005942558	0.064019035
SOD2	NIH	-0.37338	0.125442804	-2.976495977	0.005953223	0.064019035
CLPP	UCL	-1.245450005	0.425663784	-2.925900801	0.005953414	0.054211304
BCL2L1	UCL	-1.246255968	0.430023971	-2.898108133	0.005975913	0.054247181
ACAA1	NIH	1.111313333	0.373864607	2.972502111	0.006012149	0.064415885
HEXIM1	UCL	-0.944469801	0.324030482	-2.914756033	0.006032269	0.054583982
DNAJC6	UCL	-1.014898707	0.349459159	-2.904198337	0.006050363	0.054583982
TALDO1	NIH	0.338306667	0.113974565	2.968264608	0.006075275	0.064854672
PRDX5	UCL	-0.920381298	0.31887796	-2.88631205	0.00615014	0.055237275
PTGES2	UCL	0.592231388	0.203018469	2.917130597	0.006160572	0.055237275
PDCD5	NIH	0.71952	0.24302979	2.960624702	0.006190679	0.065846314
TARS1	NIH	0.57752	0.195214663	2.95838433	0.006224913	0.065970547
SIAE	UCL	-0.378565859	0.128539352	-2.945135895	0.006251079	0.055877383
TNFRSF1B	NIH	0.338053333	0.114395665	2.955123625	0.006275059	0.06626191
MTUS1	NIH	0.54202	0.183616226	2.951917774	0.006324736	0.066546228
F11R	UCL	-0.400712745	0.137469955	-2.91491144	0.006347157	0.056542598
ATP6V1G1	UCL	-0.507055206	0.175445032	-2.890108652	0.006364186	0.056542598
PLXDC1	NIH	-0.429726667	0.145753101	-2.948319206	0.006380941	0.066896962
GGACT	UCL	-0.749771003	0.260335008	-2.880023734	0.006410674	0.056783033
ULBP2	NIH	0.490913333	0.166740771	2.944170951	0.006446319	0.067341016
PPL	NIH	0.30576	0.103977459	2.94063736	0.006502511	0.067686278
GRAP2	UCL	-1.329113728	0.464999221	-2.858313881	0.006513595	0.057474705
CD40LG	UCL	-0.828343417	0.289076412	-2.865482563	0.006528088	0.057474705
ERP44	UCL	-0.28631105	0.097958598	-2.922776103	0.006565039	0.057626458
CCL19	NIH	0.821633333	0.279966332	2.93475765	0.00659704	0.068367405
PEBP1	NIH	0.588706667	0.200678769	2.933577225	0.006616174	0.068367405
RNF41	UCL	-0.645264494	0.225653177	-2.85954092	0.006619464	0.057930221
FSTL3	NIH	0.422813333	0.144195114	2.932230657	0.006638066	0.068367405

DCTN1	NIH	0.410853333	0.140386925	2.92657834	0.006730708	0.069078318
ZNRF4	UCL	-0.499682217	0.172299465	-2.900079903	0.006754132	0.058753609
IL32	UCL	0.511284225	0.174189116	2.935224869	0.006762139	0.058753609
VSIG4	NIH	0.4536	0.155096555	2.924629763	0.006762928	0.06916631
DTD1	UCL	-0.972317623	0.342167806	-2.841639708	0.006773851	0.058753609
CLSTN3	NIH	0.56574	0.193536492	2.923169656	0.006787167	0.069172346
LRRFIP1	UCL	-0.338776795	0.120049482	-2.821976321	0.006819471	0.0589743
ATOX1	NIH	0.548026667	0.18762269	2.920897609	0.006825048	0.069316896
DOK1	UCL	-1.675031038	0.591372801	-2.832445179	0.006848808	0.059053289
FXN	NIH	0.44298	0.151765942	2.918836698	0.006859582	0.069426569
STAMBP	UCL	-0.712537273	0.250379469	-2.845829478	0.00687709	0.059122746
TBCC	UCL	-0.971675021	0.341890803	-2.842062477	0.006913408	0.05926068
MVK	NIH	0.799593333	0.274275692	2.915290552	0.006919392	0.069657687
KRT6C	NIH	0.474486667	0.162830108	2.913998362	0.006941308	0.069657687
GCC1	NIH	0.53586	0.183938271	2.913259961	0.006953861	0.069657687
CD5L	NIH	0.743806667	0.255573843	2.910339558	0.007003721	0.069917693
ZNRD2	UCL	-0.958160938	0.328601997	-2.915870707	0.007068072	0.060409282
FAM3D	NIH	0.985933333	0.339413427	2.904815353	0.007098961	0.070627421
TRIM58	NIH	0.939526667	0.32363662	2.903029537	0.007130011	0.070695871
MPHOSPH8	UCL	-0.383037695	0.134887282	-2.839687241	0.00714749	0.060909952
PHACTR2	UCL	-1.441455711	0.509827292	-2.827341207	0.007169121	0.060916684
ADAM12	NIH	0.501326667	0.173221669	2.894133674	0.007286604	0.072004448
EBAG9	UCL	-1.022138375	0.360832006	-2.832726468	0.007381383	0.062538496
HYAL1	NIH	0.248786667	0.086188178	2.886552098	0.007422623	0.073101586
CRKL	UCL	-1.189412377	0.423687463	-2.807287163	0.007463439	0.063050962
SUGT1	UCL	-0.962078149	0.342323716	-2.810433823	0.007544931	0.063555715
MASP1	NIH	0.33628	0.116792653	2.879290708	0.007555143	0.073942043
SFRP1	NIH	0.535866667	0.186122508	2.879107271	0.00755852	0.073942043
NDRG1	NIH	0.733646667	0.255097152	2.875950043	0.007616859	0.074264371
CALCB	NIH	0.6391	0.222369054	2.874050994	0.007652153	0.074360626
NGFR	NIH	0.285646667	0.099475453	2.871529174	0.007699261	0.074570661
TARS1	UCL	-0.50103724	0.176963601	-2.831301103	0.007740269	0.064965884
PCBP2	UCL	-0.810751048	0.289792742	-2.797692733	0.007757048	0.064965884

FKBP14	UCL	-0.94521816	0.337053768	-2.804354234	0.007786533	0.064965884
PDE5A	UCL	-1.116220498	0.400637674	-2.786109675	0.00780124	0.064965884
CD177	UCL	-1.07355798	0.373893894	-2.871290485	0.00787149	0.065364673
PDIA4	UCL	-0.45172514	0.162686023	-2.776668409	0.007931509	0.065676491
LRRC38	NIH	-0.557146667	0.19511826	-2.855430688	0.008006497	0.077290445
CHMP1A	UCL	-0.86818449	0.312652914	-2.776831595	0.00801873	0.06621115
TIA1	UCL	-0.984982534	0.354610571	-2.77764572	0.008053796	0.066313368
IGBP1	UCL	-0.712311491	0.256012641	-2.78232938	0.008102497	0.066403126
GMPR2	UCL	-0.627452359	0.22655667	-2.769516164	0.008110132	0.066403126
SNCA	UCL	-1.461460585	0.5266427	-2.775051442	0.008146384	0.066513629
STK11	NIH	0.594546667	0.209061039	2.843890329	0.008233821	0.079207802
IMPACT	NIH	0.70024	0.246336396	2.842616886	0.008259275	0.079207802
CSTB	NIH	0.373973333	0.131633475	2.841019977	0.008291299	0.079255069
BRDT	NIH	0.47072	0.16590769	2.837240387	0.008367562	0.079511662
LAMP2	NIH	0.229933333	0.081048161	2.836996296	0.00837251	0.079511662
MGLL	UCL	-1.064521414	0.386241524	-2.756102977	0.008432516	0.068648185
MAPKAPK2	UCL	-0.75220863	0.271624065	-2.769300399	0.008454788	0.068648185
AHNAK	NIH	0.35806	0.126407305	2.832589452	0.008462314	0.079984106
ZBTB16	NIH	0.454546667	0.160510823	2.831875505	0.008476948	0.079984106
CASP8	NIH	0.479973333	0.169598203	2.830061436	0.008514239	0.080077652
DNMBP	UCL	-0.472947933	0.167816225	-2.818249143	0.008549158	0.06922213
YARS1	NIH	1.258446667	0.444945644	2.828315513	0.008550276	0.080158834
ATRN	NIH	0.171746667	0.060780253	2.825698471	0.008604561	0.080410037
CDKN2D	UCL	-1.283564437	0.466334009	-2.752457276	0.008619627	0.069290718
PDAP1	UCL	-0.902164593	0.327567155	-2.754136299	0.008629257	0.069290718
RAD23B	UCL	-0.513771028	0.185939673	-2.763106012	0.008646829	0.069290718
DDA1	UCL	-0.54498635	0.189541356	-2.875289923	0.00865245	0.069290718
CPM	NIH	0.53728	0.190436243	2.8213117	0.008696285	0.080885146
KLK13	NIH	-0.575833333	0.204150907	-2.820625885	0.008710708	0.080885146
NECTIN1	UCL	0.232905147	0.085007382	2.739822619	0.008775931	0.070087557
GPR15L	NIH	-0.628113333	0.222993397	-2.81673512	0.008792959	0.081317364
PCYT2	NIH	0.536806667	0.190640972	2.81579905	0.008812856	0.081317364
FHIT	UCL	-1.117479449	0.405269101	-2.757376383	0.008858719	0.070555957

ICA1	UCL	-0.637955534	0.23271045	-2.741413354	0.008960308	0.071171146
APRT	NIH	0.753766667	0.268477724	2.80755757	0.008989877	0.082419907
PARK7	NIH	0.54158	0.192976879	2.80645019	0.009013917	0.082419907
IL1RN	NIH	0.442093333	0.157598618	2.805185343	0.009041448	0.082419907
COMMD1	NIH	0.28888	0.102986794	2.805019835	0.009045056	0.082419907
CCL24	NIH	0.86956	0.31037216	2.801668812	0.009118407	0.082830253
ISLR2	NIH	-0.525166667	0.187777303	-2.796752633	0.009227031	0.08355748
ZP4	UCL	-0.20896425	0.076393322	-2.735373252	0.009233974	0.07314609
CA6	NIH	-0.734286667	0.262734322	-2.794787754	0.009270785	0.083575373
CEACAM16	NIH	0.752346667	0.269262643	2.794099693	0.009286153	0.083575373
SERPINA9	NIH	0.637533333	0.228311496	2.792383845	0.00932458	0.083663794
LILRB5	UCL	0.725087287	0.259025671	2.799287355	0.009351474	0.073765194
ENO2	UCL	-0.590425975	0.214968285	-2.746572481	0.009362602	0.073765194
GFRA1	NIH	0.400033333	0.143507433	2.787544344	0.009433771	0.084384648
VTA1	NIH	0.61312	0.220347661	2.782511947	0.009548588	0.084549846
AGRN	NIH	0.394973333	0.141964551	2.782196899	0.009555819	0.084549846
TXNDC9	UCL	-0.491713388	0.181585192	-2.707893642	0.009570009	0.075188949
BOLA1	NIH	0.404533333	0.145439828	2.781448102	0.009573027	0.084549846
DPY30	NIH	0.437666667	0.157358375	2.781336974	0.009575584	0.084549846
VPS4B	UCL	-1.038999491	0.383478437	-2.709407857	0.009594758	0.075188949
CDH17	NIH	-0.859866667	0.309258158	-2.780417089	0.009596769	0.084549846
GH	NIH	1.97878891	0.708733663	2.792006379	0.009690701	0.084014658
SNAP29	UCL	-0.978351187	0.362351432	-2.700006403	0.009787097	0.076491136
GUSB	NIH	0.657913333	0.237618163	2.768783859	0.009868512	0.086682873
GNAS	NIH	0.42754	0.15449849	2.767276231	0.009904253	0.086736234
ERC2	NIH	-0.656526667	0.237369164	-2.765846476	0.00993826	0.086736234
COMT	UCL	-0.925294709	0.340316418	-2.718924683	0.009938418	0.077192536
PMM2	UCL	-0.835848159	0.31059159	-2.691148721	0.009941457	0.077192536
TOP2B	UCL	-0.790417503	0.290242406	-2.723301232	0.009956559	0.077192536
MGMT	NIH	0.920793333	0.333043218	2.76478632	0.009963547	0.086736234
TAB2	UCL	-1.108026612	0.408636664	-2.711520308	0.010003235	0.077192536
HS1BP3	UCL	-0.813042335	0.299594602	-2.713808361	0.010019331	0.077192536
KITLG	NIH	-0.424613333	0.153748462	-2.761740357	0.010036536	0.087004266

IRAK4	UCL	-1.168734781	0.434691175	-2.688655414	0.010042873	0.077192536
HS6ST1	NIH	0.393246667	0.142427945	2.761021839	0.010053826	0.087004266
CRADD	UCL	-0.86253967	0.319597053	-2.698834871	0.010061702	0.077192536
DNAJC9	NIH	0.95056	0.344924878	2.755846445	0.010179195	0.087829341
FAM3D	UCL	-0.827999417	0.299895882	-2.760956278	0.010203731	0.078077237
FADD	UCL	-0.65593697	0.244425701	-2.683584281	0.010274382	0.078412581
SERPINB1	UCL	-0.753194791	0.279863455	-2.691293833	0.010315298	0.078422168
ERP29	UCL	-0.97809	0.365667201	-2.674809216	0.010329297	0.078422168
LAMP1	NIH	0.242706667	0.088319273	2.748060072	0.010370579	0.089217479
IZUMO1	NIH	0.665353333	0.242500865	2.743715299	0.010478831	0.089648417
EFNA1	NIH	0.356893333	0.1301022	2.743176771	0.010492323	0.089648417
DDR1	UCL	0.186412429	0.067966424	2.742713521	0.010495607	0.079478393
GLB1	NIH	0.506873333	0.184830539	2.742367881	0.010512618	0.089648417
VAMP8	UCL	-0.951344514	0.355332663	-2.677334824	0.01053073	0.079538304
DLL1	NIH	0.298546667	0.108993369	2.739126873	0.010594304	0.090082383
PRTFDC1	UCL	-1.612688264	0.603671366	-2.671467215	0.010644293	0.080170108
CHP1	UCL	0.55038974	0.208397266	2.641060274	0.010691154	0.080170108
ATOX1	UCL	-0.800435316	0.297564348	-2.689957047	0.010725879	0.080170108
ELAVL4	UCL	0.784631464	0.293794249	2.670683539	0.010748336	0.080170108
C1QTNF9	UCL	0.464730194	0.169212665	2.74642678	0.010751516	0.080170108
PIK3IP1	NIH	0.293906667	0.107605407	2.731337344	0.010793071	0.091471714
CD164	NIH	0.313113333	0.114681644	2.730282911	0.010820244	0.091471714
BRK1	UCL	-0.430385823	0.161161781	-2.670520397	0.010887357	0.080934766
MARS1	UCL	-0.766760303	0.288370274	-2.658943632	0.010909442	0.080934766
PTPRH	NIH	0.813806667	0.29848226	2.726482527	0.010918713	0.092038143
TCTN3	UCL	-0.352257727	0.128998523	-2.73071131	0.010989885	0.081325153
SLC28A1	NIH	0.480193333	0.176371428	2.722625422	0.01101951	0.092620878
CSF1	NIH	0.240913333	0.088621111	2.718464363	0.011129226	0.093275028
DTYMK	NIH	0.782653333	0.288243996	2.715245916	0.011214788	0.093348808
SAT1	NIH	0.70026	0.258006131	2.714121551	0.011244824	0.093348808
LDLRAP1	NIH	0.711413333	0.26217559	2.713499503	0.011261473	0.093348808
FURIN	NIH	0.49638	0.182940429	2.713342271	0.011265685	0.093348808
CA13	UCL	-1.135076351	0.428911943	-2.646408826	0.011335325	0.083452263

DNAJB6	UCL	-0.685745469	0.259813449	-2.639376339	0.011356922	0.083452263
DCXR	NIH	0.77028	0.284298032	2.709410242	0.0113715	0.093959432
ATXN3	NIH	0.686253333	0.253472232	2.707410308	0.011425677	0.09414114
OXT	UCL	-1.534258608	0.564471584	-2.718044011	0.01142964	0.083452263
LACRT	UCL	0.319063593	0.119030379	2.680522364	0.011431033	0.083452263
BANK1	UCL	-1.057177861	0.400271199	-2.641153956	0.011439106	0.083452263
CDH15	UCL	-0.750053783	0.276250746	-2.715119487	0.011448634	0.083452263
THBS2	NIH	0.424986667	0.157104924	2.705113605	0.01148819	0.094390327
IMPACT	UCL	-0.604992608	0.22922727	-2.639269785	0.011521071	0.083771368
ARFIP1	UCL	-0.763990728	0.289063644	-2.642984485	0.011612236	0.084224728
TMOD4	UCL	0.533871862	0.200262328	2.665862653	0.011690431	0.084582003
PRDX3	NIH	0.452126667	0.167626987	2.697218833	0.011705524	0.095906601
PHLDB2	NIH	1.273853333	0.472760335	2.694501292	0.011781219	0.096257167
ANKRD54	UCL	-0.494147028	0.187919417	-2.629568754	0.011781301	0.085028996
AIFM1	UCL	-1.112518401	0.418870357	-2.655996977	0.011828177	0.085157049
CELSR2	NIH	-0.341286667	0.126742768	-2.692750623	0.011830225	0.096388322
XIAP	NIH	0.352186667	0.130876023	2.690994557	0.011879573	0.096521531
ATG4A	UCL	-0.619190653	0.233960925	-2.64655584	0.011913807	0.085380557
EIF2AK2	UCL	-1.056826884	0.401823207	-2.630079264	0.011917642	0.085380557
HJV	NIH	0.422653333	0.157206617	2.688521264	0.011949403	0.096745455
POLR2F	NIH	0.33912	0.126175805	2.687678523	0.011973284	0.096745455
PLCB2	UCL	-1.117600549	0.425957178	-2.623739207	0.012016451	0.085828859
NT5C3A	UCL	-1.067182044	0.405873418	-2.629346974	0.012038944	0.085828859
SNX15	UCL	-0.522184287	0.196707227	-2.65462685	0.012157628	0.086464107
DTYMK	UCL	-0.714814483	0.272505425	-2.623120197	0.012197424	0.086536577
SEPTIN8	NIH	0.264213333	0.098682974	2.677395334	0.012268294	0.09855128
ALMS1	UCL	-0.529463462	0.19712183	-2.685970705	0.012275439	0.086868402
DSG4	UCL	0.372756649	0.138740217	2.686723853	0.012303633	0.086868402
VPS37A	NIH	0.52222	0.195137332	2.676166552	0.012303996	0.09855128
OPLAH	NIH	0.309093333	0.11555	2.674974756	0.012338717	0.09855128
SATB1	NIH	0.290213333	0.108528395	2.674077435	0.012364919	0.09855128
PRKD2	NIH	0.37112	0.138803705	2.673703853	0.012375843	0.09855128
S100A13	UCL	0.446081499	0.166834138	2.673802275	0.012396149	0.087310709

SUGT1	NIH	0.938626667	0.351178791	2.672788594	0.012402644	0.09855128
DMP1	NIH	-0.650693333	0.243544192	-2.67176699	0.012432623	0.09855128
MAP4K5	UCL	-1.295279833	0.492958488	-2.627563709	0.012490676	0.087765012
NRGN	UCL	-0.814271441	0.313019173	-2.601346853	0.012538784	0.087891766
UBXN1	UCL	-0.561620949	0.215755725	-2.603040775	0.012580757	0.087961923
PSMD9	NIH	0.78592	0.294710832	2.666749624	0.012580843	0.099456663
NUCB2	UCL	-0.30253725	0.113869568	-2.656875367	0.012608979	0.087961923
SERPINB9	UCL	-0.558378037	0.214363415	-2.60481965	0.012643349	0.087991688
HDGF	NIH	0.92976	0.348976641	2.664247087	0.012655386	0.099480607
ABHD14B	UCL	-0.520470063	0.198994775	-2.61549612	0.012710026	0.088245619
CCL23	NIH	0.374333333	0.140616056	2.662095238	0.012719812	0.099480607
ITIH1	NIH	0.151666667	0.056976692	2.661907198	0.012725456	0.099480607
ANGPTL3	NIH	0.417646667	0.156897571	2.661906517	0.012725477	0.099480607
TBCA	UCL	-0.778441346	0.29950125	-2.599125529	0.012749005	0.088306498
GLOD4	NIH	0.40556	0.15241116	2.660960006	0.012753924	0.099480607
DNAJB1	UCL	-0.940634729	0.357790945	-2.629006526	0.012841203	0.08842605
LARP1	UCL	-0.563008708	0.216676411	-2.598384863	0.012853882	0.08842605
STK4	UCL	-0.648823263	0.250628514	-2.588784697	0.012857021	0.08842605
SEL1L	NIH	0.27174	0.102297249	2.656376419	0.012892523	0.100223195
VASN	UCL	0.21569417	0.081465077	2.647688771	0.012913583	0.088564248
IL17C	NIH	0.689893333	0.259826964	2.655202998	0.01292823	0.100223195
DIABLO	UCL	-0.966234495	0.372410956	-2.594538318	0.012937713	0.088564248
YJU2	NIH	0.317	0.119423176	2.654426132	0.012951921	0.100223195
PLTP	UCL	0.305022139	0.114783076	2.657379021	0.012992565	0.088731933
EIF5	NIH	0.842906667	0.317800564	2.652313311	0.013016556	0.10045759
SNX15	NIH	0.586033333	0.221205317	2.64927327	0.013110085	0.100913153
TTR	UCL	-0.222368775	0.084822102	-2.62159	0.013125486	0.089430761
LPCAT2	NIH	0.659446667	0.249144821	2.646840757	0.013185373	0.101226291
RNF5	UCL	-0.629224408	0.244491794	-2.573601335	0.013307816	0.090462198
PKN3	UCL	-0.572295547	0.21982118	-2.603459528	0.013359124	0.090600279
ACY1	NIH	0.562026667	0.21284514	2.64054263	0.013382181	0.102391363
PALM	NIH	0.358486667	0.135803264	2.639750011	0.013407143	0.102391363
LTBR	NIH	0.272513333	0.103392273	2.635722443	0.01353465	0.103095968

DXO	UCL	-0.53264328	0.205984337	-2.585843607	0.013588787	0.091944499
CETN2	UCL	-1.107813523	0.43295402	-2.558732503	0.01366347	0.092236311
TPD52L2	NIH	0.498033333	0.189460115	2.628697518	0.013759756	0.104468791
ANXA3	UCL	-0.757172215	0.295582255	-2.561629467	0.013775735	0.092779892
MRC1	NIH	0.327973333	0.124805486	2.627875925	0.013786309	0.104468791
INHBB	NIH	0.68516	0.260861151	2.626531388	0.013829866	0.104528059
MXRA8	UCL	0.31590594	0.120527543	2.621026955	0.013948758	0.093729242
PROCR	NIH	0.348173333	0.13283518	2.621092791	0.014007364	0.105559368
ADGRG1	NIH	1.223466667	0.466946065	2.620145574	0.014038494	0.105559368
AMN	NIH	0.701913333	0.268037909	2.618709178	0.014085823	0.105643672
SPINT2	NIH	0.31198	0.119209736	2.617068122	0.014140077	0.10577935
CXCL14	NIH	0.455833333	0.174286668	2.615422853	0.014194666	0.10591683
EHBP1	NIH	0.4399	0.168370161	2.612695722	0.014285581	0.106085143
EPHB6	NIH	0.31028	0.118766772	2.612515223	0.014291618	0.106085143
PRAP1	NIH	0.554966667	0.21250978	2.611487657	0.014326028	0.106085143
SERPINA11	NIH	0.396486667	0.151900863	2.610167314	0.014370355	0.10614467
TCN2	UCL	0.281642876	0.10729488	2.624942378	0.014381417	0.096414865
AKT2	UCL	-1.354477585	0.532720482	-2.542567125	0.014481127	0.096861178
TBR1	NIH	0.4944	0.189948354	2.602812759	0.014619608	0.107713737
PRG2	NIH	0.547273333	0.210362704	2.601570154	0.014662116	0.107755498
PMVK	UCL	-1.573990577	0.619533134	-2.540607583	0.014705811	0.098139462
CYB5R2	UCL	-0.413626902	0.161864391	-2.555391576	0.014902088	0.0990318
MANF	UCL	-1.434159797	0.564565495	-2.540289497	0.014907284	0.0990318
EIF4EBP1	NIH	0.95436	0.367949492	2.593725557	0.014933128	0.109472177
NFATC1	UCL	-0.670927664	0.26545724	-2.527441573	0.014969764	0.099105519
SYTL4	UCL	-0.50533243	0.199432885	-2.533847064	0.015011529	0.099105519
SIRT2	UCL	-0.945592729	0.371570806	-2.544852054	0.015020098	0.099105519
PIK3AP1	UCL	-0.538184409	0.211829014	-2.540654839	0.01511369	0.099498457
ACP1	NIH	0.612433333	0.236677372	2.587629436	0.015146937	0.110014673
FABP9	NIH	0.530293333	0.204966199	2.587223339	0.015161281	0.110014673
GIPC2	NIH	0.511326667	0.197650317	2.587026796	0.015168227	0.110014673
FLI1	NIH	0.476233333	0.184219427	2.58514176	0.015235001	0.110014673
XCL1	NIH	0.529186667	0.204834388	2.583485479	0.015293897	0.110014673

FBLN2	NIH	0.426553333	0.165111623	2.583424027	0.015296086	0.110014673
C1S	NIH	0.265653333	0.102881193	2.582136995	0.015342003	0.110014673
SCARB2	NIH	0.301213333	0.11666157	2.581941368	0.015348994	0.110014673
MIF	NIH	0.602353333	0.233309365	2.581779485	0.015354781	0.110014673
RAB27B	UCL	-1.184412794	0.470257265	-2.518648581	0.015381712	0.101035383
RAPGEF2	NIH	0.7706	0.298568303	2.58098396	0.015383248	0.110014673
CASC3	UCL	-0.284913362	0.114032542	-2.498526797	0.015432427	0.101141223
CPB2	NIH	0.274206667	0.106343175	2.578507422	0.015472185	0.110380829
XIAP	UCL	-0.631585219	0.248269696	-2.543948089	0.0156053	0.102045397
ATXN2L	NIH	0.25792	0.100220675	2.573520877	0.015652707	0.111126447
MKI67	NIH	0.557586667	0.216717474	2.572873602	0.015676282	0.111126447
KLKB1	NIH	0.33628	0.130765072	2.571634718	0.015721497	0.111126447
PTRHD1	NIH	0.577606667	0.22462394	2.571438589	0.015728666	0.111126447
HS3ST3B1	NIH	0.344166667	0.134029972	2.567833613	0.01586098	0.111558228
GALNT10	NIH	0.301586667	0.117454205	2.567695774	0.015866059	0.111558228
SLC9A3R1	UCL	-0.526571072	0.209425489	-2.514359995	0.015869693	0.10339191
HSPB1	UCL	-0.792172356	0.315698627	-2.509267663	0.01588196	0.10339191
NXPH3	NIH	-0.362453333	0.141420622	-2.562945402	0.016042039	0.112410061
DAPP1	UCL	-1.423886939	0.568468653	-2.504776529	0.016071435	0.104392898
DHRS4L2	NIH	0.292053333	0.114023083	2.561352711	0.016101445	0.112410061
MTDH	UCL	-1.058675765	0.41681268	-2.539931761	0.01613548	0.104544471
GCHFR	NIH	0.407253333	0.159072167	2.560179704	0.016145327	0.112410061
UPK3BL1	UCL	0.22398627	0.090312118	2.480135287	0.016166302	0.104544471
ROBO1	NIH	0.262553333	0.102667803	2.557309369	0.016253173	0.112410061
PRR5	NIH	0.503813333	0.19703749	2.556941494	0.016267044	0.112410061
ZNRF4	NIH	0.355413333	0.139030617	2.556367383	0.016288712	0.112410061
APOC1	NIH	0.510373333	0.199676612	2.555999563	0.016302608	0.112410061
EIF2S2	NIH	0.655733333	0.256569707	2.555770673	0.016311261	0.112410061
STAMBP	NIH	0.519326667	0.203243549	2.555193856	0.016333086	0.112410061
PPME1	UCL	-0.713510758	0.28411522	-2.511342958	0.016356789	0.105542814
MYH9	UCL	-1.076206824	0.429733585	-2.504358192	0.016423949	0.10574274
ALDH1A1	NIH	0.707373333	0.277420489	2.549823686	0.016537577	0.113088823
RNF43	UCL	0.326830068	0.130376232	2.506822475	0.01654361	0.106279057

PRR4	NIH	-0.953853333	0.374158318	-2.549330822	0.016556464	0.113088823
IGDCC3	NIH	-0.334073333	0.131051072	-2.549184288	0.016562083	0.113088823
CST3	NIH	0.264786667	0.103954321	2.547144397	0.016640486	0.113088823
OSMR	NIH	0.20522	0.080578629	2.546829121	0.016652635	0.113088823
SUOX	NIH	0.675953333	0.265439645	2.546542486	0.016663686	0.113088823
ELN	NIH	0.592953333	0.23323127	2.542340629	0.016826479	0.113929285
GLOD4	UCL	-0.388100144	0.155493212	-2.495929819	0.016909657	0.108392382
MPRIP	NIH	0.48326	0.190340075	2.538929336	0.016959723	0.11456626
CRYZL1	NIH	0.553033333	0.218374221	2.532502836	0.017213395	0.115935597
SERPINB9	NIH	0.331293333	0.130853345	2.531791088	0.017241704	0.115935597
WASHC3	NIH	0.326226667	0.128947299	2.529922447	0.017316233	0.116169683
IL18	UCL	-0.347959092	0.137488396	-2.530825165	0.017343856	0.110932366
MARS1	NIH	0.433266667	0.171491922	2.526455244	0.017455308	0.116426452
COL2A1	NIH	0.946713333	0.374879524	2.525380216	0.017498639	0.116426452
OCLN	NIH	0.34764	0.137691138	2.524781232	0.017522825	0.116426452
LRRC37A2	NIH	0.581586667	0.230374944	2.524522231	0.017533292	0.116426452
GFOD2	NIH	0.88688	0.351375706	2.524021968	0.017553527	0.116426452
TBCB	UCL	-1.168154556	0.473272365	-2.468250084	0.017608814	0.112136694
NDUFA5	UCL	0.324467134	0.131177671	2.473493641	0.017608875	0.112136694
TXN	NIH	0.35642	0.14130368	2.522368846	0.017620545	0.116452129
FOXO3	NIH	0.9074	0.359799054	2.521963275	0.017637023	0.116452129
REN	NIH	0.71692	0.284632726	2.518754644	0.017767891	0.117051985
SLC12A2	UCL	-0.22455149	0.091936591	-2.44246049	0.01776877	0.112908943
MTHFSD	UCL	-1.197943154	0.483527539	-2.477507604	0.017956901	0.11385688
NBL1	NIH	0.22622	0.089983403	2.514019157	0.017962672	0.118069249
SCT	UCL	-0.524170621	0.211806799	-2.474758244	0.018062528	0.114278719
DENR	NIH	0.52916	0.210905922	2.508985968	0.018171858	0.11917642
DUOX2	NIH	1.270653333	0.506920802	2.506611149	0.018271336	0.119357508
TNFRSF11A	NIH	0.35372	0.14112789	2.50637915	0.018281082	0.119357508
IFT20	UCL	-0.304908045	0.123636019	-2.466174874	0.018313465	0.115616107
JPT2	NIH	0.38248	0.152720522	2.504444036	0.018362553	0.119622422
TIMM8A	UCL	-0.528449694	0.210959452	-2.504982299	0.0184168	0.115799004
PLPBP	UCL	-0.616089947	0.251072708	-2.453830816	0.018421668	0.115799004

MYCBP2	UCL	-0.517898682	0.211986487	-2.443074031	0.018478307	0.115905775
IGFBPL1	NIH	0.375793333	0.150263228	2.500900182	0.018512624	0.120332053
TGFB1	NIH	0.244533333	0.097855502	2.498922669	0.018596856	0.120611536
HDDC2	NIH	0.300333333	0.120448567	2.493457092	0.018831503	0.121649771
GPKOW	NIH	0.294433333	0.118091822	2.49325761	0.018840118	0.121649771
NDUFB7	UCL	-0.820153048	0.333468723	-2.459460185	0.018880858	0.118177192
NRTN	NIH	0.778726667	0.312685429	2.490447568	0.018961869	0.122166228
CKAP4	NIH	0.245146667	0.098548703	2.48756867	0.019087355	0.122704424
LRTM2	UCL	0.222487251	0.090879964	2.448144135	0.019090737	0.11923552
SETMAR	NIH	0.3301	0.132806102	2.485578571	0.019174546	0.122994622
OSM	UCL	-0.687845891	0.275781512	-2.494169698	0.019198588	0.119653457
HBQ1	NIH	0.97836	0.394031374	2.482949495	0.019290295	0.123466332
DNAJA1	NIH	-0.47282	0.190541952	-2.481448281	0.019356677	0.123574978
NTproBNP	UCL	-0.853696428	0.343034421	-2.488661127	0.019393449	0.120380219
TPBGL	UCL	0.319140981	0.130719842	2.441411913	0.019397565	0.120380219
DNM3	NIH	0.314066667	0.126612993	2.48052479	0.019397616	0.123574978
HRG	NIH	0.35168	0.141823306	2.479705275	0.019434014	0.123574978
SH3GLB2	UCL	-0.523621911	0.214459189	-2.441592324	0.019489524	0.120694659
SNED1	NIH	0.31286	0.126335895	2.476414162	0.019580816	0.124238367
MORF4L2	NIH	0.38858	0.156976883	2.475396329	0.019626424	0.124258201
OPLAH	UCL	-0.532435061	0.22005885	-2.41951215	0.019649774	0.121316279
MRI1	UCL	-0.516829834	0.212358139	-2.433765134	0.019695001	0.121316279
DDX25	NIH	0.80106	0.323831714	2.473692246	0.019703	0.124473594
SOD1	UCL	-0.377844593	0.155140943	-2.435492436	0.019714414	0.121316279
LGALS3	NIH	0.224346667	0.090883018	2.468521312	0.019937051	0.125680763
CRYBB1	UCL	-0.497355744	0.204508799	-2.431952783	0.019965198	0.122601415
NHLRC3	NIH	0.218593333	0.088588917	2.467502047	0.019983486	0.125702576
IRAK1	UCL	-0.5839129	0.23965325	-2.436490639	0.020140066	0.123415962
DUSP13	NIH	-0.542546667	0.220405011	-2.461589532	0.020254816	0.127135915
COL24A1	NIH	0.6779	0.275726389	2.458596733	0.020393445	0.127731961
LHPP	NIH	0.748266667	0.304569006	2.456805029	0.020476855	0.127733481
EIF4G1	NIH	0.596146667	0.242718859	2.456120097	0.020508824	0.127733481
SORT1	NIH	0.308706667	0.125736439	2.455188555	0.020552376	0.127733481

KRT18	NIH	0.854526667	0.34821254	2.454037604	0.020606304	0.127733481
BTN2A1	NIH	0.223273333	0.09102138	2.452976808	0.020656123	0.127733481
SV2A	UCL	-0.58267114	0.242838347	-2.399419808	0.020687717	0.126303196
TMOD4	NIH	1.553746667	0.633592241	2.452281713	0.020688828	0.127733481
SLA2	UCL	-1.007016476	0.419127035	-2.402652164	0.02069765	0.126303196
S100P	NIH	0.46688	0.190403344	2.452057771	0.020699374	0.127733481
METAP2	NIH	0.389153333	0.158920154	2.448734944	0.020856445	0.12789793
SHISA5	NIH	0.270826667	0.110628785	2.448066896	0.020888156	0.12789793
GADD45GIP1	UCL	-0.445258639	0.185098696	-2.405520127	0.020895731	0.127246296
PLPBP	NIH	0.44714	0.182669575	2.447807748	0.020900469	0.12789793
CD300LF	NIH	0.757146667	0.309438804	2.446838139	0.020946598	0.12789793
RNF149	NIH	0.242473333	0.099107444	2.446570335	0.020959355	0.12789793
TRAF2	NIH	0.451826667	0.18472353	2.445961626	0.020988378	0.12789793
CNPY4	UCL	-0.591890097	0.246169955	-2.404396166	0.020997179	0.12759824
C2orf69	NIH	0.58982	0.241281173	2.444533872	0.021056598	0.128046881
TPD52L2	UCL	-0.959848494	0.402213545	-2.386415144	0.021175999	0.127921085
SFRP4	UCL	0.466737218	0.190623726	2.4484739	0.021193078	0.127921085
APOB	UCL	0.18356353	0.07679566	2.390285193	0.021196988	0.127921085
PAMR1	NIH	0.337333333	0.138181015	2.441242261	0.021214652	0.12873622
GP6	UCL	-0.736212007	0.307774384	-2.392050951	0.02122536	0.127921085
C3	NIH	0.30886	0.126564133	2.440343824	0.021257981	0.12873622
NSFL1C	UCL	-0.588175779	0.244963909	-2.401071168	0.021289588	0.127922151
STK24	UCL	-0.757440221	0.317313747	-2.387038784	0.021313065	0.127922151
DBN1	NIH	0.341913333	0.140194378	2.438851959	0.021330109	0.128906133
SPINT1	NIH	-0.240253333	0.098577913	-2.43719233	0.021410612	0.128941698
CENPJ	UCL	-0.487467409	0.203782644	-2.392094832	0.021413585	0.12826211
PTH1R	NIH	0.266786667	0.109477277	2.436913606	0.021424159	0.128941698
IL12B	NIH	0.50752	0.208457298	2.434647314	0.021534603	0.129340276
EIF2S2	UCL	-0.24218842	0.101967821	-2.375145586	0.021559972	0.128642592
S100A4	UCL	-0.453376278	0.190002004	-2.386165761	0.021565128	0.128642592
ZBTB16	UCL	-0.798289874	0.334101541	-2.389363041	0.021678174	0.12905357
CD226	NIH	0.54372	0.223744877	2.430089159	0.021758321	0.130416168
PSME1	UCL	-0.345317077	0.143986241	-2.398264407	0.021788225	0.129445083

CLGN	NIH	0.541513333	0.223006227	2.42824311	0.021849532	0.130695053
UBXN1	NIH	0.639346667	0.263520123	2.426177778	0.021951992	0.131039954
DAPK2	UCL	-0.499796182	0.212254208	-2.354705649	0.022068233	0.130842689
COQ7	UCL	0.534807556	0.220563124	2.424736941	0.022194315	0.131175503
METAP1D	NIH	0.484366667	0.200045766	2.421279272	0.022196769	0.132231263
MED18	UCL	-0.713373984	0.300568977	-2.37341189	0.02221412	0.131175503
SPESP1	NIH	0.682166667	0.281950131	2.419458587	0.022288384	0.132507162
AMOT	UCL	0.332597998	0.138305339	2.404809537	0.022447001	0.132283436
CD302	NIH	0.369006667	0.152808927	2.414824021	0.022523156	0.133631302
IL1A	UCL	-0.735197274	0.300037882	-2.45034817	0.0226162	0.132983466
CNP	UCL	-0.734604179	0.310936789	-2.362551502	0.022656779	0.132983466
PPM1F	UCL	-0.385890969	0.163538774	-2.359629835	0.022777326	0.133423096
ATP6AP2	NIH	0.311906667	0.129441095	2.409641751	0.022788354	0.134931044
AGT	NIH	0.11536	0.047906518	2.408023037	0.022871775	0.135151395
NFKB1	UCL	-0.635212133	0.269385332	-2.358005648	0.022898497	0.133864615
IL1B	UCL	-0.303385574	0.126758022	-2.39342307	0.023016207	0.134284178
A1BG	NIH	0.135453333	0.056347637	2.403886657	0.023086214	0.135883975
CDC26	NIH	0.304813333	0.12680269	2.403839646	0.023088662	0.135883975
CCDC134	UCL	-0.462234394	0.198096225	-2.333383151	0.023244181	0.135344107
P4HB	UCL	-0.283086741	0.119177897	-2.37532921	0.023317898	0.135503412
MAN1A2	NIH	0.315006667	0.131298552	2.399163289	0.023333334	0.137048194
IGSF8	NIH	0.41396	0.172687967	2.397156022	0.023439082	0.137393414
HEBP1	UCL	0.396877762	0.166618276	2.381958158	0.023451263	0.136008018
INPP1	UCL	-0.405604727	0.172449397	-2.352021723	0.023566101	0.136378738
MAP4K5	NIH	0.62024	0.259072522	2.39407867	0.023602056	0.138000905
ACYP1	UCL	-0.51218443	0.218393533	-2.345236243	0.023624988	0.136378738
CPQ	NIH	0.31034	0.12967488	2.393216027	0.023647926	0.138000905
CCER2	UCL	-0.530330087	0.220734464	-2.402570387	0.023655156	0.136378738
CD34	NIH	-0.269086667	0.112469324	-2.392533858	0.023684258	0.138000905
KAZALD1	NIH	0.418133333	0.174848994	2.39139685	0.023744927	0.138079348
BECN1	NIH	0.602546667	0.252148888	2.389646335	0.02383861	0.138349077
FLI1	UCL	-0.735168327	0.310979822	-2.364038679	0.023949163	0.137801977
SNCA	NIH	1.456006667	0.609924321	2.387192339	0.02397051	0.138839095

COMMD1	UCL	-0.44120344	0.188045214	-2.34626253	0.024006625	0.137861226
AKR7L	NIH	0.822393333	0.344739948	2.385546954	0.024059322	0.139078095
CASP2	UCL	-0.543633134	0.229991422	-2.363710482	0.024090864	0.138073716
CSPG4	UCL	0.304122414	0.126914626	2.396275531	0.024187244	0.138354821
CSNK2A1	NIH	0.27826	0.116864935	2.381039278	0.02430417	0.140216366
APCS	UCL	-0.340085928	0.142891818	-2.380023797	0.024428697	0.139463051
CFH	NIH	0.251286667	0.105649608	2.378491242	0.02444358	0.140743052
CFP	NIH	0.33004	0.138847904	2.376989432	0.024526089	0.140940688
ARHGEF10	UCL	-0.870236547	0.370900601	-2.34627969	0.024567987	0.139984846
SHBG	UCL	0.464992496	0.195269549	2.38128525	0.024643045	0.14013934
CD36	NIH	0.722766667	0.304438721	2.374095728	0.024685786	0.141580246
INPPL1	NIH	0.43544	0.183486426	2.373145577	0.02473843	0.141604516
MFAP5	NIH	0.385633333	0.162724166	2.369859018	0.024921314	0.142224492
TMSB10	NIH	0.401353333	0.16944149	2.368683922	0.024987003	0.142224492
KIF1C	NIH	0.347926667	0.146892282	2.368583717	0.024992612	0.142224492
FGF7	UCL	0.545294846	0.233203769	2.338276299	0.025044992	0.141960558
PPP1R2	UCL	-0.62144955	0.267419916	-2.32387161	0.025103696	0.141960558
BSG	UCL	-0.159463979	0.067245419	-2.371373122	0.025109001	0.141960558
KIAA0319	NIH	-0.248726667	0.105126806	-2.365968081	0.025139424	0.142782168
PPIB	UCL	-0.76251371	0.3273328	-2.329475412	0.025182584	0.142101723
TNFRSF4	NIH	0.404353333	0.171119569	2.362987098	0.025307703	0.143201168
AKT1S1	UCL	-0.69016529	0.294657233	-2.342264886	0.025326728	0.142639744
CEACAM3	NIH	0.455246667	0.192758964	2.361740581	0.025378374	0.143201168
ADGRG2	NIH	-0.240706667	0.101974845	-2.360451416	0.025451652	0.143201168
IL2RB	NIH	0.608066667	0.25761369	2.360381806	0.025455614	0.143201168
VWA1	NIH	0.453186667	0.192034731	2.35992033	0.025481895	0.143201168
CTSO	NIH	0.27658	0.117231236	2.359268822	0.025519041	0.143201168
HEXIM1	NIH	0.40446	0.171481396	2.358623201	0.025555901	0.143201168
FOSB	NIH	0.722586667	0.306646279	2.356417528	0.025682191	0.143267391
RNF41	NIH	0.368046667	0.156206353	2.356156834	0.025697156	0.143267391
TST	UCL	-0.562195957	0.243294514	-2.310762986	0.025701576	0.144472511
RARRES1	NIH	0.31806	0.135034095	2.355405126	0.025740349	0.143267391
ISM1	NIH	0.404233333	0.171758728	2.353495153	0.025850395	0.143267391

CPPED1	NIH	0.43936	0.186685401	2.35347809	0.02585138	0.143267391
NUCB2	NIH	0.218393333	0.092802972	2.353301074	0.025861601	0.143267391
CXCL13	NIH	0.39724	0.168966701	2.350995775	0.025995047	0.143734429
ARL2BP	UCL	-0.506101318	0.219267915	-2.308141239	0.026127377	0.146584113
CCS	NIH	0.61628	0.262512116	2.347624972	0.026191302	0.144546336
LRIG3	NIH	0.26638	0.113651057	2.343840935	0.026413223	0.145353875
PSME1	NIH	0.373646667	0.159501183	2.342594956	0.026486669	0.145353875
SH3BP1	NIH	0.284813333	0.121580305	2.342594329	0.026486706	0.145353875
DXO	NIH	0.440593333	0.188385971	2.338779961	0.026712708	0.146319608
SLC27A4	UCL	-0.767186667	0.332824444	-2.305079093	0.026799415	0.149866458
HNRNPUL1	UCL	-0.367539527	0.159005183	-2.311494008	0.02681497	0.149866458
VEGFA	NIH	0.465306667	0.199150198	2.336460985	0.026850964	0.146701779
CEP290	UCL	-0.345468795	0.148079335	-2.332998012	0.026879568	0.149940794
RBKS	NIH	0.554826667	0.237518661	2.335928743	0.026882788	0.146701779
CRTAC1	NIH	-0.24762	0.106086399	-2.334135213	0.026990279	0.146877149
YES1	UCL	-0.889916061	0.388261098	-2.292055697	0.026995292	0.150299505
CA4	NIH	-0.279293333	0.119705548	-2.333169492	0.027048319	0.146877149
STX6	NIH	0.47122	0.201990424	2.332882867	0.027065567	0.146877149
CEP43	UCL	-0.506368115	0.219760216	-2.304184639	0.027119402	0.150577371
GTPBP2	UCL	-0.62952533	0.275853136	-2.282103219	0.027148229	0.150577371
DDX39A	NIH	0.233686667	0.100327214	2.329245061	0.027285348	0.146901268
EP300	NIH	0.37962	0.163049232	2.328253837	0.027345515	0.146901268
TMEM106A	UCL	-0.675631397	0.294852974	-2.291417946	0.027351329	0.151416543
LYZL2	NIH	0.34022	0.146158143	2.327752616	0.027375984	0.146901268
F7	NIH	0.335873333	0.144300494	2.327596551	0.027385478	0.146901268
MAPK9	NIH	0.604433333	0.259770947	2.326793433	0.02743438	0.146901268
CD300A	NIH	0.279233333	0.120009516	2.326759929	0.027436421	0.146901268
GPC5	NIH	0.539086667	0.231708633	2.326571346	0.027447916	0.146901268
SMOC1	NIH	0.308413333	0.132583613	2.326179875	0.027471793	0.146901268
ASPSCR1	UCL	-0.568742667	0.24818057	-2.291648644	0.027580996	0.152141962
CXCL3	UCL	-0.931755468	0.408531072	-2.280745655	0.027619512	0.152141962
CNTF	UCL	0.411471437	0.180619142	2.27811644	0.027638516	0.152141962
AIF1	NIH	0.504606667	0.217204525	2.323186711	0.027654973	0.147101339

PSME2	NIH	0.47026	0.202430283	2.323071394	0.027662053	0.147101339
PPP1R2	NIH	0.335866667	0.144584163	2.32298379	0.027667432	0.147101339
LRRFIP1	NIH	0.41446	0.178483324	2.322121701	0.027720419	0.147101339
SRP14	NIH	0.526773333	0.226913897	2.321467929	0.027760663	0.147101339
PARK7	UCL	-0.511166253	0.222840105	-2.2938701	0.02777004	0.152576844
PAFAH2	UCL	-0.466307742	0.20484339	-2.276410974	0.027840706	0.152576844
NT5C1A	UCL	0.285216111	0.122784158	2.322906441	0.027874114	0.152576844
STX16	UCL	-0.916914653	0.400587742	-2.288923392	0.027956986	0.152668856
LGALS8	UCL	-0.390778322	0.170447403	-2.292662225	0.028041599	0.152668856
OTUD6B	UCL	-0.582060278	0.254348211	-2.288438655	0.028047614	0.152668856
ELAC1	NIH	0.64694	0.279295463	2.316328357	0.028078894	0.148518564
GHRHR	NIH	0.9451	0.408335585	2.314517849	0.028191784	0.148846513
RBP7	NIH	0.626013333	0.270606964	2.313367417	0.02826373	0.148850957
FKBPL	NIH	0.420853333	0.181960907	2.312877748	0.028294404	0.148850957
TXNDC5	UCL	-0.374347702	0.163420646	-2.290700176	0.028491429	0.154586218
SPTLC1	UCL	-0.309713085	0.136666377	-2.266198111	0.028505635	0.154586218
NACC1	UCL	-0.402323534	0.176608834	-2.278048751	0.028589224	0.154634398
MGMT	UCL	-1.216623451	0.535800192	-2.270666321	0.028620325	0.154634398
EFNA4	NIH	0.28362	0.122907594	2.307587267	0.028627742	0.150334191
FST	UCL	0.430243398	0.186971225	2.301120931	0.028819529	0.155184737
ELOB	UCL	-0.275154632	0.121627873	-2.262266249	0.028828365	0.155184737
TOMM20	NIH	0.331293333	0.143778257	2.304196343	0.028843264	0.151158852
LECT2	NIH	1.242273333	0.539299753	2.303493238	0.028888136	0.151158852
SMAD1	NIH	0.388706667	0.168964873	2.300517615	0.029078743	0.151819449
COL1A1	NIH	-0.390386667	0.16974042	-2.299903971	0.029118192	0.151819449
CCS	UCL	-0.347617688	0.153201422	-2.2690239	0.029177553	0.156775714
COLEC12	NIH	0.25186	0.109578586	2.298441787	0.029212386	0.152039556
VSIR	UCL	-0.304626218	0.13361893	-2.279813323	0.029393257	0.157644936
PLAT	NIH	0.554813333	0.241778153	2.294720702	0.029453348	0.15299949
TRDMT1	UCL	-0.661081938	0.288561953	-2.290953234	0.02946689	0.157750401
APOF	NIH	0.329873333	0.143808907	2.293831025	0.029511226	0.15299949
NME1	NIH	0.410206667	0.178884963	2.293131075	0.029556834	0.15299949
SEPTIN8	UCL	-0.259481041	0.112910867	-2.298105108	0.02957753	0.158053238

SERPINF1	NIH	0.145833333	0.063616651	2.292376769	0.029606055	0.15299949
CHAD	NIH	-0.38292	0.167254086	-2.289450788	0.029797689	0.153718238
ZNF75D	UCL	0.515810803	0.22698839	2.272410512	0.029885746	0.15923485
TNFAIP2	UCL	-0.318950557	0.140656987	-2.267577055	0.029907606	0.15923485
BLOC1S3	UCL	-0.249545326	0.111668063	-2.234706322	0.029965116	0.159250973
PIBF1	NIH	0.633026667	0.277053778	2.284851236	0.030101205	0.154595559
PGD	UCL	-0.420734504	0.187662448	-2.241974927	0.030117924	0.159772579
F2	NIH	0.210633333	0.09220137	2.284492438	0.030124998	0.154595559
ITPA	NIH	0.54478	0.238470796	2.284472605	0.030126314	0.154595559
COPE	NIH	1.064426667	0.466333665	2.28254305	0.03025457	0.154877233
LCAT	NIH	0.29124	0.127621827	2.282054776	0.030287103	0.154877233
NEDD4L	NIH	0.576433333	0.252736484	2.280768188	0.03037298	0.155007869
POSTN	UCL	0.300306586	0.132950579	2.258783597	0.030409243	0.161025754
NCAM1	NIH	-0.365066667	0.160110956	-2.280085483	0.030418638	0.155007869
BOLA2_BOLA2B	UCL	-0.500841049	0.223508581	-2.240813516	0.030544923	0.161451734
TPR	NIH	0.477513333	0.209823975	2.275780611	0.030707976	0.156210138
HSPG2	NIH	0.256546667	0.112958856	2.271151431	0.031021887	0.157533022
MICALL2	UCL	-0.640548916	0.283160247	-2.262142807	0.031153429	0.164370889
SCN4B	UCL	-0.333558134	0.147235609	-2.265471886	0.031218031	0.164414965
RP2	NIH	0.196713333	0.086823093	2.26567987	0.031396663	0.15890486
SERPINE1	NIH	0.302473333	0.133533347	2.265152048	0.031433032	0.15890486
SLAMF8	NIH	0.495513333	0.218785786	2.264833303	0.031455013	0.15890486
GATA3	UCL	-0.287400921	0.130376732	-2.204388137	0.031622998	0.166248243
PRKAR2A	NIH	0.417913333	0.184734929	2.262232349	0.031634899	0.15953807
TNXB	NIH	-0.211466667	0.093520656	-2.261176032	0.031708221	0.15963261
BRDT	UCL	-0.309503763	0.139395855	-2.220322577	0.031858575	0.167186021
MEP1B	UCL	1.263579056	0.558813704	2.261181224	0.032061863	0.167673069
EGFL7	UCL	0.38997308	0.172360103	2.26254843	0.032074896	0.167673069
PDGFA	NIH	0.334493333	0.148293529	2.255616519	0.032096657	0.161310521
ABO	UCL	-1.399872004	0.619400226	-2.260044386	0.032123475	0.167673069
SOD1	NIH	0.45808	0.203533037	2.250641989	0.03244786	0.16255756
C1QL2	NIH	0.273386667	0.12147667	2.250528158	0.032455937	0.16255756
UBE2Z	NIH	0.384526667	0.171045358	2.248097645	0.032628829	0.163008889

ASPSCR1	NIH	0.50042	0.222681437	2.247246145	0.032689595	0.163008889
TNFRSF21	NIH	0.257113333	0.114429475	2.246915251	0.032713237	0.163008889
DLK1	UCL	-0.531393658	0.236271888	-2.249076951	0.032842396	0.171120008
CAPG	NIH	0.504853333	0.22540886	2.239722666	0.033230958	0.164991613
ALDH2	NIH	0.233926667	0.104448069	2.239645674	0.033236539	0.164991613
PSMG4	NIH	0.6733	0.300709014	2.23904163	0.03328036	0.164991613
HMCN2	UCL	0.479117029	0.213393062	2.245232455	0.033387778	0.17365209
GSR	NIH	0.18426	0.082396732	2.236253752	0.033483281	0.165702206
GIT1	NIH	0.45878	0.205223096	2.235518366	0.033536994	0.165702206
GLI2	NIH	0.285086667	0.127604801	2.234137464	0.033638064	0.16592131
BGN	NIH	0.310026667	0.138891538	2.232149422	0.033784054	0.166360871
ITGB6	UCL	-0.302712691	0.137737601	-2.197749123	0.03382783	0.175590106
GGH	UCL	-0.302528069	0.135637911	-2.230409381	0.03388054	0.175590106
GZMB	NIH	-0.431353333	0.193450929	-2.229781663	0.033958673	0.166732889
PDIA4	NIH	0.241913333	0.108501767	2.229579663	0.033973607	0.166732889
NAP1L4	NIH	0.575106667	0.258113066	2.228119158	0.034081766	0.166983525
RELT	UCL	-0.271987631	0.122028787	-2.228880884	0.03412572	0.176547754
STOML2	NIH	-0.741286667	0.332822978	-2.227270095	0.034144786	0.167012539
LXN	NIH	0.328986667	0.147824222	2.22552612	0.034274558	0.167367417
EGFL7	NIH	0.308766667	0.138823213	2.224171735	0.034375647	0.167581279
MAN1A2	UCL	0.242499394	0.108520985	2.234585259	0.034421446	0.17776305
RWDD1	NIH	0.417213333	0.187771452	2.22192101	0.034544231	0.167911039
RNASE1	NIH	0.339773333	0.152931443	2.221736269	0.034558101	0.167911039
ZBTB17	NIH	0.367626667	0.165846108	2.216673462	0.034940176	0.169485928
CCL17	UCL	-0.681555667	0.308120248	-2.211979482	0.034949528	0.180171906
B4GAT1	UCL	0.225930918	0.102354548	2.207336385	0.035035708	0.180238706
HDGFL2	UCL	-0.474773952	0.216819632	-2.189718463	0.03508581	0.180238706
IL13RA1	NIH	0.205933333	0.092991198	2.214546507	0.035101822	0.169757042
DCTN2	NIH	0.417106667	0.188360092	2.214411033	0.03511214	0.169757042
PRDX3	UCL	-0.646940224	0.295645965	-2.188226122	0.035219345	0.180607272
HSBP1	NIH	0.39734	0.179673722	2.211453046	0.035338121	0.170567665
DNAJC21	UCL	-0.193562545	0.089020486	-2.174359573	0.035547813	0.181972431
PDGFB	NIH	0.376313333	0.170411375	2.208264165	0.035583206	0.171186461

ESAM	NIH	0.206326667	0.093433962	2.208261994	0.035583374	0.171186461
NME3	UCL	0.186714577	0.084266821	2.215754365	0.035644372	0.182147728
SSH3	UCL	-0.219889734	0.101282153	-2.171061024	0.03571643	0.182197425
SMAD5	NIH	0.134953333	0.061167915	2.206276488	0.035736744	0.171641995
CD38	NIH	-0.31572	0.143252377	-2.203942494	0.035917792	0.172007991
RBM17	UCL	-0.48700722	0.226563739	-2.149537353	0.03592693	0.182951943
ERBB3	NIH	0.22162	0.100563654	2.203778319	0.035930558	0.172007991
FABP5	NIH	0.44138	0.200577274	2.200548402	0.036182543	0.172931273
CD74	NIH	0.32104	0.145985499	2.199122533	0.036294288	0.17318237
SOD3	UCL	0.211997574	0.096578869	2.195072028	0.03634176	0.184742549
LAG3	UCL	0.387035474	0.176400387	2.194073833	0.036414129	0.184789061
RBP1	NIH	0.356566667	0.162356853	2.196191041	0.036525002	0.173999401
DNPH1	UCL	-0.419647668	0.193493716	-2.168792234	0.036583245	0.18512404
PBLD	UCL	-0.505014969	0.231661973	-2.179964901	0.036606806	0.18512404
GRPEL1	NIH	0.386313333	0.176085527	2.193896006	0.036706545	0.174579907
SYT1	NIH	-0.235906667	0.107595604	-2.192530719	0.036814926	0.174811132
MTHFD2	UCL	-0.147750187	0.069127805	-2.137348161	0.03695029	0.18653834
SNAP25	NIH	-0.253913333	0.115966426	-2.189541762	0.037053202	0.175361864
TFPI2	NIH	0.398906667	0.182313033	2.188031543	0.037174121	0.175361864
ACRBP	UCL	-0.23977697	0.110949522	-2.161135673	0.037190515	0.187427371
STIP1	NIH	0.55512	0.253797237	2.187257852	0.037236205	0.175361864
VASN	NIH	0.178173333	0.081462201	2.187190262	0.037241633	0.175361864
CHRDL1	NIH	0.342126667	0.156469146	2.186543966	0.037293572	0.175361864
ARAF	UCL	-0.421596454	0.196045123	-2.150507229	0.037300531	0.187658263
SKAP1	NIH	0.899766667	0.411556821	2.186251377	0.037317108	0.175361864
NELL2	NIH	-0.21846	0.099960715	-2.185458561	0.037380947	0.175361864
LTBP3	NIH	0.861046667	0.394074867	2.184982446	0.037419332	0.175361864
USO1	NIH	0.49426	0.226273401	2.184348657	0.037470484	0.175361864
NUMB	UCL	-0.768723946	0.357154352	-2.152357775	0.037584781	0.188763428
IGBP1	NIH	0.78362	0.359173742	2.181729644	0.037682524	0.176072497
NCR3LG1	NIH	0.309693333	0.142047614	2.180207928	0.037806219	0.176368723
S100A4	NIH	0.4619	0.212157178	2.177159422	0.038055116	0.176682307
CCN2	NIH	0.34166	0.156951336	2.176853084	0.038080208	0.176682307

NOTCH2	NIH	-0.27352	0.125649746	-2.176844836	0.038080884	0.176682307
SCIN	NIH	0.691626667	0.317780639	2.176427957	0.038115055	0.176682307
ST6GAL1	NIH	0.46752	0.214930007	2.17521977	0.038214244	0.176861808
GET3	NIH	0.637406667	0.293151982	2.174321534	0.038288136	0.176923851
RP2	UCL	-0.29548296	0.13720058	-2.15365678	0.038588332	0.193166519
DPEP1	UCL	0.392767992	0.180484316	2.176189051	0.038593653	0.193166519
WFDC2	UCL	-0.3493111	0.16058465	-2.175245891	0.038890841	0.194321245
ADH4	NIH	0.69744	0.322125217	2.165120775	0.039052429	0.17966894
CA5A	NIH	0.699226667	0.323143527	2.163826929	0.039160995	0.17966894
IDS	NIH	0.072693333	0.033609732	2.162865589	0.039241836	0.17966894
C1QBP	NIH	0.515046667	0.238148802	2.162709461	0.039254979	0.17966894
VIM	NIH	0.54238	0.250805947	2.162548402	0.039268541	0.17966894
TGOLN2	NIH	0.208913333	0.096617729	2.162267071	0.039292241	0.17966894
NFU1	NIH	0.432733333	0.200151352	2.162030531	0.039312178	0.17966894
PCYT2	UCL	-0.625577383	0.292842025	-2.13622817	0.039445462	0.196438416
SWAP70	UCL	-0.231488432	0.107724029	-2.148902467	0.039478039	0.196438416
AK1	NIH	0.7837	0.362844741	2.15987697	0.039494108	0.180038549
FNDC1	NIH	0.348246667	0.16125393	2.159616614	0.039516153	0.180038549
TNFSF14	UCL	-0.372638656	0.174035758	-2.141161452	0.039516178	0.196438416
STAU1	NIH	0.539273333	0.249815023	2.158690566	0.039594655	0.180115654
ERI1	UCL	-0.250067173	0.117336657	-2.13119395	0.039724323	0.197137857
QPCT	NIH	0.241086667	0.111778231	2.15683022	0.039752779	0.180139875
PRUNE2	NIH	0.32592	0.151136261	2.156464619	0.039783921	0.180139875
MDGA1	NIH	0.607933333	0.281913291	2.156455024	0.039784738	0.180139875
IL1RL1	NIH	0.438626667	0.20357611	2.154607765	0.039942422	0.180574319
MAX	UCL	-0.930974129	0.438966618	-2.120831267	0.039945799	0.197853643
WFIKKN2	UCL	0.287234619	0.133166705	2.156955207	0.040003935	0.197853643
PODXL2	UCL	0.22234593	0.10303935	2.157873962	0.040349143	0.199223896
CASP10	NIH	0.745053333	0.346735245	2.148767236	0.040444658	0.182307752
AMPD3	NIH	0.505466667	0.235243058	2.148699607	0.040450506	0.182307752
GIPC2	UCL	-0.221195794	0.102704591	-2.153708921	0.04055273	0.199891452
CCL15	NIH	0.472906667	0.220266115	2.146978744	0.040599578	0.182494017
CDH2	NIH	0.408053333	0.190076709	2.146782403	0.040616617	0.182494017

IGFBP3	NIH	0.26136	0.12181857	2.145485706	0.040729311	0.182719685
TNN	NIH	0.334086667	0.155962	2.142102997	0.041024612	0.183762621
YOD1	UCL	-0.538947403	0.254775338	-2.115382938	0.041029604	0.201901569
RGMB	UCL	-0.190700618	0.088942081	-2.144098897	0.041115897	0.201986164
ITGAM	NIH	-0.264686667	0.123641021	-2.140767398	0.041141732	0.184005453
F2R	NIH	0.235913333	0.110246846	2.139864688	0.04122106	0.184078781
DHPS	UCL	-0.234354416	0.112279742	-2.08723686	0.041429827	0.203186884
TNFSF13	UCL	0.205513123	0.096155169	2.137307085	0.041571077	0.203538122
NT5C3A	NIH	0.638653333	0.29903758	2.1356959	0.041589179	0.185214047
HRC	NIH	-0.32978	0.154423763	-2.135552154	0.041601924	0.185214047
MSRA	UCL	-0.559910199	0.266581053	-2.100337559	0.041732413	0.203831286
BCAN	NIH	-0.235593333	0.110407625	-2.133850197	0.041753096	0.185604568
REG3G	UCL	-0.348053913	0.16512853	-2.107775763	0.04181722	0.203831286
ACADSB	UCL	-0.735530964	0.351774786	-2.090914393	0.041840155	0.203831286
PDIA2	NIH	-0.375493333	0.176098023	-2.132297264	0.041891458	0.185937048
GCLM	NIH	0.543106667	0.254897322	2.130688007	0.04203527	0.186277082
PKLR	NIH	0.7883	0.370191208	2.129440094	0.042147093	0.186277082
CTSE	NIH	0.48168	0.226214546	2.129306045	0.042159121	0.186277082
MAMDC4	UCL	0.549844728	0.25772019	2.133494968	0.042317281	0.205812668
NMNAT1	NIH	0.828913333	0.389821356	2.126392823	0.042421269	0.187152657
FOLH1	NIH	0.63962	0.300914557	2.125586766	0.042494059	0.187191448
SFRP1	UCL	0.657400316	0.313499548	2.096973731	0.042743075	0.207538218
HNRNPUL1	NIH	0.574526667	0.270898517	2.120818794	0.042926904	0.188813827
HYAL1	UCL	0.203492177	0.095925361	2.121359519	0.043044548	0.208655414
NXPH1	NIH	-0.37272	0.175936957	-2.118486117	0.043140096	0.18946664
ADGRD1	UCL	-0.27685031	0.130922202	-2.114616976	0.043233205	0.209222944
OTUD6B	NIH	0.51842	0.244986316	2.116118194	0.043357474	0.189815866
SH3GLB2	NIH	0.686886667	0.324660332	2.115708629	0.043395171	0.189815866
NEXN	NIH	0.4161	0.196743021	2.114941601	0.043465849	0.189815866
KIT	NIH	-0.25292	0.119611754	-2.114507907	0.043505857	0.189815866
SDCCAG8	NIH	0.447713333	0.211775525	2.114093842	0.043544084	0.189815866
FKBP4	UCL	-0.460413085	0.219649712	-2.096124237	0.043649796	0.210889839
SIRPB1	NIH	0.349666667	0.165560559	2.112016709	0.043736303	0.190370068

ST13	UCL	-0.422586454	0.202571748	-2.086107552	0.043753398	0.211041557
PSMG3	NIH	0.773026667	0.36634332	2.11011536	0.043912916	0.190728623
BOLA2_BOLA2B	NIH	0.59242	0.280804137	2.109726751	0.043949091	0.190728623
NDST1	NIH	0.40304	0.191213622	2.107799619	0.044128878	0.190815824
ST13	NIH	0.700686667	0.332446723	2.107666038	0.044141365	0.190815824
TGFBR1	NIH	0.20932	0.099325505	2.107414413	0.044164893	0.190815824
GNE	UCL	-0.425365878	0.20352655	-2.089977338	0.044242954	0.212720127
CD109	NIH	-0.2758	0.130974918	-2.105746693	0.04432112	0.191208373
GAS2	NIH	0.46884	0.222738081	2.104893771	0.044401209	0.191271777
FGF16	UCL	-0.706540346	0.335824941	-2.103894795	0.044434153	0.212720127
CSNK2A1	UCL	-0.25256632	0.122133269	-2.067956772	0.044463716	0.212720127
BRD1	UCL	0.168538436	0.081978994	2.055873443	0.044467713	0.212720127
THTPA	UCL	-0.643249224	0.309016975	-2.081598349	0.044470208	0.212720127
SEL1L	UCL	-0.176944214	0.084271845	-2.09968364	0.044538049	0.212720127
EFEMP1	NIH	0.309513333	0.147188437	2.102837282	0.044594842	0.191340796
CCDC134	NIH	0.415786667	0.197780096	2.102267496	0.044648624	0.191340796
FKBP7	UCL	-0.440024802	0.212710458	-2.068656176	0.044696156	0.21312702
STAB2	NIH	0.224513333	0.106901393	2.100190913	0.04484512	0.191340796
HK2	NIH	1.43398	0.682842039	2.100017162	0.044861596	0.191340796
STX3	NIH	0.437986667	0.208618984	2.099457382	0.044914714	0.191340796
ATP6V1G1	NIH	0.4227	0.201345139	2.09938021	0.044922042	0.191340796
CDH5	NIH	-0.26038	0.124035778	-2.099233006	0.044936021	0.191340796
VSTM1	NIH	0.68378	0.3257359	2.099185261	0.044940556	0.191340796
MYDGF	UCL	-0.725641491	0.350500207	-2.0703026	0.045172681	0.215048447
NEFL	UCL	-0.371623851	0.17756706	-2.092864806	0.04526218	0.215124151
CES3	NIH	0.472446667	0.225434563	2.095715319	0.045271239	0.192168299
AARSD1	NIH	0.724646667	0.345832088	2.095371398	0.045304132	0.192168299
ATG4A	NIH	0.805513333	0.384619104	2.094314412	0.045405356	0.192168299
FRZB	NIH	0.196693333	0.093937233	2.093880421	0.045446976	0.192168299
TPMT	NIH	0.446426667	0.213222863	2.093709187	0.045463406	0.192168299
CRADD	NIH	0.5046	0.241218977	2.091875215	0.045639716	0.192600555
CFI	NIH	0.187586667	0.089714163	2.090937039	0.045730142	0.192600555
PAK4	NIH	0.762706667	0.364853676	2.090445341	0.045777598	0.192600555

FGF16	NIH	0.822166667	0.393397612	2.089912704	0.045829055	0.192600555
DTX3	NIH	0.181233333	0.086770466	2.08865229	0.045951025	0.192836079
PER3	UCL	-0.791720972	0.38270926	-2.068726983	0.046129792	0.218891853
SF3B4	NIH	0.376826667	0.180618766	2.086309608	0.046178491	0.193513016
BRD1	NIH	0.660826667	0.316953089	2.084935244	0.046312401	0.193796529
LACTB2	UCL	-0.410263109	0.199867275	-2.052677757	0.046385227	0.219747193
SNRPB2	NIH	0.669413333	0.3214051	2.082771348	0.046523938	0.193812233
HNRNPK	UCL	-0.290397628	0.141038436	-2.058996367	0.046528	0.220066898
DPT	NIH	0.263433333	0.126490541	2.082632666	0.046537524	0.193812233
PPM1B	NIH	0.419086667	0.201259351	2.082321468	0.046568024	0.193812233
SOD3	NIH	-0.19314	0.092792312	-2.081422434	0.046656237	0.193812233
ITGA11	NIH	-0.38124	0.183191404	-2.081102018	0.046687712	0.193812233
FBP1	NIH	0.76726	0.368726548	2.080837423	0.046713718	0.193812233
PIK3AP1	NIH	0.49442	0.23796618	2.0776902	0.047024029	0.19482335
RAB2B	UCL	-0.458161548	0.223443386	-2.050459209	0.047078011	0.222308603
SPP1	UCL	-0.387746265	0.185777579	-2.08715318	0.047163147	0.22235142
UBE2L6	NIH	0.32898	0.158651578	2.073600548	0.047429992	0.196163092
GPC1	NIH	-0.21788	0.105102948	-2.073015108	0.04748836	0.196163092
TTF2	UCL	-0.417887194	0.204228393	-2.046175788	0.047500711	0.223239416
PHLDB1	UCL	-0.331186211	0.161937287	-2.045151032	0.047516466	0.223239416
JAM3	UCL	-0.288518686	0.142435764	-2.025605636	0.047580621	0.223239416
CXCL5	NIH	-1.121146667	0.541111201	-2.071933948	0.047596318	0.196163092
CLSPN	NIH	0.482833333	0.233056858	2.071740504	0.047615657	0.196163092
EPN1	NIH	0.627793333	0.303237724	2.070300898	0.047759796	0.196480172
WFIKKN2	NIH	-0.302526667	0.146224751	-2.068915585	0.047898864	0.196775529
HRG	UCL	0.226765835	0.110381163	2.054388898	0.047963649	0.224675873
ADAMTS16	NIH	0.444473333	0.214949501	2.06780351	0.048010761	0.196958594
FBP1	UCL	0.594730987	0.292270876	2.034862302	0.048043733	0.224690931
MENT	UCL	0.138875637	0.067837984	2.04716634	0.048175856	0.224948925
RNASET2	NIH	0.256806667	0.124348395	2.065218983	0.048271713	0.19743446
CHEK2	NIH	0.598233333	0.28973087	2.064789759	0.048315172	0.19743446
NUDT10	NIH	0.464	0.224789515	2.064153213	0.048379685	0.19743446
STC2	NIH	0.218333333	0.105810674	2.063433921	0.048452677	0.19743446

CLEC4M	NIH	0.37732	0.182870332	2.063319929	0.048464254	0.19743446
IL17RA	NIH	-0.311046667	0.150846174	-2.06201231	0.048597225	0.197700812
YWHAQ	NIH	0.268353333	0.130447438	2.05717596	0.049091842	0.199435609
SERPIND1	NIH	0.255946667	0.12456144	2.054782494	0.049338267	0.199990001
BCHE	NIH	0.27406	0.133398381	2.054447715	0.049372822	0.199990001
IFNLR1	NIH	0.486066667	0.236659955	2.053861065	0.049433426	0.199990001
APOC1	UCL	0.290167152	0.141113471	2.05626826	0.049519879	0.230406753
JPT2	UCL	-0.749796178	0.369615243	-2.028585648	0.049563898	0.230406753
SNAP23	UCL	-0.795180834	0.394368358	-2.016340352	0.0495812	0.230406753
MYDGF	NIH	0.511333333	0.249235885	2.051603982	0.049667208	0.20040765
PROS1	UCL	-0.163472027	0.079582051	-2.05413188	0.049671233	0.230458753
HAO1	NIH	1.051226667	0.512408183	2.051541527	0.049673691	0.20040765
LILRA2	NIH	0.33932	0.165733924	2.047378057	0.050107546	0.201879577
OXT	NIH	1.2327	0.602399868	2.046315189	0.050218838	0.202049657
SCRG1	UCL	-0.220556845	0.107602249	-2.049741953	0.050302713	0.232842828
SH2D1A	UCL	-0.224604241	0.110441229	-2.033699219	0.050344395	0.232842828
PYDC1	UCL	-0.305391415	0.14992892	-2.036907993	0.050487642	0.233136459
HMGCL	NIH	0.440046667	0.215483709	2.042134274	0.050658737	0.20353957
TRAF3	NIH	0.450026667	0.220446837	2.041429454	0.05073323	0.203559256
CAT	NIH	0.563813333	0.276640163	2.038074759	0.051089116	0.204706388
MTSS2	UCL	-0.741134282	0.368621065	-2.01055868	0.05109173	0.235553827
BLMH	NIH	0.285733333	0.140243104	2.037414501	0.051159419	0.20470766
PTRHD1	UCL	-0.474477901	0.236925596	-2.002645177	0.051284374	0.235694498
IL1RL1	UCL	0.397638627	0.19504165	2.03873699	0.051304641	0.235694498
CYB5A	UCL	-0.445422406	0.220136869	-2.023388485	0.051364145	0.235694498
KCNC4	UCL	-0.329230852	0.164055704	-2.006823561	0.051484423	0.235876126
DNAJB2	NIH	0.462366667	0.227556973	2.031872106	0.051752935	0.206717387
MAP2K6	UCL	-0.754501614	0.375532453	-2.009151561	0.0517975	0.236939113
C1GALT1C1	NIH	0.345986667	0.170368099	2.030818377	0.05186646	0.206717387
HEG1	NIH	-0.212433333	0.104608248	-2.030751278	0.051873696	0.206717387
IL16	NIH	0.320633333	0.157945312	2.030027542	0.051951806	0.206746982
LONP1	UCL	-0.692855937	0.347549526	-1.993545915	0.051999402	0.23749102
CHMP1A	NIH	0.435006667	0.214417582	2.028782632	0.052086406	0.206780497

NFX1	NIH	0.503106667	0.248001685	2.028642133	0.052101616	0.206780497
NOTCH2	UCL	0.181217908	0.08937558	2.027599799	0.052174237	0.237917778
CIRBP	UCL	-0.824506654	0.412287791	-1.999832814	0.052283678	0.238020801
CNPY2	NIH	0.955346667	0.471390938	2.026654715	0.052317188	0.20735471
MTSS1	UCL	-0.685041929	0.342317151	-2.001190785	0.05242124	0.238020801
FABP5	UCL	-0.588795684	0.291714465	-2.018397287	0.052441121	0.238020801
CNPY4	NIH	0.493246667	0.243603393	2.02479391	0.052519739	0.207875829
EIF5	UCL	-0.306460743	0.154368919	-1.985249004	0.052680134	0.238734935
APOH	NIH	0.287833333	0.14231871	2.022456034	0.052775199	0.208604673
SEMA3F	UCL	0.185678999	0.091769694	2.023315015	0.052902485	0.239371461
PPM1F	NIH	0.225946667	0.111797399	2.021036881	0.052930804	0.208937384
TNFRSF10B	NIH	0.2771	0.137338185	2.017647165	0.053304109	0.209998463
F13B	NIH	0.211493333	0.104867706	2.01676322	0.053401837	0.209998463
NECTIN2	NIH	0.230866667	0.114493605	2.016415388	0.053440336	0.209998463
SESTD1	UCL	-0.481171271	0.242206751	-1.986613785	0.053477333	0.24115284
ARFIP1	NIH	0.392973333	0.194927636	2.015995994	0.053486788	0.209998463
DPY30	UCL	-0.350514821	0.174034036	-2.014059026	0.053534096	0.24115284
LBR	UCL	-0.454084471	0.228063024	-1.991048189	0.053543686	0.24115284
VSIG10	UCL	0.268969451	0.13410394	2.005678967	0.053758927	0.241718796
SORD	NIH	0.626213333	0.31100459	2.01351798	0.05376198	0.210546312
LCN15	NIH	0.441726667	0.219388676	2.013443334	0.053770289	0.210546312
NELL1	UCL	0.267031832	0.132307595	2.018265326	0.053834737	0.241718796
WNT9A	NIH	-0.29566	0.146953221	-2.011932767	0.053938676	0.210923299
APBB1IP	NIH	0.411066667	0.204484461	2.010258696	0.054125831	0.211268925
VCAM1	NIH	0.228253333	0.113567302	2.009850798	0.054171519	0.211268925
SUMF2	NIH	0.44872	0.223376581	2.008805035	0.054288808	0.211318971
MCTS1	NIH	0.244253333	0.121612941	2.008448536	0.054328843	0.211318971
C2orf69	UCL	-0.348929769	0.174941462	-1.994551576	0.054369295	0.243717992
IGFBP1	UCL	0.963724433	0.481223563	2.002654289	0.054495779	0.243717992
ANGPTL4	NIH	0.347693333	0.173277879	2.006565037	0.054540791	0.211861637
SLC1A4	UCL	-0.350315145	0.17841699	-1.96346292	0.054565162	0.243717992
ITM2A	UCL	-0.412786847	0.206870522	-1.995387472	0.054613508	0.243717992
B2M	NIH	0.239653333	0.11958325	2.004071088	0.05482255	0.212596298

NRCAM	UCL	0.29522642	0.146866099	2.010174039	0.05484609	0.244382804
IGFBP6	NIH	0.203966667	0.10183161	2.002979878	0.054946233	0.212596298
RPA2	NIH	0.374233333	0.186839714	2.0029646	0.054947966	0.212596298
CALCA	NIH	0.51856	0.259219212	2.00046901	0.055231759	0.213412016
C1QA	NIH	0.12358	0.061820314	1.999019303	0.055397207	0.213545061
LZTFL1	UCL	-0.501440751	0.254610183	-1.969444996	0.055411902	0.246528141
NCF2	NIH	0.63986	0.320107969	1.998888072	0.055412206	0.213545061
GFRAL	UCL	0.439675995	0.219374638	2.004224364	0.055556792	0.246725197
PRND	UCL	0.413184857	0.207217375	1.993968203	0.05562501	0.246725197
DDX1	NIH	0.456346667	0.228576648	1.996471079	0.055689079	0.21432968
SLMAP	UCL	-0.604553798	0.302393594	-1.999228193	0.055842266	0.24731355
PPP1R14A	UCL	-0.5668233	0.288658685	-1.963645404	0.055964705	0.247480836
CD207	NIH	0.317766667	0.159425343	1.993200464	0.056065671	0.215495517
MORF4L2	UCL	-0.244921498	0.122134425	-2.005343687	0.056168547	0.248007044
PODXL	NIH	-0.115066667	0.057781867	-1.991397517	0.056274224	0.215650476
DCC	UCL	-0.185116646	0.094174415	-1.965678734	0.056304841	0.24823386
CXCL3	NIH	-0.960053333	0.482396869	-1.990173229	0.056416229	0.215650476
ACVRL1	UCL	-0.171550256	0.086428128	-1.984889176	0.056606248	0.249186843
DAND5	NIH	1.415193333	0.711691003	1.988494063	0.056611507	0.215650476
PDIA3	NIH	0.26452	0.133042533	1.988236347	0.056641531	0.215650476
GART	NIH	-0.248073333	0.124772871	-1.988199285	0.05664585	0.215650476
HAVCR2	NIH	0.2926	0.147205607	1.987696025	0.056704522	0.215650476
CCN3	NIH	0.324513333	0.163265195	1.987645525	0.056710413	0.215650476
CXCL11	NIH	-0.693133333	0.348809397	-1.987140656	0.056769332	0.215650476
GPHA2	NIH	0.3653	0.183832134	1.987138989	0.056769527	0.215650476
PRSS8	NIH	0.37082	0.186695769	1.986226052	0.056876206	0.215775489
ITGB1	NIH	-0.15474	0.077970615	-1.984593813	0.057067375	0.216220299
OMD	NIH	-0.315586667	0.159170453	-1.982696294	0.057290321	0.21657688
ECSCR	NIH	0.3571	0.18012313	1.982532731	0.057309574	0.21657688
RAB37	UCL	-0.589598019	0.296430855	-1.988990043	0.057390786	0.251502601
RANBP2	UCL	-0.376252145	0.184546289	-2.038795504	0.057445709	0.251502601
BPIFA2	UCL	0.57764098	0.2919469	1.97858234	0.057470575	0.251502601
OBP2B	UCL	0.491688849	0.247568888	1.986068815	0.057476475	0.251502601

TMED1	NIH	-0.678973333	0.34273252	-1.98105897	0.05748331	0.216904818
DPP10	NIH	-0.2467	0.124562022	-1.980539455	0.057544663	0.216904818
BAIAP2	UCL	-0.646184072	0.327322568	-1.974150686	0.057672101	0.251981392
KCNH2	UCL	0.713097265	0.360438335	1.978416821	0.057866286	0.252452467
TNFAIP8L2	UCL	-0.386165484	0.198702656	-1.943433928	0.058262848	0.25348983
CASP9	UCL	-0.468741674	0.24134029	-1.942243766	0.058277511	0.25348983
PREB	UCL	0.249737998	0.12679151	1.969674457	0.058379175	0.253554721
LGALS9	NIH	0.30828	0.156218623	1.973388279	0.058395045	0.219630985
OGFR	NIH	0.19068	0.096635114	1.973195782	0.058418088	0.219630985
DNAJA4	NIH	0.559286667	0.283986265	1.969414493	0.058872328	0.221054634
PLAT	UCL	0.330307187	0.168641542	1.958634762	0.058873627	0.255322866
CHAC2	UCL	-0.47343487	0.242448786	-1.952721139	0.059039398	0.255662461
CTF1	UCL	-0.618598896	0.317503649	-1.94832059	0.059172755	0.255860893
NACC1	NIH	0.3196	0.162632788	1.965163388	0.059386695	0.221666834
VMO1	NIH	0.585586667	0.297993735	1.965097244	0.059394729	0.221666834
C8B	NIH	0.230766667	0.117437839	1.965011178	0.059405184	0.221666834
GP2	NIH	-0.775233333	0.394660952	-1.964302092	0.059491386	0.221666834
FKBP1B	UCL	-0.305552881	0.1580017	-1.933858193	0.059504196	0.256871757
STXBP3	NIH	0.201526667	0.102606727	1.964068759	0.059519775	0.221666834
ERMAP	NIH	0.320013333	0.162941319	1.963979024	0.059530696	0.221666834
F9	NIH	0.26156	0.133198199	1.963690221	0.059565857	0.221666834
EGF	UCL	-0.901732014	0.464996519	-1.939223151	0.059582296	0.256871757
TDO2	NIH	0.352346667	0.179520337	1.962711703	0.059685122	0.221828441
UNC79	UCL	-0.308594202	0.159282178	-1.937405723	0.059741463	0.256942643
THRAP3	UCL	0.806552682	0.413445123	1.95080952	0.059774546	0.256942643
MRPL52	NIH	0.1859	0.09481094	1.960744195	0.059925562	0.222439427
AGR2	UCL	-0.760821739	0.392911084	-1.936371282	0.060211779	0.25844204
RET	UCL	-0.325761996	0.166398291	-1.957724411	0.06048995	0.259255313
SERPINA6	NIH	0.15944	0.081551869	1.955074741	0.060623138	0.224743572
AZI2	NIH	0.392673333	0.201034499	1.953263421	0.060847495	0.225060847
IL15RA	NIH	0.289946667	0.14848769	1.952664669	0.060921818	0.225060847
TCOF1	NIH	0.24212	0.124003731	1.952521892	0.060939552	0.225060847
TOP1	UCL	-1.017783302	0.519599489	-1.958784262	0.060997169	0.261046449

PM20D1	UCL	-1.372175578	0.702333978	-1.953736571	0.061369625	0.262083917
TYRO3	UCL	0.216614212	0.110956044	1.952252475	0.061418913	0.262083917
SLC51B	NIH	0.468706667	0.240751286	1.946850108	0.06164771	0.227389095
AHNAK2	NIH	0.284373333	0.146150135	1.94576169	0.061784424	0.227606349
TRAF2	UCL	-0.510113079	0.264375802	-1.929499885	0.061855809	0.263563453
ABO	NIH	1.56678	0.805814915	1.944342268	0.061963112	0.227815968
XG	NIH	0.224493333	0.115522261	1.94329069	0.062095784	0.227815968
DYNLT1	NIH	0.4868	0.250546362	1.942953777	0.062138342	0.227815968
PDLIM5	NIH	0.536986667	0.276396722	1.942811268	0.062156352	0.227815968
ASGR1	UCL	-0.265639319	0.136519736	-1.945794267	0.062300311	0.264725588
CD8A	UCL	0.439868351	0.226363371	1.943195796	0.062309683	0.264725588
EIF4E	NIH	0.72974	0.375871387	1.941461959	0.062327094	0.227815968
CBS	NIH	0.557933333	0.287504247	1.940609014	0.062435237	0.227815968
TF	NIH	0.176933333	0.091182962	1.940420998	0.062459097	0.227815968
BNIP2	UCL	-0.205812404	0.10792802	-1.906941351	0.062510756	0.264945618
UROS	NIH	0.79372	0.409160738	1.939873321	0.062528645	0.227815968
FSTL1	NIH	0.14712	0.075844205	1.939765868	0.062542298	0.227815968
THY1	NIH	0.269653333	0.139096213	1.938610165	0.062689307	0.228067443
ADIPOQ	UCL	0.428268312	0.220671909	1.940746847	0.062702741	0.264945618
CLEC1B	UCL	-0.600848026	0.313182989	-1.918520631	0.062718481	0.264945618
EHD3	UCL	-0.963086174	0.503400483	-1.913161005	0.062769125	0.264945618
MYL6B	UCL	0.496725407	0.257265905	1.930785998	0.062814681	0.264945618
APOL1	NIH	-0.62296	0.321515894	-1.937571397	0.062821699	0.228210167
GALNT3	NIH	0.411893333	0.212636345	1.937078695	0.062884579	0.228210167
CD99	NIH	0.124153333	0.064160408	1.935045896	0.063144592	0.228424395
BAG4	NIH	0.37318	0.1928862	1.934715913	0.063186888	0.228424395
FGFBP1	NIH	-0.36282	0.187617839	-1.93382464	0.063301252	0.228424395
PLXDC2	NIH	-0.470253333	0.243201328	-1.933596898	0.063330503	0.228424395
GABARAPL1	NIH	0.344653333	0.178247233	1.933569056	0.06333408	0.228424395
CAMSAP1	NIH	0.456353333	0.236137977	1.932570687	0.063462457	0.228507876
CD300C	NIH	0.308153333	0.159493895	1.932069772	0.063526954	0.228507876
VWF	NIH	-0.468346667	0.2425107	-1.931241246	0.063633758	0.228507876
TNXB	UCL	0.238375898	0.123225942	1.934461969	0.06373659	0.268446761

SCRIB	NIH	0.324493333	0.168124642	1.930075977	0.063784236	0.228507876
DEFA1_DEFA1B	NIH	0.5899	0.305677749	1.929810078	0.063818616	0.228507876
CPTP	NIH	0.203513333	0.105482354	1.929359046	0.063876971	0.228507876
PRDX6	NIH	0.358346667	0.185753693	1.929149618	0.063904083	0.228507876
PHACTR2	NIH	0.435506667	0.225881874	1.92802839	0.064049403	0.228747868
PSMG3	UCL	-0.317977221	0.16735473	-1.900019329	0.064306749	0.270458456
SCLY	NIH	0.408673333	0.212316445	1.924831276	0.064465355	0.229952638
ILKAP	NIH	0.4764	0.247720184	1.923137603	0.064686656	0.230460986
PLA2G1B	NIH	-0.48856	0.254155584	-1.922287101	0.064798035	0.230576949
EFHD1	UCL	0.228426458	0.119344847	1.914003529	0.064946774	0.272757787
PPCDC	NIH	0.343586667	0.178973357	1.919764334	0.065129389	0.231474438
CHMP6	UCL	-0.347023991	0.183514108	-1.890993534	0.065176979	0.273331865
PNPT1	UCL	-0.863833458	0.447584349	-1.929990315	0.065481536	0.273890822
UMOD	UCL	0.338737306	0.176336331	1.920972855	0.065497668	0.273890822
GCNT1	NIH	0.247186667	0.128949772	1.916922098	0.065504467	0.232342131
CASP9	NIH	0.456853333	0.238352744	1.916711028	0.065532396	0.232342131
MERTK	NIH	0.217546667	0.113542704	1.915989829	0.065627903	0.232399052
GIPR	NIH	0.450466667	0.235265953	1.914712521	0.065797352	0.232717356
CA2	NIH	1.05126	0.549285404	1.91386844	0.065909537	0.232832602
EPHA1	UCL	-0.240195373	0.125654883	-1.911548263	0.065998582	0.27521937
NDRG1	UCL	-0.850921942	0.442740463	-1.921943021	0.066045608	0.27521937
PTPRN2	NIH	-0.20646	0.107945288	-1.912635597	0.066073689	0.232889204
IL6	NIH	0.668046667	0.349295904	1.912552251	0.0660848	0.232889204
NAGPA	UCL	0.142471992	0.074550328	1.911084713	0.066113503	0.27521937
HGFAC	NIH	0.185526667	0.09705114	1.911638194	0.066206753	0.23303821
DUSP13	UCL	-0.27918766	0.146629915	-1.904029337	0.066282237	0.27521937
TADA3	UCL	-0.321786211	0.1702228	-1.890382559	0.066286157	0.27521937
BAMBI	NIH	0.272793333	0.142749651	1.910991248	0.066293187	0.233061984
TRPV3	UCL	0.443687216	0.233794627	1.897764812	0.06638083	0.275221511
MEGF9	NIH	0.15908	0.083297525	1.909780636	0.066455191	0.233294319
ITM2A	NIH	-0.435553333	0.228121274	-1.909306072	0.06651879	0.233294319
TMED4	UCL	0.369760491	0.194272185	1.903311534	0.066769079	0.276439119
IL22RA1	NIH	0.657253333	0.3446854	1.906820924	0.066852707	0.234184632

FLRT2	NIH	0.257253333	0.13495443	1.906223709	0.066933168	0.234186024
GAD2	NIH	0.327773333	0.172138182	1.904129173	0.067216024	0.234894706
GALNT2	UCL	0.207581409	0.109271064	1.8996924	0.06722147	0.27791847
FUOM	NIH	0.440986667	0.231810759	1.902356341	0.067456244	0.235288309
YTHDF3	NIH	0.51982	0.273285827	1.902111085	0.067489535	0.235288309
LZTFL1	NIH	0.34382	0.18084159	1.901221953	0.067610346	0.235428882
RBM17	NIH	0.56752	0.29866993	1.900157811	0.067755182	0.23563725
OPTC	NIH	-0.347586667	0.182978939	-1.899599315	0.067831304	0.23563725
DIPK1C	UCL	-0.351927409	0.186974895	-1.882217445	0.067900685	0.280330087
SCGB3A1	UCL	0.223609236	0.117699469	1.899832155	0.068073823	0.280648498
MYH9	NIH	0.502053333	0.264662486	1.896956919	0.068192465	0.236610866
LMOD1	UCL	-0.259020419	0.137337765	-1.886010154	0.06835737	0.281158996
SUMF1	UCL	0.549846426	0.28992182	1.896533435	0.068390026	0.281158996
BCL2	NIH	0.50944	0.268928914	1.894329592	0.068553217	0.237580759
FUT1	NIH	-0.460073333	0.243052702	-1.892895369	0.068750844	0.237580883
PCDH1	NIH	0.12538	0.066256957	1.892329573	0.068828943	0.237580883
EBAG9	NIH	0.482126667	0.254782674	1.892305542	0.068832261	0.237580883
SERPINB1	NIH	0.476633333	0.251974955	1.891590115	0.068931128	0.237580883
NFIC	NIH	0.2919	0.154342625	1.891246824	0.068978612	0.237580883
METAP1	NIH	0.423533333	0.224025531	1.890558327	0.06907393	0.237580883
TFAP2A	UCL	0.462662055	0.244691958	1.890793873	0.069178106	0.283126584
F2R	UCL	-0.339121644	0.181601232	-1.867397269	0.069226919	0.283126584
CRLF1	NIH	0.201093333	0.106430252	1.889437723	0.069229315	0.237580883
CR1	NIH	0.252026667	0.133389481	1.889404356	0.069233946	0.237580883
PDCD6	UCL	0.427199017	0.225556488	1.893977959	0.069253392	0.283126584
PPCDC	UCL	-0.306713008	0.163662161	-1.874061831	0.069265779	0.283126584
MAPK13	NIH	-0.5379	0.284782091	-1.888812592	0.069316128	0.237580883
LPP	UCL	-0.438086179	0.235266327	-1.862086193	0.069395067	0.283126584
SPINK6	UCL	-0.401861345	0.213370537	-1.883396603	0.069449798	0.283126584
RANBP1	NIH	0.711633333	0.37699696	1.887636796	0.069479668	0.237580883
ERCC1	NIH	-0.208713333	0.110581963	-1.88740846	0.069511466	0.237580883
SERPINI2	NIH	-0.50276	0.266392529	-1.887290168	0.069527944	0.237580883
AKR1B1	NIH	0.46414	0.246108687	1.885914738	0.06971979	0.237958443

IFT20	NIH	0.268586667	0.142584523	1.883701411	0.070029468	0.238577489
ASRGL1	UCL	-0.712145313	0.382115272	-1.863692363	0.070056642	0.285202736
FGF5	NIH	0.286773333	0.15225935	1.883453025	0.070064295	0.238577489
NPTX2	NIH	-0.26788	0.142289512	-1.882640518	0.070178325	0.238687906
SPINK6	NIH	-0.363566667	0.193226045	-1.881561391	0.07033002	0.238926026
DDX58	UCL	-0.386869675	0.207594537	-1.86358312	0.070493431	0.286581779
PIKFYVE	UCL	-0.468751502	0.249899812	-1.875757719	0.070685	0.286961464
WFIKKN1	NIH	0.299486667	0.159415269	1.878657348	0.070739658	0.240038861
NCAN	UCL	0.239668703	0.127726847	1.87641603	0.070886015	0.287350775
MAP1LC3A	UCL	-0.2266274	0.123164268	-1.84004179	0.071063889	0.287350775
MAP1LC3B2	NIH	0.65604	0.349643293	1.876312265	0.071071953	0.240886979
TOR1AIP1	UCL	-0.325979803	0.174875487	-1.864068019	0.071075816	0.287350775
LMOD2	UCL	0.452779589	0.242628909	1.866140319	0.071310874	0.287902881
TBC1D17	NIH	0.50328	0.268504712	1.874380514	0.071346692	0.241538281
SNU13	UCL	-0.297522503	0.158037989	-1.882601167	0.071535296	0.288021781
FMR1	UCL	-0.965741703	0.525908802	-1.836329224	0.07161917	0.288021781
SERPINB6	UCL	-0.373620358	0.201486254	-1.854321829	0.071635934	0.288021781
KIF20B	NIH	-1.125526667	0.60167823	-1.870645488	0.071880501	0.242815696
TNF	NIH	0.237333333	0.126876943	1.8705789	0.071890049	0.242815696
PTN	NIH	0.773493333	0.413885409	1.868858666	0.07213709	0.243369075
NFAT5	UCL	-0.505758628	0.273405356	-1.849849014	0.072156804	0.289717497
PXN	NIH	0.513346667	0.274934212	1.86716183	0.072381488	0.243746039
ATG16L1	NIH	0.35576	0.190559225	1.866926153	0.07241549	0.243746039
DTD1	NIH	0.412446667	0.221009701	1.866192594	0.07252141	0.243821981
CSNK1D	UCL	-0.663524009	0.354645272	-1.870951232	0.072523724	0.290791281
CD160	NIH	0.40544	0.217324049	1.865601173	0.072606903	0.243829153
INSL5	NIH	0.886673333	0.475659774	1.864091481	0.072825533	0.244282895
IMPA1	UCL	-0.344313009	0.18761052	-1.835254273	0.072989593	0.292258331
TRIM21	NIH	0.409546667	0.219852214	1.862827121	0.073009071	0.244618022
NPC2	NIH	0.280946667	0.15093767	1.861342278	0.073225125	0.244899793
TMED8	NIH	0.41066	0.220655152	1.861094091	0.073261291	0.244899793
MEP1B	NIH	1.104653333	0.593732209	1.860524521	0.073344348	0.244899793
AMFR	UCL	-0.358202257	0.194629503	-1.840431442	0.073381362	0.292820598

PGA4	UCL	0.430977231	0.232360619	1.854777425	0.073396815	0.292820598
NTRK2	UCL	0.162665131	0.08832082	1.841752941	0.07343055	0.292820598
BPIFB1	UCL	0.344287677	0.186129604	1.849720137	0.07383176	0.293840158
FLT3LG	UCL	-0.46876018	0.252163169	-1.858955778	0.073969807	0.293840158
IL18R1	NIH	0.301206667	0.162266241	1.856249732	0.073970312	0.24661672
GNPDA2	UCL	-0.197652095	0.107238981	-1.843099338	0.073987806	0.293840158
DFFA	NIH	0.34452	0.185638706	1.855862971	0.074027173	0.24661672
ADM	NIH	0.262313333	0.141398378	1.855136796	0.074134035	0.246691753
CKMT1A_CKMT1B	NIH	-0.62212	0.335677959	-1.853323946	0.074401389	0.246936519
SERPINA4	NIH	0.16204	0.087450352	1.852937076	0.07445855	0.246936519
PECR	NIH	0.138006667	0.074499229	1.852457644	0.074529441	0.246936519
CHAC2	NIH	0.419266667	0.226343043	1.852350575	0.074545281	0.246936519
HNRNPK	NIH	0.830146667	0.448311338	1.851719097	0.074638759	0.246966483
ADAM9	NIH	0.25274	0.13658701	1.850395577	0.07483501	0.247300676
CLSTN1	NIH	0.277706667	0.150119961	1.84989834	0.074908854	0.247300676
ADA2	UCL	0.403862226	0.217970168	1.852832564	0.074967037	0.297325167
DNPH1	NIH	0.655253333	0.354751691	1.847075999	0.075329189	0.24840798
RARRES1	UCL	-0.183786616	0.100095211	-1.836117984	0.075370013	0.298518356
BTD	NIH	0.183066667	0.099192307	1.845573226	0.075553826	0.248868176
RABGAP1L	NIH	0.515073333	0.279345731	1.843856113	0.075811207	0.249210933
DUT	NIH	0.478786667	0.259681979	1.843742366	0.075828284	0.249210933
LAIR2	NIH	0.896786667	0.487434524	1.839809498	0.076420747	0.250876191
CBLN4	UCL	0.197722642	0.107335697	1.84209585	0.076483005	0.302516677
CSF2	UCL	-0.23697838	0.129853627	-1.824965428	0.076648846	0.302762942
DGKZ	NIH	0.33024	0.17970951	1.8376323	0.076750437	0.251426627
CDC27	NIH	0.33114	0.180205668	1.837567068	0.076760334	0.251426627
ALDH3A1	NIH	0.451473333	0.245929871	1.835780794	0.077031769	0.252033472
DBN1	UCL	-0.439900374	0.240905818	-1.826026362	0.077107999	0.304165563
SSC4D	NIH	1.220493333	0.665120585	1.83499558	0.077151348	0.252142672
TNN	UCL	0.340842238	0.185535908	1.837068849	0.077321883	0.304469025
TNFSF10	UCL	0.218262755	0.118967429	1.834642951	0.077393255	0.304469025
IL18BP	NIH	0.236353333	0.12899155	1.832316407	0.077560554	0.253197121
ADAMTS13	NIH	0.204693333	0.111775282	1.831293368	0.0777173	0.253425977

APOE	NIH	0.661553333	0.36178422	1.828585373	0.078133521	0.254499496
KLRD1	NIH	0.47122	0.257983116	1.82655364	0.078447054	0.255236522
IL33	NIH	0.336046667	0.18411839	1.825166225	0.078661777	0.255650776
TAB2	NIH	0.331393333	0.181662647	1.824223846	0.078807912	0.25573582
GZMA	NIH	0.1827	0.1001716	1.823870232	0.078862807	0.25573582
PYY	UCL	-0.739700409	0.406322195	-1.820477488	0.079050921	0.310293622
NFYA	UCL	-0.31141668	0.169969758	-1.832188761	0.079086127	0.310293622
COL4A1	NIH	-0.39856	0.218751637	-1.821974937	0.079157591	0.256052539
BAP18	NIH	0.451873333	0.248046397	1.821729076	0.0791959	0.256052539
OGA	NIH	0.34856	0.191353033	1.821554614	0.079223093	0.256052539
DNER	UCL	0.181619947	0.099666776	1.822271723	0.079563205	0.311746983
GBP6	NIH	0.493233333	0.271164578	1.818944556	0.079630882	0.257086457
HAGH	NIH	0.466006667	0.256418067	1.817370643	0.079877654	0.257258275
MRPS16	NIH	0.26596	0.146345929	1.81733788	0.079882798	0.257258275
DPP6	NIH	-0.308773333	0.169942991	-1.816923021	0.079947956	0.257258275
NCAM1	UCL	0.222963969	0.122577993	1.818955945	0.080102881	0.313441392
GRSF1	UCL	-0.392901579	0.220558185	-1.781396506	0.080270837	0.313678685
NUDT15	NIH	0.464766667	0.256153905	1.814403988	0.080344575	0.258001355
BACH1	NIH	0.3663	0.201892036	1.814336056	0.080355294	0.258001355
SUGP1	UCL	-0.219767516	0.122459	-1.794621193	0.080460539	0.314000209
PCDHB15	NIH	0.354793333	0.195706843	1.812881592	0.08058509	0.25845547
AIF1L	UCL	-0.382931014	0.210498276	-1.819164604	0.080683876	0.31445196
GADD45GIP1	NIH	0.128086667	0.070711153	1.811406834	0.080818667	0.258920701
SNX5	NIH	0.148486667	0.082053604	1.80963004	0.08110085	0.259540467
SEMA4C	UCL	0.131175638	0.07384928	1.776261565	0.081122383	0.315474003
ENOX2	UCL	-0.181185719	0.102017612	-1.776023922	0.081161974	0.315474003
PSRC1	NIH	0.411146667	0.227358534	1.808362583	0.081302657	0.259644763
MST1	NIH	0.825566667	0.456540352	1.808310399	0.081310975	0.259644763
C1QTNF5	UCL	0.220500924	0.122426988	1.801081023	0.081486679	0.316315487
ACRV1	NIH	0.630633333	0.349019278	1.806872494	0.081540462	0.260093621
CANT1	NIH	0.153486667	0.084985533	1.806032876	0.081674719	0.260202944
CRYM	NIH	0.290586667	0.160941156	1.805546042	0.081752651	0.260202944
TSPAN15	UCL	-0.213642868	0.120559517	-1.772094597	0.08181893	0.317183995

ADAMTSL4	UCL	0.135792225	0.075626345	1.795567732	0.082034437	0.317598226
PINLYP	NIH	0.332813333	0.184648821	1.802412445	0.082255802	0.261069693
ARL2BP	NIH	0.325586667	0.180710123	1.801706855	0.082369461	0.261069693
APOA4	NIH	0.294493333	0.163523226	1.800926636	0.082495297	0.261069693
SHPK	UCL	0.218317771	0.122196688	1.786609562	0.082583371	0.319300519
DKK4	NIH	0.3641	0.202256729	1.800187325	0.082614687	0.261069693
TCN2	NIH	0.184226667	0.102340926	1.80012703	0.08262443	0.261069693
NAGA	NIH	0.31536	0.17520094	1.79999034	0.082646523	0.261069693
NOTCH3	NIH	-0.3057	0.169836132	-1.799970338	0.082649756	0.261069693
PVR	NIH	0.368386667	0.204734902	1.799334956	0.082752518	0.261075378
CA7	UCL	0.255138409	0.143555277	1.777283387	0.082792969	0.319688044
MSMB	NIH	-0.429726667	0.238888877	-1.7988559	0.082830069	0.261075378
IL1RL2	NIH	0.362066667	0.201488603	1.796958545	0.083137828	0.26148536
CEP85	NIH	0.3584	0.199479269	1.796677934	0.083183427	0.26148536
MECR	NIH	0.25632	0.142685234	1.796401728	0.083228332	0.26148536
TGFA	UCL	-0.134303486	0.075759702	-1.772756247	0.083294608	0.321200709
MYOM1	NIH	0.296886667	0.16535187	1.795484174	0.083377651	0.261673423
CA12	NIH	0.207906667	0.115961199	1.792898563	0.083799657	0.262715966
BRSK2	UCL	-0.337600851	0.190229582	-1.774702164	0.084002845	0.323505029
MDM1	NIH	0.28016	0.156443278	1.790808811	0.084142063	0.263506995
TEX101	UCL	0.647869916	0.361351909	1.792905751	0.084381606	0.324536096
CCL3	NIH	0.33984	0.189976384	1.788853925	0.08446345	0.26413797
SIGLEC10	NIH	0.259433333	0.145057561	1.788485418	0.084524151	0.26413797
FLT1	NIH	-0.11534	0.064534856	-1.787251227	0.084727718	0.264211988
SIRT5	NIH	0.468506667	0.262186364	1.78692232	0.084782038	0.264211988
MMP10	NIH	-0.277633333	0.155388919	-1.786699691	0.084818823	0.264211988
CEMIP2	NIH	0.177066667	0.099166138	1.785555741	0.085008052	0.264519736
CD274	NIH	0.208326667	0.116804458	1.783550654	0.085340596	0.26527231
CIT	UCL	-0.270484177	0.152090674	-1.778440255	0.085342425	0.326627373
RRP15	UCL	0.795419165	0.446511396	1.781408431	0.085358174	0.326627373
SERPINA7	UCL	0.11954054	0.067351889	1.77486543	0.085363073	0.326627373
SPESP1	UCL	0.372805526	0.208724981	1.7861088	0.085429693	0.326627373
TREML2	UCL	-0.256588168	0.144131825	-1.780232563	0.085520284	0.326627373

CD69	UCL	-0.652248113	0.37107187	-1.757740656	0.085607335	0.326627373
NMT1	NIH	0.919233333	0.51596958	1.781564978	0.085671013	0.26601668
MRPL52	UCL	-0.157087944	0.089797398	-1.749359636	0.085707559	0.326627373
MFAP3	NIH	0.621726667	0.349085215	1.7810169	0.085762405	0.266018063
EGFLAM	NIH	0.197053333	0.110712465	1.779865825	0.085954617	0.266198733
GPR37	NIH	0.529966667	0.297804617	1.779578411	0.086002668	0.266198733
PRKRA	UCL	-0.444738749	0.252654431	-1.760264985	0.086389605	0.328712235
OGFR	UCL	-0.192478508	0.10847599	-1.774388125	0.086479545	0.328712235
IL6	UCL	-0.633022459	0.355094293	-1.782688347	0.086796452	0.329488349
STX5	NIH	0.216506667	0.12229977	1.770294967	0.087567055	0.270754372
AXIN1	NIH	0.365766667	0.206848001	1.768287173	0.087908561	0.270971266
ENTPD6	NIH	0.236393333	0.133707305	1.767991155	0.087959006	0.270971266
CILP	NIH	0.410433333	0.232177637	1.767755667	0.087999154	0.270971266
BTC	NIH	0.376353333	0.212905034	1.767705189	0.088007761	0.270971266
PEBP1	UCL	-0.352465899	0.201013823	-1.753441098	0.088318034	0.334829588
CSDE1	NIH	0.4851	0.274813468	1.765197327	0.088436324	0.272003222
LGALS3BP	NIH	0.348626667	0.197560597	1.764656875	0.088528912	0.272003222
EDDM3B	UCL	-0.521073834	0.294840317	-1.767308621	0.08861917	0.335536053
SCGB1A1	NIH	0.446266667	0.253052462	1.763534184	0.088721511	0.272066383
RCOR1	NIH	0.334246667	0.189541041	1.763452734	0.088735497	0.272066383
RGS10	UCL	-0.367057396	0.210984418	-1.739736983	0.088782758	0.335720571
DDHD2	NIH	0.244726667	0.138853311	1.762483477	0.088902083	0.272291719
PRL	NIH	0.558853333	0.317487429	1.760237671	0.089289088	0.273190987
CXCL1	NIH	-0.77204	0.438824579	-1.759336276	0.089444822	0.273381509
AGT	UCL	0.095871986	0.05487547	1.747082746	0.089466827	0.337870199
CLIP2	NIH	0.490406667	0.278936012	1.758133211	0.089653034	0.27340742
THTPA	NIH	0.34622	0.196992783	1.757526312	0.089758225	0.27340742
PPME1	NIH	0.868733333	0.494334636	1.757379049	0.089783765	0.27340742
AXL	NIH	0.240273333	0.136745041	1.757089921	0.089833927	0.27340742
SEZ6L2	NIH	-0.16676	0.094952157	-1.756252882	0.089979282	0.27340742
IL22RA1	UCL	0.199298959	0.115119875	1.731229809	0.090012204	0.339491189
NFATC3	NIH	0.80232	0.456888429	1.75605235	0.090014135	0.27340742
FCN2	NIH	0.215013333	0.122701315	1.752331128	0.090662973	0.274838537

PMVK	NIH	0.761926667	0.434822019	1.752272499	0.090673227	0.274838537
FAM171B	UCL	-0.207521393	0.118004394	-1.758590392	0.090924187	0.342142469
GADD45B	NIH	-0.497526667	0.284168651	-1.750814751	0.090928505	0.275326995
CFHR5	UCL	-0.292232512	0.166883536	-1.751116494	0.090952188	0.342142469
KEL	UCL	0.273492427	0.156158987	1.751371681	0.091066316	0.342142469
PRC1	UCL	0.693004016	0.395938917	1.750280125	0.091417502	0.342939522
MYL4	UCL	0.39693697	0.226524939	1.752288168	0.091513112	0.342939522
ADD1	NIH	0.505593333	0.289379537	1.747163392	0.091570599	0.276984492
LDLRAP1	UCL	-0.783894789	0.453152471	-1.729869832	0.091704839	0.343087737
ANGPTL2	UCL	0.260714657	0.149233609	1.747023735	0.091787414	0.343087737
ITPA	UCL	-0.443206673	0.255284332	-1.736129555	0.091953376	0.343269118
PON2	NIH	0.347033333	0.198933722	1.744467097	0.092047207	0.277897329
ENO1	NIH	0.227773333	0.130601781	1.744029303	0.092124791	0.277897329
TSNAX	UCL	-0.23953586	0.139793143	-1.713502214	0.092151509	0.343569976
RECK	NIH	0.115293333	0.066132841	1.743359761	0.092243552	0.277897329
APEX1	NIH	0.522773333	0.299874023	1.743309836	0.092252412	0.277897329
ERMAP	UCL	-0.256622491	0.148159131	-1.732073414	0.092411748	0.343877995
IGDCC4	NIH	-0.201146667	0.115451897	-1.742255194	0.092439758	0.278175199
MAP3K5	UCL	-0.825500117	0.475588064	-1.735746077	0.092469416	0.343877995
AGRP	NIH	0.34276	0.196855899	1.741172101	0.092632494	0.278468698
PACS2	UCL	-0.502921704	0.290282904	-1.732522641	0.092697684	0.344288856
RILPL2	NIH	0.345153333	0.198332977	1.740272031	0.092792919	0.278664566
MPRIP	UCL	0.445079952	0.256479875	1.735340648	0.092849043	0.344413393
CRISP2	NIH	0.366853333	0.210900576	1.739461035	0.092937669	0.278813007
ATP6V1D	UCL	-0.140048855	0.080780941	-1.733686845	0.093036856	0.344672661
VASH1	UCL	-0.216923179	0.126340667	-1.716970344	0.093416203	0.34563995
BRK1	NIH	-0.354006667	0.203845042	-1.736645952	0.093441601	0.279836492
BRME1	UCL	-0.302844929	0.174934769	-1.73118775	0.093556405	0.345721076
LRP11	NIH	0.270126667	0.155629118	1.735707751	0.093610062	0.279836492
FAS	NIH	0.242613333	0.13979252	1.735524428	0.093643009	0.279836492
PLA2G2A	NIH	0.32486	0.187193717	1.735421491	0.093661513	0.279836492
IL9	UCL	0.362242125	0.209486522	1.729190601	0.093866806	0.346366821
TSPAN1	NIH	-0.52544	0.302999222	-1.734129863	0.093893963	0.279984462

CEP350	NIH	0.483573333	0.278864074	1.734082581	0.093902481	0.279984462
DNAJC9	UCL	-0.557367195	0.324566184	-1.717268229	0.094015409	0.346366821
CTSC	UCL	-0.39901239	0.229596806	-1.737883014	0.094086643	0.346366821
SPRING1	NIH	0.23502	0.135833318	1.730208782	0.094602615	0.281784775
ANXA5	UCL	-0.245549696	0.144221051	-1.702592613	0.094848882	0.348321722
ST3GAL1	NIH	-0.219146667	0.12675876	-1.728848301	0.094849546	0.282024508
PDGFRA	UCL	0.227606939	0.131780319	1.727169425	0.094859213	0.348321722
CNDP1	NIH	0.293786667	0.169965387	1.728508802	0.094911251	0.282024508
PLA2G10	NIH	-0.494546667	0.286167372	-1.72817279	0.094972356	0.282024508
GFER	UCL	-0.470732065	0.273800586	-1.719251491	0.094975167	0.348321722
USP8	UCL	-0.56362852	0.328021067	-1.718269271	0.095211334	0.348750287
TP53I3	UCL	-0.294415393	0.17175296	-1.714179442	0.096432197	0.352627035
DDC	UCL	0.411538671	0.238388494	1.726336136	0.096510991	0.352627035
DNPEP	NIH	0.39918	0.232233368	1.718874442	0.096676504	0.286793889
DYNLT3	UCL	-0.292205535	0.172992986	-1.689117817	0.09675801	0.352691028
MFAP3L	UCL	-0.429510869	0.248565245	-1.727960271	0.096769827	0.352691028
CD99	UCL	0.131440168	0.076611807	1.715664645	0.097229767	0.353612599
SDHB	NIH	0.270393333	0.15758744	1.715830485	0.097239956	0.288173121
STX8	UCL	-0.256848531	0.150944209	-1.701612357	0.097276876	0.353612599
SEMA4D	UCL	-0.192972604	0.113037688	-1.707152788	0.097503359	0.353612599
CDC26	UCL	-0.346845875	0.204132592	-1.699120515	0.097506587	0.353612599
PALM3	NIH	0.3732	0.217710889	1.714199975	0.097542908	0.288625557
TDP1	NIH	0.370653333	0.216307012	1.713552095	0.097663506	0.288625557
NFKBIE	NIH	0.368826667	0.215257959	1.713417098	0.09768865	0.288625557
INSR	NIH	0.11534	0.067411713	1.710978629	0.098143778	0.289677649
CD2AP	UCL	-0.380720446	0.224515733	-1.695740614	0.098248714	0.355862444
IL12A_IL12B	UCL	-0.36726656	0.214529022	-1.711966783	0.098720143	0.357127447
TMEM106A	NIH	0.394313333	0.230904444	1.707690532	0.09876031	0.291177482
GTF2IRD1	NIH	0.425753333	0.249385712	1.707208202	0.098851022	0.291177482
CCAR2	NIH	0.4187	0.245409027	1.706131211	0.099053826	0.291293607
PRND	NIH	0.417726667	0.244906665	1.705656592	0.099143311	0.291293607
FUS	NIH	0.40216	0.235813805	1.7054133	0.099189208	0.291293607
CCL5	UCL	-0.833517362	0.491664865	-1.695295763	0.099262242	0.358644666

CA2	UCL	-0.514635617	0.303628346	-1.694952479	0.099465502	0.358935384
SUGP1	NIH	0.22076	0.129839657	1.700250951	0.100167298	0.293518279
PI16	NIH	0.202886667	0.119376869	1.699547557	0.100301193	0.293518279
BNIP3L	NIH	0.26232	0.154366631	1.699330995	0.100342447	0.293518279
CNTNAP4	NIH	1.048486667	0.617010502	1.699301169	0.100348129	0.293518279
FKBP14	NIH	0.207466667	0.122191378	1.697883031	0.100618643	0.294015515
ACYP1	NIH	0.427626667	0.25205464	1.696563356	0.100870925	0.294363569
FETUB	NIH	0.207526667	0.122347447	1.696207581	0.100939029	0.294363569
IL13	NIH	1.199486667	0.708028268	1.694122568	0.101338929	0.295235425
LTBP3	UCL	-0.329698761	0.194037513	-1.699149591	0.101447542	0.365636456
TIGAR	NIH	0.42248	0.249548971	1.692974325	0.101559726	0.295584277
CLTA	UCL	-0.195827027	0.116159134	-1.685851296	0.101627501	0.365833974
RASSF2	UCL	-0.338632337	0.201066352	-1.684182025	0.101852442	0.366192729
TCL1B	NIH	0.45982	0.271928232	1.690960872	0.10194787	0.296263626
CD58	NIH	-0.113986667	0.067419283	-1.690713122	0.101995717	0.296263626
BLVRB	NIH	0.569366667	0.336908268	1.689975348	0.102138309	0.296383485
NIT1	NIH	0.36196	0.214604634	1.686636459	0.10278572	0.297966532
TXLNA	NIH	0.411553333	0.244233217	1.685083371	0.103088034	0.298122542
BCL2L1	NIH	0.382873333	0.227228548	1.684970208	0.103110091	0.298122542
TPPP3	UCL	0.853107703	0.507114068	1.6822797	0.103176295	0.370496695
RAD23B	NIH	0.177353333	0.105291144	1.684408829	0.103219568	0.298122542
IL10	NIH	0.48314	0.286896185	1.68402379	0.103294713	0.298122542
SAFB2	NIH	0.240673333	0.1429393	1.683745015	0.103349148	0.298122542
ENG	NIH	-0.130613333	0.07760902	-1.682965891	0.10350141	0.29826761
SCPEP1	UCL	-0.350449777	0.209896326	-1.66963274	0.103603426	0.371553256
PADI2	UCL	0.411451208	0.245193142	1.678069804	0.103783424	0.371553256
GSN	UCL	0.161404574	0.096823501	1.666997904	0.103851868	0.371553256
APOBR	UCL	0.315197368	0.1869707	1.685811567	0.103989165	0.371589645
GP1BB	UCL	-0.479171104	0.289108905	-1.657406933	0.104234586	0.372011836
PDCD5	UCL	-0.315142812	0.189262542	-1.665109259	0.104418174	0.372212588
GDNF	NIH	-0.28256	0.168383026	-1.678078883	0.104460758	0.300735943
CSF3	UCL	-0.551403386	0.327526103	-1.683540278	0.104580982	0.372338869
TREM2	NIH	0.43756	0.260971281	1.676659586	0.104740766	0.300989487

SERPINB8	NIH	0.360833333	0.215218668	1.676589384	0.104754632	0.300989487
CD300LF	UCL	0.367270839	0.21835954	1.681954628	0.104851443	0.372847651
CCL18	UCL	-0.302309092	0.181303508	-1.667419981	0.105155871	0.37312626
LIF	NIH	0.387026667	0.231136403	1.674451372	0.105177664	0.301356041
PECAM1	UCL	0.150711723	0.090240417	1.670113331	0.105185097	0.37312626
UNG	NIH	0.2794	0.166890254	1.674154083	0.105236599	0.301356041
PRTN3	NIH	0.5765	0.344381272	1.674016699	0.105263844	0.301356041
DGKA	NIH	0.318846667	0.19051335	1.673618502	0.105342844	0.301356041
GHRHR	UCL	0.742355343	0.444134104	1.671466652	0.105391104	0.373403877
SCARA5	NIH	0.178373333	0.106627871	1.672858433	0.105493775	0.301356041
SRP14	UCL	-0.287308369	0.17270926	-1.663537721	0.105564219	0.373564423
ZPR1	NIH	0.89468	0.535031797	1.672199681	0.105624733	0.301356041
RPS10	NIH	0.325033333	0.194401306	1.671970932	0.105670239	0.301356041
PRSS53	NIH	0.35322	0.211327106	1.671437268	0.105776468	0.301356041
CDH1	NIH	0.178873333	0.107028282	1.671271646	0.105809454	0.301356041
MSLN	UCL	-0.506181466	0.301848149	-1.676940763	0.1058514	0.374127743
CWC15	NIH	0.526333333	0.31523829	1.669636429	0.106135595	0.301715251
CLC	NIH	0.444873333	0.266457908	1.669582025	0.10614646	0.301715251
BTLA	NIH	0.987406667	0.591584303	1.669088685	0.106245028	0.301715251
YOD1	NIH	0.704346667	0.422185091	1.668336191	0.106395523	0.301849569
ITGA6	UCL	-0.163609092	0.099711748	-1.640820622	0.106440138	0.375754255
FUCA1	UCL	-0.615897188	0.36870068	-1.670453081	0.106804655	0.376465906
PSPN	NIH	0.48788	0.292795166	1.666284339	0.106806788	0.302722728
CPE	UCL	-0.292084729	0.177341403	-1.647019384	0.107021861	0.376465906
DNAJB2	UCL	-0.293195268	0.177941182	-1.647708898	0.107028111	0.376465906
NUDC	NIH	0.292986667	0.176031992	1.664394427	0.107186768	0.303505612
LSP1	UCL	-0.269039865	0.161987335	-1.660869751	0.107500298	0.377672321
IDUA	NIH	0.305733333	0.184132366	1.660399743	0.107993644	0.305494591
GGT	NIH	-0.383456872	0.230666158	-1.662388948	0.108002777	0.309376375
VIM	UCL	-0.942907641	0.575933872	-1.637180389	0.108049782	0.379147075
DPT	UCL	0.157652234	0.094887593	1.661463102	0.108208702	0.379249444
SYAP1	UCL	-0.266325758	0.163372036	-1.630179583	0.108676096	0.38043141
PBXIP1	NIH	-0.166006667	0.100186394	-1.656978165	0.108688782	0.307113628

GLO1	NIH	0.42072	0.253973575	1.656550292	0.108775972	0.307113628
HMBS	NIH	0.677273333	0.408997953	1.65593331	0.1089018	0.307172388
SHH	UCL	-0.583192445	0.343937584	-1.695634533	0.10906828	0.380524265
EDIL3	UCL	0.310168839	0.187668578	1.652747848	0.109074922	0.380524265
PRKD2	UCL	-0.353028717	0.211909807	-1.665938548	0.109093169	0.380524265
CHRDL1	UCL	0.241534424	0.145774946	1.656899426	0.109338863	0.380926696
CCT5	NIH	0.225726667	0.136497885	1.653700837	0.109358109	0.307829147
NSFL1C	NIH	0.478133333	0.289196865	1.653314374	0.109437262	0.307829147
PNLIPRP2	NIH	1.864746667	1.127927525	1.653250431	0.109450363	0.307829147
ULBP2	UCL	-0.463518012	0.279883157	-1.656112563	0.109475421	0.380948398
CR2	NIH	0.34598	0.209390649	1.652318297	0.109641493	0.308070478
CHGB	NIH	-0.312993333	0.189535784	-1.651368028	0.109836628	0.308322587
BOC	UCL	0.16284744	0.09882339	1.647863325	0.11014114	0.382809219
CD2	NIH	0.209126667	0.126907204	1.647870733	0.110557278	0.310039673
CEACAM3	UCL	-0.593109023	0.358009971	-1.656682975	0.110594883	0.383886812
OMG	NIH	-0.476886667	0.289483245	-1.647372255	0.110660314	0.310039673
CACNB1	UCL	0.620473818	0.37784701	1.642129755	0.11071385	0.383886812
TYMP	NIH	-0.28064	0.170455413	-1.646412951	0.110858828	0.310298634
EGFLAM	UCL	-0.187540287	0.114322234	-1.640453306	0.110865319	0.383956549
GSR	UCL	0.15489071	0.094202447	1.644232337	0.111132044	0.384424811
ATF2	UCL	0.457409711	0.278393714	1.643031747	0.111384703	0.384843366
ENPEP	NIH	0.37714	0.229456828	1.643620734	0.111438324	0.311622466
RCC1	UCL	0.240645964	0.146846292	1.638760914	0.111579957	0.385062828
CLEC3B	UCL	0.128340915	0.07807402	1.643836395	0.111722406	0.385099756
OSCAR	NIH	0.2472	0.150541837	1.642068443	0.111761575	0.312227895
SCP2	UCL	-0.266143083	0.164867686	-1.614282876	0.112087339	0.385873432
PNLIPRP1	UCL	-0.485125078	0.29553134	-1.641535133	0.112210885	0.385873432
CLEC4D	NIH	0.4699	0.286710864	1.638933361	0.112416805	0.313758736
TXNRD1	UCL	-0.217454251	0.133247317	-1.631959692	0.112444391	0.386222038
IRAK4	NIH	0.433713333	0.265081477	1.636151039	0.11300098	0.315088528
SMS	UCL	-0.276459927	0.16990529	-1.627141375	0.113148014	0.387790955
CD3E	NIH	0.195713333	0.119673008	1.635400809	0.113158928	0.315228444
PCBD1	UCL	0.372675343	0.226912116	1.642377451	0.113166502	0.387790955

NFE2	NIH	0.368366667	0.22532261	1.634841116	0.113276882	0.315256785
LEPR	UCL	0.160230761	0.09827993	1.630350782	0.113906305	0.389068106
CXADR	NIH	0.3238	0.198428828	1.631819348	0.113915476	0.316732669
IDUA	UCL	0.297612708	0.182002144	1.635215395	0.113934266	0.389068106
TALDO1	UCL	-0.219798708	0.135087228	-1.627087267	0.113963195	0.389068106
L3HYPDH	UCL	-0.504143186	0.312656442	-1.612450979	0.114071627	0.389068106
RAB6B	UCL	-0.177775705	0.110126728	-1.614283008	0.11422436	0.389134971
MSRA	NIH	0.329093333	0.201904047	1.629949171	0.114312198	0.317354386
NUB1	NIH	0.335406667	0.205803454	1.629742655	0.114356076	0.317354386
PTPRS	UCL	0.181822675	0.111441975	1.631545706	0.11447187	0.389524188
KLK3	NIH	-0.702033333	0.430984086	-1.628907785	0.114533604	0.317545774
GJA8	NIH	0.483953333	0.297333845	1.627642938	0.114802998	0.317991257
KIR2DL3	UCL	-0.605427697	0.37178194	-1.628448378	0.114982047	0.390805259
HGFAC	UCL	0.190560149	0.117059635	1.627889486	0.115341274	0.391570897
CCND2	NIH	-0.325986667	0.200605502	-1.625013591	0.115364692	0.319244771
DDX53	UCL	0.184877063	0.115653921	1.598536927	0.115551213	0.391828534
SEC31A	NIH	0.205693333	0.126661012	1.623967232	0.115588853	0.319562755
APOBR	NIH	-0.295053333	0.181808231	-1.622882156	0.115821689	0.319904099
RRM2B	NIH	0.316253333	0.194952459	1.622207461	0.115966662	0.320002345
RPE	UCL	-0.3819583	0.237667649	-1.607111032	0.116307891	0.39314064
MET	UCL	0.12785914	0.078888384	1.620760025	0.116354965	0.39314064
PAK4	UCL	-0.512235398	0.320452571	-1.598474922	0.11639122	0.39314064
CELA2A	UCL	-0.392976228	0.24234003	-1.621590243	0.116476153	0.39314064
SCRIB	UCL	-0.538070815	0.332391939	-1.61878419	0.116634396	0.39322069
CCL2	NIH	0.34574	0.213681036	1.618019112	0.116869984	0.322191049
SPINT3	UCL	-1.101048813	0.678065503	-1.623808921	0.117103834	0.394229728
IMPG1	UCL	0.14120118	0.088472544	1.595988692	0.117203433	0.394229728
THPO	UCL	-0.32602596	0.201407163	-1.618740644	0.117697764	0.395270293
PODXL	UCL	0.100433826	0.062249533	1.613406905	0.117783245	0.395270293
IRAK1	NIH	0.328273333	0.203438161	1.613627115	0.117823476	0.324513811
OSTN	NIH	-0.441873333	0.274029485	-1.612502881	0.118068576	0.324584702
IFNL1	NIH	0.648713333	0.40230507	1.61249107	0.118071153	0.324584702
VASP	NIH	0.194033333	0.120500615	1.61022692	0.118566061	0.325019021

SERPINB6	NIH	0.169186667	0.105092139	1.609888889	0.118640096	0.325019021
EGFR	NIH	-0.116853333	0.072609032	-1.609349836	0.118758238	0.325019021
EFCAB2	UCL	-0.206962348	0.129834733	-1.594044547	0.118848741	0.397453277
ERVV-1	NIH	0.322206667	0.200270684	1.608855876	0.118866582	0.325019021
RETN	NIH	0.323206667	0.200960536	1.608309136	0.118986599	0.325019021
NCK2	NIH	0.15986	0.099405751	1.608156459	0.119020131	0.325019021
CPTP	UCL	-0.277998528	0.17120128	-1.623811035	0.119042755	0.397453277
CCNE1	UCL	0.73358389	0.455656372	1.609949811	0.119058476	0.397453277
DTX2	NIH	0.91902	0.571542568	1.607964221	0.119062363	0.325019021
LHPP	UCL	-0.346632083	0.216310745	-1.602472787	0.119097783	0.397453277
PON1	UCL	0.175889716	0.109460215	1.606882605	0.119113606	0.397453277
CSNK1D	NIH	0.317233333	0.197375963	1.607254136	0.119218467	0.325019021
UGDH	NIH	0.930686667	0.579071411	1.607205344	0.1192292	0.325019021
FAM20A	NIH	0.18162	0.113128638	1.605429029	0.119620472	0.325601472
MAPT	NIH	1.56442	0.974579934	1.605224923	0.119665498	0.325601472
TNFSF9	NIH	0.36408	0.226910102	1.604512078	0.119822865	0.325726654
PLA2G4A	NIH	0.194593333	0.121323869	1.603916312	0.119954516	0.325781764
GFOD2	UCL	0.651262129	0.406299555	1.602911252	0.12035634	0.401142054
IL12B	UCL	-0.256479189	0.16002934	-1.602701033	0.120708353	0.40185708
LAIR1	NIH	0.237686667	0.148650343	1.598964805	0.121053317	0.328181124
PECAM1	NIH	-0.14724	0.092119504	-1.598358587	0.121188413	0.328181124
RALY	NIH	0.42254	0.264371554	1.598280881	0.121205739	0.328181124
FOXO1	NIH	0.46064	0.288275192	1.597917589	0.121286767	0.328181124
PODXL2	NIH	0.21872	0.137036837	1.59606719	0.121700175	0.32875431
LRRC59	UCL	-0.311400976	0.197042314	-1.580376164	0.121741594	0.404640706
SPINK1	UCL	-0.341023131	0.213245912	-1.599201256	0.121821355	0.404640706
PDGFRB	NIH	0.19654	0.123187869	1.595449299	0.121838479	0.32875431
ANGPT1	NIH	0.208093333	0.130436934	1.595355913	0.121859394	0.32875431
FKBP7	NIH	-0.34498	0.216293878	-1.594959611	0.12194818	0.32875431
TLR4	NIH	0.34774	0.218098237	1.594419125	0.122069355	0.328777959
LRFN2	UCL	-0.549728081	0.343624597	-1.599792581	0.122371986	0.40591161
CD209	NIH	0.281746667	0.176901896	1.592671832	0.12246177	0.329531443
ARL13B	UCL	-0.653494626	0.412585835	-1.583899813	0.122481711	0.40591161

ACP6	NIH	0.330826667	0.207928174	1.591062245	0.122824178	0.329992829
RAB6A	UCL	0.22731091	0.143556407	1.583425739	0.122825129	0.406513215
UROD	NIH	0.59282	0.372707077	1.590578865	0.122933186	0.329992829
CNGB3	UCL	-0.585757417	0.366081281	-1.600074759	0.12294139	0.406513215
RASSF2	NIH	0.555626667	0.349361038	1.590408222	0.122971687	0.329992829
THSD1	UCL	0.229405977	0.145999232	1.571282081	0.123111925	0.406617127
KRT19	UCL	-0.357504259	0.225558773	-1.584971642	0.12336554	0.406994891
PTH	NIH	0.546473333	0.344164842	1.587824399	0.123555872	0.331256577
DNM3	UCL	-0.350251955	0.220809339	-1.586218938	0.123627491	0.407399274
LAG3	NIH	0.29816	0.187980188	1.586124602	0.123941429	0.331985969
CDON	UCL	0.220083064	0.139048563	1.582778417	0.124412603	0.40936533
STAT2	UCL	-0.435590484	0.277358836	-1.570494345	0.124504201	0.40936533
AHSA1	NIH	0.331266667	0.209275421	1.582921996	0.124670549	0.333633444
ACTN4	UCL	-0.13546415	0.086129131	-1.572802933	0.124848742	0.409481731
HSBP1	UCL	-0.504598972	0.320564143	-1.57409674	0.124849101	0.409481731
SMARCA2	UCL	-0.460076527	0.29347281	-1.567697283	0.124959871	0.409481731
TXNRD1	NIH	0.123253333	0.077946344	1.581258686	0.125050614	0.33434465
ATP5IF1	UCL	-0.668991916	0.427118794	-1.566290045	0.125107839	0.409507518
BTN1A1	UCL	0.233617497	0.14869791	1.571087968	0.12538526	0.409676882
TBC1D17	UCL	-0.246762313	0.157413658	-1.567604208	0.125439894	0.409676882
LMNB1	NIH	0.668106667	0.423312338	1.578283	0.125732933	0.335861945
CLEC4D	UCL	-0.383032051	0.242590058	-1.578927239	0.125791855	0.410121295
NCR1	NIH	0.272466667	0.172739547	1.57732651	0.125952902	0.336142555
PSMD1	UCL	0.340787888	0.216375773	1.574981723	0.125997983	0.410121295
TARM1	UCL	0.94139708	0.596325419	1.578663346	0.126088447	0.410121295
AGBL2	UCL	0.647318615	0.410572451	1.576624572	0.126137203	0.410121295
TMPRSS11D	NIH	-0.20238	0.128441297	-1.575661446	0.126336581	0.336579522
IQGAP2	NIH	0.339693333	0.215593813	1.575617264	0.126346774	0.336579522
FH	UCL	0.315734285	0.201969044	1.563280582	0.12654278	0.410982829
GLA	NIH	0.23692	0.150582127	1.573360695	0.126868317	0.337661354
CD300LG	UCL	0.161001536	0.102246415	1.574642354	0.126879693	0.411619692
TADA3	NIH	0.27856	0.177147838	1.57247191	0.127074219	0.337901901
SIGLEC5	UCL	-1.057991917	0.671909779	-1.574604135	0.127123536	0.411953542

TCL1B	UCL	0.613881052	0.390579681	1.571717838	0.127265995	0.411958476
CRIP2	UCL	-0.201485296	0.128514437	-1.567802808	0.127580007	0.412290926
FMNL1	NIH	0.506973333	0.322956228	1.569789616	0.12769728	0.338978416
NCAM2	NIH	-0.194506667	0.1239108	-1.569731351	0.127710842	0.338978416
DCXR	UCL	0.369555617	0.233577192	1.582156262	0.127742641	0.412290926
GNPDA1	NIH	0.1954	0.124567987	1.568621321	0.127969442	0.339134386
CPM	UCL	-0.230413627	0.146544386	-1.572312891	0.12797875	0.412290926
DBI	NIH	0.32732	0.208685601	1.568483877	0.128001491	0.339134386
MPIG6B	UCL	-0.632513191	0.407587625	-1.551845915	0.128018964	0.412290926
CCN3	UCL	0.233310715	0.148576213	1.570310013	0.128073952	0.412290926
AKT1S1	NIH	0.370773333	0.236570219	1.567286597	0.128280955	0.339567234
CHRDL2	UCL	-0.387118772	0.246472156	-1.570638969	0.128433786	0.412994453
SLIT2	NIH	0.32632	0.208440207	1.565532894	0.128691199	0.340335945
ITGA6	NIH	0.208486667	0.133213957	1.565051221	0.128804065	0.340335945
GOLGA3	NIH	0.363486667	0.232422613	1.563904054	0.129073196	0.340469843
DNAJC21	NIH	0.14036	0.089753257	1.563842967	0.129087541	0.340469843
PTPRK	NIH	0.154993333	0.099153821	1.563160475	0.12924789	0.340585655
HIF1A	UCL	-0.361720477	0.23076507	-1.567483661	0.129271741	0.414814723
VTCN1	UCL	-0.237175686	0.153757215	-1.542533701	0.129283686	0.414814723
AMY2B	NIH	-0.372926667	0.238789386	-1.561738875	0.129582413	0.341159818
OCLN	UCL	-0.259282194	0.167765391	-1.545504665	0.129905339	0.41548372
FGFBP2	UCL	0.391818132	0.250687838	1.56297224	0.130043539	0.41548372
GGT1	UCL	-0.386778923	0.24772478	-1.561325126	0.130074434	0.41548372
BTNL10	UCL	-0.572985739	0.365730317	-1.566689203	0.130206966	0.41548372
CDC25A	UCL	-0.285542229	0.184138834	-1.550689895	0.130228473	0.41548372
CD48	NIH	0.162826667	0.104443903	1.558986799	0.130232031	0.34245172
NECAP2	NIH	0.29812	0.19126578	1.558668777	0.13030727	0.34245172
NRP2	UCL	0.241167911	0.154607005	1.559876997	0.130568377	0.41548372
IKZF2	UCL	1.476335856	0.947809907	1.557628641	0.130616931	0.41548372
PRSS2	UCL	-0.38743537	0.248509256	-1.559037982	0.130629333	0.41548372
AMIGO2	NIH	0.106333333	0.068297248	1.55691973	0.130721705	0.343232483
CASP10	UCL	-1.104339929	0.720211323	-1.53335541	0.1308189	0.415634398
TMED10	UCL	0.37944212	0.244426956	1.552374281	0.131181139	0.416332757

TNFAIP8L2	NIH	0.244366667	0.157196854	1.554526448	0.131290536	0.344330188
SART1	NIH	0.51156	0.329152987	1.554170917	0.13137521	0.344330188
LPO	UCL	0.352406894	0.226866262	1.553368455	0.131404359	0.416588875
IL12RB1	NIH	0.223173333	0.143647525	1.55361767	0.131507062	0.344367194
NXPH3	UCL	0.207500801	0.133778547	1.551076804	0.131673488	0.41689428
CD164L2	NIH	0.40124	0.258472099	1.55235324	0.131808811	0.344460305
GIGYF2	NIH	0.331693333	0.213716888	1.552022097	0.13188793	0.344460305
LGALS1	NIH	0.240313333	0.154842193	1.5519887	0.131895912	0.344460305
CCL26	UCL	0.666406509	0.429785445	1.550556254	0.131909226	0.41689428
PRAP1	UCL	-0.258709241	0.166876817	-1.550300666	0.13192857	0.41689428
CD6	NIH	0.33012	0.21278308	1.551439144	0.132027307	0.344495873
FRMD7	NIH	0.456913333	0.294946469	1.549139865	0.132578211	0.344757987
THBS4	NIH	0.33196	0.214292238	1.549099505	0.132587898	0.344757987
TARBP2	NIH	0.2947	0.19025587	1.548966663	0.132619786	0.344757987
CA4	UCL	-0.152451321	0.098292202	-1.551001172	0.132662566	0.418706954
CDC42BPB	NIH	0.344466667	0.222439652	1.548584809	0.132711482	0.344757987
PSAP	NIH	0.178826667	0.115479218	1.548561457	0.132717092	0.344757987
IL7	UCL	-0.419948194	0.273053116	-1.53797254	0.132788692	0.418706954
CEBPB	UCL	-0.167522358	0.108757346	-1.540331418	0.133273715	0.419783478
PON2	UCL	-0.340094948	0.219623313	-1.548537553	0.13342107	0.419795251
RLN1	NIH	0.786206667	0.508891881	1.544938515	0.133589694	0.34669963
SMC3	NIH	0.265333333	0.17179513	1.544475292	0.133701601	0.34669963
ALDH3A1	UCL	-0.477417976	0.309458649	-1.542752086	0.133828817	0.420334551
LIF	UCL	-0.602987535	0.3927056	-1.535469663	0.133880078	0.420334551
MMP1	UCL	-0.629814685	0.409536799	-1.537870798	0.134076294	0.420498936
KCNH2	NIH	0.430973333	0.279363902	1.542695137	0.134132365	0.347508563
CASP3	NIH	0.270266667	0.175421607	1.540669202	0.134623979	0.348473574
PTGDS	UCL	-0.171388452	0.111251769	-1.540545861	0.134648132	0.421839755
CALY	UCL	-0.328058596	0.21184263	-1.548595746	0.135093612	0.422782258
CCN1	NIH	-0.269326667	0.175123713	-1.53792232	0.135292882	0.349617493
GBP1	NIH	0.329246667	0.214148693	1.53746755	0.135403886	0.349617493
GMFG	NIH	0.372186667	0.242091022	1.537383189	0.135424485	0.349617493
HARS1	NIH	0.237493333	0.154662327	1.53556033	0.135870221	0.350257657

TPP1	NIH	0.198306667	0.1291572	1.535389945	0.135911946	0.350257657
MAVS	NIH	0.344473333	0.224431797	1.53486867	0.136039662	0.350278179
HLA-E	UCL	-0.13688281	0.089662582	-1.526643643	0.136350542	0.426259501
AMOTL2	UCL	-0.128204583	0.084130557	-1.523876553	0.136566668	0.426444528
TRIM40	UCL	-0.365501033	0.241009757	-1.516540395	0.136701513	0.426444528
PDCD6	NIH	0.26362	0.172086057	1.531907961	0.136766913	0.351574275
OPHN1	NIH	0.515413333	0.336466627	1.531840877	0.136783427	0.351574275
ELOB	NIH	0.177306667	0.1157968	1.531187964	0.136944245	0.35167746
CCL23	UCL	-0.210369348	0.137514868	-1.529793476	0.137093222	0.427210541
VEGFC	NIH	0.132986667	0.086909258	1.530178374	0.137193219	0.35167746
HLA-DRA	NIH	0.437806667	0.286186632	1.52979426	0.137288041	0.35167746
COMT	NIH	0.333993333	0.218488416	1.528654649	0.137569679	0.35167746
C9orf40	NIH	0.88906	0.5816179	1.528598072	0.137583674	0.35167746
ANKRA2	UCL	-0.37111639	0.23604113	-1.572253068	0.137641906	0.428463569
ACRBP	NIH	0.178853333	0.117022933	1.528361402	0.137642227	0.35167746
GLP1R	NIH	0.425986667	0.278738089	1.528268592	0.137665194	0.35167746
IL25	NIH	-0.193353333	0.126559893	-1.527761514	0.137790732	0.351691005
ACOX1	UCL	-0.202956614	0.133623995	-1.518863544	0.137880145	0.428748578
EGLN1	NIH	0.42256	0.276938976	1.525823506	0.138271387	0.35208992
SLIRP	NIH	0.33342	0.218545787	1.525629956	0.138319465	0.35208992
CHCHD6	UCL	0.158193377	0.104167922	1.518638119	0.138449931	0.430062857
GNE	NIH	0.382026667	0.250508591	1.525004253	0.138474983	0.35208992
MN1	NIH	0.4086	0.267996029	1.524649459	0.138563229	0.35208992
EDF1	NIH	0.3816	0.250341212	1.524319533	0.138645332	0.35208992
TIMP4	NIH	0.277546667	0.182090542	1.524223406	0.138669261	0.35208992
HNMT	NIH	0.328646667	0.215739638	1.523348561	0.138887189	0.352337404
BCAT1	UCL	-0.240405471	0.157761979	-1.523849231	0.139293681	0.432224446
IKBKG	NIH	0.29842	0.19622808	1.520781324	0.139528305	0.353657098
ENTPD5	UCL	0.163887132	0.107621359	1.522812332	0.139560072	0.43230514
AHSA1	UCL	-0.327577168	0.215970568	-1.51676764	0.139615481	0.43230514
TNFRSF6B	NIH	0.312946667	0.205870272	1.520115863	0.139694882	0.35373645
WASL	NIH	0.420333333	0.276591436	1.519690341	0.139801482	0.35373645
VGF	NIH	-0.16098	0.105969051	-1.519122782	0.139943768	0.353790424

ZNF830	UCL	-0.501698596	0.329107401	-1.5244221	0.140030172	0.433130363
TSNAX	NIH	0.393453333	0.25913687	1.518322472	0.140144603	0.353992198
C1QTNF1	NIH	-0.256726667	0.169205072	-1.5172516	0.140413701	0.354365898
ADA	UCL	-0.210628236	0.138593181	-1.519759014	0.140447133	0.433960855
ITGB7	NIH	-0.161253333	0.106315325	-1.516745895	0.140540924	0.35438121
CA14	UCL	-0.207292658	0.137121891	-1.511740071	0.140641966	0.434103976
NMRK2	NIH	-0.190026667	0.125423679	-1.515078075	0.140961171	0.355124721
ZCCHC8	NIH	0.315393333	0.208233648	1.514612727	0.141078607	0.355124721
S100A12	NIH	0.450286667	0.297415935	1.513996439	0.141234258	0.355210838
CALB1	NIH	-0.257366667	0.170266767	-1.511549616	0.141853604	0.356349298
ATXN2	NIH	0.968973333	0.641175061	1.511246135	0.141930575	0.356349298
PRDX6	UCL	-0.279945093	0.186322892	-1.502472883	0.14210073	0.438143917
C19orf12	NIH	0.480933333	0.318410006	1.510421545	0.142139884	0.356470935
FABP1	NIH	0.514853333	0.340940952	1.51009531	0.142222763	0.356470935
CFHR4	UCL	-0.480232828	0.317783974	-1.511192718	0.142309626	0.438219303
CBLIF	UCL	0.439986822	0.292005748	1.506774527	0.142425022	0.438219303
CD2AP	NIH	0.536026667	0.355153821	1.509280301	0.142429983	0.356684675
MEP1A	UCL	-0.460584317	0.30544945	-1.507890475	0.143084623	0.43978586
CEP290	NIH	0.481993333	0.320226501	1.505163788	0.143480364	0.359007755
PDXDC1	UCL	-0.181355744	0.121643291	-1.490881602	0.143533048	0.440700734
LETM1	NIH	0.23998	0.159614131	1.503500963	0.143906428	0.35976607
RPL14	UCL	-0.28668272	0.190409136	-1.505614315	0.143964653	0.441562099
GAST	UCL	0.894203413	0.597550549	1.496448149	0.144205252	0.441836428
DDI2	NIH	0.680633333	0.453087698	1.502211021	0.144237652	0.360286193
SEZ6L	NIH	-0.14112	0.093975712	-1.501664598	0.144378145	0.360329415
SLC44A4	NIH	0.394213333	0.262610525	1.50113303	0.144514925	0.360363303
CTRB1	NIH	-0.41922	0.279483312	-1.499982227	0.144811401	0.360795014
CRELD2	NIH	0.316633333	0.211309655	1.498432869	0.14521133	0.361263266
CSF1R	NIH	0.2437	0.162659725	1.498219673	0.145266431	0.361263266
BLOC1S3	NIH	0.149486667	0.099802849	1.497819634	0.145369868	0.361263266
CA1	NIH	0.724893333	0.484781976	1.495297617	0.146023347	0.362579194
PI3	UCL	-0.444273709	0.297044361	-1.495647678	0.146059562	0.44704932
IL11	NIH	-0.56584	0.378682738	-1.494232358	0.146300078	0.362958208

CRELD2	UCL	-0.415962517	0.277784787	-1.497427274	0.146311527	0.4472539
FIS1	UCL	0.395358888	0.266492753	1.483563372	0.146432426	0.4472539
ARHGEF10	NIH	0.4302	0.288125296	1.49310042	0.146594593	0.363301648
LPP	NIH	-0.26326	0.176359548	-1.492745941	0.146686922	0.363301648
NAMPT	NIH	0.320933333	0.215142004	1.491727916	0.146952341	0.363651098
DSC2	NIH	0.214006667	0.143617165	1.490119	0.147372607	0.36438282
ATP5F1D	UCL	0.213620287	0.144967559	1.473573039	0.147601059	0.450352711
KIR3DL2	UCL	0.602665887	0.405543759	1.486068702	0.147850851	0.450388639
VGF	UCL	-0.167992786	0.113670553	-1.477891869	0.148017473	0.450388639
MPI	UCL	-0.372320559	0.251675678	-1.47936647	0.14822715	0.450388639
TMSB10	UCL	-0.448833278	0.302341728	-1.484523097	0.148229172	0.450388639
PILRA	NIH	0.228826667	0.153955616	1.486315818	0.148369889	0.366538789
MMP10	UCL	0.332548261	0.22330229	1.489229064	0.148438443	0.450556147
VIT	UCL	0.273816741	0.183950716	1.488533164	0.148721712	0.450651081
FLT3	NIH	-0.2138	0.1439875	-1.484851113	0.148755415	0.366656911
RRM2B	UCL	-0.514835319	0.347963921	-1.479565233	0.148778068	0.450651081
PZP	NIH	0.25124	0.169227588	1.484627912	0.148814235	0.366656911
WIF1	NIH	-0.19992	0.134662428	-1.484601182	0.14882128	0.366656911
ASAH2	NIH	-0.66476	0.447882036	-1.484230102	0.148919115	0.366656911
NLGN1	UCL	0.670519847	0.451780033	1.484173266	0.148990863	0.45082846
PPIE	NIH	0.326873333	0.220509819	1.482352734	0.149414875	0.367568132
PRTN3	UCL	0.233658318	0.157498744	1.483556705	0.149504753	0.451398357
ZP3	UCL	-1.723139533	1.161804999	-1.483157272	0.149631604	0.451398357
TNR	UCL	0.237447897	0.159990166	1.484140571	0.149659366	0.451398357
NOTCH1	UCL	0.079066456	0.053531817	1.476999295	0.149796923	0.451398357
VCPKMT	NIH	-0.608273333	0.411135555	-1.479495816	0.150171853	0.368848065
SERPINC1	NIH	0.094993333	0.064209093	1.479437389	0.150187366	0.368848065
DDX1	UCL	-0.250630153	0.171809047	-1.458771567	0.150214448	0.451807363
SOX9	UCL	-0.210454957	0.143849191	-1.463024962	0.150241792	0.451807363
ALDH5A1	NIH	0.313306667	0.212000667	1.477856982	0.150607473	0.369354715
PACS2	NIH	0.248013333	0.167836108	1.477711421	0.150646214	0.369354715
ICAM3	NIH	-0.163526667	0.110884779	-1.474744038	0.151437724	0.370932822
LILRA6	NIH	0.549526667	0.372930206	1.473537562	0.151760489	0.370932822

HS6ST1	UCL	0.189625678	0.128892905	1.471187869	0.151761308	0.455380995
GLB1	UCL	-0.284488956	0.193471521	-1.470443581	0.151766767	0.455380995
ERP29	NIH	0.412793333	0.280145283	1.473497353	0.151771255	0.370932822
IL10RA	NIH	0.33862	0.229822063	1.473400751	0.151797124	0.370932822
HK2	UCL	0.135378603	0.092910888	1.457080069	0.152038353	0.455380995
CHGA	UCL	0.731987377	0.497607999	1.471012078	0.152053319	0.455380995
GTF2IRD1	UCL	-0.459901794	0.311898605	-1.474523408	0.152782197	0.45663542
IL1R1	UCL	0.147734319	0.10042565	1.471081532	0.152784619	0.45663542
RAD51	UCL	0.499491713	0.340587336	1.466559852	0.153449229	0.457951378
FGF5	UCL	0.265988764	0.181293288	1.467173806	0.153538266	0.457951378
CXCL12	NIH	0.306126667	0.208698023	1.466840283	0.153562244	0.374932858
SHC1	UCL	-0.577404609	0.397390472	-1.452990572	0.153715517	0.458012698
SOD2	UCL	-0.245120999	0.166719552	-1.470259468	0.153878858	0.458032487
TG	UCL	0.51359286	0.350516537	1.465245733	0.154090422	0.45819563
PCDH17	NIH	0.26976	0.184160274	1.464811028	0.154111542	0.375766604
PBK	NIH	0.28984	0.197937432	1.4643011	0.154249822	0.375766604
NT5C	NIH	0.38494	0.262952674	1.463913617	0.154354964	0.375766604
PPP1R12A	NIH	0.364353333	0.248981148	1.463377194	0.154500615	0.375766604
HSPB1	NIH	0.4177	0.285468269	1.463209907	0.15454606	0.375766604
ANGPTL4	UCL	-0.322644976	0.222403945	-1.450716065	0.154619723	0.459302288
C1QA	UCL	0.160886218	0.109800994	1.465252837	0.154783115	0.459320857
DNAJB14	NIH	0.302786667	0.207151998	1.461664235	0.154966459	0.376167509
PSTPIP2	NIH	0.344153333	0.235516541	1.46127033	0.155073741	0.376167509
ASS1	UCL	0.403966424	0.278877091	1.448546466	0.155188704	0.460057384
VWC2	NIH	0.22422	0.153553341	1.460209189	0.155363045	0.376167509
SCN3A	NIH	0.54424	0.372772485	1.459979002	0.155425859	0.376167509
SMAD3	NIH	0.178226667	0.122113295	1.459518939	0.155551463	0.376167509
GGCT	NIH	0.285373333	0.195547068	1.459358791	0.155595205	0.376167509
ROBO4	NIH	0.116073333	0.079540401	1.459300332	0.155611175	0.376167509
RASA1	NIH	0.22534	0.154634622	1.457241571	0.156174415	0.37721731
TET2	UCL	-0.689005803	0.47208954	-1.459481189	0.156676344	0.463996912
CCL25	NIH	-0.322286667	0.221482185	-1.455135848	0.156752188	0.378300454
CINP	UCL	-0.277966859	0.193785718	-1.434403223	0.157018248	0.464538806

GH1	NIH	1.183646667	0.814441425	1.453323261	0.157250897	0.379191158
MANEAL	NIH	0.31736	0.218531751	1.452237481	0.157550242	0.379413697
CNP	NIH	0.29756	0.204924399	1.452047691	0.157602612	0.379413697
GRHPR	UCL	-0.31933893	0.2221141	-1.43772471	0.157788593	0.466345862
ARHGEF1	NIH	0.382213333	0.263646063	1.449721376	0.158245669	0.380648505
MYO9B	UCL	-0.284357729	0.196001036	-1.450797076	0.158495484	0.467920689
SERPINI2	UCL	-0.332718007	0.229508564	-1.449697568	0.158641602	0.467920689
PSMD5	UCL	-0.580587365	0.400265805	-1.450504534	0.158859241	0.468090284
RSPO3	UCL	-0.156552375	0.108324936	-1.445210872	0.159619987	0.469858229
CYP24A1	NIH	-0.347053333	0.240290743	-1.444305879	0.159750774	0.383953174
UBE2L6	UCL	-0.339006779	0.236781033	-1.43173114	0.160495163	0.471959117
SCG3	UCL	0.23373253	0.161954877	1.443195373	0.160706303	0.47210505
HDAC9	NIH	-0.385406667	0.267560313	-1.440447811	0.160829973	0.386229616
DNAJB1	NIH	0.531893333	0.369872199	1.438046263	0.161504675	0.387531726
SEPTIN7	UCL	0.323475311	0.225355024	1.43540315	0.161730287	0.474636174
GRP	NIH	-0.31182	0.217159784	-1.435901227	0.162109213	0.388663483
CACNB3	UCL	-0.372755064	0.262294253	-1.421133175	0.162472023	0.476024133
REST	NIH	0.41026	0.285997241	1.434489361	0.162508103	0.389300739
TNFSF13B	UCL	0.144805381	0.100934029	1.434653723	0.162531133	0.476024133
KIAA1549L	UCL	-0.151875442	0.107354919	-1.414704084	0.162691792	0.476024133
TNC	NIH	0.200813333	0.140109088	1.433264158	0.162854888	0.389812232
WWP2	NIH	0.21058	0.146993596	1.43257942	0.163048954	0.389957638
LY75	NIH	0.254846667	0.1779585	1.432056728	0.163197218	0.389993351
VEGFD	NIH	-0.198406667	0.138628664	-1.431209545	0.163437752	0.390249326
LILRA5	UCL	-0.231641809	0.16160053	-1.433422334	0.163529433	0.477863674
SELENOP	UCL	0.124367826	0.086938727	1.43052274	0.163647464	0.477863674
SCARF2	UCL	0.164076435	0.114797353	1.429270189	0.164029051	0.478499915
MUC13	NIH	0.237093333	0.165962588	1.42859506	0.164181839	0.391706262
LRFN2	NIH	0.248386667	0.173968053	1.427771724	0.164416718	0.391946943
MAPT	UCL	-1.380450365	0.980364124	-1.408099634	0.164629085	0.479771501
MET	NIH	-0.10294	0.072157279	-1.426605894	0.164749759	0.392421045
CFHR2	NIH	0.229213333	0.160829509	1.425194507	0.165153663	0.393063031
GLO1	UCL	-0.367947891	0.259651626	-1.417082946	0.165356135	0.481410341

FAS	UCL	0.138856683	0.09706099	1.430612678	0.166024699	0.482875816
BRAP	NIH	0.32658	0.229710171	1.421704574	0.166155772	0.395126532
MMP9	UCL	-0.346202175	0.24314332	-1.423860523	0.166534782	0.48318784
CXCL5	UCL	-0.698197001	0.495391471	-1.409384382	0.1666024	0.48318784
ATP6AP2	UCL	-0.27847826	0.196719426	-1.415611389	0.166720492	0.48318784
VNN2	NIH	0.21922	0.154494066	1.418954172	0.166948921	0.396690167
GAGE2A	UCL	0.237486106	0.16688338	1.423066249	0.166972087	0.48318784
PRAME	UCL	-0.13843091	0.098891884	-1.39982074	0.167082686	0.48318784
ILKAP	UCL	-0.379420501	0.269505227	-1.407840971	0.167199887	0.48318784
LRRC37A2	UCL	0.260953132	0.183935608	1.41872003	0.167289119	0.48318784
SPARCL1	NIH	-0.195986667	0.138263466	-1.417487006	0.167373242	0.397375595
LACTB2	NIH	0.199206667	0.140616224	1.416669146	0.167610146	0.397615311
NPPC	UCL	-0.272543206	0.192314258	-1.417176284	0.167887976	0.48443885
LILRB4	NIH	0.259653333	0.183477233	1.415180126	0.168042143	0.398226901
HGF	NIH	0.234806667	0.165959591	1.414842401	0.168140247	0.398226901
MMP12	UCL	-0.360057052	0.254242414	-1.416195852	0.168366132	0.48491176
MRI1	NIH	0.415426667	0.293796672	1.413993779	0.168386959	0.398302258
CD63	NIH	0.225866667	0.159758987	1.41379631	0.168444408	0.398302258
APEX1	UCL	-0.358915752	0.256539829	-1.399064438	0.168523004	0.48491176
PTEN	UCL	-0.79814144	0.570036924	-1.40015744	0.168549555	0.48491176
ANGPT2	NIH	0.19264	0.136394411	1.412374591	0.168858484	0.398958857
KRT14	UCL	-0.257161763	0.184048779	-1.397247867	0.169003283	0.485739032
RILP	NIH	0.536433333	0.379974527	1.411761303	0.169037352	0.399059125
KLK11	NIH	0.31596	0.224226631	1.409110056	0.169812327	0.400565367
TSPAN1	UCL	-0.569965962	0.401441995	-1.419796558	0.170046422	0.487847745
ACE	UCL	0.113267768	0.080640837	1.404595631	0.170070767	0.487847745
CLASP1	UCL	-0.213025262	0.153407828	-1.388620544	0.170446858	0.488447223
PTP4A3	NIH	0.389053333	0.276587687	1.406618415	0.170543204	0.40178106
IGLC2	NIH	0.093353333	0.066376714	1.406416912	0.170602419	0.40178106
ECHS1	UCL	-0.496645478	0.358080211	-1.386967116	0.170947879	0.489403183
HDGFL2	NIH	0.336466667	0.239447247	1.405180772	0.170966039	0.401815456
ITGA2	NIH	-0.20756	0.147718536	-1.40510464	0.170988454	0.401815456
NID2	NIH	0.3674	0.261500905	1.404966459	0.171029143	0.401815456

IL5	NIH	-1.095506667	0.780125173	-1.404270371	0.171234231	0.401974419
TNFSF13	NIH	0.148706667	0.106035663	1.402421244	0.171779982	0.402932194
HMOX2	UCL	0.458498272	0.326977681	1.402231094	0.171999097	0.491735617
THPO	NIH	0.257626667	0.183830998	1.401432131	0.172072471	0.403294853
MAPK9	UCL	0.204529993	0.146468623	1.396408245	0.172256518	0.491735617
PTPRB	UCL	-0.207450949	0.147965434	-1.402023053	0.172267284	0.491735617
BIRC2	UCL	-0.430225325	0.306828701	-1.4021678	0.172467153	0.491825842
PON1	NIH	-0.132406667	0.094605309	-1.399569093	0.172624453	0.404264633
SDHB	UCL	0.544619239	0.388052142	1.403469224	0.172656157	0.491884938
CXCL13	UCL	-0.214007723	0.153059839	-1.398196451	0.172842926	0.491937559
CD7	NIH	0.2135	0.152721378	1.397970621	0.173099162	0.40505204
ANKRD54	NIH	0.411526667	0.295017627	1.394922302	0.174007293	0.406851583
RARRES2	UCL	-0.248986429	0.1785737	-1.394306268	0.17444901	0.495623135
EIF4EBP1	UCL	-0.324908955	0.23412396	-1.387764647	0.174476978	0.495623135
SNX2	NIH	0.31896	0.228955778	1.393107452	0.174549737	0.407793914
LAMB1	NIH	0.17666	0.12690348	1.392081604	0.174856943	0.408185601
SLIT2	UCL	0.291298113	0.209695972	1.38914501	0.174975842	0.496141862
THBS4	UCL	0.335487535	0.241274576	1.39048026	0.174999062	0.496141862
APOM	NIH	0.257253333	0.184953675	1.390906852	0.175209263	0.40845005
SPINK8	NIH	0.316646667	0.227676779	1.390772779	0.175249508	0.40845005
TBR1	UCL	-0.385524114	0.276663567	-1.393476262	0.175376262	0.496314348
SKIV2L	UCL	0.154298537	0.111804756	1.380071317	0.175399494	0.496314348
FGR	NIH	0.444533333	0.319761046	1.390204776	0.175420089	0.408522103
APOE	UCL	0.247400945	0.17838534	1.386890568	0.175724764	0.496746054
CHRM1	NIH	0.46072	0.331756539	1.388729221	0.175863833	0.409229684
RAPGEF2	UCL	-0.291246407	0.21143555	-1.377471326	0.176000243	0.496746054
LTB	NIH	0.206726667	0.14892146	1.388159016	0.176035548	0.409303638
RBP1	UCL	0.20933639	0.151481401	1.381928004	0.17623742	0.496746054
MAP1LC3B2	UCL	-0.233765241	0.167776591	-1.393312617	0.176417313	0.496746054
CYTH3	UCL	-0.286051315	0.208316163	-1.373159485	0.176473488	0.496746054
IL6ST	UCL	0.117173088	0.0844383	1.387677016	0.176571724	0.496746054
CD276	UCL	0.244115232	0.175862608	1.388101968	0.176787805	0.496875725
AHSP	NIH	0.851853333	0.614837947	1.385492449	0.176840319	0.41084824

DKK1	NIH	0.212946667	0.153753557	1.384986932	0.17699321	0.410877094
CLPS	UCL	-0.375369854	0.271007291	-1.385091349	0.177154864	0.497429074
DCTN6	NIH	0.459626667	0.332063044	1.38415483	0.177245101	0.411135543
LTA4H	UCL	-0.298505425	0.215361504	-1.386066772	0.177334463	0.497455504
PYY	NIH	0.4602	0.332593522	1.383670965	0.177391704	0.411149551
S100P	UCL	-0.169837316	0.122409777	-1.387448943	0.177684289	0.497821384
CEACAM20	UCL	-0.254644112	0.185213233	-1.374869974	0.1779695	0.497821384
ARNTL	UCL	0.277155703	0.200762062	1.380518311	0.177975828	0.497821384
CCL20	NIH	0.649946667	0.470722296	1.380743321	0.178280763	0.41231229
CERT	NIH	0.272166667	0.197141831	1.380562739	0.178335716	0.41231229
C9	NIH	-0.240733333	0.174393511	-1.380403042	0.178384324	0.41231229
AKR1C4	NIH	0.61254	0.443816942	1.380163626	0.178457217	0.41231229
IL2RA	UCL	-0.263689641	0.190806389	-1.381974909	0.178492573	0.498789475
EPHA4	UCL	0.164907009	0.11947947	1.38021209	0.178745486	0.498862989
HDDC2	UCL	-0.140172817	0.102149388	-1.372233552	0.178883221	0.498862989
USP25	NIH	0.291233333	0.211254823	1.378587855	0.17893756	0.413095788
STX7	UCL	-0.323602893	0.235402252	-1.37468053	0.179030884	0.498862989
ASS1	NIH	0.511826667	0.371663581	1.377123541	0.179384836	0.413801771
IL3RA	UCL	0.154656734	0.113007166	1.368556883	0.179487105	0.498908822
VMO1	UCL	-0.371082583	0.269475738	-1.377053777	0.179690664	0.498908822
CDC27	UCL	-0.496201632	0.361496354	-1.372632466	0.179707748	0.498908822
ATP2B4	UCL	0.287977288	0.209463708	1.374831427	0.179730068	0.498908822
ORM1	NIH	0.154353333	0.112200452	1.375692617	0.179822759	0.414172876
DEFB4A_DEFB4B	NIH	-0.70304	0.511051849	-1.375672549	0.179828907	0.414172876
CKMT1A_CKMT1B	UCL	0.274227785	0.199414903	1.375161942	0.180183432	0.499371659
ZNF75D	NIH	0.364013333	0.264864757	1.374336616	0.180238525	0.414719376
NFKB1	NIH	0.371913333	0.270716449	1.373811361	0.180399777	0.414719376
DCUN1D2	UCL	0.508723409	0.371896256	1.367917536	0.180440434	0.499371659
MED18	NIH	0.22066	0.160653786	1.373512605	0.180491544	0.414719376
SCG2	UCL	-0.192807554	0.140408357	-1.373191438	0.180571472	0.499371659
ATF4	UCL	-0.175400897	0.129363195	-1.355879448	0.180580172	0.499371659
SEMA6C	NIH	-0.144286667	0.105107014	-1.372759637	0.180722992	0.414925236
SIRT1	NIH	0.28548	0.208033997	1.372275705	0.180871866	0.414941339

ECE1	UCL	0.189722944	0.139032143	1.364597703	0.181032084	0.500148187
AGXT	NIH	0.4181	0.304846385	1.37151044	0.181107483	0.414984939
KLK7	NIH	-0.21954	0.160097132	-1.371292524	0.181174621	0.414984939
NAGK	UCL	-0.368097741	0.268683485	-1.370005084	0.181283403	0.500189178
LY9	UCL	0.261154947	0.190339911	1.372045126	0.181389165	0.500189178
SCN3B	NIH	0.207733333	0.151621293	1.370080217	0.181548478	0.415515884
PTP4A3	UCL	0.398239597	0.291261396	1.367292757	0.181821764	0.500909536
HBEGF	NIH	0.28184	0.205869173	1.369024781	0.18187445	0.415779407
TXNDC15	NIH	0.11782	0.086076204	1.368787131	0.181947911	0.415779407
MCFD2	NIH	0.1217	0.08903448	1.366886184	0.182536359	0.416487004
MNDA	NIH	0.55962	0.409418066	1.366866894	0.182542338	0.416487004
FSHB	UCL	-0.174011708	0.127109161	-1.368994228	0.182669005	0.502691488
AKR1B10	NIH	0.416453333	0.304808147	1.366280191	0.18272426	0.416577132
UROS	UCL	-0.605371245	0.441992372	-1.369641838	0.182881107	0.502691488
CANT1	UCL	0.113498177	0.083177675	1.364526924	0.183061868	0.502691488
GAPDH	UCL	-0.173090451	0.128140014	-1.350791568	0.183327431	0.502691488
C1QTNF1	UCL	0.224866849	0.164441172	1.367460754	0.183328473	0.502691488
ATP5IF1	NIH	0.365106667	0.267981776	1.362430954	0.183921326	0.418979657
CFHR4	NIH	-0.267653333	0.196567427	-1.361636248	0.184169231	0.419188524
CXCL10	NIH	0.234686667	0.172409249	1.361218539	0.184299638	0.419188524
NBN	NIH	0.420646667	0.309222611	1.360336054	0.184575383	0.419489507
CD244	UCL	0.157827745	0.115894798	1.36181906	0.184621212	0.504514407
KLRF1	UCL	-0.188615628	0.138914579	-1.357781372	0.184761325	0.504514407
ENSA	UCL	-0.33082782	0.242673364	-1.363263832	0.184776054	0.504514407
PAXX	UCL	-0.175332259	0.129547397	-1.353421712	0.184837932	0.504514407
IL9	NIH	0.37314	0.274529655	1.359197422	0.18493164	0.419972862
EPS8L2	UCL	0.195474894	0.143447491	1.362693016	0.184996124	0.504514407
LAMP3	UCL	-0.341418383	0.250870288	-1.360935905	0.18502889	0.504514407
NAA80	NIH	0.198513333	0.146184755	1.357961936	0.185318807	0.42052561
TSPAN8	UCL	-0.620658049	0.456849735	-1.358560599	0.185510259	0.505355532
GORASP2	NIH	0.182226667	0.134298735	1.356875528	0.18565978	0.420604995
BDNF	NIH	0.27006	0.199064772	1.356643856	0.185732554	0.420604995
NEO1	NIH	-0.075146667	0.055398434	-1.356476366	0.185785181	0.420604995

HSPA1A	UCL	-0.246885442	0.182871233	-1.350050735	0.186340904	0.507145682
NPM1	NIH	0.440733333	0.325427339	1.354321781	0.186463206	0.421813518
PTH1R	UCL	-0.109644191	0.080991119	-1.353780421	0.186553133	0.507250985
HNF1A	NIH	0.365233333	0.26984223	1.353506951	0.186720127	0.422068293
ROBO2	UCL	0.117549902	0.086927054	1.352282132	0.187001655	0.507892948
PAGR1	NIH	0.270206667	0.199791837	1.352440975	0.187056651	0.422502474
MIA	UCL	-0.211786927	0.156390747	-1.354216478	0.187136745	0.507892948
VPS53	NIH	0.199406667	0.147616742	1.350840454	0.187562815	0.423318854
PRDX2	NIH	0.34278	0.253859541	1.350274247	0.187742133	0.423396869
AKT3	UCL	-0.313537512	0.23408845	-1.339397615	0.187788381	0.509188719
S100A11	NIH	0.216566667	0.160458407	1.349674793	0.187932126	0.423498819
EDEM2	NIH	0.316426667	0.23453274	1.349179081	0.188089351	0.423526829
RNASEH2A	NIH	0.30034	0.22274006	1.34838789	0.188340505	0.423766137
KLB	UCL	0.460603911	0.341982537	1.346863837	0.188829029	0.511221121
ATP1B3	NIH	0.33726	0.250410873	1.3468265	0.188836915	0.424556477
MZT1	UCL	-0.187511214	0.140677746	-1.332913125	0.188887722	0.511221121
MICALL2	NIH	0.26894	0.199890607	1.345435903	0.189279881	0.424910479
BCAN	UCL	0.150325159	0.112072024	1.341326352	0.189305525	0.511616984
FGF23	UCL	-0.279319415	0.207670491	-1.345012542	0.189384049	0.511616984
FAM3C	NIH	0.15	0.111589404	1.344213645	0.18966989	0.424910479
CYB5R2	NIH	0.354633333	0.263843944	1.344102609	0.189705351	0.424910479
LRTM1	NIH	-0.488473333	0.363482476	-1.343870379	0.189779534	0.424910479
CEACAM18	NIH	0.32608	0.242661496	1.343764897	0.189813237	0.424910479
ADAM15	NIH	0.19504	0.145173515	1.343495745	0.189899255	0.424910479
MITD1	NIH	0.284593333	0.211926186	1.342888947	0.190093291	0.424910479
CNST	NIH	0.351166667	0.261628119	1.34223595	0.190302272	0.424910479
MAPRE3	NIH	0.563866667	0.42015237	1.342052804	0.190360917	0.424910479
SLAMF7	NIH	-0.424073333	0.316051892	-1.341783876	0.190447056	0.424910479
CD200R1	UCL	0.316618524	0.235790492	1.342795977	0.1906045	0.514438555
CHCHD6	NIH	0.175546667	0.130883122	1.34124755	0.190618933	0.424969801
MATN2	UCL	0.189031605	0.140848746	1.342089377	0.191007628	0.515051012
HRC	UCL	-0.162516575	0.121263317	-1.340195696	0.191226125	0.515164942
NAAA	NIH	-0.309793333	0.231613841	-1.337542401	0.191809619	0.427025798

LATS1	NIH	0.32354	0.241988814	1.33700395	0.191983134	0.427025798
APP	NIH	0.174933333	0.130841825	1.336983291	0.191989794	0.427025798
KDM3A	NIH	0.37702	0.28208159	1.33656365	0.192125111	0.427025798
DPP10	UCL	0.210003919	0.15745526	1.333737081	0.192987133	0.518701011
GET3	UCL	-0.406479895	0.30453772	-1.334744001	0.193052321	0.518701011
AXL	UCL	0.116538225	0.08728502	1.335145773	0.193071057	0.518701011
SERPINA11	UCL	0.304977098	0.22861775	1.334004458	0.193250222	0.518705599
ECE1	NIH	0.1731	0.129854757	1.333027789	0.193268218	0.428738767
PROS1	NIH	0.126833333	0.09519288	1.332382566	0.193477379	0.428738767
CRTAM	NIH	0.30556	0.229354947	1.33225816	0.193517728	0.428738767
IGDCC3	UCL	-0.14437419	0.109556544	-1.317805269	0.193549565	0.518967839
ITGAV	NIH	-0.10892	0.081790057	-1.331702214	0.193698117	0.428738767
SIGLEC8	UCL	-0.272021946	0.204079108	-1.332924025	0.193703015	0.518967839
DBH	NIH	0.639326667	0.480172916	1.331450912	0.193779701	0.428738767
TNFAIP2	NIH	0.201306667	0.151234536	1.331089262	0.193897155	0.428738767
PCARE	NIH	0.656166667	0.492982814	1.331013269	0.193921842	0.428738767
DDX4	NIH	0.320033333	0.240667398	1.329774351	0.194324666	0.429304871
AIFM1	NIH	0.328273333	0.246981344	1.329142225	0.194530446	0.429435136
APLP1	UCL	0.325075528	0.245790433	1.32257193	0.19483725	0.521276138
H2AP	UCL	0.546402635	0.411755518	1.327007439	0.194921252	0.521276138
PNLIPRP1	NIH	0.399353333	0.300763658	1.327797833	0.194968655	0.429773072
RPGR	NIH	0.35036	0.263870792	1.327771056	0.19497739	0.429773072
PTPRN2	UCL	-0.187310368	0.141353421	-1.325120871	0.195301531	0.5218157
SPRY2	NIH	-0.261573333	0.197228488	-1.326245191	0.195475692	0.430546987
SIGLEC10	UCL	-0.191956413	0.144590443	-1.327587146	0.195519736	0.521921634
SPON1	UCL	0.177443577	0.133573281	1.328436163	0.19580929	0.522217659
CLEC1B	NIH	0.27654	0.208749277	1.324747106	0.195965879	0.431223538
MYL6B	NIH	0.582613333	0.439939114	1.324304465	0.196110896	0.431223538
APOD	NIH	-0.212146667	0.16025453	-1.323810736	0.196272749	0.431223538
SYAP1	NIH	0.166713333	0.125963375	1.323506405	0.196372565	0.431223538
IL2RA	NIH	0.222053333	0.168007595	1.321686279	0.196970359	0.431784516
SERPINA6	UCL	0.089199271	0.067815683	1.315319217	0.197050558	0.524777949
VWA1	UCL	-0.238883964	0.180762882	-1.321532171	0.197128357	0.524777949

SCRG1	NIH	0.174066667	0.131769531	1.320993293	0.197198329	0.431784516
GP5	NIH	-0.131093333	0.099239803	-1.320975354	0.197204233	0.431784516
STX16	NIH	0.25532	0.193287769	1.320932005	0.197218501	0.431784516
ADAMTS4	NIH	0.203526667	0.154150158	1.320314357	0.197421875	0.431906495
TPR	UCL	0.224317246	0.17062437	1.314684683	0.197543957	0.525405811
ANGPTL2	NIH	0.308966667	0.234253137	1.318943559	0.197873817	0.432222794
AP2B1	UCL	-0.189218925	0.144234036	-1.311888162	0.197934534	0.525793103
PRKRA	NIH	0.314426667	0.238427691	1.318750625	0.19793749	0.432222794
CCN1	UCL	-0.547337299	0.415023291	-1.318811043	0.198049335	0.525793103
INPP5J	NIH	0.273793333	0.207786735	1.317665124	0.198296027	0.432222794
AMY2A	NIH	-0.310246667	0.235488209	-1.317461575	0.198363315	0.432222794
OLFM4	NIH	0.79466	0.603390789	1.316990605	0.198519071	0.432222794
NRCAM	NIH	0.160066667	0.12155042	1.316874646	0.198557434	0.432222794
IL10	UCL	-0.396711035	0.300894056	-1.318440914	0.198564368	0.526682076
GAL	NIH	-0.407286667	0.309352882	-1.316576281	0.198656171	0.432222794
TTF2	NIH	0.691893333	0.525636154	1.316297077	0.198748601	0.432222794
FAM20A	UCL	0.258018376	0.196044795	1.316119492	0.198901568	0.527098172
PFDN4	UCL	-0.208693128	0.160291711	-1.301958327	0.199493304	0.527935661
NUDT16	UCL	-0.416130732	0.318827676	-1.30519012	0.199578825	0.527935661
ZNF830	NIH	0.291833333	0.222240168	1.313143953	0.19979474	0.433891802
KLK4	NIH	0.431706667	0.328771619	1.31308982	0.199812737	0.433891802
LILRB2	NIH	0.147833333	0.112638269	1.312460987	0.200021892	0.434023764
SORCS2	UCL	-0.192451109	0.146595326	-1.312805213	0.200035434	0.528312423
CRYGD	UCL	-0.565134839	0.431597939	-1.309401152	0.200098697	0.528312423
FOLR1	UCL	-0.162046494	0.124230671	-1.304400062	0.200299017	0.528312423
TMPRSS11B	NIH	0.615286667	0.469209899	1.311324991	0.200400159	0.434427466
CTRL	UCL	-0.313839633	0.239063046	-1.312790237	0.200444227	0.528312423
ISM2	NIH	0.100393333	0.076577064	1.311010475	0.200504985	0.434427466
JCHAIN	NIH	-0.2336	0.178425512	-1.309229812	0.201099261	0.435392552
GLA	UCL	-0.146577389	0.111954374	-1.309260052	0.201397751	0.530344859
FOLH1	UCL	0.733537834	0.560005322	1.309876541	0.201578221	0.530344859
ZNF174	NIH	0.210133333	0.160772445	1.30702331	0.201837535	0.436667743
ANP32C	UCL	-0.497036221	0.379593673	-1.309390165	0.20187796	0.530351286

BAG4	UCL	-0.222793594	0.171849519	-1.296445844	0.202064403	0.530351286
FOXO1	UCL	-0.543070579	0.417184507	-1.301751551	0.202124985	0.530351286
PLTP	NIH	-0.21024	0.160960541	-1.30615863	0.202127415	0.43687713
IL3RA	NIH	-0.174366667	0.133621996	-1.30492488	0.202541579	0.43687713
GOPC	NIH	0.257946667	0.197682146	1.304855658	0.202564836	0.43687713
SEPTIN7	NIH	-0.53374	0.409046377	-1.304839818	0.202570158	0.43687713
GID8	NIH	0.328513333	0.251828979	1.304509649	0.202681116	0.43687713
ADAMTSL4	NIH	0.205453333	0.157660108	1.303140891	0.203141608	0.437547277
RPA2	UCL	-0.162075507	0.125740107	-1.288972241	0.203760843	0.534164076
STOML2	UCL	0.639812424	0.49336262	1.296840089	0.204005006	0.534324939
PGLYRP1	NIH	0.27972	0.215076133	1.300562718	0.204011163	0.439000526
MEGF11	NIH	0.16526	0.127099598	1.300240142	0.204120161	0.439000526
PPBP	NIH	0.181973333	0.140008393	1.299731608	0.204292084	0.439000526
FAM172A	NIH	0.15822	0.121767326	1.299363343	0.204416655	0.439000526
SNX18	NIH	0.25912	0.199500749	1.298842239	0.204593025	0.439056933
CACNA1C	UCL	-0.247263773	0.188803848	-1.309633124	0.205213836	0.537009885
PALLD	NIH	0.276686667	0.213383138	1.29666603	0.205330836	0.440317226
GBP2	UCL	0.208412307	0.162308787	1.284048208	0.205470657	0.537201011
PDP1	UCL	-0.656048324	0.507972276	-1.291504192	0.205881431	0.537457286
ENDOU	UCL	-0.274719451	0.211995257	-1.29587546	0.205936421	0.537457286
GFRA2	NIH	0.07922	0.06120359	1.294368508	0.206111987	0.441545948
CEACAM1	NIH	0.21262	0.164300372	1.294093235	0.206205731	0.441545948
SCARA5	UCL	0.14265513	0.110548785	1.290426945	0.206662022	0.538125179
IL18RAP	UCL	1.030493429	0.794972529	1.296262941	0.206685435	0.538125179
LMNB1	UCL	0.344468344	0.268265671	1.284056744	0.206744638	0.538125179
PSME2	UCL	-0.177070538	0.137907845	-1.28397727	0.207475663	0.539527662
ADD1	UCL	-0.335629152	0.261965775	-1.281194657	0.207652624	0.539527662
CD1C	NIH	-0.153513333	0.119051712	-1.289467666	0.20778586	0.444384395
ICA1	NIH	0.25632	0.198840131	1.289075797	0.207920149	0.444384395
IL4	NIH	1.03568	0.803674104	1.288681563	0.208055317	0.444384395
ANGPTL7	NIH	0.177893333	0.138069039	1.288437539	0.208139016	0.444384395
HGF	UCL	-0.162478958	0.125969916	-1.289823504	0.208331357	0.539925064
CPA1	UCL	-0.306168203	0.237671175	-1.288200822	0.208361485	0.539925064

CBLN1	UCL	-0.317162661	0.249192645	-1.272760924	0.208361781	0.539925064
MPO	NIH	0.433333333	0.336536366	1.287627067	0.208417191	0.444653744
RECK	UCL	0.087395338	0.067888444	1.287337468	0.208672797	0.539925064
FDX1	NIH	0.496193333	0.385596402	1.286820446	0.208694326	0.44469998
SEMA7A	UCL	0.157889119	0.122510896	1.28877614	0.208830179	0.539925064
SCN2B	UCL	0.120171846	0.093629932	1.283476807	0.208913872	0.539925064
TXNDC5	NIH	0.173533333	0.134925246	1.286144276	0.208926858	0.44469998
INSL3	NIH	0.304833333	0.237083269	1.285764847	0.209057428	0.44469998
LEO1	NIH	0.150833333	0.117371694	1.285091219	0.209289394	0.44469998
CD244	NIH	0.174646667	0.135908068	1.285035315	0.209308653	0.44469998
RCC1	NIH	0.252406667	0.196438841	1.284912215	0.209351067	0.44469998
ADCYAP1R1	UCL	0.591216542	0.460078204	1.285034884	0.209556419	0.540793147
JAM2	UCL	-0.118898891	0.092497071	-1.285434117	0.209619786	0.540793147
S100A14	NIH	0.377273333	0.293906095	1.283652635	0.209785431	0.445299264
CALB2	UCL	-0.348350257	0.272161866	-1.279937789	0.20983762	0.540877744
NUMB	NIH	0.237453333	0.185119823	1.282700739	0.210114147	0.445673589
SH3BGRL2	UCL	0.414906708	0.323567415	1.282288292	0.210135773	0.540986965
LRP2BP	UCL	0.484912838	0.378628259	1.280709577	0.210250151	0.540986965
SUMF1	NIH	0.330933333	0.258217296	1.281607924	0.21049201	0.446151543
INSL4	UCL	-0.217136155	0.169257767	-1.282872618	0.210667567	0.541584257
EHD3	NIH	0.41522	0.324367577	1.28009095	0.211017394	0.446941259
REPS1	UCL	-0.221064483	0.173491309	-1.274210703	0.211342465	0.542363605
ERVV-1	UCL	0.224300324	0.175664096	1.276870624	0.211368242	0.542363605
CD276	NIH	0.20964	0.163909846	1.278995772	0.211397316	0.44742196
GKN1	UCL	0.169179114	0.132722117	1.274686672	0.211527372	0.542363605
CPLX2	NIH	-0.167206667	0.130793736	-1.278399657	0.211604329	0.447536271
IL2RG	NIH	0.210346667	0.164615285	1.277807624	0.211810078	0.447647744
CTRC	UCL	-0.304882432	0.238321341	-1.279291357	0.21194722	0.542963824
CNPY2	UCL	-0.20633784	0.16263989	-1.268679161	0.212243319	0.54304857
ACP6	UCL	-0.341113122	0.266935675	-1.2778851	0.212457431	0.54304857
CGB3_CGB5_CGB8	UCL	-0.172197759	0.136556674	-1.260998486	0.212537655	0.54304857
SERPINA7	NIH	0.111626667	0.087604041	1.274218233	0.213060761	0.449098523
GFRA3	NIH	-0.177253333	0.139108143	-1.274212488	0.213062767	0.449098523

TDRKH	NIH	0.30328	0.238018407	1.27418717	0.21307161	0.449098523
CD28	NIH	0.17764	0.139426611	1.274075292	0.213110684	0.449098523
STX3	UCL	-0.131041979	0.103333381	-1.268147598	0.21311896	0.543343299
PPT1	UCL	0.443449989	0.347951409	1.274459528	0.213204614	0.543343299
CD46	UCL	-0.103498751	0.081113379	-1.275976315	0.213210662	0.543343299
HLA-A	UCL	-0.234045397	0.183714347	-1.273963628	0.213738619	0.544214272
CXCL8	NIH	0.26616	0.209263672	1.271888222	0.213875631	0.450386048
AOC3	UCL	0.157278106	0.123656749	1.271892624	0.214338905	0.544831685
PVALB	UCL	-0.503824762	0.397777171	-1.266600495	0.214353896	0.544831685
EIF4G3	NIH	0.2688	0.211584778	1.270412754	0.214392868	0.450753584
CST7	NIH	0.5682	0.447361821	1.270112855	0.214498116	0.450753584
DAPK2	NIH	0.311513333	0.245272194	1.270071948	0.214512475	0.450753584
CXCL6	UCL	-0.360511135	0.284975105	-1.265061854	0.214643301	0.544949637
TCL1A	NIH	-0.876746667	0.690681279	-1.269393992	0.214750557	0.45092992
GLRX	UCL	-0.248021669	0.196317488	-1.263370226	0.214773172	0.544949637
FFA	NIH	0.296403695	0.233447623	1.269679642	0.215033421	0.456421215
PHYKPL	NIH	0.139386667	0.109968596	1.267513375	0.215412036	0.451795591
AMDHD2	NIH	-0.152606667	0.120414572	-1.267343841	0.215471743	0.451795591
LSP1	NIH	0.28358	0.224067158	1.2656027	0.216085672	0.452758302
CUZD1	UCL	-0.439008305	0.346768316	-1.265998895	0.216331998	0.54803449
BRME1	NIH	-0.196426667	0.155302809	-1.264797899	0.216369894	0.453029305
EPO	UCL	-0.548440229	0.432873163	-1.266976738	0.216496842	0.54803449
TNFRSF9	UCL	-0.206038706	0.162778232	-1.26576326	0.216551432	0.54803449
CADPS	NIH	0.305606667	0.241753724	1.264123927	0.21660813	0.453203706
ABCA2	NIH	0.193193333	0.152915736	1.263397332	0.216865191	0.453417216
ELOA	NIH	0.32274	0.255789552	1.261740355	0.217452277	0.454043596
LYPD3	NIH	0.176646667	0.140009575	1.261675611	0.21747524	0.454043596
HIP1R	UCL	-0.167314593	0.132199969	-1.265617493	0.217564218	0.549480679
CFH	UCL	-0.09333175	0.074138145	-1.258889741	0.217673627	0.549480679
LECT2	UCL	-0.400421292	0.317353159	-1.261752976	0.217872208	0.549480679
TPMT	UCL	-0.285722729	0.228365496	-1.251164183	0.217874823	0.549480679
C5	NIH	0.086146667	0.068350115	1.260373402	0.217937509	0.454562525
LRIG1	NIH	-0.198893333	0.157839307	-1.260100146	0.218034606	0.454562525

DSCAM	UCL	0.252620456	0.200380943	1.260701001	0.218138959	0.549672565
PI3	NIH	-0.223986667	0.17783268	-1.259536022	0.218235162	0.454656588
F11	NIH	0.14708	0.116825438	1.258972377	0.218435687	0.454750453
CRYM	UCL	-0.40383836	0.321723641	-1.255233711	0.218525336	0.550171883
PGM2	UCL	0.235857866	0.188016765	1.254451245	0.218737111	0.550231133
GPR101	UCL	0.318513985	0.253674059	1.255603299	0.219062317	0.550575367
EIF2AK2	NIH	0.447673333	0.356094814	1.257174538	0.219076229	0.455759579
TLR4	UCL	-0.264872978	0.213341363	-1.241545353	0.219579553	0.551271775
APOA1	UCL	0.071736791	0.05714359	1.255377748	0.219716599	0.551271775
CSH1	NIH	0.218533333	0.174431483	1.252831937	0.220629291	0.458263079
CD27	NIH	0.188326667	0.150321717	1.252824082	0.220632108	0.458263079
PEPD	NIH	0.093266667	0.074464618	1.252496407	0.220749633	0.458263079
CSPG5	UCL	0.205068987	0.163741722	1.252393006	0.220832996	0.55323212
FDX2	UCL	-0.359133915	0.28719807	-1.25047468	0.220876457	0.55323212
SIAE	NIH	0.205273333	0.163991294	1.251733115	0.221023584	0.45850637
ATP6V1G2	NIH	0.634453333	0.508175675	1.248492134	0.222189653	0.460598679
RTBDN	NIH	-0.111386667	0.089277036	-1.247651929	0.222492707	0.46090026
HAVCR1	UCL	-0.418270306	0.335245647	-1.247653206	0.223146362	0.558142172
WAS	UCL	-0.18096794	0.146928649	-1.231672247	0.223218679	0.558142172
SEZ6L	UCL	0.120616169	0.096961808	1.243955447	0.22366529	0.558591858
IFNL2	NIH	0.365393333	0.293681335	1.244183032	0.223747206	0.462945011
KDM3A	UCL	0.561581166	0.451021237	1.245132425	0.223819413	0.558591858
STAT2	NIH	0.20314	0.16332658	1.243765713	0.223898485	0.462945011
FCRL5	NIH	-0.362306667	0.291334406	-1.243610981	0.223954595	0.462945011
GRIK2	UCL	0.131537907	0.10608015	1.239986048	0.22397183	0.558591858
ARHGAP1	NIH	0.164433333	0.132313686	1.242753778	0.224265633	0.463072251
MOG	NIH	-0.181386667	0.146031056	-1.24211022	0.224499363	0.463072251
ESR1	NIH	0.308406667	0.248349471	1.241825342	0.224602885	0.463072251
LRTM2	NIH	-0.13476	0.108533748	-1.241641448	0.224669729	0.463072251
SERPING1	UCL	-0.092624841	0.074833787	-1.237740934	0.224737548	0.559686716
IL13RA2	UCL	-0.160689696	0.130308171	-1.233151341	0.224793775	0.559686716
RBM25	NIH	0.2396	0.193029362	1.241261941	0.224807725	0.463072251
CSF2RA	NIH	0.324953333	0.261950807	1.240512817	0.225080308	0.463307461

UMOD	NIH	-0.310433333	0.25047359	-1.239385491	0.225490976	0.463826375
MCEE	UCL	0.128504246	0.104855938	1.225531411	0.225504371	0.560816762
IGFBPL1	UCL	-0.158936224	0.128061663	-1.241091363	0.225631376	0.560816762
ARG2	UCL	-0.133548314	0.108352422	-1.232536494	0.22585089	0.56088543
SPP1	NIH	-0.24034	0.194160134	-1.237844224	0.226053349	0.464509988
REPS1	NIH	0.421746667	0.340776624	1.23760445	0.226140931	0.464509988
DLL4	NIH	-0.501286667	0.405388818	-1.236557705	0.226523577	0.464723799
PDCD1	NIH	0.199493333	0.161349451	1.236405405	0.226579292	0.464723799
PSMA1	UCL	0.429127912	0.347138343	1.236187015	0.226636542	0.562358754
NME3	NIH	-0.124733333	0.10097488	-1.235290727	0.226987381	0.464723799
SCGN	UCL	0.255736052	0.206898236	1.236047519	0.227079622	0.562578316
FCRL5	UCL	0.50812139	0.410269465	1.238506476	0.22710996	0.562578316
TFPI	NIH	0.177073333	0.143410915	1.234727032	0.227193963	0.464723799
CLEC10A	NIH	-0.199906667	0.161903776	-1.234725163	0.227194649	0.464723799
ANG	NIH	0.183173333	0.1483527	1.234715199	0.227198302	0.464723799
IL20RA	UCL	-0.219187688	0.177707857	-1.233415855	0.227487383	0.562784748
KIR3DL1	UCL	-0.998085649	0.808899841	-1.233880387	0.227578369	0.562784748
PENK	UCL	0.136906509	0.111332303	1.229710558	0.227962564	0.563258306
PAIP2B	NIH	-0.247306667	0.200760554	-1.231848893	0.228250942	0.466550669
SULT2A1	UCL	-0.316598008	0.256732503	-1.233182414	0.22853687	0.563845168
NEK7	UCL	0.497854744	0.404641798	1.230359165	0.228786982	0.563845168
TRAF3IP2	UCL	0.182490694	0.149077379	1.224134042	0.228950081	0.563845168
DCBLD2	UCL	0.230588861	0.187275511	1.231281442	0.229097743	0.563845168
KLK6	UCL	-0.143671685	0.117475978	-1.222987772	0.229164577	0.563845168
RICTOR	NIH	0.36632	0.298008319	1.22922743	0.229216868	0.46789693
PTPRR	NIH	-0.156286667	0.127145731	-1.22919319	0.229229505	0.46789693
LAYN	NIH	0.200833333	0.163482339	1.228471126	0.229496111	0.468012299
KLK15	NIH	0.305326667	0.248602206	1.2281736	0.229606034	0.468012299
SMS	NIH	0.171673333	0.139885554	1.227241332	0.229950721	0.468388482
PINLYP	UCL	0.281875409	0.22958041	1.227785108	0.230303789	0.566171551
POLR2A	NIH	0.172786667	0.140970608	1.22569285	0.230524099	0.469229637
ZBP1	UCL	0.230991336	0.188614726	1.224672858	0.23079367	0.566624788
PTTG1	NIH	-0.17552	0.143306339	-1.22478881	0.230859345	0.469521301

NPR1	UCL	0.252685876	0.206345202	1.224578392	0.23089782	0.566624788
CSPG5	NIH	-0.396213333	0.323587123	-1.22444098	0.230988428	0.469521301
ALCAM	UCL	0.110133378	0.089862873	1.225571527	0.231069705	0.566624788
SNRPB2	UCL	-0.177005892	0.146259236	-1.210220264	0.231277998	0.566660174
RAB11FIP3	NIH	0.34732	0.283883212	1.223460864	0.23135245	0.469934665
SERPIND1	UCL	-0.160602215	0.131314462	-1.223035248	0.231536487	0.566706604
SHD	UCL	-0.838389517	0.684575779	-1.224684751	0.231684705	0.566706604
CAPS	NIH	0.519633333	0.425271094	1.221887264	0.231937795	0.470571284
SLC39A14	NIH	0.17364	0.142123601	1.221753454	0.231987621	0.470571284
STX8	NIH	0.310493333	0.254238731	1.221266846	0.232168882	0.470612598
ASPN	UCL	0.148176816	0.121557441	1.218985981	0.232257786	0.566902535
FGR	UCL	-0.241131307	0.199206565	-1.210458636	0.232350708	0.566902535
ADAM22	UCL	0.168157762	0.137772342	1.220548041	0.232595477	0.566902535
EXTL1	UCL	0.351685361	0.288268709	1.219991451	0.232691486	0.566902535
IGF2R	UCL	-0.134703684	0.110314412	-1.22108872	0.232734534	0.566902535
KLRF1	NIH	0.446013333	0.365701798	1.219609353	0.232787091	0.471538948
GPD1	UCL	0.270750106	0.221964382	1.219790776	0.233173572	0.566961388
LGALS3	UCL	0.125947692	0.103274147	1.219547157	0.233221807	0.566961388
IMMT	UCL	0.250104485	0.20539915	1.217651025	0.233340592	0.566961388
IFI30	NIH	0.1901	0.156245291	1.216676666	0.233883935	0.473432879
TLR1	NIH	-0.19784	0.162679909	-1.216130506	0.234088629	0.473519529
BSG	NIH	0.07144	0.058785872	1.215257974	0.234415919	0.473853879
ENAH	NIH	0.2145	0.176714171	1.213824556	0.234954342	0.474527985
TREML2	NIH	0.18748	0.15449441	1.213506693	0.235073863	0.474527985
IL1RL2	UCL	0.196410646	0.161874585	1.21335073	0.235140315	0.570859752
PTPRF	UCL	0.117545955	0.097058461	1.211084059	0.235512385	0.571022957
LPCAT2	UCL	-0.064125399	0.053484863	-1.198944826	0.23559825	0.571022957
CRH	UCL	0.539524394	0.445327115	1.21152379	0.236039614	0.571618716
SOWAHA	NIH	0.228886667	0.189074014	1.210566497	0.236181573	0.475758313
LAP3	NIH	-0.60642	0.501074027	-1.210240338	0.236304692	0.475758313
IL10RB	NIH	0.112233333	0.092760774	1.209922345	0.236424774	0.475758313
TNFRSF9	NIH	0.215533333	0.178151049	1.209834769	0.236457853	0.475758313
PMCH	NIH	0.218253333	0.18041459	1.20973217	0.236496611	0.475758313

BSND	NIH	0.137013333	0.113303401	1.20926055	0.236674831	0.475789608
ZNF174	UCL	0.159442577	0.132456391	1.203736376	0.236935913	0.573314299
PSMC3	NIH	-0.187573333	0.155227312	-1.208378412	0.237008449	0.476133045
CRYBB1	NIH	0.153213333	0.126920831	1.207156712	0.237471067	0.476734983
TPPP2	UCL	0.490203811	0.406489131	1.205945679	0.237592858	0.574166104
FCRL2	UCL	0.397300752	0.329108899	1.207201489	0.237845799	0.574166104
AZU1	NIH	0.43852	0.363611325	1.206013042	0.237904745	0.477278039
IL19	NIH	-0.355633333	0.295049056	-1.205336284	0.238161649	0.477465952
STAM	UCL	-0.132306783	0.110975277	-1.192218536	0.238203239	0.574166104
CCT5	UCL	-0.139557474	0.11669435	-1.195923147	0.23832564	0.574166104
NTRK3	NIH	-0.124693333	0.103491262	-1.204868232	0.238339447	0.477495125
GIMAP8	UCL	-0.21636659	0.180569071	-1.198248342	0.238444738	0.574166104
SMAD2	UCL	-0.140027762	0.117307708	-1.193679119	0.238466524	0.574166104
CX3CL1	NIH	-0.160013333	0.133116	-1.202059355	0.239408524	0.479139359
FGF12	NIH	0.883013333	0.734710846	1.201851502	0.239487775	0.479139359
CPB1	UCL	-0.267210241	0.222447409	-1.20122883	0.239853992	0.57654104
HPGDS	UCL	0.171775486	0.143228831	1.199308019	0.240035919	0.57654104
OGN	UCL	-0.223594532	0.186067041	-1.201688005	0.240044627	0.57654104
CYTH3	NIH	0.29964	0.249747626	1.199771163	0.240282056	0.480156029
PSIP1	NIH	0.421806667	0.351604943	1.199660799	0.240324248	0.480156029
MMP8	UCL	-0.541775833	0.451617445	-1.199634421	0.240369878	0.576848238
NCS1	UCL	-0.117241357	0.097781439	-1.199014443	0.240698378	0.577162724
SSC5D	NIH	0.252346667	0.210594108	1.19826081	0.240859939	0.480897831
TXNDC15	UCL	-0.118673244	0.0989852	-1.198898867	0.241056284	0.577547145
VWC2	UCL	-0.212914002	0.178144689	-1.195174571	0.242070032	0.579237127
FHIP2A	UCL	-0.182447404	0.153041114	-1.19214634	0.242157978	0.579237127
MRPL28	UCL	-0.10499324	0.088864335	-1.181500317	0.242962535	0.580686419
CASC3	NIH	0.186526667	0.156414736	1.192513385	0.243068422	0.484709291
ADA	NIH	0.136073333	0.114192229	1.191616408	0.243414441	0.484709291
TEX33	NIH	0.288346667	0.242027312	1.191380691	0.243505432	0.484709291
CCDC50	UCL	-0.332160179	0.278534622	-1.192527437	0.243547758	0.581609555
LPA	NIH	-0.661633333	0.555575549	-1.190897142	0.243692169	0.484709291
SNCG	NIH	0.47272	0.397074277	1.190507739	0.243842626	0.484709291

TRDMT1	NIH	0.280033333	0.235318972	1.190015964	0.244032736	0.484709291
PLA2G7	NIH	0.174833333	0.146948168	1.189761914	0.244130989	0.484709291
SFTPD	NIH	0.31006	0.260618493	1.189708363	0.244151703	0.484709291
LBP	NIH	0.289886667	0.243773892	1.189162074	0.244363092	0.484709291
MFGE8	UCL	-0.289963218	0.243501009	-1.190809102	0.244407473	0.583093986
DIPK2B	NIH	0.138706667	0.116658312	1.188999432	0.244426053	0.484709291
IL25	UCL	-0.197699122	0.167805925	-1.178141488	0.24456833	0.583093986
CTNNA1	NIH	-0.270526667	0.227915608	-1.186959808	0.245216639	0.485947609
CRISP3	UCL	0.181249779	0.152581751	1.187886344	0.245592223	0.584473666
IL15RA	UCL	-0.167917555	0.142471625	-1.178603488	0.24575578	0.584473666
CDH17	UCL	0.324748327	0.274020097	1.185125948	0.245960282	0.584473666
BST1	UCL	-0.234885604	0.198144375	-1.185426559	0.246215683	0.584473666
SAT2	NIH	0.233413333	0.197075433	1.184385745	0.24621708	0.487599837
ICAM1	UCL	-0.147678949	0.124628431	-1.184953931	0.246424148	0.584473666
NOS3	UCL	-0.249958493	0.211595498	-1.181303454	0.246517047	0.584473666
DEFB118	NIH	-0.459986667	0.388712472	-1.183359679	0.246616712	0.488060814
CLU	UCL	0.075228867	0.063792452	1.179275365	0.246667825	0.584473666
CSH1	UCL	0.128256757	0.109213514	1.1743671	0.246746666	0.584473666
STK24	NIH	0.32214	0.272339651	1.182861176	0.246811042	0.488115144
SMAD2	NIH	0.10378	0.087836449	1.181514061	0.24733675	0.48882432
FAM171A2	UCL	-0.784142596	0.66281154	-1.183055135	0.24741912	0.585591974
ACVRL1	NIH	0.104486667	0.088569077	1.179719497	0.248038356	0.48987994
TP53INP1	NIH	0.17384	0.147541583	1.178244103	0.248616278	0.490690022
GABARAP	UCL	-0.236038567	0.201438269	-1.17176626	0.249101439	0.587009729
ACADM	UCL	-0.283758295	0.241640666	-1.174298596	0.249238914	0.587009729
SFTPA1	UCL	-0.458425745	0.388161773	-1.181017238	0.249322788	0.587009729
F11	UCL	0.149051521	0.12671882	1.176238236	0.249642925	0.587009729
TNFRSF10B	UCL	-0.139920375	0.118832031	-1.177463471	0.24964453	0.587009729
FMNL1	UCL	-0.237117121	0.203115831	-1.167398524	0.249655146	0.587009729
TCN1	UCL	0.167784459	0.14264356	1.176249801	0.249701439	0.587009729
LCAT	UCL	0.084840946	0.072333915	1.172906862	0.249831315	0.587009729
FGA	UCL	-0.174376623	0.148107232	-1.177367379	0.249960472	0.587009729
HJV	UCL	-0.159879518	0.13601484	-1.175456431	0.250026381	0.587009729

BMP6	NIH	0.233293333	0.198633246	1.174492881	0.250090127	0.493266097
EPHB4	NIH	0.12226	0.104147302	1.173914237	0.250318047	0.493382943
MELTF	UCL	0.149339273	0.127219223	1.173873486	0.250430632	0.587486948
GIMAP8	NIH	0.198153333	0.168903816	1.173172622	0.250610382	0.493607234
PNMA1	NIH	0.30794	0.262575017	1.172769609	0.25076935	0.493607234
GZMA	UCL	0.198660854	0.169250941	1.17376514	0.250786124	0.587794339
MYO6	UCL	-0.143057974	0.121746065	-1.175052137	0.250963851	0.587794339
RELB	UCL	0.254197489	0.21749297	1.16876186	0.251231572	0.587911008
CD300C	UCL	-0.206275479	0.175962013	-1.17227278	0.25142721	0.587911008
VIPR1	UCL	-0.086188643	0.073683938	-1.169707339	0.251652483	0.587911008
VSNL1	NIH	0.18384	0.157094186	1.170253367	0.251763558	0.495122971
ZBTB17	UCL	-0.180896025	0.154281376	-1.172507203	0.251818194	0.587911008
NRP2	NIH	-0.1372	0.117268531	-1.169964341	0.251877942	0.495122971
SELP	UCL	-0.224015264	0.191453396	-1.170077258	0.252287498	0.588536597
IGF1	NIH	-0.25276452	0.21610046	-1.169662106	0.252359111	0.499999308
CD40	UCL	-0.218809633	0.187939799	-1.164253841	0.252634444	0.58871371
HEPACAM2	UCL	0.206426658	0.176566513	1.169115566	0.252766235	0.58871371
PDAP1	NIH	0.26772	0.229670825	1.165668299	0.253582637	0.497598361
CARHSP1	NIH	0.392526667	0.33674312	1.165656084	0.253587496	0.497598361
S100G	UCL	-0.250907745	0.214163801	-1.171569349	0.253622805	0.5897758
AIDA	UCL	-0.185438986	0.160776198	-1.153398256	0.253646167	0.5897758
SMNDC1	NIH	0.388566667	0.333389084	1.165505068	0.253647575	0.497598361
LRP1	NIH	-0.101413333	0.087053566	-1.164953232	0.2538672	0.497695416
RAB10	UCL	-0.220976818	0.190985405	-1.157035102	0.254096943	0.5897758
RAB6B	NIH	0.28746	0.246900571	1.164274343	0.254137583	0.497891782
TIE1	UCL	-0.197103955	0.170725691	-1.154506704	0.254401053	0.5897758
FEN1	NIH	0.35114	0.301820235	1.16340775	0.25448303	0.498234848
CCL16	UCL	-0.227785386	0.195633416	-1.164348049	0.25451942	0.5897758
LRRN1	UCL	-0.193866894	0.166630571	-1.163453339	0.254631239	0.5897758
EDAR	UCL	-0.317245262	0.273685197	-1.1591612	0.254634642	0.5897758
TNIP1	NIH	0.256333333	0.220818884	1.160830669	0.255512357	0.499915482
CA11	UCL	0.085317743	0.073860382	1.155121883	0.255597065	0.591536201
CEACAM5	UCL	-0.352856606	0.3047573	-1.157828232	0.256068489	0.59215838

CD3D	NIH	0.185933333	0.160474999	1.158643618	0.25638829	0.501135979
TDGF1	NIH	1.310466667	1.131255555	1.158417884	0.256478824	0.501135979
SNAPIN	UCL	-0.212411539	0.184079991	-1.153908894	0.256938527	0.593455612
ATXN2	UCL	-0.523819803	0.451930068	-1.159072699	0.257035513	0.593455612
CNTN2	NIH	-0.211306667	0.182681765	-1.156692712	0.257171499	0.502153962
HRAS	UCL	-0.316329242	0.27280146	-1.159558463	0.25729142	0.593577602
DYNC1H1	NIH	0.13362	0.1156389	1.155493519	0.257653794	0.502760071
LILRB4	UCL	-0.203913729	0.176347129	-1.156320094	0.257803341	0.593854533
FCRL1	UCL	0.31607553	0.273324843	1.156409813	0.257817791	0.593854533
SDC1	NIH	0.132126667	0.11439012	1.15505313	0.257831077	0.5027706
PARP1	UCL	-0.166953141	0.145207063	-1.149759092	0.258166724	0.594190028
FLRT2	UCL	0.169607787	0.146994424	1.153838234	0.258451672	0.594377841
MMP12	NIH	0.36126	0.313425831	1.152617187	0.2588133	0.503847933
ARSB	NIH	0.297793333	0.258391913	1.152487049	0.258865851	0.503847933
CLINT1	NIH	0.157526667	0.136694234	1.152401694	0.258900323	0.503847933
GRIN2B	NIH	-0.143313333	0.124468323	-1.151404068	0.259303473	0.504296981
NUP50	UCL	-0.098576837	0.086385598	-1.141125835	0.259616349	0.59658694
BATF	UCL	-0.467942979	0.406878085	-1.15008155	0.260276714	0.597439187
SSC5D	UCL	-0.201389089	0.175165079	-1.149710266	0.260544734	0.597439187
ICAM4	UCL	0.260273623	0.22640337	1.149601364	0.260600398	0.597439187
PFKFB2	NIH	0.382993333	0.333748925	1.147549265	0.260865545	0.50699782
SLC39A14	UCL	0.208415886	0.182016593	1.145037841	0.260933321	0.597733619
HMOX2	NIH	0.16418	0.143176516	1.146696435	0.26121206	0.507334181
EBI3_IL27	UCL	0.150911107	0.1314157	1.14834914	0.261292361	0.59808737
AOC1	NIH	0.466113333	0.406799641	1.145805665	0.26157435	0.507647013
CD200R1	NIH	-0.12416	0.108394344	-1.145447219	0.261720238	0.507647013
SPINK2	UCL	0.190232668	0.166047765	1.145650273	0.261811395	0.598331905
SOWAHA	UCL	0.186095548	0.163061122	1.141262525	0.262071725	0.598331905
HSD17B3	UCL	-0.190572374	0.167351596	-1.138754449	0.262184444	0.598331905
AZU1	UCL	0.249179162	0.219717416	1.134089259	0.262217985	0.598331905
TFF1	NIH	0.32628	0.285192855	1.144067932	0.262282163	0.508168833
FYB1	NIH	0.248326667	0.217081268	1.143934107	0.262336731	0.508168833
VWC2L	NIH	0.22328	0.195420437	1.142562178	0.262896615	0.508828793

TLR2	NIH	-0.188126667	0.164764815	-1.141789083	0.263212498	0.508828793
PLXNB3	UCL	-0.146644464	0.128554454	-1.140718657	0.263235807	0.600185853
SCP2	NIH	0.300553333	0.263264612	1.141639703	0.263273566	0.508828793
UBQLN3	NIH	0.440046667	0.385533777	1.141395885	0.263373263	0.508828793
RNF168	NIH	0.1489	0.13053833	1.140661141	0.263673864	0.509073303
LTA	UCL	0.181737148	0.159307263	1.140796371	0.263815812	0.601039454
CSF2RB	NIH	-0.212153333	0.186204703	-1.139355396	0.264208693	0.509494038
CRYGD	NIH	0.32966	0.289358536	1.139278642	0.264240156	0.509494038
CELSR2	UCL	-0.123965179	0.109018104	-1.13710635	0.264370661	0.601834456
QSOX1	NIH	0.078126667	0.068680434	1.137538913	0.264954029	0.510487795
VAT1	NIH	-0.07748	0.068133908	-1.137172397	0.265104602	0.510487795
NOS2	NIH	-0.287226667	0.252739123	-1.136455107	0.265399461	0.510719358
NOMO1	NIH	0.1388	0.122223824	1.135621484	0.265742441	0.511043155
FOLR2	UCL	0.169293522	0.149697961	1.130900653	0.266478601	0.605508032
CRTAM	UCL	0.268588866	0.23691587	1.133688789	0.266615259	0.605508032
SSBP1	UCL	-0.162521933	0.144453001	-1.125085195	0.266732487	0.605508032
TXK	NIH	0.257786667	0.227508879	1.133083984	0.26678843	0.512603531
FGF3	UCL	0.113312913	0.100837146	1.123721939	0.266812982	0.605508032
PPP1R9B	NIH	0.27032	0.238629258	1.132803253	0.266904334	0.512603531
UPB1	UCL	0.403742588	0.352579883	1.145109541	0.267064945	0.605609646
SPRING1	UCL	-0.15295572	0.13554804	-1.128424433	0.26755333	0.606246809
IL17D	UCL	-0.555927294	0.491217273	-1.131734009	0.267906289	0.606576361
SERPINI1	UCL	0.089984291	0.07998552	1.12500726	0.268747144	0.607108992
TNFRSF13C	UCL	-0.216399801	0.192190136	-1.12596726	0.26915262	0.607108992
CD84	UCL	-0.145328328	0.129196214	-1.124865225	0.26971874	0.607108992
HYOU1	NIH	0.125686667	0.111622516	1.125997431	0.2697254	0.517681624
PRSS22	UCL	-0.129053224	0.115347959	-1.118816708	0.269846666	0.607108992
STX4	UCL	-0.166836845	0.148503695	-1.123452481	0.269867702	0.607108992
HTRA2	UCL	-0.224046682	0.199735297	-1.121718023	0.269884471	0.607108992
TNFRSF12A	UCL	0.150034699	0.13325946	1.125884034	0.269924536	0.607108992
SSB	UCL	-0.375369862	0.333577024	-1.125286922	0.269973827	0.607108992
IL1RN	UCL	-0.345968484	0.307048331	-1.126755788	0.270100404	0.607108992
ACOX1	NIH	0.288046667	0.256022105	1.125085145	0.270105183	0.518070597

TIGAR	UCL	-0.279159542	0.24932738	-1.119650567	0.270526254	0.607108992
CST7	UCL	-0.302135224	0.268765272	-1.12416021	0.270536367	0.607108992
CEP112	UCL	0.171079494	0.15260432	1.121065862	0.270633944	0.607108992
PEPD	UCL	0.113507911	0.101144309	1.122237254	0.271159493	0.607313582
COL18A1	UCL	-0.116652058	0.103944308	-1.122255377	0.271308869	0.607313582
IL2RG	UCL	-0.18311072	0.163717121	-1.118457976	0.271439143	0.607313582
SMPDL3A	NIH	0.22102	0.197033518	1.121738078	0.271501871	0.519855547
TIMD4	UCL	-0.267225418	0.237916154	-1.123191567	0.271562321	0.607313582
APOB	NIH	0.175693333	0.15668368	1.121325041	0.271674588	0.519855547
PFDN4	NIH	0.1561	0.139210799	1.121321055	0.271676255	0.519855547
NFASC	NIH	-0.116246667	0.103684962	-1.121152618	0.271746712	0.519855547
COL4A1	UCL	-0.179546211	0.15986775	-1.12309212	0.271763998	0.607313582
C9orf40	UCL	-0.313672029	0.281609964	-1.113852735	0.272113569	0.607630223
SMAD3	UCL	-0.185504562	0.167110872	-1.110068781	0.27234345	0.607679316
COL6A3	UCL	0.163343234	0.145843186	1.119992225	0.272719012	0.608013379
TXNDC9	NIH	0.06292	0.056266792	1.118243945	0.272965484	0.521845779
TYRO3	NIH	0.16248	0.145497349	1.116721376	0.273605031	0.522415275
EDNRB	NIH	0.476993333	0.427151431	1.116684386	0.273620582	0.522415275
RTKN2	UCL	-0.264143449	0.233345628	-1.131983708	0.273899001	0.608013379
IGFL4	UCL	0.391134925	0.350246572	1.116741621	0.274036625	0.608013379
HIF1A	NIH	0.493846667	0.442700533	1.115532126	0.274105319	0.522617067
IGFBP7	NIH	0.113253333	0.101548639	1.115261954	0.274219065	0.522617067
NFYA	NIH	0.181913333	0.163127664	1.115159308	0.27426229	0.522617067
DKK4	UCL	0.272654111	0.244589346	1.114742384	0.274404172	0.608013379
NXPH1	UCL	-0.207092282	0.187638254	-1.103678365	0.274452795	0.608013379
CBX2	UCL	0.170169836	0.153220849	1.110618018	0.274504711	0.608013379
LSM1	NIH	0.204633333	0.183597951	1.114573078	0.274509246	0.5227471
CD2	UCL	0.144217559	0.13072456	1.103217019	0.27465131	0.608013379
MAN2B2	UCL	-0.288746761	0.258925898	-1.115171418	0.274842014	0.608013379
ERN1	UCL	0.217879667	0.195221588	1.116063389	0.274869258	0.608013379
KRT6C	UCL	0.1848607	0.166903256	1.107591938	0.274918291	0.608013379
KLK8	UCL	-0.173126096	0.155465992	-1.113594641	0.27514298	0.608013379
AHSP	UCL	-0.347890765	0.313392157	-1.110081275	0.275169417	0.608013379

TYMP	UCL	-0.271937106	0.244968171	-1.11009159	0.275197297	0.608013379
FGL1	NIH	0.300313333	0.270054165	1.112048515	0.275574579	0.524434381
TGM2	NIH	0.373233333	0.335781511	1.111536286	0.275791095	0.524505172
LEO1	UCL	-0.108580509	0.098456421	-1.102828118	0.276006454	0.608946984
TGFB1	UCL	-0.140922081	0.126717119	-1.112099783	0.276161981	0.608946984
RABEPK	NIH	-0.24014	0.216241467	-1.110517811	0.276221963	0.524667308
CLEC6A	NIH	0.251413333	0.226399212	1.110486785	0.276235096	0.524667308
LILRA6	UCL	0.408223083	0.367273409	1.111496431	0.276270551	0.608946984
TFPI	UCL	-0.141173957	0.126989118	-1.111701221	0.276453181	0.608946984
ID4	UCL	0.13369563	0.120765191	1.107070912	0.277013041	0.609405709
CSF2RB	UCL	-0.199442997	0.180252957	-1.106461724	0.277078408	0.609405709
ZHX2	NIH	0.25858	0.233340097	1.108167879	0.27721795	0.525891942
LYN	NIH	0.158633333	0.143203799	1.10774529	0.277397333	0.525891942
MFGE8	NIH	0.300626667	0.271398732	1.107693702	0.277419236	0.525891942
BPIFA2	NIH	-0.698526667	0.630944119	-1.107113365	0.27766573	0.526018303
ADAM9	UCL	-0.14739299	0.133047085	-1.107825774	0.277732568	0.610385184
PAFAH2	NIH	0.22336	0.201901695	1.106280956	0.278019563	0.526347717
PGLYRP2	UCL	0.126352657	0.114214261	1.106277407	0.278268206	0.611102904
CRNN	NIH	0.256646667	0.232311977	1.104750043	0.278671155	0.527240057
KIR3DL1	NIH	0.541786667	0.490821817	1.103835746	0.279060823	0.527580644
C7orf50	NIH	0.13572	0.122992548	1.103481489	0.27921191	0.527580644
INSR	UCL	-0.082276443	0.075141607	-1.094951864	0.279589757	0.612780444
CD72	UCL	0.187038608	0.169713453	1.102084743	0.279617792	0.612780444
MBL2	UCL	-0.490452067	0.443986024	-1.104656543	0.279661003	0.612780444
DNAJB8	UCL	-0.180341483	0.163582477	-1.102449885	0.28042595	0.613544524
FLT4	UCL	-0.168297908	0.152878485	-1.100860648	0.280429519	0.613544524
MTDH	NIH	0.341613333	0.310767175	1.099258095	0.281017665	0.530520451
EIF4B	NIH	0.36714	0.3340689	1.098994847	0.281130495	0.530520451
MCAM	UCL	0.104270863	0.095252443	1.094679145	0.281865559	0.616225152
AGER	UCL	0.158496089	0.144356693	1.09794763	0.282259276	0.616624711
HSD11B1	UCL	-0.167200147	0.152687574	-1.095047507	0.282777696	0.617295897
FCGR3B	UCL	0.254652952	0.232511342	1.095228085	0.283320058	0.617747505
RNASE6	UCL	-0.209080832	0.190943559	-1.094987615	0.283499944	0.617747505

OSBPL2	UCL	-0.07009149	0.0644725	-1.087153289	0.283618594	0.617747505
LBP	UCL	-0.274946336	0.251138564	-1.094799346	0.283850885	0.617793104
PTPRM	NIH	-0.110666667	0.101288264	-1.092591208	0.283885142	0.534722212
NTproBNP	NIH	-0.613	0.561072437	-1.092550551	0.283902693	0.534722212
CDON	NIH	0.09694	0.088765044	1.092096575	0.284098717	0.534722212
GID8	UCL	-0.159055508	0.146168929	-1.088162234	0.284237202	0.618173617
ASRGL1	NIH	0.292673333	0.268267048	1.090977576	0.284582306	0.534722212
KLRC1	UCL	-0.195571463	0.180433207	-1.083899503	0.28462208	0.618516758
LHB	NIH	0.4338	0.397699	1.090774682	0.284670052	0.534722212
LPA	UCL	-0.688086045	0.630876782	-1.09068215	0.284956263	0.618516758
IL7R	NIH	-0.240013333	0.22017365	-1.090109255	0.284957966	0.534722212
PRSS53	UCL	0.161863552	0.14841019	1.090649848	0.285029789	0.618516758
SNX9	NIH	0.228133333	0.209392116	1.089502976	0.285220469	0.534722212
CELA2A	NIH	-0.330506667	0.303433676	-1.089222104	0.285342138	0.534722212
FCRL3	NIH	0.527853333	0.484634485	1.089178236	0.285361144	0.534722212
EIF1AX	NIH	0.20632	0.189458948	1.088995807	0.285440192	0.534722212
GALNT5	NIH	0.24166	0.221932456	1.088889856	0.285486109	0.534722212
TJP3	NIH	-0.392993333	0.361035737	-1.088516436	0.285647984	0.534722212
PCSK7	NIH	0.14026	0.128877659	1.088318962	0.285733613	0.534722212
PPM1B	UCL	0.35592901	0.32728226	1.087529188	0.286030604	0.619772256
SIRPA	UCL	-0.295885865	0.271942837	-1.088044342	0.286161142	0.619772256
FGF9	NIH	-0.200773333	0.184678099	-1.08715291	0.286239615	0.535326645
YAP1	UCL	0.111329954	0.10229971	1.088272426	0.286430003	0.619772256
EPN1	UCL	-0.611498174	0.562028759	-1.088019365	0.286574535	0.619772256
ITIH3	UCL	0.227062879	0.208992339	1.086465082	0.286668522	0.619772256
PGR	NIH	0.298833333	0.275263169	1.085627746	0.286902414	0.536223361
LUZP2	NIH	-0.211513333	0.194918936	-1.085134867	0.287116841	0.536281455
AGXT	UCL	0.376870745	0.346917749	1.086340338	0.287386112	0.620447709
WARS	UCL	-0.158381178	0.145843387	-1.085967503	0.287405473	0.620447709
DAB2	NIH	0.250286667	0.230903918	1.083942916	0.287635869	0.536834665
ATP1B4	NIH	0.233506667	0.215489187	1.083611991	0.287780087	0.536834665
ADAMTSL5	UCL	-0.230385767	0.212370639	-1.084828712	0.288026882	0.621265018
TFF3	UCL	-0.111091939	0.102527497	-1.083533118	0.288209156	0.621265018

DDA1	NIH	0.271913333	0.251208012	1.082423015	0.288298669	0.537149955
AP3S2	NIH	0.257886667	0.238313901	1.082130189	0.28842649	0.537149955
BATF	NIH	0.571626667	0.528324277	1.081961763	0.288500027	0.537149955
LCP1	UCL	-0.170213411	0.157905443	-1.077945176	0.288775589	0.621542804
DUSP3	UCL	-0.548380893	0.50730111	-1.08097712	0.288912669	0.621542804
HSP90B1	UCL	0.728422381	0.673441818	1.081641148	0.28897594	0.621542804
SDK2	NIH	0.277786667	0.257050916	1.080667875	0.289065402	0.53752867
IPCEF1	NIH	0.333786667	0.308881978	1.080628493	0.289082623	0.53752867
MFAP3L	NIH	-0.31176	0.288603879	-1.080234959	0.289254744	0.53752867
NCLN	NIH	0.536373333	0.496742829	1.079780727	0.289453504	0.537556507
INHBC	UCL	0.253058321	0.234053759	1.081197422	0.289815536	0.622633129
CDHR1	NIH	0.18578	0.172216223	1.078760161	0.289900429	0.538044895
SERPINA9	UCL	0.347052503	0.321350934	1.079979754	0.289908891	0.622633129
CA8	UCL	0.099056945	0.092258979	1.073683515	0.290600815	0.623535587
CD248	UCL	0.180052998	0.166902577	1.078791005	0.290755732	0.623535587
KLK1	NIH	-0.621333333	0.577314597	-1.076247399	0.291002902	0.539748566
CPE	NIH	-0.141953333	0.132098767	-1.074599987	0.291727315	0.5407493
NAMPT	UCL	-0.24699072	0.230552055	-1.071301316	0.29192803	0.62559064
LGALS8	NIH	0.158946667	0.147985794	1.074067061	0.29196193	0.540841448
CTF1	NIH	0.543733333	0.506524545	1.073459003	0.292229785	0.540995014
CNTN3	NIH	0.21054	0.196448214	1.071732828	0.292991129	0.542061386
NOTCH3	UCL	0.1109256	0.103527149	1.071463872	0.293055976	0.627127968
CD3G	UCL	0.24017337	0.224030679	1.072055713	0.293074514	0.627127968
IL3	UCL	0.296127462	0.278704685	1.062513399	0.293628524	0.627584457
UXS1	UCL	0.306251817	0.286089319	1.070476232	0.293783094	0.627584457
DOK1	NIH	0.327573333	0.306169037	1.069910062	0.293796597	0.543207994
LRIG1	UCL	0.175198203	0.163628324	1.07070829	0.293931961	0.627584457
GMPR	NIH	0.306873333	0.287353347	1.067930256	0.294673233	0.544484653
NGFR	UCL	0.128345984	0.120466525	1.065407872	0.295959176	0.631451586
IGHMBP2	NIH	0.225106667	0.211502684	1.064320614	0.296276294	0.547101111
HEBP1	NIH	0.31586	0.296969599	1.063610554	0.296592359	0.547339211
TRIM26	UCL	0.210874282	0.199497926	1.057024937	0.296747986	0.631679115
NTF4	UCL	-0.146331638	0.13681002	-1.069597371	0.296773057	0.631679115

IGSF3	NIH	0.274586667	0.258367448	1.062775784	0.296964238	0.547460621
EIF1AX	UCL	0.257920485	0.243652365	1.058559329	0.297002338	0.631679115
CETN3	NIH	0.155853333	0.146668556	1.062622678	0.297032481	0.547460621
ACY1	UCL	0.282085634	0.265312035	1.06322216	0.297213313	0.631679115
NBN	UCL	-0.165519067	0.156387494	-1.058390682	0.297293441	0.631679115
TIMP3	UCL	0.529831094	0.499987465	1.059688755	0.297446535	0.631679115
MYO9B	NIH	0.254386667	0.239650367	1.061490827	0.297537313	0.548045743
PCSK7	UCL	-0.141153198	0.134025093	-1.053184853	0.297594525	0.631679115
CCL24	UCL	-0.359269551	0.338251872	-1.062136179	0.297819556	0.631679115
CEACAM8	UCL	-0.172057385	0.162384522	-1.059567647	0.298339793	0.631679115
NCR1	UCL	-0.163623769	0.154447211	-1.059415502	0.298824206	0.631679115
GOLGA3	UCL	-0.403709159	0.377547211	-1.069294508	0.298826536	0.631679115
NFATC1	NIH	0.292146667	0.276018619	1.058431012	0.298905087	0.550013854
ADAMTS13	UCL	0.080261204	0.075879142	1.057750541	0.298933218	0.631679115
POF1B	NIH	-0.157433333	0.148766285	-1.058259492	0.29898189	0.550013854
ENTR1	UCL	-0.273045751	0.257970739	-1.058436906	0.299072701	0.631679115
GLI2	UCL	0.2492045	0.235602249	1.057733958	0.299091309	0.631679115
EXOSC10	NIH	0.32062	0.303610574	1.056023827	0.299984234	0.551401991
PILRA	UCL	-0.256139373	0.242210843	-1.057505806	0.300077478	0.63330431
NEK7	NIH	-0.161793333	0.153266985	-1.05563069	0.300160738	0.551401991
KEL	NIH	-0.151713333	0.143761027	-1.055316149	0.300302008	0.551401991
ENTPD2	UCL	0.226666983	0.214587185	1.056293194	0.300624645	0.633945482
LGALS7_LGALS7B	UCL	-0.153156044	0.145466746	-1.052859494	0.30083938	0.633945482
DBI	UCL	-0.254931678	0.242872528	-1.04965218	0.301031929	0.633945482
HAO1	UCL	0.567538812	0.537972858	1.054958077	0.301496053	0.634135943
FGFR2	UCL	-0.082451369	0.078322352	-1.052718254	0.301556264	0.634135943
LBR	NIH	0.27404	0.260452912	1.052167159	0.30171889	0.553656055
CXCL16	UCL	-0.13179879	0.125094968	-1.053589858	0.30202294	0.634293203
LY96	UCL	0.194916841	0.185233804	1.052274676	0.302065049	0.634293203
TPBGL	NIH	-0.161726667	0.153957994	-1.050459687	0.302489122	0.554139672
PTK7	NIH	0.231026667	0.219929797	1.050456417	0.302490598	0.554139672
ANXA11	NIH	0.394893333	0.376315577	1.049367492	0.302982529	0.554139672
CRIM1	UCL	-0.096837858	0.092166706	-1.050681548	0.303074025	0.635725268

RNASE3	NIH	0.45268	0.43152885	1.049014452	0.303142137	0.554139672
CPA2	NIH	-0.225546667	0.215058226	-1.048770236	0.303252581	0.554139672
CSDE1	UCL	-0.344085034	0.328655727	-1.046946715	0.303268799	0.635725268
SCARB1	UCL	0.465961293	0.444896013	1.04734877	0.303469537	0.635725268
HAVCR1	NIH	0.26396	0.251842546	1.048115198	0.303548955	0.554139672
MXRA8	NIH	-0.18846	0.179812801	-1.048090005	0.303560357	0.554139672
CGN	UCL	-0.136322038	0.130854338	-1.041784631	0.303616994	0.635725268
SCG3	NIH	-0.160826667	0.153478626	-1.047876639	0.303656941	0.554139672
SBSN	NIH	0.170406667	0.162631394	1.047809174	0.303687485	0.554139672
ADGRE2	UCL	0.153917472	0.146801838	1.048471018	0.303841824	0.635740624
APOM	UCL	0.134250119	0.128538663	1.044433761	0.304285336	0.63582551
CLSTN3	UCL	0.295983975	0.282781875	1.046686511	0.304317444	0.63582551
NPDC1	UCL	-0.109510897	0.105043454	-1.042529468	0.304967947	0.636729506
CDNF	NIH	-0.116466667	0.111465038	-1.044871722	0.305019461	0.55600527
BRSK2	NIH	0.277473333	0.265596838	1.044716255	0.305090071	0.55600527
MANF	NIH	0.304053333	0.291178356	1.044216808	0.305316986	0.556072343
LMOD1	NIH	0.16982	0.162808638	1.043065051	0.305840716	0.556359722
CREBZF	UCL	-0.070936779	0.068643804	-1.033403966	0.305857221	0.638130375
PSG1	UCL	-0.604058935	0.578479	-1.044219297	0.306112245	0.638206913
TREH	NIH	0.401673333	0.385373196	1.042297018	0.306190307	0.556359722
MUC16	NIH	0.750986667	0.720741553	1.041963882	0.30634203	0.556359722
KCNC4	NIH	-0.257493333	0.247370198	-1.040923018	0.306816417	0.556359722
CYTL1	NIH	-0.148	0.142204661	-1.040753508	0.306893722	0.556359722
TST	NIH	0.184793333	0.177569466	1.040681925	0.306926372	0.556359722
CLEC12A	NIH	0.227826667	0.218939402	1.040592348	0.306967231	0.556359722
ARHGAP25	NIH	0.246066667	0.236482432	1.040528316	0.306996442	0.556359722
ESAM	UCL	-0.108186194	0.103875903	-1.041494612	0.307392875	0.640420081
AMBN	NIH	0.16822	0.161914134	1.038945745	0.307718997	0.556928359
GUSB	UCL	-0.336253042	0.323392067	-1.039768986	0.307937106	0.641096981
RABEP1	NIH	0.244006667	0.234980102	1.038414164	0.307961967	0.556928359
DDR1	NIH	0.10164	0.097888408	1.038325192	0.308002647	0.556928359
CLEC2L	NIH	0.233586667	0.225026435	1.038041004	0.308132608	0.556928359
ECI2	NIH	0.24624	0.237280834	1.037757646	0.308262227	0.556928359

CLINT1	UCL	0.117000774	0.113841919	1.027747727	0.308487367	0.641288643
EPHA2	UCL	-0.131963219	0.127020848	-1.038909919	0.308523487	0.641288643
IKZF2	NIH	0.34504	0.33275378	1.036922858	0.308644314	0.556990122
RAB44	NIH	0.324433333	0.312947629	1.036701683	0.308745602	0.556990122
RNF168	UCL	0.213818122	0.206476612	1.03555613	0.30898594	0.641288643
ACTN4	NIH	0.098866667	0.095427786	1.036036469	0.30905038	0.556990122
CD72	NIH	0.232386667	0.224307209	1.036019606	0.309058109	0.556990122
BLOC1S2	UCL	0.366846444	0.353442556	1.037923809	0.309116775	0.641288643
RCOR1	UCL	0.26583012	0.256723744	1.035471499	0.309629626	0.641288643
GAMT	NIH	0.135133333	0.130598657	1.034722227	0.309653136	0.557718856
IGLON5	UCL	-0.263108817	0.254320381	-1.034556556	0.309878898	0.641288643
SCARF1	UCL	-0.154125541	0.149329078	-1.032120085	0.310037593	0.641288643
DGCR6	UCL	0.185542861	0.180074786	1.030365577	0.310059791	0.641288643
KRT17	UCL	0.099658504	0.097300347	1.024235848	0.310128096	0.641288643
GABARAP	NIH	0.253126667	0.244932393	1.033455246	0.310234992	0.558422985
RBM25	UCL	-0.325083503	0.317489835	-1.02391783	0.310276963	0.641288643
LYPLA2	UCL	-0.422343673	0.408699463	-1.033384458	0.310645091	0.641288643
MANEAL	UCL	0.178285751	0.173599367	1.026995396	0.310661895	0.641288643
BCAM	NIH	-0.100073333	0.096941695	-1.032304353	0.310764193	0.55888198
HIP1	NIH	0.15114	0.14646039	1.031951372	0.310926626	0.55888198
TINAGL1	NIH	-0.102733333	0.099593403	-1.031527492	0.311121762	0.55888198
CRISP2	UCL	0.295857512	0.286455367	1.032822373	0.311524466	0.642404597
NEFL	NIH	-0.22524	0.218567026	-1.03053056	0.311581045	0.55888198
ANKMY2	UCL	-0.471904581	0.459417791	-1.027179596	0.311642052	0.642404597
ARHGAP5	NIH	0.305813333	0.29688129	1.030086245	0.31178589	0.55888198
NPHS1	NIH	0.144546667	0.140346652	1.029926004	0.31185979	0.55888198
CD5	NIH	0.161766667	0.15710774	1.029654339	0.311985104	0.55888198
LETM1	UCL	-0.23880835	0.232825242	-1.025697848	0.312002569	0.642617974
TSPAN15	NIH	0.30972	0.300821158	1.029581835	0.312018555	0.55888198
KITLG	UCL	-0.114297116	0.111150234	-1.028311965	0.312287982	0.642617974
TMPRSS15	UCL	-0.430074172	0.418425865	-1.027838399	0.312405111	0.642617974
SIT1	NIH	0.228773333	0.222393753	1.028685971	0.31243208	0.559203487
PPP1CC	NIH	0.259466667	0.252313571	1.028350023	0.31258725	0.559203487

CHGB	UCL	-0.149434722	0.145377738	-1.027906507	0.312640229	0.642649361
RLN2	NIH	0.18704	0.181965347	1.027888019	0.31280073	0.559203487
ANK2	NIH	-0.452106667	0.440015904	-1.027478014	0.312990268	0.559203487
RBM19	UCL	0.333178246	0.324496351	1.026754983	0.313128454	0.643200613
DRG2	NIH	0.143973333	0.140179952	1.027060796	0.313183222	0.559203487
SIGLEC6	NIH	-0.183886667	0.179102684	-1.026710838	0.313345133	0.559203487
TNFRSF13B	NIH	0.166353333	0.162216543	1.025501655	0.31390502	0.559861088
CD209	UCL	-0.151589581	0.14779896	-1.025647143	0.314220136	0.644989788
ICAM2	NIH	-0.117853333	0.115070678	-1.024182137	0.314516787	0.560610361
PKLR	UCL	-0.192874839	0.189232517	-1.019247866	0.314897224	0.645926025
SOX2	UCL	-0.089433999	0.088270937	-1.013176047	0.315333771	0.645947656
FZD8	UCL	-0.151981141	0.150010647	-1.013135693	0.315352872	0.645947656
CD226	UCL	-0.184486211	0.18120856	-1.018087728	0.315570733	0.645947656
OSBPL2	NIH	0.090486667	0.088631542	1.020930751	0.316027749	0.562672898
NDST1	UCL	-0.631750776	0.617714853	-1.022722332	0.316064664	0.646312749
LTA4H	NIH	-0.143393333	0.14048282	-1.02071793	0.316126824	0.562672898
SMPDL3B	UCL	0.367943484	0.360429873	1.020846248	0.316191321	0.646312749
TOP2B	NIH	0.3181	0.311724851	1.020451207	0.316251024	0.562672898
PTTG1	UCL	0.185119087	0.182438211	1.014694704	0.316720002	0.646940996
GP2	UCL	-0.391086049	0.384055863	-1.018305113	0.317146386	0.647100441
MAEA	UCL	0.188944593	0.186458645	1.013332435	0.317240825	0.647100441
COL15A1	UCL	-0.121290111	0.119356977	-1.01619624	0.318331253	0.648871864
SMAD5	UCL	0.096244741	0.094738855	1.015895131	0.318751804	0.649276323
CCNE1	NIH	0.20466	0.20191797	1.013579922	0.31946227	0.567980442
KIAA1549	NIH	0.27208	0.268629762	1.012843843	0.3198076	0.567980442
CDCP1	NIH	-0.323706667	0.319607857	-1.012824497	0.31981668	0.567980442
EPB41L5	NIH	-0.34448	0.340537625	-1.011576915	0.320402581	0.568620488
MTSS2	NIH	0.311753333	0.30829138	1.011229486	0.320565875	0.568620488
CEBPB	NIH	0.41138	0.407205085	1.010252611	0.321025321	0.569090342
PLXNB2	UCL	0.125194533	0.123956357	1.009988809	0.321116645	0.653213818
HEG1	UCL	-0.128252587	0.126854731	-1.011019347	0.321154722	0.653213818
CES2	UCL	0.243768029	0.241994364	1.007329365	0.321508276	0.653213818
ADGRF5	UCL	-0.108271729	0.10774322	-1.004905259	0.321578749	0.653213818

GIP	NIH	-0.188646667	0.186989286	-1.008863507	0.321679429	0.569414113
COCH	NIH	0.1781	0.176539665	1.008838439	0.321691241	0.569414113
FHIP2A	NIH	-0.18072	0.179190869	-1.00853353	0.321834944	0.569414113
ADAMTS1	NIH	0.140993333	0.139844962	1.008211749	0.321986647	0.569414113
COX5B	UCL	-0.257967395	0.25656685	-1.005458791	0.322134196	0.653887678
SERPINB5	UCL	0.174569787	0.174853671	0.998376448	0.32239144	0.653955711
HPGDS	NIH	-0.146786667	0.145743942	-1.007154495	0.322485433	0.569951596
ST3GAL1	UCL	0.259303562	0.25705356	1.008753046	0.322697685	0.654122977
EPGN	NIH	0.10846	0.107801913	1.006104592	0.322981277	0.570482748
PPP2R5A	NIH	0.376333333	0.374311594	1.005401219	0.323313756	0.570482748
PNMA2	NIH	-0.175766667	0.174843492	-1.005280007	0.323371076	0.570482748
LIFR	UCL	0.103158925	0.102789341	1.003595547	0.323693066	0.655685955
TGFB2	NIH	0.21616	0.21527609	1.004105939	0.32392664	0.570580954
CD40	NIH	0.113766667	0.113312561	1.00400755	0.323973227	0.570580954
LRP2BP	NIH	-0.47224	0.470393344	-1.00392577	0.324011954	0.570580954
GH2	UCL	0.859163813	0.856192168	1.003470768	0.324534799	0.656925298
CR1	UCL	0.167969427	0.167437301	1.003178063	0.324754381	0.656925298
PRKAR2A	UCL	-0.240261494	0.240898881	-0.99735413	0.325370145	0.657643859
AREG	NIH	0.144213333	0.144121639	1.000636228	0.325572323	0.572621446
ALDH5A1	UCL	-0.238531611	0.238174034	-1.001501326	0.32572218	0.657643859
ADGRE5	UCL	0.151002582	0.150869755	1.000880407	0.325784574	0.657643859
MIA	NIH	-0.137213333	0.137201411	-1.000086894	0.325833397	0.572621446
L3HYPDH	NIH	0.1501	0.150112341	0.999917791	0.325913793	0.572621446
PBLD	NIH	0.2805	0.280640885	0.999497989	0.326113436	0.572621446
ERBIN	NIH	0.248693333	0.248837125	0.999422144	0.326149514	0.572621446
ST8SIA1	UCL	0.279369689	0.279634013	0.999054748	0.326152973	0.657933154
SCN3A	UCL	0.173592429	0.174689227	0.993721435	0.326404199	0.657985844
FGFR2	NIH	0.096186667	0.09644139	0.997358774	0.327132075	0.573878369
PSIP1	UCL	0.290503741	0.291221023	0.997536983	0.327232496	0.659200955
PDCL2	NIH	0.181166667	0.181759878	0.996736289	0.327428895	0.573878369
MILR1	NIH	0.191406667	0.192043556	0.996683622	0.327454017	0.573878369
SHMT1	NIH	0.25608	0.257132453	0.995906961	0.327824631	0.574183859
IL17C	UCL	-0.254167055	0.256221863	-0.991980359	0.327967622	0.659925283

TINAGL1	UCL	0.073588861	0.073960045	0.994981297	0.328043598	0.659925283
SLC9A3R1	NIH	0.224633333	0.225971735	0.99407713	0.328698939	0.575370674
GSTA1	UCL	0.348085466	0.349580212	0.995724169	0.328736254	0.660863873
IGF1R	NIH	0.07824	0.078745846	0.993576217	0.328938557	0.575445741
ECHDC3	UCL	0.402438816	0.405450745	0.992571406	0.329010867	0.66096135
ITIH3	NIH	0.173233333	0.174486277	0.992819244	0.329300891	0.575735269
PAM	UCL	0.077859931	0.078446831	0.99251849	0.329382455	0.661253377
RNF5	NIH	0.158	0.159275327	0.991992943	0.32969672	0.575743574
FOXJ3	NIH	0.166326667	0.167673044	0.991970219	0.32970761	0.575743574
NUBP1	NIH	-0.26378	0.266058104	-0.99143757	0.329962947	0.575743574
SCRN1	UCL	-0.162752662	0.165369636	-0.984175003	0.329971956	0.661982173
ABRAXAS2	NIH	0.18288	0.184509883	0.991166417	0.330092983	0.575743574
SSBP1	NIH	0.172793333	0.174455921	0.990469871	0.330427181	0.575971651
SRPK2	NIH	0.23124	0.233601267	0.989891891	0.330704667	0.575971651
CLEC7A	NIH	-0.174106667	0.175932244	-0.989623409	0.330833618	0.575971651
LRPAP1	UCL	0.348802752	0.352426981	0.989716369	0.330957635	0.66350423
IL21R	NIH	0.18912	0.191229855	0.988966915	0.331149076	0.575971651
TPSG1	NIH	0.15724	0.159014011	0.988843683	0.331208314	0.575971651
SIL1	UCL	0.090192978	0.091233337	0.988596728	0.331283453	0.663592585
GPIHBP1	UCL	-0.243784987	0.248225596	-0.982110592	0.331517689	0.663592585
VCAN	UCL	-0.08509156	0.086112075	-0.988148992	0.33168278	0.663592585
CLEC11A	NIH	0.172753333	0.175095548	0.986623219	0.332276936	0.577260955
IST1	NIH	0.360293333	0.365230095	0.986483146	0.332344427	0.577260955
ITGB2	NIH	-0.09156	0.092885073	-0.985734279	0.332705406	0.577544992
RNASE1	UCL	-0.120260785	0.121917656	-0.986409921	0.332747606	0.665116191
BABAM1	UCL	-0.106261537	0.108373918	-0.980508397	0.332987234	0.665116191
LCN2	NIH	0.202353333	0.205502173	0.98467734	0.33321534	0.578087112
C2	UCL	0.145825096	0.147797149	0.986657028	0.33328768	0.665116191
PLIN1	UCL	0.126003746	0.128102902	0.983613513	0.333354505	0.665116191
PADI4	NIH	0.369773333	0.375763936	0.984057538	0.33351462	0.578263344
IGSF21	NIH	0.333133333	0.338857991	0.983106027	0.333974424	0.57871753
PRKCQ	UCL	0.192422885	0.196293472	0.980281634	0.334012398	0.665695874
CCND2	UCL	-0.23224947	0.237961589	-0.975995628	0.334100529	0.665695874

CDHR5	UCL	-0.177077917	0.180460998	-0.981253114	0.334963951	0.666245234
CGREF1	NIH	0.201773333	0.205716594	0.980831585	0.335075262	0.580281316
S100A12	UCL	-0.395976938	0.404551407	-0.978804996	0.335219044	0.666245234
TREML1	UCL	-0.137682029	0.141136411	-0.975524514	0.335229733	0.666245234
EGFR	UCL	0.058682986	0.059895717	0.979752628	0.335287971	0.666245234
FCRL2	NIH	0.30538	0.311704158	0.979711026	0.335618523	0.580423753
EPPK1	NIH	0.300966667	0.307258012	0.979524228	0.335709143	0.580423753
USP47	NIH	-0.333486667	0.340489093	-0.979434213	0.335752817	0.580423753
VPS28	UCL	-0.116140152	0.119313554	-0.973402833	0.335848689	0.666446668
VTI1A	NIH	0.19632	0.200552757	0.978894544	0.336014739	0.580533439
TUBB3	UCL	-0.111103913	0.11449962	-0.970343067	0.336048067	0.666446668
NCAM2	UCL	0.105782012	0.10790006	0.980370274	0.336073345	0.666446668
MMP15	UCL	0.159308881	0.162641004	0.979512404	0.336414801	0.6666715
CEACAM8	NIH	0.281746667	0.288282716	0.977327642	0.336776002	0.581505198
IL1A	NIH	-0.291873333	0.298788155	-0.976857108	0.337004834	0.581557015
CNTN5	NIH	-0.221006667	0.226353334	-0.976379109	0.337237405	0.581615217
HSDL2	UCL	-0.222887082	0.228527562	-0.975318164	0.337311027	0.667994669
CUZD1	NIH	-0.405806667	0.416054897	-0.975368082	0.337729678	0.582120984
IL16	UCL	-0.119209135	0.122358869	-0.974258228	0.338681964	0.670255505
GSN	NIH	-0.06652	0.068343386	-0.97332023	0.338728277	0.583272473
C1QTNF6	NIH	0.253333333	0.260314886	0.973180355	0.338796558	0.583272473
GRSF1	NIH	0.19196	0.197461972	0.97213655	0.339306388	0.583319722
EDAR	NIH	0.334266667	0.343876912	0.97205324	0.339347102	0.583319722
RELT	NIH	-0.085186667	0.087649673	-0.971899428	0.33942228	0.583319722
FUOM	UCL	0.255666636	0.262696229	0.973240605	0.339652049	0.671118019
SPINT1	UCL	-0.103821063	0.106797113	-0.97213361	0.339877373	0.671118019
POLR2A	UCL	-0.11705771	0.120812639	-0.968919403	0.339916163	0.671118019
SLC16A1	UCL	-0.261800052	0.269207951	-0.972482615	0.340036191	0.671118019
TXN	UCL	-0.104118336	0.107912828	-0.964837437	0.340411895	0.671406188
CALY	NIH	0.178166667	0.183752698	0.969600274	0.340547355	0.584909579
GCG	UCL	-0.542539758	0.560063685	-0.968710831	0.340932143	0.671978863
B3GAT3	NIH	0.12498	0.129029216	0.968617838	0.34102887	0.585392867
CLEC7A	UCL	0.192811895	0.198922583	0.969281077	0.341394991	0.672437708

ANG	UCL	-0.107305677	0.111122897	-0.965648665	0.342134633	0.673440762
GSAP	NIH	-0.208506667	0.215885308	-0.965821476	0.34240195	0.587405105
TFF3	NIH	0.113226667	0.117362021	0.964764118	0.342922108	0.587534597
REN	UCL	0.355421811	0.368348626	0.964906031	0.343095484	0.674824392
AOC1	UCL	0.195439419	0.203661971	0.959626475	0.343401085	0.674824392
MAGEA3	UCL	-0.280254844	0.291928008	-0.96001355	0.343530173	0.674824392
ENTPD2	NIH	-0.152213333	0.157996128	-0.963399135	0.343594385	0.587534597
EIF2AK3	NIH	-0.19414	0.201523841	-0.963359962	0.343613692	0.587534597
TNFRSF11B	NIH	0.11536	0.119857877	0.962473243	0.344050908	0.587534597
CD33	NIH	-0.36746	0.381856724	-0.9622981	0.34413731	0.587534597
CELA3A	NIH	-0.269593333	0.280164615	-0.962267605	0.344152356	0.587534597
CLIC5	NIH	-0.16776	0.174377392	-0.962051319	0.344259078	0.587534597
GRAP2	NIH	0.26676	0.277377786	0.96172085	0.344422185	0.587534597
PTGR1	NIH	0.36212	0.376630735	0.961472249	0.344544919	0.587534597
TRIM26	NIH	0.274733333	0.28576618	0.961392048	0.344584521	0.587534597
AKAP12	NIH	0.10856	0.112943964	0.961184609	0.344686964	0.587534597
ERBB3	UCL	0.093513611	0.09724312	0.961647573	0.345085721	0.677100584
ARTN	UCL	-0.098582784	0.103280641	-0.954513668	0.345152197	0.677100584
RAC3	UCL	-0.114723844	0.120298552	-0.953659387	0.345722997	0.677765473
NIT2	UCL	-0.26141381	0.27422978	-0.953265581	0.345967035	0.677789306
HEPH	UCL	-0.131987675	0.137743493	-0.9582135	0.346350285	0.678085654
RANBP2	NIH	0.15904	0.166097194	0.957511659	0.34650423	0.590041879
MELTF	NIH	-0.145466667	0.151986344	-0.957103531	0.346706556	0.590041879
SLC51B	UCL	-0.343662507	0.358709184	-0.95805327	0.346762358	0.678438
ENO2	NIH	0.148353333	0.155021301	0.956986764	0.346764457	0.590041879
RGMB	NIH	-0.108653333	0.113584865	-0.956582845	0.346964797	0.590041879
KCNIP4	UCL	0.367044591	0.384969448	0.953438234	0.347353847	0.678831382
PSMG4	UCL	-0.15792596	0.166247502	-0.94994486	0.347429401	0.678831382
ING1	NIH	0.612646667	0.641325287	0.955282255	0.347610404	0.5907963
DCTN6	UCL	-0.35681554	0.373502658	-0.955322626	0.347921471	0.678831382
BTC	UCL	-0.294143322	0.30998878	-0.948883769	0.348104727	0.678831382
SERPINA4	UCL	0.087226605	0.091237959	0.956034155	0.348124612	0.678831382
MTIF3	NIH	0.134593333	0.141151706	0.953536713	0.34847815	0.59192717

PAFAH1B3	UCL	0.355646229	0.372900882	0.95372858	0.348828105	0.679359387
NID1	UCL	-0.12437529	0.13068139	-0.951744467	0.348884555	0.679359387
COL28A1	UCL	0.329265726	0.345519788	0.952957653	0.349092644	0.679359387
PNPT1	NIH	0.309986667	0.325689851	0.951784851	0.349350494	0.592797981
TNFSF12	NIH	0.13694	0.1438909	0.951693259	0.349396143	0.592797981
PRR4	UCL	0.224286679	0.236661505	0.947710862	0.349790401	0.680264366
CCL8	NIH	-0.151866667	0.159836227	-0.950139211	0.350171276	0.593768685
TIMM10	UCL	-0.154812609	0.164045206	-0.94371919	0.350294292	0.680791368
TSPAN7	UCL	0.352718395	0.371575312	0.949251427	0.350529777	0.68079637
CNTNAP4	UCL	0.427864841	0.450735293	0.949259682	0.351198502	0.681390798
IL1B	NIH	0.2447	0.258225577	0.947621079	0.351429718	0.59536063
PQBP1	NIH	0.245326667	0.258934653	0.947446253	0.351517199	0.59536063
SAA4	UCL	0.049665111	0.052429694	0.947270649	0.351529499	0.681390798
DMP1	UCL	-0.185406113	0.195502463	-0.948356918	0.351535177	0.681390798
MUCL3	UCL	0.172487398	0.182143466	0.946986471	0.35195517	0.681752792
LYPD1	NIH	0.659246667	0.697467192	0.94520097	0.352642016	0.596920079
SAT1	UCL	-0.191741478	0.202366515	-0.947496069	0.353372301	0.683332111
CTSD	UCL	-0.142256569	0.150510728	-0.945158998	0.353427649	0.683332111
KIRREL2	UCL	-0.145722972	0.154542094	-0.942933855	0.353976459	0.683332111
CHM	UCL	-0.199309118	0.2132562	-0.93459941	0.354007462	0.683332111
CD5L	UCL	0.188432553	0.199751034	0.943337058	0.354218432	0.683332111
RSPO1	NIH	0.13026	0.138297546	0.941882222	0.354308997	0.599216154
TNR	NIH	0.140353333	0.149044874	0.941685075	0.354408187	0.599216154
ANGPTL3	UCL	0.141842567	0.150871408	0.940155387	0.35457579	0.683332111
HIP1	UCL	-0.102909986	0.109902217	-0.936377707	0.354580733	0.683332111
ENOPH1	UCL	0.1131903	0.120228599	0.941459034	0.354640716	0.683332111
MME	NIH	0.339373333	0.360610758	0.941107068	0.354699105	0.599361572
Testosterone	NIH	0.205532937	0.218299904	0.941516386	0.354788615	0.601799178
IL1RAP	NIH	0.162393333	0.172795001	0.939803422	0.35535583	0.600124597
DAND5	UCL	-0.657121492	0.704458037	-0.932804307	0.355400722	0.683720563
GSTT2B	UCL	-0.91942274	0.977730819	-0.940363873	0.355488798	0.683720563
ADH4	UCL	0.290794669	0.309429759	0.939776026	0.35554818	0.683720563
KLKB1	UCL	0.099726557	0.106157784	0.939418228	0.355935383	0.683720563

ADGRG2	UCL	0.11856187	0.126256796	0.939053373	0.35601187	0.683720563
CCL25	UCL	0.201530024	0.214973718	0.937463547	0.356542873	0.684290753
SAA4	NIH	0.1653	0.17641134	0.937014594	0.356763445	0.602154112
CRH	NIH	-0.3659	0.391010283	-0.935781016	0.357387253	0.60285912
LILRA2	UCL	-0.138514474	0.148086987	-0.935358851	0.357651562	0.68541887
LAMB1	UCL	-0.148065909	0.158264368	-0.93556061	0.357745839	0.68541887
ADGRG1	UCL	-0.509346435	0.54444913	-0.935526217	0.357860296	0.68541887
CEACAM20	NIH	-0.134693333	0.144086782	-0.934807006	0.357880311	0.602999677
SKIV2L	NIH	0.32912	0.35216455	0.934563118	0.358003842	0.602999677
CA13	NIH	-0.326093333	0.34898876	-0.934394945	0.358089039	0.602999677
CD200	UCL	0.085724678	0.091948872	0.932308097	0.35826644	0.68541887
COL9A2	UCL	-0.110357118	0.119166458	-0.926075344	0.358380751	0.68541887
VSIG2	UCL	0.323220476	0.346147263	0.933765799	0.358612491	0.68541887
FGF7	NIH	0.353213333	0.378527884	0.933123684	0.358733497	0.603502355
UPK3A	NIH	0.48562	0.520497392	0.932992186	0.358800203	0.603502355
ZHX2	UCL	0.086423166	0.09313903	0.927894201	0.358889333	0.68541887
CTSC	NIH	0.151906667	0.162919289	0.932404431	0.359098459	0.603656892
MFAP3	UCL	0.162045551	0.174440358	0.928945302	0.359243284	0.68541887
CTSV	UCL	0.153041228	0.164129049	0.932444497	0.359257359	0.68541887
SCN2B	NIH	0.187693333	0.201466837	0.931633892	0.359489717	0.603967503
PRKAR1A	UCL	-0.337960909	0.36408205	-0.928254796	0.359629354	0.68541887
HAGH	UCL	-0.328251967	0.353719321	-0.928001237	0.359710074	0.68541887
CDHR5	NIH	0.16292	0.174961031	0.931178784	0.359720941	0.604009043
VEGFD	UCL	-0.120627544	0.12957616	-0.930939333	0.360184164	0.685875122
IFIT1	NIH	-0.190146667	0.205353411	-0.925948423	0.362385363	0.608133784
ERBB2	UCL	-0.080895738	0.087369508	-0.925903553	0.362523862	0.689881022
GFRA1	UCL	0.127535216	0.138210073	0.922763535	0.364548614	0.692712437
AMIGO2	UCL	0.190959228	0.207103788	0.92204604	0.364582959	0.692712437
PADI4	UCL	-0.215037656	0.234611688	-0.916568383	0.364722696	0.692712437
THAP12	NIH	-0.349173333	0.379154367	-0.920926577	0.364955796	0.611500428
ANXA3	NIH	0.26522	0.288016061	0.920851423	0.364994354	0.611500428
DOCK9	NIH	0.240866667	0.26158301	0.920803941	0.365018717	0.611500428
ITGA11	UCL	0.135736212	0.147356966	0.921138752	0.365304968	0.692991883

HTR1B	UCL	-0.154873915	0.168762757	-0.917701976	0.365343993	0.692991883
IL17RA	UCL	0.150214857	0.163383895	0.91939819	0.366068576	0.693915985
PGD	NIH	0.187453333	0.204143567	0.918242667	0.366334468	0.613252152
DPP7	NIH	0.2191	0.238683351	0.917952591	0.366483679	0.613252152
RABGAP1L	UCL	-0.244392961	0.266782088	-0.916077097	0.36648662	0.694258192
BRD2	UCL	-0.264359752	0.288288621	-0.91699683	0.367203233	0.694669419
TNFRSF8	NIH	-0.15046	0.164161848	-0.916534515	0.367213693	0.614122386
PCARE	UCL	0.35324493	0.385036872	0.917431436	0.367292312	0.694669419
PNLIPRP2	UCL	0.913329859	0.995733044	0.917243697	0.367416668	0.694669419
CPPED1	UCL	-0.242425222	0.264688044	-0.915890335	0.367675568	0.694709557
CEBPA	NIH	0.200333333	0.21905734	0.914524634	0.36825	0.615503571
TP53INP1	UCL	0.06684269	0.073233825	0.91272973	0.368293016	0.694996626
LTBR	UCL	-0.110416741	0.120644111	-0.915226945	0.368303036	0.694996626
ADGRV1	UCL	-0.1754617	0.192598144	-0.911024875	0.368717091	0.695172365
TNFSF11	UCL	-0.19149086	0.210388495	-0.910177433	0.368871823	0.695172365
LRCH4	NIH	0.32284	0.353526575	0.913198676	0.368934721	0.616295864
IL1R2	UCL	-0.091128038	0.099877111	-0.91240162	0.369743054	0.695415343
TNFSF14	NIH	0.236813333	0.259796747	0.911533097	0.369796004	0.617382027
CDHR2	UCL	-0.274775579	0.301101739	-0.912567225	0.369798378	0.695415343
TACC3	NIH	0.331166667	0.363531952	0.910969902	0.370087534	0.617445376
NPPB	UCL	-0.479047541	0.530231312	-0.903468977	0.370147156	0.695415343
LRP1	UCL	0.13612733	0.149520771	0.91042421	0.370151312	0.695415343
MDK	UCL	-0.395764462	0.433848201	-0.912218747	0.370190309	0.695415343
RPL14	NIH	0.25974	0.285324052	0.91033335	0.370417218	0.617445376
TNFAIP8	UCL	-0.100433591	0.11097296	-0.905027598	0.370466591	0.695425603
BCL7A	UCL	-0.326002603	0.359000725	-0.908083411	0.370687194	0.695425603
KYNU	NIH	0.16058	0.176532068	0.909636431	0.370778387	0.617445376
NLGN1	NIH	-0.16926	0.186121269	-0.90940708	0.370897295	0.617445376
SLURP1	NIH	-0.139606667	0.153557438	-0.909149494	0.371030872	0.617445376
NTF3	UCL	0.125491599	0.137876257	0.910175556	0.371032916	0.695425603
ARNT	NIH	0.29646	0.326177106	0.908892729	0.371164055	0.617445376
CD93	UCL	-0.089914991	0.098918006	-0.908985079	0.371329887	0.695425603
SLC4A1	NIH	0.327946667	0.361045497	0.908325044	0.371458621	0.617445376

ITGB5	UCL	-0.129821635	0.143120236	-0.907080924	0.3716374	0.695425603
DNAJA1	UCL	-0.134131984	0.148512797	-0.903167851	0.371754986	0.695425603
SARG	NIH	0.229473333	0.252801712	0.907720646	0.371772405	0.617445376
CCL16	NIH	0.208093333	0.229320844	0.907433141	0.371921729	0.617445376
PRC1	NIH	0.287613333	0.316968207	0.907388587	0.371944872	0.617445376
NME1	UCL	-0.087048555	0.096601517	-0.901109606	0.372253418	0.695425603
CD83	NIH	0.13252	0.146178544	0.906562592	0.372374113	0.617807305
PLA2G15	UCL	0.111302785	0.122648636	0.907493053	0.372455574	0.695425603
TRIM25	NIH	0.217753333	0.24038761	0.905842582	0.37274854	0.618077937
IFI30	UCL	-0.154444045	0.170561176	-0.905505278	0.37276539	0.695425603
ARSB	UCL	-0.210221334	0.232395975	-0.904582508	0.372985149	0.695425603
COL24A1	UCL	-0.202610305	0.224975178	-0.900589601	0.373018714	0.695425603
BCL2L11	UCL	-0.121629327	0.135184439	-0.899728752	0.373050751	0.695425603
PF4	NIH	0.152413333	0.168398299	0.905076442	0.373147227	0.618388463
SLC13A1	UCL	-0.112916392	0.124489146	-0.907038048	0.373464308	0.695732169
LSM1	UCL	-0.328016247	0.364376257	-0.900213009	0.373691243	0.695732169
NRN1	NIH	-0.12436	0.137777107	-0.902617299	0.374428804	0.620135047
EPHA2	NIH	0.100293333	0.111190582	0.90199486	0.374753641	0.620135047
TBCB	NIH	0.335053333	0.371524053	0.901834835	0.374837184	0.620135047
SPINK4	NIH	-0.35422	0.392956686	-0.901422505	0.375052502	0.620140514
CFC1	NIH	0.170433333	0.189205572	0.900783902	0.375386139	0.620341501
COLEC12	UCL	-0.109973472	0.122192456	-0.900002142	0.376215922	0.699883262
NOMO1	UCL	-0.089240085	0.099306011	-0.898637293	0.376399756	0.699883262
DNAJB6	NIH	0.295193333	0.328420839	0.898826441	0.376410015	0.621682267
FOXJ3	UCL	-0.125971475	0.140857481	-0.894318671	0.376916653	0.700337064
STAU1	UCL	0.173247772	0.193725389	0.894295645	0.377123003	0.700337064
GPNMB	NIH	0.105046667	0.117072511	0.897278671	0.377220881	0.622319013
SFTPA1	NIH	0.337273333	0.375939763	0.897147274	0.377289772	0.622319013
GRIN2B	UCL	-0.425473031	0.473886557	-0.897837308	0.377622097	0.700415812
SLITRK1	NIH	-0.1242	0.138566406	-0.896321147	0.37772309	0.622319013
LGMN	NIH	-0.116333333	0.129793034	-0.896298746	0.377734844	0.622319013
BGLAP	UCL	-0.224862082	0.250715872	-0.896880122	0.377762974	0.700415812
TP53	NIH	0.166166667	0.185441138	0.896061514	0.377859339	0.622319013

CTLA4	UCL	-0.11818294	0.133007434	-0.88854387	0.378049087	0.700415812
TACSTD2	UCL	0.137517155	0.153565308	0.895496229	0.378345118	0.700415812
CDK5RAP3	UCL	-0.35664704	0.398061298	-0.895960099	0.37836352	0.700415812
KLRK1	UCL	-0.130130592	0.146308459	-0.889426303	0.378775944	0.700544695
CCDC80	UCL	0.115710689	0.129471944	0.893712458	0.378912475	0.700544695
ITGB1BP1	NIH	0.17436	0.195099963	0.89369571	0.379102324	0.624014798
GFAP	UCL	-0.196926213	0.220435052	-0.893352536	0.37920004	0.700633196
LYPD1	UCL	0.35239389	0.394928216	0.892298588	0.379697523	0.70087838
ADM	UCL	-0.090693601	0.101629183	-0.892397227	0.379920008	0.70087838
APOL1	UCL	-0.120255971	0.134757347	-0.892388979	0.380052081	0.70087838
SOX2	NIH	0.10596	0.118833141	0.891670448	0.380168493	0.625417797
IGDCC4	UCL	0.113517365	0.127223255	0.892268994	0.38040183	0.701081053
RGL2	NIH	-0.262326667	0.294759365	-0.889968895	0.381065752	0.626541497
CCL19	UCL	-0.39201654	0.440272877	-0.890394482	0.381235189	0.702174202
AMY1A_AMY1B_AMY1C	NIH	-0.164106667	0.184498145	-0.889475973	0.381325934	0.626617054
LRTM1	UCL	0.371519951	0.417922656	0.888968199	0.381745092	0.702670595
TNFRSF10A	NIH	0.120426667	0.135633278	0.887884366	0.382166826	0.627245992
QPCT	UCL	0.108002602	0.121535152	0.888653196	0.382201738	0.703068395
NPR1	NIH	0.287406667	0.323774093	0.887676541	0.382276715	0.627245992
HDAC8	NIH	0.32608	0.367399935	0.887534179	0.382352001	0.627245992
TPPP3	NIH	0.35764	0.403690937	0.885925265	0.383203522	0.628290527
GC	UCL	0.088050741	0.099494021	0.884985244	0.383225714	0.704508655
CNTN4	NIH	0.074913333	0.084612502	0.885369552	0.383497918	0.628420958
ATXN10	UCL	-0.267322306	0.303536138	-0.880693508	0.384052835	0.704872888
ARSA	UCL	-0.15501547	0.175285435	-0.884360243	0.384096991	0.704872888
KIAA1549L	NIH	0.221653333	0.250754151	0.883946816	0.384252296	0.629304573
ASGR2	UCL	-0.116027665	0.131110086	-0.884963687	0.384285975	0.704872888
SLITRK2	UCL	0.120053788	0.135703307	0.884678429	0.384388431	0.704872888
EDF1	UCL	-0.272676935	0.309684456	-0.880499263	0.384961705	0.705481545
MDH1	UCL	0.155053229	0.175936831	0.881300568	0.385420263	0.705654786
PDIA5	UCL	-0.137839097	0.157373084	-0.875874662	0.385539067	0.705654786
MMP13	UCL	-0.291836072	0.331736634	-0.879722173	0.386154107	0.70612701

NLGN2	NIH	-0.221946667	0.252303225	-0.879682243	0.386519234	0.632410104
APOA2	UCL	-0.173204676	0.198478591	-0.872661759	0.386573968	0.70612701
GFRAL	NIH	0.18036	0.205055587	0.879566377	0.386580946	0.632410104
CA1	UCL	-0.284336221	0.323712201	-0.878361148	0.386615302	0.70612701
YY1	UCL	0.148019257	0.168983846	0.875937316	0.386884117	0.70612701
CD55	UCL	-0.088260469	0.10038487	-0.879220835	0.387236237	0.70612701
SEZ6L2	UCL	-0.110673006	0.125918342	-0.878926802	0.387246527	0.70612701
LCP1	NIH	-0.173113333	0.197251777	-0.877626229	0.387615227	0.633747646
LRPAP1	NIH	0.082406667	0.093984688	0.876809489	0.388051158	0.633764057
PTEN	NIH	0.52518	0.598976589	0.876795537	0.388058607	0.633764057
SNAP25	UCL	-0.120391424	0.137694944	-0.874334384	0.388311484	0.707153903
CCL2	UCL	0.15707834	0.179289294	0.876116675	0.388481404	0.707153903
ACRV1	UCL	0.395165806	0.450976833	0.876244138	0.388720352	0.707153903
TGFBI	UCL	-0.151779195	0.173252437	-0.876058068	0.388777394	0.707153903
RNF4	NIH	0.399333333	0.456825612	0.874148303	0.389473736	0.635720244
IL10RB	UCL	-0.102699556	0.117407841	-0.874724854	0.38972708	0.708019215
CABP2	UCL	-0.180678164	0.207677496	-0.869993947	0.389792035	0.708019215
GPRC5C	UCL	-0.342552525	0.391509838	-0.874952532	0.389979793	0.708019215
CASP7	NIH	-0.094886667	0.108731861	-0.872666633	0.390267233	0.635874352
FOLR2	NIH	0.11676	0.133859377	0.872258653	0.390485906	0.635874352
CELA3A	UCL	-0.172774635	0.198030293	-0.872465685	0.39056375	0.708639256
PAG1	NIH	0.184453333	0.211503326	0.872106064	0.390567712	0.635874352
TNFAIP8	NIH	0.264446667	0.303397517	0.871617768	0.390829571	0.635874352
NINJ1	NIH	0.255346667	0.293064263	0.871299231	0.391000453	0.635874352
PMS1	NIH	0.204653333	0.23490944	0.871200974	0.391053174	0.635874352
MSLN	NIH	-0.19736	0.226555653	-0.871132532	0.3910899	0.635874352
HLA-DRA	UCL	0.08887762	0.102358761	0.868295196	0.391350341	0.70962596
MYH4	NIH	-0.366566667	0.421164673	-0.87036423	0.391502322	0.636191273
DNLZ	UCL	0.224031267	0.259179582	0.864386249	0.392243508	0.71050317
PCSK9	UCL	0.133275899	0.153367585	0.868996525	0.392359816	0.71050317
PDGFC	UCL	-0.167040487	0.192239862	-0.868917013	0.392563332	0.71050317
PARD3	NIH	0.270413333	0.311576772	0.86788669	0.392834151	0.63800105
CWC15	UCL	-0.101897687	0.118191343	-0.862141711	0.393283478	0.710936772

PIGR	UCL	-0.145891974	0.168179579	-0.867477338	0.393289347	0.710936772
BRD3	UCL	0.157911289	0.183267848	0.861642079	0.393734039	0.71130074
KRT14	NIH	0.326706667	0.377349781	0.865792649	0.39396208	0.63915152
PRSS27	NIH	0.105773333	0.122173932	0.865760242	0.393979552	0.63915152
EFNA4	UCL	0.087512314	0.101094265	0.86565063	0.394262256	0.711571965
CPLX2	UCL	-0.138756718	0.161659561	-0.858326699	0.394371052	0.711571965
MLLT1	NIH	0.268413333	0.310516338	0.864409696	0.394708124	0.639942525
TAGLN3	NIH	-0.286033333	0.331039639	-0.86404557	0.394904703	0.639942525
BNIP3L	UCL	-0.200662726	0.232514209	-0.863012744	0.395069871	0.71183608
PTPRK	UCL	0.076864511	0.08901041	0.863545184	0.395188271	0.71183608
TIMP4	UCL	0.111137738	0.128861825	0.862456652	0.395248019	0.71183608
ANXA2	NIH	0.1761	0.204292979	0.861997316	0.396011652	0.641176854
CHI3L1	NIH	0.288006667	0.334182267	0.861825103	0.396104812	0.641176854
NTRK2	NIH	0.091806667	0.106593794	0.861275909	0.396401995	0.641264218
PDIA2	UCL	0.091773754	0.107408466	0.854436877	0.396503536	0.713568113
TSC22D1	NIH	0.120746667	0.140279366	0.860758571	0.396682071	0.641264218
RNF4	UCL	0.22634029	0.26281357	0.861219953	0.396757526	0.713568113
PSMD9	UCL	-0.161419013	0.188409348	-0.856746308	0.396942098	0.713568113
PDRG1	NIH	0.190526667	0.221507294	0.860137214	0.397018626	0.641264218
TANK	UCL	-0.244008188	0.284539852	-0.857553647	0.397499864	0.714131594
KLK14	NIH	-0.132233333	0.153916677	-0.859122844	0.397568446	0.641264218
RUVBL1	NIH	0.375446667	0.43709909	0.858950923	0.39766168	0.641264218
ACTA2	NIH	0.158013333	0.183966311	0.858925377	0.397675535	0.641264218
TCTN3	NIH	0.09794	0.114079361	0.858525146	0.397892643	0.641264218
FARSA	NIH	0.201633333	0.234870234	0.858488237	0.397912668	0.641264218
HSPG2	UCL	-0.093838443	0.109402099	-0.857738964	0.398355014	0.715228321
FRMD7	UCL	0.08280574	0.096988301	0.853770399	0.398874885	0.715721081
ARHGAP45	NIH	0.167166667	0.19515129	0.856600368	0.398937802	0.642562263
C1S	UCL	0.058883409	0.068858223	0.855139835	0.399128607	0.715721081
FGF6	NIH	0.219326667	0.256180185	0.856142199	0.399186845	0.642609533
CNGB3	NIH	0.109506667	0.127996134	0.855546675	0.399510696	0.642777111
RNASET2	UCL	-0.098915968	0.115457649	-0.856729448	0.399589145	0.715721081
C9	UCL	0.201359553	0.235161314	0.856261389	0.399608897	0.715721081

BNIP2	NIH	0.1746	0.204236468	0.854891399	0.399867234	0.642997065
EPPK1	UCL	-0.28937851	0.338888651	-0.853904398	0.400244063	0.716419715
PRTG	NIH	0.103933333	0.121950006	0.852261813	0.401300029	0.644946475
CASP4	UCL	-0.717194496	0.841293655	-0.85249008	0.401702788	0.718590728
ICAM4	NIH	-0.152613333	0.179263616	-0.851334682	0.401805973	0.645404981
CD101	NIH	0.129053333	0.151756466	0.850397595	0.40231776	0.645848016
FOLR3	NIH	-0.995966667	1.171696149	-0.850021286	0.402523396	0.645848016
PLAU	NIH	0.085426667	0.100648027	0.848766435	0.403209595	0.646594334
MNAT1	UCL	0.1451966	0.172009745	0.844118452	0.403322361	0.720009054
MICB_MICA	UCL	-0.709096835	0.835312106	-0.848900464	0.40340616	0.720009054
UGDH	UCL	0.466208909	0.549355867	0.848646456	0.403430594	0.720009054
C1R	UCL	0.038874283	0.045939046	0.846214415	0.403480955	0.720009054
IGF2R	NIH	0.090453333	0.106681861	0.847879219	0.403695203	0.647018339
NPPB	NIH	-0.772286667	0.911348465	-0.847410948	0.403951655	0.647074803
ECM1	UCL	-0.107468971	0.127456238	-0.843183296	0.40420693	0.720616156
SSB	NIH	-0.2827	0.333935113	-0.846571652	0.404411559	0.647456929
UBAC1	UCL	0.108737086	0.128750905	0.844553955	0.404632247	0.720616156
CLEC4M	UCL	-0.129259379	0.152711521	-0.846428471	0.40477305	0.720616156
TMEM25	UCL	0.1123332	0.133217458	0.843231826	0.404807297	0.720616156
PM20D1	NIH	-0.819533333	0.969480138	-0.845332773	0.405091023	0.647972693
SDC4	NIH	-0.113626667	0.134585864	-0.844268955	0.405675048	0.647972693
S100A3	UCL	-0.103709047	0.122551984	-0.84624535	0.40568501	0.721461135
IL13RA1	UCL	-0.097721423	0.11563495	-0.845085528	0.406091586	0.721461135
ASAH1	NIH	0.157986667	0.187303201	0.843480869	0.406108041	0.647972693
SHC1	NIH	0.36502	0.432796004	0.843399655	0.406152679	0.647972693
MZB1	UCL	0.179236047	0.212530835	0.843341374	0.406221817	0.721461135
MDK	NIH	0.128906667	0.152926114	0.842934299	0.406408511	0.647972693
CLEC11A	UCL	0.131458445	0.155915049	0.843141484	0.406511456	0.721461135
INPP5D	NIH	0.238353333	0.282838435	0.842719037	0.406526886	0.647972693
CD22	NIH	0.191606667	0.227376338	0.842685164	0.406545516	0.647972693
F10	NIH	0.080946667	0.096081976	0.842475038	0.406661091	0.647972693
PXDNL	NIH	-0.294446667	0.349552017	-0.842354364	0.406727475	0.647972693
STC2	UCL	-0.140040712	0.166049496	-0.84336728	0.406785774	0.721461135

MDGA1	UCL	0.249390376	0.295815374	0.8430609	0.406957914	0.721461135
HCLS1	NIH	0.19266	0.228857276	0.841834718	0.407013414	0.648075251
GH1	UCL	0.599411842	0.714888893	0.838468534	0.407173764	0.721461135
LAYN	UCL	-0.099143436	0.117757411	-0.841929485	0.407256542	0.721461135
F7	UCL	0.088358853	0.105231721	0.839659867	0.407823146	0.72202729
ACADSB	NIH	0.193206667	0.229913368	0.840345511	0.407833561	0.649027838
CEACAM19	NIH	-0.122306667	0.145704758	-0.839414364	0.408346896	0.649491393
MLN	UCL	-0.351332699	0.418753942	-0.838995563	0.408693715	0.723130587
EGF	NIH	-0.25016	0.298536592	-0.83795423	0.409152675	0.650419333
MFAP5	UCL	-0.142345769	0.169847123	-0.838081715	0.409357862	0.723867533
FBN2	NIH	0.25722	0.307353093	0.836887625	0.409741913	0.65078583
CPB1	NIH	0.218246667	0.260832376	0.836731506	0.409828205	0.65078583
ADAMTS16	UCL	0.089740565	0.107302476	0.836332665	0.40989576	0.724228254
IL20RB	UCL	0.130485223	0.15613824	0.835703175	0.410057393	0.724228254
BCL2L11	NIH	0.095173333	0.113846343	0.835980594	0.410243415	0.651091692
KLK7	UCL	0.13279049	0.158812931	0.836144068	0.410390824	0.724379456
ENDOU	NIH	0.206526667	0.247201478	0.835458867	0.410532054	0.651091736
AKR7L	UCL	0.248945924	0.297384777	0.837117242	0.41066922	0.724433392
TEX101	NIH	0.276193333	0.330700778	0.83517594	0.410688633	0.651091736
SLC28A1	UCL	0.291295652	0.349242512	0.834078447	0.411360048	0.724943158
CHAD	UCL	0.147252214	0.17649078	0.834333749	0.411457588	0.724943158
ADH1B	UCL	0.256555159	0.306804935	0.83621588	0.411723416	0.724943158
NUDT10	UCL	-0.045811487	0.055248145	-0.82919503	0.411950251	0.724943158
DDC	NIH	-0.200453333	0.24117488	-0.831153448	0.41291883	0.654135255
DTX3	UCL	0.060932606	0.073487301	0.829158308	0.413279141	0.726710487
HMMR	NIH	0.21242	0.255805359	0.830396991	0.413339077	0.654135255
ATP6V1D	NIH	-0.145353333	0.175161827	-0.829823116	0.41365807	0.654135255
TERF1	UCL	-0.119841097	0.145102561	-0.825906149	0.413671568	0.726710487
PGR	UCL	0.397176807	0.478276584	0.830433312	0.413700394	0.726710487
CD69	NIH	0.224233333	0.270324125	0.829498047	0.41383883	0.654135255
CD79B	NIH	-0.1615	0.194748638	-0.829274093	0.413963392	0.654135255
VAV3	NIH	-0.427446667	0.515459459	-0.829253707	0.413974732	0.654135255
DNAJB8	NIH	0.19314	0.233032737	0.828810589	0.414221264	0.654135255

GOLM2	NIH	0.079906667	0.096449433	0.828482496	0.414403861	0.654135255
LELP1	NIH	0.212146667	0.256187239	0.828092247	0.414621116	0.654135255
DHODH	UCL	0.132464459	0.160885994	0.823343634	0.414877484	0.72833235
EPCAM	UCL	0.389638513	0.471164276	0.826969558	0.415529096	0.72833235
CACNA1H	UCL	-0.164123657	0.197859544	-0.829495781	0.415671153	0.72833235
CNTN5	UCL	0.162270102	0.196278473	0.826734075	0.41585387	0.72833235
TFF1	UCL	-0.201011677	0.24330893	-0.826158239	0.415869549	0.72833235
THRAP3	NIH	0.368506667	0.446645381	0.825054244	0.41631482	0.656453287
ZNRD2	NIH	0.267853333	0.32492036	0.82436611	0.416699055	0.656705139
PVR	UCL	-0.109452016	0.13272344	-0.824662287	0.416806344	0.729535894
FES	NIH	0.13844	0.168133672	0.823392471	0.417243086	0.657208416
NTRK3	UCL	0.085861226	0.104370368	0.82265903	0.417342368	0.729837087
TNFRSF21	UCL	0.111933449	0.136003566	0.823018483	0.4174778	0.729837087
FGFBP2	NIH	-0.184013333	0.223963198	-0.821623082	0.41823288	0.658412903
SNCG	UCL	-0.240201769	0.292304701	-0.821751303	0.418304978	0.730014354
ITGB1	UCL	0.067715836	0.082389489	0.821898972	0.41847987	0.730014354
GLIPR1	UCL	0.096155538	0.1177073	0.816903779	0.418553217	0.730014354
REG1B	UCL	-0.272066502	0.330912161	-0.822171362	0.418670865	0.730014354
KRT8	UCL	-0.565979563	0.689612821	-0.820720766	0.419196555	0.730014354
CYB5A	NIH	-0.20274	0.247390614	-0.819513709	0.419414763	0.659918334
GAMT	UCL	0.155426437	0.19063312	0.815317073	0.419570078	0.730014354
DEFB104A_DEFB104B	UCL	-0.281698534	0.343643867	-0.81973974	0.41981319	0.730014354
CD4	UCL	-0.11835092	0.144264535	-0.820374321	0.4198161	0.730014354
TPSD1	UCL	-0.107913005	0.131644646	-0.819729542	0.420079969	0.730014354
EVI2B	UCL	-0.208886041	0.255252387	-0.818350979	0.420322879	0.730014354
DBH	UCL	-0.59051938	0.721623919	-0.818320131	0.420326431	0.730014354
GFER	NIH	0.134446667	0.164583142	0.816892089	0.420886541	0.661878029
APOD	UCL	-0.079059108	0.097039788	-0.814708168	0.421696414	0.731958799
ATF4	NIH	-0.126006667	0.154739478	-0.814314928	0.422336472	0.663522979
ZBP1	NIH	0.19562	0.240252546	0.814226544	0.422386252	0.663522979
BAX	UCL	-0.427476796	0.528314812	-0.809132711	0.423639713	0.734854707
IL2	NIH	0.299726667	0.369302951	0.811601061	0.423866645	0.665491109

CTHRC1	UCL	0.094223071	0.115963834	0.812521181	0.423867614	0.734854707
DCLRE1C	UCL	-0.120104035	0.148900578	-0.806605567	0.424825519	0.736078833
SUSD2	NIH	-0.114013333	0.140824566	-0.809612534	0.424990014	0.666896884
IL13RA2	NIH	0.231273333	0.286023717	0.808580967	0.425573494	0.667003582
SH3BGRL2	NIH	0.196866667	0.243502102	0.808480357	0.425630427	0.667003582
MMP1	NIH	0.160126667	0.198146177	0.808123928	0.425832164	0.667003582
HNF1A	UCL	-0.185810597	0.230431349	-0.806359887	0.425858187	0.737430972
EREG	NIH	-0.283133333	0.350464508	-0.807880191	0.425970151	0.667003582
PCDH17	UCL	0.136887058	0.169603036	0.80710264	0.426504658	0.738113152
MMP7	UCL	-0.134469126	0.167608578	-0.802280689	0.427305309	0.739061195
LGALS4	NIH	-0.143813333	0.178632504	-0.805079313	0.42755779	0.668958971
FUT8	NIH	0.15514	0.19275152	0.804870438	0.427676333	0.668958971
DOC2B	NIH	-0.1561	0.194067105	-0.804360944	0.427965572	0.669053607
SPTLC1	NIH	0.09482	0.117942096	0.803953832	0.428196775	0.669057462
HAVCR2	UCL	-0.194895903	0.242477817	-0.803767972	0.428638088	0.740927931
NRP1	NIH	-0.097306667	0.121262393	-0.802447194	0.429053077	0.670037507
SERPINA3	UCL	-0.045175038	0.056457638	-0.800158121	0.429584428	0.742124872
WDR46	NIH	0.185	0.230932141	0.801101134	0.429819001	0.670652387
IL22	NIH	-0.231513333	0.289048629	-0.800949427	0.429905377	0.670652387
SLIRP	UCL	-0.1015571	0.127397653	-0.797166171	0.430159598	0.742229287
AFAP1	UCL	-0.17376646	0.217173937	-0.800125757	0.430432536	0.742229287
CD83	UCL	-0.114521658	0.143043886	-0.800605055	0.430505684	0.742229287
EFCAB14	UCL	-0.080989239	0.10131209	-0.799403493	0.430735026	0.742229287
CD164	UCL	-0.072442867	0.090753161	-0.7982407	0.431194185	0.742229287
MKI67	UCL	0.179916788	0.225613233	0.797456719	0.431452964	0.742229287
ENOX2	NIH	0.107026667	0.134292683	0.796965734	0.432177324	0.673837246
NPL	UCL	-0.149818144	0.187839473	-0.797586055	0.432233623	0.742229287
CD109	UCL	0.115087817	0.144541548	0.796226541	0.432599806	0.742229287
HNMT	UCL	-0.201774381	0.253182155	-0.796953409	0.43260844	0.742229287
NMT1	UCL	0.252699971	0.317878064	0.79495882	0.432839456	0.742229287
PPIE	UCL	0.217516403	0.273202941	0.796171528	0.43291236	0.742229287
PSCA	UCL	0.945240432	1.187230389	0.796172707	0.432937843	0.742229287
TCP11	NIH	0.380173333	0.478286549	0.794865199	0.433378231	0.675349667

SHISA5	UCL	-0.098717531	0.124114619	-0.795373923	0.43352949	0.742229287
PRSS27	UCL	0.122431656	0.154109433	0.794446216	0.433781133	0.742229287
CADPS	UCL	0.09279784	0.117571707	0.789287167	0.43407355	0.742229287
F3	UCL	0.101196207	0.127528651	0.793517423	0.434094383	0.742229287
THBD	NIH	0.090706667	0.114314122	0.7934861	0.434167787	0.676219797
ICOSLG	UCL	0.059887579	0.075538178	0.792812068	0.434514897	0.742229287
EFNB2	UCL	0.140915024	0.177320948	0.794689096	0.434520378	0.742229287
MORC3	UCL	-0.049133036	0.062372358	-0.787737347	0.434749275	0.742229287
LRP2	UCL	-0.118201316	0.150289826	-0.786489135	0.434896654	0.742229287
CSTB	UCL	-0.098997491	0.124981925	-0.792094469	0.434977341	0.742229287
HMGCS1	NIH	0.177713333	0.224701916	0.790884816	0.435659448	0.678048467
PPP1R14A	NIH	0.22212	0.280940376	0.790630392	0.43580551	0.678048467
CGA	NIH	0.181893333	0.230180824	0.790219315	0.436041569	0.678055071
ITGB1BP1	UCL	-0.216127193	0.275272827	-0.785138133	0.436227639	0.743928465
IL5RA	UCL	0.207284398	0.262567238	0.789452636	0.436825079	0.744190793
INSL3	UCL	0.332072769	0.421198429	0.788399826	0.437445301	0.744190793
RIPK4	UCL	0.298671956	0.379232534	0.787569444	0.437545966	0.744190793
SGSH	UCL	0.221091392	0.280684357	0.787686903	0.437714091	0.744190793
CCL14	UCL	0.137886751	0.175227323	0.786902115	0.437781111	0.744190793
FES	UCL	-0.292764789	0.371796262	-0.787433385	0.437909054	0.744190793
TFF2	NIH	0.187926667	0.238800101	0.786962257	0.437914655	0.680605934
CALCOCO1	NIH	0.218786667	0.278244498	0.786310847	0.438289856	0.680722039
GLYR1	UCL	-0.141881116	0.181000481	-0.783871483	0.438496887	0.744646569
CSF3R	NIH	0.09322	0.11862871	0.78581315	0.438576653	0.680722039
TSLP	UCL	0.309205468	0.393567884	0.785647103	0.438686757	0.744646569
CTLA4	NIH	0.136693333	0.174066481	0.78529383	0.43887603	0.680722039
WIF1	UCL	-0.073251922	0.093174063	-0.786183622	0.43903349	0.744802607
CA8	NIH	0.074186667	0.09455555	0.784582888	0.439286075	0.680722039
SUSD5	NIH	-0.115366667	0.147062335	-0.784474601	0.439348551	0.680722039
B3GNT7	UCL	0.136785523	0.174255214	0.784972343	0.439551268	0.745248467
SMPDL3B	NIH	0.28248	0.360574136	0.783417255	0.43995887	0.680722039
PKD1	NIH	-0.103226667	0.131765582	-0.78341146	0.439962216	0.680722039
FDX2	NIH	0.158973333	0.202953228	0.783300345	0.440026384	0.680722039

CPXM2	NIH	0.096513333	0.123229364	0.783200775	0.440083889	0.680722039
TAP1	NIH	-0.11378	0.145430134	-0.782368802	0.440564562	0.680842913
FADD	NIH	0.161286667	0.206269256	0.781922956	0.44082228	0.680842913
PPP3R1	NIH	0.0916	0.117156953	0.781857137	0.440860334	0.680842913
FCRL6	UCL	0.281822439	0.360423368	0.781920554	0.441088269	0.747316278
RSPO1	UCL	0.111652104	0.142988719	0.780845542	0.441343749	0.747316278
CTRC	NIH	-0.254093333	0.325430966	-0.780790275	0.441477428	0.681131009
GLT8D2	NIH	-0.209593333	0.268536096	-0.780503389	0.441643458	0.681131009
KAZALD1	UCL	0.135317975	0.173197436	0.781293175	0.44178583	0.747316278
JUN	UCL	-0.425510954	0.544840204	-0.780983032	0.441793544	0.747316278
CCL26	NIH	0.19058	0.244340202	0.779978072	0.441947572	0.681131009
CD93	NIH	-0.104313333	0.133816248	-0.779526663	0.442209	0.681131009
CHEK2	UCL	-0.070424503	0.090851184	-0.775163293	0.442307797	0.747460019
KRT8	NIH	-0.339526667	0.435721337	-0.779228919	0.442381487	0.681131009
CDC123	UCL	-0.168653953	0.217241946	-0.776341568	0.442389953	0.747460019
TSPYL1	NIH	-0.189813333	0.243662666	-0.779000479	0.442513852	0.681131009
M6PR	NIH	0.10394	0.133521554	0.778451096	0.442832279	0.681131009
PTX3	NIH	0.166013333	0.21336068	0.778087758	0.443042949	0.681131009
CTSV	NIH	0.14342	0.184368911	0.777896876	0.44315365	0.681131009
CLEC2L	UCL	-0.09773871	0.125834091	-0.776726794	0.443255595	0.748489951
FH	NIH	0.16182	0.208124772	0.777514365	0.443375535	0.681131009
ESR1	UCL	0.24878141	0.320748737	0.775627092	0.444167787	0.749276702
LHB	UCL	-0.422416459	0.543938332	-0.776588877	0.444234185	0.749276702
BCR	NIH	0.118466667	0.152762529	0.775495585	0.444547689	0.682573223
SAG	UCL	0.232181084	0.301832661	0.769237773	0.444984634	0.749826462
PPY	UCL	-0.369765679	0.477072916	-0.775071623	0.445073182	0.749826462
SH2B3	NIH	0.160293333	0.207062438	0.774130426	0.44534139	0.683099969
SEMA3G	NIH	-0.100266667	0.129526399	-0.77410217	0.445357826	0.683099969
IL15	UCL	-0.114911618	0.148438867	-0.774134301	0.445660161	0.750266268
TEX33	UCL	0.176933239	0.228276903	0.775081652	0.44606886	0.750266268
LCN2	UCL	-0.11911591	0.154101025	-0.772972859	0.446104268	0.750266268
PDGFRA	NIH	0.090233333	0.116954861	0.771522725	0.446859866	0.685044606
CILP	UCL	0.166615005	0.216578682	0.769304733	0.4475668	0.75171379

COL3A1	UCL	0.090691152	0.117592006	0.771235697	0.447640049	0.75171379
TAFA5	UCL	-0.137499927	0.178726619	-0.769330991	0.447882744	0.75171379
KIAA1549	UCL	0.202046385	0.264411327	0.764136649	0.447993645	0.75171379
MCEE	NIH	-0.110886667	0.1440958	-0.76953434	0.448019796	0.686008291
ANXA5	NIH	0.46756	0.607631009	0.76948015	0.448051434	0.686008291
CD80	NIH	0.13164	0.171179878	0.76901562	0.448322689	0.686008291
BTN1A1	NIH	-0.08008	0.104157225	-0.768837689	0.448426616	0.686008291
SKAP1	UCL	-0.185053696	0.240898985	-0.768179644	0.448477358	0.752093698
IL17A	UCL	-0.107404457	0.139866864	-0.767904946	0.448736538	0.752096847
GALNT10	UCL	0.112335002	0.146281381	0.767937804	0.449059134	0.752206217
POF1B	UCL	-0.136173445	0.177446754	-0.767404544	0.44965118	0.752766552
LAMTOR5	NIH	0.111893333	0.145973652	0.766531027	0.449775203	0.687431898
PLG	NIH	0.067433333	0.087982291	0.766442118	0.449827232	0.687431898
MRPS16	UCL	-0.079471407	0.103992369	-0.764204219	0.450050953	0.752888314
GFRA3	UCL	0.103873028	0.135717254	0.765363466	0.450739775	0.752888314
ENPP5	UCL	-0.139883744	0.182635645	-0.765916992	0.450742374	0.752888314
WFDC1	UCL	-0.210893304	0.276597822	-0.762454681	0.450807928	0.752888314
LAIR2	UCL	0.212943847	0.278283322	0.765205207	0.451011782	0.752888314
RRM2	NIH	0.129693333	0.169742369	0.764059875	0.451222645	0.688767546
CCL7	NIH	0.170533333	0.223281053	0.763760879	0.451397965	0.688767546
COX5B	NIH	0.250673333	0.328216281	0.763744359	0.451407653	0.688767546
PAMR1	UCL	0.098115023	0.128488047	0.763612068	0.451779831	0.752922149
C2CD2L	UCL	0.119763761	0.158096992	0.757533459	0.451906258	0.752922149
BOLA1	UCL	-0.20251031	0.265828636	-0.76180773	0.452307154	0.752922149
FGD3	NIH	0.197593333	0.259314192	0.761984261	0.45244055	0.68945737
CIT	NIH	0.230666667	0.302815575	0.761739771	0.452584138	0.68945737
KHDC3L	UCL	-0.134523569	0.177272416	-0.758852232	0.452635542	0.752922149
MERTK	UCL	0.056683426	0.074354665	0.762338523	0.452854904	0.752922149
CC2D1A	NIH	0.265146667	0.348308876	0.761240051	0.452877706	0.68945737
GATA3	NIH	0.13642	0.179228434	0.761151549	0.45292971	0.68945737
CPXM1	UCL	-0.161559135	0.212458584	-0.76042649	0.45297075	0.752922149
COPE	UCL	-0.250610833	0.328234227	-0.763512189	0.452992504	0.752922149
FASLG	NIH	0.139833333	0.183757483	0.760966744	0.453038313	0.68945737

XPNPEP2	UCL	-0.300494236	0.394644219	-0.761430731	0.453119051	0.752922149
CD3E	UCL	0.062316216	0.082526583	0.755104762	0.453350319	0.752922149
IL10RA	UCL	-0.097154282	0.127926761	-0.759452372	0.454273097	0.75402627
LILRB1	NIH	0.1081	0.142533342	0.758419035	0.454537079	0.691175451
DHRS4L2	UCL	-0.083549638	0.110371137	-0.756988106	0.454537578	0.754037083
DKKL1	NIH	0.279933333	0.369205135	0.758205416	0.45466288	0.691175451
DENR	UCL	-0.167535959	0.221613744	-0.755981808	0.454868897	0.754158699
LILRA5	NIH	-0.087086667	0.11491381	-0.757843347	0.454876152	0.691175451
STEAP4	NIH	0.257033333	0.339385297	0.757349642	0.455167058	0.691258383
C1QL2	UCL	0.13536531	0.178936341	0.756499819	0.455641068	0.754760024
CTSB	UCL	0.126779542	0.167833604	0.755388306	0.456026944	0.754760024
BHLHE40	UCL	-0.361850249	0.481704388	-0.751187364	0.456040848	0.754760024
CDH23	UCL	-0.116359983	0.153879103	-0.756177937	0.456278006	0.754760024
GUK1	NIH	0.119306667	0.157940919	0.755387948	0.456324038	0.692655844
AREG	UCL	-0.141751429	0.187778753	-0.754885348	0.456522656	0.754760024
CCN4	UCL	-0.098498658	0.130525367	-0.754632302	0.45724271	0.755523144
SRPK2	UCL	-0.169349604	0.224880851	-0.75306369	0.457758828	0.755948619
CIAPIN1	NIH	0.17818	0.236863031	0.752249091	0.458178915	0.695110647
PI16	UCL	-0.065824684	0.087662735	-0.750885581	0.45846997	0.756457702
PLXNA4	UCL	-0.257017401	0.344070967	-0.746989505	0.458795707	0.756457702
NFAT5	NIH	0.205453333	0.273510439	0.751171818	0.458816546	0.695601684
DKKL1	UCL	0.229535185	0.305411686	0.75155993	0.458843484	0.756457702
TAFA5	NIH	-0.1014	0.135038164	-0.750898836	0.458978205	0.695601684
FUT3_FUT5	NIH	-0.185826667	0.247814646	-0.749861518	0.45959281	0.696105609
MTHFD2	NIH	0.083053333	0.110806555	0.749534479	0.459786679	0.696105609
MYH7B	UCL	-0.104443895	0.14051804	-0.743277487	0.460420721	0.757998891
MUC16	UCL	-0.479190255	0.639374454	-0.749467314	0.460434172	0.757998891
DEFB103A_DEFB103B	UCL	0.20377766	0.272597099	0.747541556	0.460556288	0.757998891
LMOD2	NIH	0.35648	0.476733258	0.747755677	0.460842	0.697072034
RALB	NIH	-0.118326667	0.158263682	-0.747655213	0.460901646	0.697072034
PNLIP	UCL	-0.14807623	0.198228342	-0.746998277	0.461129668	0.758515487
PAFAH1B3	NIH	0.247133333	0.331028566	0.746561955	0.461551007	0.697693383

ITIH5	UCL	-0.183305402	0.245988209	-0.745179629	0.46161839	0.75881664
REG1A	UCL	-0.197128928	0.263903958	-0.746972229	0.461831954	0.75881664
ROR1	UCL	0.078079704	0.104754877	0.745356269	0.462556844	0.759580705
CAMKK1	NIH	0.193026667	0.259230876	0.744612946	0.462709994	0.699084057
SMPD3	UCL	0.247809501	0.33297251	0.744234114	0.463039131	0.759861498
GABRA4	UCL	-0.237366527	0.320098349	-0.74154249	0.463339308	0.759861498
RIDA	UCL	0.170817743	0.229346594	0.744801745	0.463507715	0.759861498
YY1	NIH	-0.1863	0.250671999	-0.743202275	0.463549923	0.699825127
ITGAM	UCL	-0.122862749	0.165261686	-0.743443635	0.463866735	0.760023805
LGALS7_LGALS7B	NIH	0.14136	0.190482939	0.742113706	0.46419868	0.699825127
ADGRE1	NIH	0.179866667	0.242444413	0.741888271	0.4643331	0.699825127
RAP1A	NIH	0.203273333	0.274045734	0.741749671	0.464415754	0.699825127
SLITRK1	UCL	0.115744385	0.156294906	0.740551235	0.464689158	0.760944767
VSIG2	NIH	0.207713333	0.280225987	0.741235086	0.464722702	0.699825127
ERI1	NIH	0.148413333	0.200266398	0.741079556	0.464815498	0.699825127
MMP7	NIH	0.047346667	0.063897417	0.740979353	0.46487529	0.699825127
AIF1L	NIH	0.179853333	0.243196989	0.739537666	0.46573605	0.700744611
CLSTN2	UCL	0.10886591	0.147070067	0.74023159	0.465751041	0.762206396
RIPK4	NIH	-0.31404	0.424864049	-0.739154092	0.46596522	0.700744611
NPC2	UCL	-0.091070834	0.123812068	-0.735557	0.465981125	0.762206396
GNGT1	NIH	0.18062	0.244742632	0.737999744	0.466655295	0.701421757
NPTX1	NIH	-0.101306667	0.137470909	-0.736931669	0.467294328	0.702021525
PIK3IP1	UCL	-0.081283705	0.110278113	-0.737079217	0.467581725	0.764045328
DCTPP1	UCL	0.0944778	0.128220858	0.736836436	0.46782418	0.764045328
DEFA1_DEFA1B	UCL	-0.142730149	0.193857341	-0.736263835	0.467889544	0.764045328
MAEA	NIH	0.137526667	0.186924647	0.73573319	0.46801199	0.702738743
PRL	UCL	0.284770418	0.386979542	0.735879774	0.468287365	0.76426799
ALPI	NIH	-0.4022	0.547041848	-0.735227116	0.468315227	0.702833267
CLIC5	UCL	-0.088386939	0.120935394	-0.730860799	0.468728257	0.764460368
EFEMP1	UCL	0.104966967	0.142865344	0.734726592	0.468928306	0.764460368
KIF22	NIH	0.264486667	0.360336173	0.733999767	0.469051122	0.703576683
SCGB2A2	NIH	-0.11094	0.151425369	-0.732638137	0.469868318	0.70434008
ARMCX2	UCL	0.217489462	0.296861925	0.732628349	0.469887156	0.765596519

NFKBIE	UCL	-0.259753397	0.354965112	-0.731771625	0.470242933	0.765749356
PGLYRP2	NIH	0.092073333	0.125786565	0.731980664	0.470263204	0.70434008
PLCB2	NIH	0.17894	0.244542023	0.731735175	0.470410697	0.70434008
CD300E	NIH	0.125173333	0.171165732	0.731299027	0.470672806	0.70434008
ATP6V1F	NIH	0.274986667	0.376118815	0.731116487	0.470782532	0.70434008
GSTA3	UCL	0.253027014	0.345589439	0.732160724	0.470998423	0.766404811
IL15	NIH	-0.095086667	0.130122607	-0.730746708	0.471004853	0.70434008
LILRA3	UCL	-0.388612644	0.531786323	-0.730768407	0.471169841	0.766404811
CDAN1	NIH	0.046726667	0.06400068	0.730096405	0.471395981	0.704564765
PMM2	NIH	0.1822	0.249716091	0.729628592	0.471677467	0.704625429
LEG1	UCL	0.165507813	0.22761364	0.727143652	0.473171855	0.768218728
NPTN	UCL	0.362196764	0.498118615	0.727129549	0.473176016	0.768218728
CRX	NIH	-0.480853333	0.661559983	-0.726847671	0.473352774	0.706767159
CX3CL1	UCL	-0.089157572	0.122732357	-0.726439013	0.473659239	0.768218728
VSNL1	UCL	0.141568647	0.19468338	0.727173769	0.473989472	0.768218728
GP1BA	UCL	-0.086521242	0.119134673	-0.72624736	0.474006929	0.768218728
IL22	UCL	-0.244621769	0.337031032	-0.725813786	0.474114656	0.768218728
EVPL	NIH	-0.11442	0.157730416	-0.72541494	0.474217239	0.707696644
FLT1	UCL	-0.07169705	0.098985249	-0.724320544	0.474258918	0.768218728
DAG1	UCL	-0.109698768	0.151906412	-0.722147056	0.474387549	0.768218728
CA12	UCL	0.14496488	0.200054054	0.724628556	0.474762266	0.768399836
HS1BP3	NIH	0.123506667	0.170746908	0.723331791	0.475475774	0.708696375
CA9	NIH	-0.15822	0.21879213	-0.723152153	0.475584393	0.708696375
RNF31	NIH	-0.13088	0.180997685	-0.723103172	0.475614012	0.708696375
SPTBN2	UCL	-0.086326526	0.11980986	-0.720529398	0.475615641	0.769355018
CEP43	NIH	0.206086667	0.285338667	0.722252855	0.476128373	0.708771339
FLT4	NIH	0.08626	0.119437481	0.722218848	0.476148951	0.708771339
CASP4	NIH	0.67512	0.935811732	0.72142716	0.476628147	0.709123769
TNFSF13B	NIH	0.08736	0.121181939	0.720899504	0.476947683	0.709238421
RAB39B	NIH	-0.233133333	0.323809247	-0.719971204	0.477510141	0.709714006
TGFBR3	UCL	-0.070039364	0.097318768	-0.719690202	0.477869748	0.772573713
MRPL58	NIH	0.102666667	0.14279716	0.71896855	0.47811808	0.710245017
DPEP2	NIH	-0.04764	0.066297308	-0.71858121	0.478353054	0.710245017

C7	NIH	-0.100346667	0.139862459	-0.717466772	0.479029485	0.710616281
TAX1BP1	NIH	0.119413333	0.166464083	0.717351944	0.479099213	0.710616281
NFKB2	UCL	0.074899497	0.104882989	0.714124353	0.479195894	0.774217639
ENPP7	NIH	-0.232766667	0.324653846	-0.716968764	0.479331939	0.710616281
IL24	UCL	-0.094345756	0.131913045	-0.715211716	0.479416328	0.774217639
CD40LG	NIH	0.102613333	0.143223491	0.716456026	0.479643454	0.710717884
EBI3_IL27	NIH	0.081146667	0.113465166	0.715168093	0.480426453	0.710998593
ATP1B1	NIH	0.130446667	0.182412529	0.715119007	0.480456309	0.710998593
KDR	NIH	0.071946667	0.100632442	0.714945051	0.480562126	0.710998593
AFP	UCL	-0.207800556	0.290518072	-0.715275833	0.480643548	0.775259149
EIF5A	UCL	0.079790937	0.111939548	0.712803815	0.480820847	0.775259149
FGFR4	NIH	0.178386667	0.249685602	0.714445149	0.480866288	0.711088925
AKAP12	UCL	0.065819873	0.092423897	0.712152102	0.481539279	0.775259149
ARNT	UCL	-0.307068174	0.430221367	-0.713744593	0.481653255	0.775259149
NECTIN4	NIH	0.074453333	0.104411334	0.713077117	0.481699224	0.711960703
PILRB	UCL	-0.23242879	0.325543065	-0.713972482	0.481738816	0.775259149
ATP5PO	UCL	0.224592959	0.315876912	0.711014164	0.481916779	0.775259149
GART	UCL	0.056260186	0.07907926	0.711440466	0.481917849	0.775259149
CSF3	NIH	-0.137733333	0.193316466	-0.712475954	0.482065508	0.712142228
TPT1	UCL	-0.326914419	0.459043385	-0.712164534	0.482775505	0.775744452
GAST	NIH	0.16612	0.233600025	0.71113006	0.482886129	0.712671709
CD177	NIH	0.439973333	0.618731803	0.711088926	0.482911223	0.712671709
NRXN3	UCL	0.060962582	0.086180752	0.707380485	0.483267352	0.775744452
SPINK5	UCL	-0.095367586	0.13424687	-0.710389642	0.483408042	0.775744452
SCGB3A2	UCL	0.252922909	0.35582504	0.710806943	0.48340998	0.775744452
NELL2	UCL	0.084908827	0.119428264	0.710960909	0.48354649	0.775744452
CABP2	NIH	-0.140066667	0.19728299	-0.709978427	0.483588941	0.713311978
AHCY	NIH	0.136413333	0.192420305	0.70893419	0.484226716	0.713892714
LGALS9	UCL	0.11682364	0.164684454	0.709378678	0.484349593	0.776268084
NCF2	UCL	-0.205143257	0.289147566	-0.709475994	0.484485494	0.776268084
UPK3A	UCL	-0.177521286	0.251889742	-0.704757904	0.484669604	0.776268084
AMY2A	UCL	0.135348823	0.191391013	0.707184841	0.485722791	0.776826395
COL5A1	UCL	-0.135541154	0.191695844	-0.707063602	0.485913893	0.776826395

CXADR	UCL	0.161266598	0.22839314	0.706092127	0.486045736	0.776826395
CBX2	NIH	-0.127953333	0.181320822	-0.70567369	0.486221183	0.716430468
STAB2	UCL	0.059167839	0.083961487	0.704702128	0.486498265	0.776826395
CST1	NIH	-0.309013333	0.438282344	-0.7050554	0.486599922	0.716430468
PCNA	UCL	0.105554046	0.149972841	0.703821076	0.486614278	0.776826395
SHPK	NIH	-0.170146667	0.24137016	-0.704920055	0.486682851	0.716430468
IFNL1	UCL	-0.225494989	0.322332307	-0.699573031	0.487089863	0.776826395
SPINK4	UCL	0.216486738	0.307114498	0.704905626	0.487114863	0.776826395
NPY	UCL	0.228720912	0.324375605	0.705111322	0.487144297	0.776826395
MORN4	UCL	-0.236509988	0.335841809	-0.704230329	0.487626095	0.777170706
MMUT	NIH	0.19656	0.279477253	0.703313054	0.487668116	0.717519738
TGM2	UCL	0.163596178	0.233243148	0.701397575	0.488094482	0.777416759
FTCD	UCL	0.258411609	0.367389887	0.70337159	0.488312408	0.777416759
IL17F	NIH	0.30938	0.440689583	0.702036108	0.488451829	0.718311514
IFNW1	NIH	-0.244986667	0.349266626	-0.701431653	0.488823058	0.718496203
KRT5	NIH	0.17202	0.245448169	0.700840429	0.489186315	0.718668996
COX6B1	UCL	0.137990802	0.19777324	0.697722311	0.489469028	0.778162557
PCDH12	UCL	-0.05497251	0.078839581	-0.697270442	0.489684974	0.778162557
DDAH1	UCL	0.136942446	0.195583457	0.700173974	0.489799325	0.778162557
CD36	UCL	-0.122976789	0.175953264	-0.698917348	0.489977964	0.778162557
CDH4	UCL	-0.102379251	0.147370613	-0.694706011	0.490111963	0.778162557
COPB2	UCL	-0.180708909	0.259946373	-0.69517765	0.49051205	0.778374985
TERF1	NIH	0.14054	0.201252884	0.698325398	0.490733302	0.720579773
GPI	UCL	-0.126677268	0.18200278	-0.696018313	0.491164311	0.778987131
AP3B1	NIH	0.123026667	0.176619747	0.696562353	0.491819395	0.721812208
UBE2B	UCL	-0.067515281	0.097235234	-0.694349963	0.491868599	0.779335838
FGF21	UCL	-0.321549957	0.463201166	-0.694190733	0.492060491	0.779335838
CRYBB2	NIH	0.201326667	0.28925938	0.696007391	0.492161551	0.721952125
PDGFRB	UCL	-0.101460948	0.145686656	-0.696432681	0.492184043	0.779335838
DCUN1D2	NIH	0.220846667	0.317629431	0.695296611	0.492599972	0.722233041
CLEC4A	UCL	-0.114837883	0.16506278	-0.69572246	0.49260395	0.779578423
F12	UCL	-0.118972435	0.171261739	-0.694681926	0.493174738	0.779734384
STX1B	UCL	-0.106712703	0.15441841	-0.691062047	0.493336716	0.779734384

CLUL1	UCL	0.151129507	0.217697635	0.694217496	0.493502775	0.779734384
ANPEP	NIH	-0.071073333	0.102638567	-0.69246225	0.494350444	0.724436397
APP	UCL	0.156708487	0.226759663	0.69107744	0.49481783	0.781389799
CCL11	NIH	0.086873333	0.125704215	0.691093239	0.495197186	0.725313855
MRPL24	UCL	-0.082539075	0.120040219	-0.687595171	0.495559602	0.782097456
PLIN3	UCL	0.09824379	0.142350593	0.690153706	0.49580109	0.782097456
IMPG1	NIH	0.132126667	0.191548201	0.689782865	0.496008425	0.725749596
PRSS22	NIH	-0.062766667	0.091027476	-0.689535399	0.496161712	0.725749596
RBM19	NIH	-0.178566667	0.259013529	-0.689410579	0.49623904	0.725749596
ACAN	UCL	0.074608859	0.108158939	0.68980761	0.496252409	0.782202888
MSR1	UCL	-0.183144914	0.265894843	-0.68878701	0.496864312	0.782202888
CENPF	UCL	-0.1132511	0.165147845	-0.685755846	0.497140881	0.782202888
NCLN	UCL	0.226861312	0.329874717	0.687719611	0.497460298	0.782202888
LCN15	UCL	0.153416397	0.223108308	0.687631933	0.497478962	0.782202888
GGT5	UCL	-0.071909288	0.104715763	-0.686709294	0.497735667	0.782202888
WASF3	UCL	-0.673852237	0.984815815	-0.684241892	0.497875597	0.782202888
PTK7	UCL	-0.15453203	0.225629691	-0.684892266	0.498008749	0.782202888
Cholesterol	NIH	0.068669412	0.100106945	0.685960515	0.498586486	0.723985021
TEK	NIH	0.070746667	0.103269461	0.685068613	0.498933144	0.729307629
YJU2	UCL	-0.082377916	0.121017692	-0.680709692	0.499118399	0.782621654
RAD51	NIH	-0.150753333	0.220238762	-0.68449955	0.499286842	0.729307629
LAT2	NIH	0.145293333	0.212328514	0.684285548	0.499419891	0.729307629
BMPER	UCL	0.066178727	0.096647264	0.684744961	0.499472876	0.782621654
PALLD	UCL	0.110676368	0.162289081	0.681970513	0.499585765	0.782621654
KLRD1	UCL	0.131807571	0.192595969	0.684373467	0.499595758	0.782621654
GALNT7	UCL	0.064206813	0.094000062	0.683050751	0.499614097	0.782621654
PCNA	NIH	-0.129026667	0.188733588	-0.683644434	0.499818601	0.729525652
TIA1	NIH	-0.14166	0.207651802	-0.682199713	0.500717727	0.730473491
KLF4	UCL	-0.11438271	0.168758385	-0.677789786	0.501152788	0.784371208
TTR	NIH	-0.091766667	0.134768003	-0.680923249	0.501512889	0.730784499
MORF4L1	NIH	-0.119753333	0.175870872	-0.680916243	0.501517255	0.730784499
SPINK2	NIH	-0.145606667	0.214032022	-0.680303186	0.501899406	0.730784499
CREB3	NIH	-0.152606667	0.224337879	-0.680253675	0.501930276	0.730784499

IVD	UCL	-0.240509287	0.355211167	-0.677088194	0.501950781	0.784371208
TP53BP1	UCL	-0.262693505	0.38602641	-0.680506561	0.502130568	0.784371208
VNN1	UCL	-0.247410178	0.363782576	-0.680104531	0.502602162	0.784371208
LEFTY2	UCL	0.195515171	0.287664571	0.679663713	0.502730083	0.784371208
CTAG1A_CTAG1B	UCL	-0.109763738	0.162426017	-0.675776823	0.502881104	0.784371208
IL5	UCL	0.307700926	0.453923753	0.677869187	0.502896065	0.784371208
SERPINE2	NIH	0.118046667	0.173943957	0.678647701	0.502932169	0.731878903
POLR2F	UCL	0.062996277	0.092916621	0.677987168	0.50293468	0.784371208
CDSN	UCL	-0.131082584	0.193188403	-0.67852201	0.503146088	0.784371208
ARG2	NIH	0.15874	0.234262862	0.677614875	0.50357709	0.732211142
PCOLCE	NIH	-0.10818	0.159679912	-0.677480334	0.503661134	0.732211142
PALM3	UCL	0.159578596	0.235260798	0.678305087	0.503723961	0.784853485
NXPE4	NIH	-0.173433333	0.256200632	-0.676943425	0.503996606	0.73233486
PDE5A	NIH	0.14148	0.209227123	0.67620296	0.504459467	0.732643467
IGSF9	UCL	-0.146152363	0.216245216	-0.675864032	0.504576089	0.785762338
JCHAIN	UCL	-0.167833088	0.249044828	-0.673907142	0.505221387	0.785831269
HEPH	NIH	-0.08466	0.125462935	-0.674780961	0.505349015	0.733236355
NPY	NIH	-0.140033333	0.207533981	-0.674748938	0.505369057	0.733236355
CCN5	UCL	0.112107545	0.166018773	0.67527029	0.505575095	0.785831269
ST8SIA1	NIH	-0.072493333	0.107574165	-0.673891667	0.505905765	0.733651147
RNASE3	UCL	-0.257167359	0.383178907	-0.671141742	0.506014718	0.785831269
IL1RAP	UCL	0.134131143	0.198923558	0.67428486	0.506091253	0.785831269
PAPPA	UCL	-0.122719091	0.18264553	-0.671897592	0.506107668	0.785831269
GOLM2	UCL	-0.054564224	0.081048865	-0.673226251	0.506233418	0.785831269
GIP	UCL	-0.155243578	0.231309876	-0.671149807	0.506871564	0.78614872
MFAP4	UCL	0.107110894	0.159132344	0.673093171	0.506975825	0.78614872
DUSP3	NIH	0.407353333	0.606264777	0.671906647	0.507149731	0.735090665
SLC44A4	UCL	-0.219465829	0.327256048	-0.67062421	0.508219701	0.787019352
IL31	UCL	0.096920427	0.144817326	0.669259891	0.508219827	0.787019352
SAFB2	UCL	-0.07788828	0.116791652	-0.666899381	0.508612962	0.787019352
DIPK2B	UCL	0.068662456	0.102579905	0.669355811	0.508660744	0.787019352
HMCN2	NIH	-0.226733333	0.338810601	-0.669203775	0.508846277	0.737111589
CNDP1	UCL	-0.102384028	0.152957492	-0.669362622	0.508883536	0.787019352

CDH22	NIH	-0.15616	0.233463945	-0.668882726	0.509048003	0.737111589
LRRC25	NIH	0.08732	0.1306943	0.668124011	0.509524903	0.737437082
MEGF11	UCL	0.076664753	0.115069755	0.666245902	0.509600522	0.787590667
IGFBP4	UCL	-0.125166828	0.187359097	-0.668058452	0.509791838	0.787590667
NT5C	UCL	-0.184376687	0.276932128	-0.665782943	0.510889596	0.788869673
ANPEP	UCL	-0.076866126	0.115490589	-0.665561811	0.511700306	0.789704327
PLA2G7	UCL	-0.097917933	0.147592604	-0.663433874	0.512107683	0.789915968
PKN3	NIH	0.162226667	0.244408507	0.663752128	0.512277704	0.740554947
Free_Testosterone	NIH	0.101571241	0.153221435	0.662904905	0.513011457	0.734640795
ACADM	NIH	0.251946667	0.380398824	0.662322411	0.513179712	0.740554947
CEACAM1	UCL	0.078051708	0.117781037	0.662684839	0.513589305	0.790632524
EZR	UCL	0.059924291	0.090682078	0.660817359	0.513823982	0.790632524
CLGN	UCL	0.129345433	0.195935674	0.66014233	0.513886681	0.790632524
LSM8	NIH	0.22372	0.338381315	0.661147616	0.513921542	0.740554947
FGFBP3	UCL	0.083911896	0.127036619	0.660533132	0.514011937	0.790632524
SFTPA2	NIH	-0.132133333	0.199907216	-0.660973306	0.51403166	0.740554947
GBA	NIH	-0.179473333	0.2715655	-0.660884146	0.514087992	0.740554947
VEGFB	NIH	0.0783	0.118502914	0.660743247	0.514177018	0.740554947
TP73	NIH	0.126566667	0.191639762	0.660440535	0.514368315	0.740554947
NPDC1	NIH	0.0881	0.133404295	0.660398527	0.514394864	0.740554947
DCTD	NIH	0.13896	0.210419289	0.66039573	0.514396632	0.740554947
CALB1	UCL	0.11300716	0.171148077	0.660288809	0.514497862	0.790632524
TP53BP1	NIH	0.174733333	0.264738359	0.660022725	0.51463241	0.740554947
GIPR	UCL	0.138786013	0.210870819	0.658156559	0.514638354	0.790632524
GAS6	UCL	0.071635279	0.108574957	0.659777179	0.514885025	0.790632524
COMMD9	NIH	0.068726667	0.104205243	0.659531757	0.514942844	0.740554947
IL36G	UCL	-0.228177264	0.346025626	-0.659423023	0.514973334	0.790632524
SIGLEC5	NIH	0.27718	0.420435203	0.659269248	0.515108867	0.740554947
BCL7A	NIH	-0.232726667	0.353010462	-0.659262803	0.515112943	0.740554947
CES3	UCL	0.207475522	0.314921726	0.658816158	0.515215882	0.790632524
IL17A	NIH	0.291946667	0.442955714	0.659087709	0.515223698	0.740554947
RFC4	UCL	0.04851458	0.073809458	0.657294896	0.515277098	0.790632524
PFDN6	UCL	0.164309787	0.249961909	0.657339303	0.515673125	0.790670171

PILRB	NIH	0.1353	0.205529556	0.658299482	0.515722448	0.740636365
CPVL	NIH	0.107533333	0.163375389	0.658197872	0.515786761	0.740636365
LRRC38	UCL	-0.095048801	0.145232224	-0.654460823	0.516168877	0.790670171
SNU13	NIH	0.149006667	0.22663064	0.657486854	0.516236914	0.740919025
SLK	UCL	0.165254627	0.251707021	0.656535628	0.516404697	0.790670171
PDCD1LG2	UCL	-0.08613261	0.131049462	-0.657252681	0.516412526	0.790670171
CPXM1	NIH	0.132826667	0.202187958	0.656946478	0.516579176	0.741046635
ATXN2L	UCL	-0.082614154	0.126017677	-0.65557591	0.516654131	0.790670171
GIMAP7	NIH	0.087186667	0.13287261	0.656167337	0.517072883	0.741391266
CCL28	UCL	-0.246199575	0.375352397	-0.65591582	0.517361748	0.79133877
ACOT13	NIH	0.102326667	0.156212456	0.655048064	0.517782568	0.742045082
TAGLN3	UCL	-0.110775003	0.17026747	-0.650594053	0.517969088	0.791853371
TOP1	NIH	-0.427906667	0.654192606	-0.654098904	0.518384808	0.74254435
AKR1B10	UCL	-0.201616358	0.308016773	-0.654562918	0.518428132	0.792140842
CD46	NIH	0.059906667	0.091660249	0.653573028	0.51871864	0.742658845
GPR158	NIH	-0.08604	0.131874983	-0.652436101	0.519440772	0.743097836
CHRDL2	NIH	-0.134266667	0.205838792	-0.652290394	0.519533359	0.743097836
ATP6V1F	UCL	-0.193288937	0.297753914	-0.649156663	0.519670894	0.793624882
HMOX1	UCL	0.155027044	0.2378006	0.651920322	0.520077264	0.793830727
AMBN	UCL	0.060645237	0.093287524	0.650089463	0.520584345	0.794190001
CFB	NIH	0.081406667	0.125144286	0.650502466	0.520670199	0.744123738
CRX	UCL	0.267921087	0.412000542	0.650293045	0.520910434	0.794272925
WAS	NIH	0.241713333	0.372008626	0.649751958	0.521147805	0.744123738
ADAMTS15	NIH	-0.127533333	0.196327658	-0.649594331	0.521248146	0.744123738
CLUL1	NIH	-0.162653333	0.250404323	-0.6495628	0.521268219	0.744123738
RALB	UCL	-0.093325825	0.144367028	-0.646448334	0.521580668	0.794565062
FXYD5	UCL	0.163329046	0.252120645	0.647820991	0.521645691	0.794565062
CRTAP	NIH	-0.070073333	0.108104603	-0.648199349	0.522136605	0.744484673
IL1R2	NIH	-0.06568	0.101345003	-0.648083261	0.522210579	0.744484673
CASP1	UCL	0.174797664	0.271443806	0.643955249	0.522233252	0.795045727
PFDN2	NIH	-0.210993333	0.32574662	-0.647722249	0.522440657	0.744484673
MZB1	NIH	0.114046667	0.176115428	0.647567721	0.522539157	0.744484673
SLC12A2	NIH	0.121726667	0.188263798	0.646575007	0.523172177	0.74494039

ADGRD1	NIH	-0.09866	0.152661254	-0.646267456	0.523368376	0.74494039
ICAM5	UCL	0.114798903	0.177623262	0.646305563	0.523549043	0.796219689
ERC2	UCL	0.145649781	0.225675868	0.645393689	0.523712936	0.796219689
GADD45B	UCL	0.082194418	0.12765023	0.643903406	0.523821574	0.796219689
PLEKHO1	NIH	0.085153333	0.13196824	0.645256264	0.524013736	0.745288792
AIDA	NIH	0.185106667	0.286949034	0.64508552	0.524122751	0.745288792
CES2	NIH	0.148173333	0.229941779	0.644395002	0.524563748	0.745553432
MOCS2	UCL	-0.120089748	0.187078546	-0.641921537	0.524651521	0.797066734
F10	UCL	-0.059669754	0.092539223	-0.644805006	0.524988356	0.797164138
CTAG1A_CTAG1B	NIH	-0.153513333	0.23879684	-0.642861661	0.525543727	0.746583488
TNFRSF17	NIH	-0.108593333	0.169131834	-0.642063242	0.526054399	0.746793417
ITGAL	UCL	-0.131797404	0.206311432	-0.638827441	0.526147768	0.798509827
MAPRE3	UCL	-0.271747829	0.423380842	-0.641851974	0.526739051	0.798571591
PAEP	UCL	-0.343221087	0.535497368	-0.640938887	0.527000357	0.798571591
PROK1	UCL	0.129598963	0.202224959	0.640865319	0.527008073	0.798571591
HTRA2	NIH	0.100473333	0.156849771	0.640570481	0.52700989	0.746793417
ECHS1	NIH	0.212073333	0.331231959	0.640256254	0.52721114	0.746793417
NOP56	NIH	0.21194	0.331096674	0.640115159	0.52730152	0.746793417
GALNT3	UCL	-0.104062676	0.162824433	-0.639109711	0.52748012	0.798872741
APCS	NIH	-0.109786667	0.171636201	-0.639647498	0.527601143	0.746793417
LYAR	NIH	0.17124	0.267721683	0.639619466	0.527619105	0.746793417
PCDH12	NIH	0.04592	0.071833606	0.639255117	0.527852606	0.746793417
CHGA	NIH	0.2516	0.393627368	0.639183198	0.527898703	0.746793417
FN1	NIH	-0.18652	0.291879669	-0.639030463	0.527996608	0.746793417
SUSD5	UCL	-0.1079108	0.168817083	-0.6392173	0.528168929	0.799137888
TXK	UCL	-0.189827036	0.296853213	-0.639464313	0.528201984	0.799137888
ESYT2	NIH	0.210733333	0.329970171	0.638643586	0.528244643	0.746793417
RASGRF1	NIH	0.410093333	0.642658833	0.638119812	0.528580545	0.746862136
NPL	NIH	0.17596	0.275898147	0.637771591	0.528803926	0.746862136
GPR37	UCL	-0.209469347	0.328354463	-0.637936652	0.528883326	0.799165063
NAPRT	UCL	-0.055056822	0.086539147	-0.636207128	0.529132379	0.799165063
IL2RB	UCL	0.151365239	0.237632538	0.636971856	0.529133086	0.799165063
DPP4	UCL	-0.089735762	0.14079545	-0.637348451	0.529398731	0.799165063

TGFBR3	NIH	0.10996	0.172709615	0.636675612	0.52950732	0.747370503
PPP1R12B	UCL	-0.210507538	0.330086544	-0.637734381	0.529586975	0.799165063
CCL17	NIH	-0.18018	0.283151695	-0.636337353	0.529724515	0.747370503
SPARC	UCL	-0.253303987	0.399111338	-0.634669985	0.529982665	0.7993495
SMARCA2	NIH	-0.150766667	0.23709886	-0.635881028	0.530017594	0.747370503
KLB	NIH	-0.253933333	0.399650049	-0.635389221	0.530333559	0.747370503
ADCYAP1R1	NIH	-0.06726	0.105925702	-0.63497337	0.530600805	0.747370503
IL13	UCL	-0.098330395	0.154992459	-0.634420513	0.530671959	0.799976346
MORN4	NIH	-0.239153333	0.376723948	-0.634823814	0.530696935	0.747370503
PPIF	NIH	0.08722	0.137478614	0.634425947	0.530952715	0.747370881
CCL27	UCL	-0.147266298	0.23232235	-0.633887779	0.531352841	0.800589873
CA11	NIH	0.041193333	0.065055096	0.633206861	0.53173685	0.748114616
BEX3	UCL	-0.103919722	0.165253498	-0.628850363	0.53200386	0.800969941
IL31RA	UCL	0.110327592	0.174593032	0.631912918	0.532153139	0.800969941
TRIM40	NIH	0.19888	0.314427994	0.632513656	0.532183007	0.748382353
PAEP	NIH	-0.33298	0.526929585	-0.631925041	0.532562004	0.748555435
ANGPTL1	UCL	0.082132687	0.129973209	0.631920127	0.532636779	0.801285284
ABCA2	UCL	-0.123384014	0.195372792	-0.6315312	0.533260173	0.801810435
CCL22	NIH	-0.090733333	0.143913174	-0.630472743	0.533497725	0.749510493
CD70	UCL	-0.130142031	0.206604203	-0.629909894	0.534072203	0.802522672
CLEC4G	UCL	0.10532512	0.167467682	0.628928031	0.534519751	0.802522672
PLG	UCL	-0.050348359	0.080011034	-0.629267694	0.534560623	0.802522672
TIMD4	NIH	-0.120646667	0.191923463	-0.628618642	0.534693602	0.75082995
ROBO2	NIH	0.06854	0.109108432	0.628182432	0.53497516	0.750864849
DKK3	UCL	0.083144093	0.13240207	0.627966715	0.535199193	0.802522672
ISM1	UCL	-0.091996604	0.146704562	-0.627087548	0.535431091	0.802522672
CRLF1	UCL	-0.092187257	0.146790621	-0.628018712	0.535612661	0.802522672
KLK12	NIH	-0.377833333	0.602896337	-0.626697013	0.535934537	0.751850609
PTN	UCL	-0.245619326	0.392257688	-0.626168291	0.536758936	0.802522672
SEPTIN9	UCL	-0.032331908	0.052040417	-0.621284574	0.536933309	0.802522672
MTR	NIH	0.180333333	0.288481403	0.625112509	0.536958915	0.751870334
FXYD5	NIH	0.139753333	0.223583187	0.625061909	0.536991645	0.751870334
GGH	NIH	0.070533333	0.112865991	0.62492991	0.537077031	0.751870334

NMI	UCL	-0.237307321	0.379987377	-0.624513695	0.537148833	0.802522672
FCRLB	UCL	0.243219638	0.389121304	0.625048372	0.537245866	0.802522672
NGF	UCL	0.027032844	0.043483415	0.621681722	0.537250812	0.802522672
NCR3LG1	UCL	0.096884495	0.154982983	0.625129887	0.537294343	0.802522672
SMOC1	UCL	0.071222328	0.113925576	0.6251654	0.537303069	0.802522672
GAGE2A	NIH	-0.107313333	0.171860821	-0.624419996	0.537406949	0.751870334
PTPRB	NIH	-0.065106667	0.104302232	-0.624211633	0.537541791	0.751870334
CDH4	NIH	-0.1532	0.245479263	-0.624085302	0.537623556	0.751870334
CDH23	NIH	-0.078546667	0.12589763	-0.623893132	0.537747945	0.751870334
GPHA2	UCL	0.127164942	0.203991641	0.623383101	0.538230501	0.803032646
FAP	UCL	0.076958897	0.123534093	0.622976982	0.538406782	0.803032646
SOST	UCL	-0.096555683	0.155231751	-0.622009884	0.538943251	0.803032646
FABP9	UCL	0.158577207	0.25484508	0.622249434	0.538973261	0.803032646
CD79B	UCL	-0.138290779	0.22208554	-0.622691503	0.539018149	0.803032646
CD84	NIH	0.062473333	0.100497329	0.621641728	0.539206387	0.752834833
FHIT	NIH	0.211993333	0.341272159	0.621185549	0.539502151	0.752834833
VNN1	NIH	0.169033333	0.272172174	0.621052956	0.539588134	0.752834833
IGSF21	UCL	0.159129474	0.256376477	0.620686717	0.539693529	0.803303584
TRPV3	NIH	0.121833333	0.196225332	0.620884837	0.539697164	0.752834833
FCAR	NIH	0.118993333	0.191664304	0.620842435	0.539724665	0.752834833
SERPINA12	UCL	0.215121756	0.346615596	0.62063496	0.53991499	0.803303584
ST6GAL1	UCL	-0.08896547	0.143273545	-0.620948343	0.540024476	0.803303584
MARCO	UCL	0.067645976	0.109081381	0.620142281	0.540306652	0.803314518
CACNB1	NIH	-0.17904	0.288940643	-0.619642837	0.540503002	0.753268279
SV2A	NIH	0.146913333	0.237121555	0.619569711	0.540550468	0.753268279
LTB	UCL	-0.097433332	0.156387362	-0.623025612	0.540730792	0.803536403
MMP15	NIH	-0.095953333	0.155284126	-0.61792107	0.541621173	0.754400919
MST1	UCL	0.169397783	0.274024766	0.618184208	0.541849589	0.804789811
ID4	NIH	-0.087113333	0.141254448	-0.616712144	0.542407015	0.755135896
SCGN	NIH	0.09756	0.158410277	0.615869131	0.542955355	0.755539683
BHMT2	UCL	0.055670883	0.090960873	0.612031099	0.542994261	0.80575714
DGKZ	UCL	-0.101989136	0.165713797	-0.6154535	0.543052195	0.80575714
EPHX2	NIH	-0.24348	0.395848755	-0.615083405	0.543466695	0.755891623

SCLY	UCL	0.148514381	0.24139008	0.615246417	0.543559501	0.806100671
TRAF3IP2	NIH	0.07586	0.123420397	0.614647188	0.543750689	0.755927169
IGLC2	UCL	0.041222193	0.0670926	0.614407447	0.543972234	0.806287025
DYNLT3	NIH	-0.092026667	0.149825199	-0.614226894	0.544024389	0.755948379
CD27	UCL	0.101894074	0.165885365	0.614243902	0.544443986	0.806287025
NT5E	UCL	0.169641099	0.276298005	0.613978733	0.544512688	0.806287025
TYRP1	NIH	0.11598	0.189230016	0.612904879	0.544885772	0.756785795
SPOCK1	UCL	0.0601588	0.098374268	0.61152983	0.545166461	0.806846363
CGB3_CGB5_CGB8	NIH	0.107226667	0.175077779	0.612451603	0.545181277	0.756836846
CD302	UCL	0.08780397	0.143664245	0.611174829	0.54624794	0.80696854
TPSAB1	UCL	-0.168151641	0.275248719	-0.610907989	0.54638355	0.80696854
EPHX2	UCL	-0.119573706	0.195543894	-0.611492914	0.54645577	0.80696854
ANK2	UCL	0.106562287	0.175020807	0.608854963	0.547145463	0.80696854
TARM1	NIH	-0.210886667	0.346069972	-0.609375802	0.547188699	0.759263256
MAPK13	UCL	0.105373346	0.173960803	0.605730395	0.547533565	0.80696854
CEP152	NIH	0.252773333	0.41527533	0.608688538	0.547637769	0.759526066
PF4	UCL	-0.313626133	0.516035647	-0.607760597	0.547745608	0.80696854
CRELD1	UCL	-0.095390437	0.156579782	-0.609212988	0.547751942	0.80696854
NENF	UCL	-0.268368445	0.440663862	-0.609009423	0.547774621	0.80696854
TRAF3	UCL	-0.073361409	0.120930602	-0.606640567	0.548261862	0.80696854
MORF4L1	UCL	0.069332795	0.114547478	0.605275614	0.548285915	0.80696854
BGN	UCL	0.275226044	0.454636192	0.60537645	0.548409022	0.80696854
RANGAP1	UCL	0.176430486	0.290623456	0.607075867	0.548561919	0.80696854
AHNAK	UCL	0.064486326	0.106442728	0.605831201	0.549799394	0.808144501
KDR	UCL	0.069780468	0.115309005	0.60516061	0.550237535	0.808144501
LYVE1	UCL	-0.057140436	0.094513261	-0.604575863	0.550343037	0.808144501
MTPN	NIH	0.13962	0.23094866	0.604549947	0.550346049	0.762201521
GUCA2A	NIH	-0.080553333	0.133328727	-0.604170871	0.550594461	0.762201521
SLURP1	UCL	-0.151383996	0.252157346	-0.600355287	0.550691451	0.808144501
HBEGF	UCL	-0.226878278	0.376213249	-0.60305765	0.550743704	0.808144501
LEG1	NIH	-0.127366667	0.210895403	-0.603932874	0.550750454	0.762201521
POSTN	NIH	0.119313333	0.197688965	0.603540684	0.55100756	0.762201521
LRG1	NIH	0.06092	0.100961316	0.603399427	0.551100179	0.762201521

MGLL	NIH	0.144766667	0.239997648	0.603200357	0.551230717	0.762201521
CRKL	NIH	0.1985	0.329211367	0.602956094	0.551390912	0.762201521
CRTAC1	UCL	-0.089570184	0.148551305	-0.602957906	0.551573073	0.808955389
KIRREL2	NIH	-0.113606667	0.188700849	-0.602046399	0.551987731	0.76266609
ARHGAP30	UCL	-0.118143187	0.195878353	-0.603145706	0.552115609	0.809000482
CYP24A1	UCL	-0.122076913	0.203317241	-0.600425781	0.552283164	0.809000482
ADGRB3	UCL	0.079800777	0.132758759	0.601096132	0.552650047	0.809000482
FCGR2A	NIH	-0.134846667	0.224358475	-0.601032194	0.552653509	0.763225455
ALPP	UCL	-0.377536422	0.627170874	-0.601967402	0.552710901	0.809000482
ACTA2	UCL	-0.110251881	0.183502641	-0.600819042	0.553052901	0.809095911
HTR1A	UCL	-0.325256324	0.541697444	-0.600439097	0.553341503	0.809113164
TOM1L2	NIH	0.156433333	0.260843683	0.599720612	0.553515116	0.764010639
LXN	UCL	0.065434702	0.109748932	0.596221771	0.553849054	0.809223703
QDPR	UCL	-0.097816727	0.163575757	-0.597990368	0.55409268	0.809223703
UNC79	NIH	0.108106667	0.180554682	0.598747513	0.554154815	0.764010639
SEPTIN3	NIH	-0.187993333	0.314018856	-0.598668932	0.554206489	0.764010639
BMP4	UCL	0.077950969	0.130473642	0.597446101	0.554247641	0.809223703
SPINK1	NIH	-0.118413333	0.197824688	-0.598577126	0.554266864	0.764010639
SELL	UCL	0.052322729	0.087701067	0.596603104	0.554856075	0.809707593
DYNC1H1	UCL	-0.079984906	0.134318515	-0.595486829	0.556181359	0.811236582
SH3GL3	NIH	-0.222126667	0.37308618	-0.595376293	0.556373944	0.76551328
RAB10	NIH	0.097806667	0.164306868	0.595268278	0.55644512	0.76551328
HDAC9	UCL	0.069772852	0.117944663	0.59157278	0.556517374	0.811321838
SERPING1	NIH	0.039613333	0.066562161	0.595132922	0.55653432	0.76551328
JAM2	NIH	-0.053713333	0.090370555	-0.594367638	0.557038785	0.76551328
KRT19	NIH	0.16402	0.276010106	0.594253602	0.557113975	0.76551328
TPSG1	UCL	-0.10644995	0.179499018	-0.593039178	0.557191174	0.811401807
GGA1	UCL	0.21801471	0.36637486	0.595059141	0.557245856	0.811401807
MAPKAPK2	NIH	0.183826667	0.309570372	0.593812209	0.557405062	0.76551328
SEMA7A	NIH	0.073186667	0.123259166	0.593762469	0.557437869	0.76551328
SCN2A	NIH	0.174953333	0.294661709	0.593743022	0.557450696	0.76551328
WFIKKN1	UCL	0.097178009	0.163615485	0.593941394	0.557451281	0.811401807
GZMH	UCL	-0.202219215	0.340971615	-0.593067593	0.557682597	0.811401807

SCARB2	UCL	-0.069305213	0.116991802	-0.592393759	0.558706231	0.812102662
PRELP	UCL	-0.056901856	0.096128404	-0.591935927	0.558719963	0.812102662
TJAP1	NIH	0.115913333	0.196055455	0.591227281	0.559111317	0.767433412
ACP5	UCL	0.071539552	0.121030062	0.591089118	0.559388376	0.812652809
CPVL	UCL	0.087958784	0.148959303	0.590488694	0.559654501	0.812652809
MPO	UCL	-0.12099022	0.205697243	-0.588195633	0.560219209	0.812951334
IQGAP2	UCL	-0.204478551	0.347764363	-0.587980173	0.560563043	0.812951334
ECSCR	UCL	0.446028178	0.757100416	0.589126844	0.560694454	0.812951334
CPA1	NIH	0.155166667	0.264023045	0.587701225	0.561443092	0.770272535
SYT1	UCL	0.073509951	0.125250923	0.586901468	0.561764582	0.813835614
TNFRSF11A	UCL	-0.114681911	0.195194248	-0.587527106	0.561861194	0.813835614
CNTN1	NIH	-0.070466667	0.12007616	-0.58684977	0.5620069	0.770684568
MAN2B2	NIH	0.154053333	0.262714046	0.58639169	0.562310347	0.770739346
FCER2	UCL	0.111196984	0.189556404	0.586616869	0.562398426	0.814210301
VSTM1	UCL	-0.15657753	0.267414099	-0.585524587	0.562829578	0.814431118
SRC	NIH	0.2229	0.380762982	0.585403546	0.562965208	0.771275519
LSM8	UCL	0.204583576	0.350397952	0.583860649	0.563331392	0.814753913
CDKL5	NIH	-0.143746667	0.2460199	-0.584288778	0.563704451	0.771926741
HSD17B14	UCL	0.177626492	0.303967174	0.584360771	0.564027831	0.815192131
EIF5A	NIH	0.090226667	0.154663599	0.583373639	0.564311681	0.772396662
AMY1A_AMY1B_AMY1C	UCL	0.089492798	0.153376953	0.583482696	0.564460536	0.815192131
VAT1	UCL	-0.03462176	0.059553364	-0.58135692	0.564471048	0.815192131
CD101	UCL	0.100241239	0.172215261	0.582069431	0.565525236	0.815914683
WNT9A	UCL	0.064354868	0.110623146	0.581748666	0.565529643	0.815914683
ANXA4	NIH	0.1142	0.196708103	0.580555646	0.56618361	0.774596379
PPBP	UCL	-0.283220887	0.489086982	-0.579080812	0.566368557	0.816050367
LPL	UCL	-0.122466581	0.21132005	-0.579531291	0.566734057	0.816050367
COL1A1	UCL	-0.068635153	0.118440975	-0.579488247	0.566854029	0.816050367
GPA33	UCL	0.384729082	0.66389839	0.579499947	0.56700083	0.816050367
IL12RB2	UCL	-0.11686231	0.201723125	-0.579320345	0.567019602	0.816050367
CHL1	NIH	-0.084953333	0.146807827	-0.578670329	0.567437736	0.775291546
FSHB	NIH	-0.087386667	0.151043747	-0.57855203	0.567516476	0.775291546

ACY3	NIH	-0.17054	0.294971612	-0.578157331	0.567779228	0.775291546
PRKG1	NIH	0.215766667	0.373453395	0.577760624	0.568043379	0.775291546
CRHR1	NIH	0.31126	0.538749519	0.577745295	0.568053587	0.775291546
NRGN	NIH	0.12798	0.221709651	0.577241448	0.56838917	0.775291546
SPRR3	NIH	0.119826667	0.207703967	0.576910825	0.568609434	0.775291546
OGN	NIH	0.129086667	0.2239926	0.576298801	0.569017281	0.775291546
IL20RA	NIH	0.201373333	0.349682336	0.575875052	0.569299751	0.775291546
DSG2	NIH	-0.07574	0.131536165	-0.575811223	0.569342305	0.775291546
DENND2B	NIH	0.218873333	0.381071679	0.574362634	0.570308497	0.775965767
GP1BA	NIH	0.086453333	0.150543842	0.574273463	0.570367999	0.775965767
FOS	UCL	0.08552961	0.14950342	0.57209133	0.570470981	0.820613523
KHDC3L	NIH	0.158066667	0.275486939	0.573771908	0.57070274	0.776060212
SH3BP1	UCL	-0.048716515	0.085292902	-0.571167284	0.571069176	0.820937604
TIMM10	NIH	-0.03616	0.063084101	-0.573203068	0.571082507	0.776198477
VSTM2L	UCL	0.09783957	0.17127929	0.571228258	0.571281884	0.820937604
VPS4B	NIH	0.163386667	0.285229764	0.572824744	0.571335152	0.776198477
GGT1	NIH	-0.102506667	0.179190695	-0.572053514	0.571850354	0.776330087
NDUFB7	NIH	-0.050573333	0.088432673	-0.57188516	0.57196285	0.776330087
DPP6	UCL	0.08427733	0.147648254	0.570798009	0.571965285	0.820937604
CNTN4	UCL	0.070730046	0.12364934	0.572021217	0.572164381	0.820937604
BCAT2	UCL	-0.233843051	0.409135309	-0.571554316	0.572185813	0.820937604
GSTT2B	NIH	0.461326667	0.807526423	0.57128368	0.572364856	0.7765154
NECAP2	UCL	-0.198504746	0.347826887	-0.570699831	0.572381402	0.820937604
MRPL28	NIH	0.097886667	0.17179116	0.56980037	0.573356846	0.777500591
SHH	NIH	0.15728	0.276311568	0.569212506	0.573750229	0.777673503
NEO1	UCL	-0.051639282	0.091041211	-0.567207769	0.573894813	0.822663135
TNFRSF19	UCL	-0.067905143	0.119330584	-0.569050626	0.574147382	0.822663135
VTCN1	NIH	-0.119846667	0.211411162	-0.56688902	0.57530636	0.77942154
HBQ1	UCL	-0.113252979	0.200783661	-0.564054754	0.576110009	0.824355851
BANK1	NIH	0.177373333	0.313560316	0.565675324	0.576120056	0.780162576
IFNW1	UCL	0.053462785	0.094906327	0.563321607	0.576622132	0.824355851
REG1A	NIH	0.113153333	0.200302529	0.564912156	0.576631999	0.780494492
CARHSP1	UCL	-0.104079968	0.184899565	-0.562900016	0.576651656	0.824355851

ALPI	UCL	0.374467786	0.663736165	0.564181681	0.577328277	0.824355851
IGFBP6	UCL	-0.063466479	0.11250364	-0.564128224	0.577350689	0.824355851
TMCO5A	UCL	-0.103145571	0.184166054	-0.560068313	0.577666512	0.824355851
FGFBP1	UCL	-0.08635124	0.153240561	-0.563501201	0.577785426	0.824355851
RASA1	UCL	0.073007727	0.130401942	0.559866873	0.577802981	0.824355851
RETN	UCL	-0.079055353	0.14033951	-0.563315013	0.577866965	0.824355851
WFDC1	NIH	-0.081106667	0.144103981	-0.562834324	0.57802698	0.782020775
IDO1	UCL	-0.174393645	0.30997587	-0.562603938	0.578355877	0.824650844
CR2	UCL	0.164865463	0.293249233	0.562202537	0.578689374	0.824724056
SLC34A3	UCL	0.150685689	0.268648183	0.560903435	0.579141437	0.824966092
CCER2	NIH	-0.126433333	0.225445106	-0.560816491	0.57938328	0.783290557
TIE1	NIH	-0.078393333	0.13982797	-0.56064129	0.579501116	0.783290557
TPSAB1	NIH	0.105966667	0.189155866	0.560208197	0.579792457	0.783322372
PDZK1	UCL	0.154376505	0.275509761	0.560330439	0.579799043	0.8252848
APOH	UCL	0.040184601	0.071955635	0.558463573	0.57992986	0.8252848
C2CD2L	NIH	0.141286667	0.252483851	0.559586944	0.580210499	0.783421746
B3GNT7	NIH	0.080333333	0.1438914	0.558291417	0.58108274	0.783421746
NENF	NIH	-0.311046667	0.557254761	-0.558176778	0.581159954	0.783421746
NOP56	UCL	0.159177116	0.285249638	0.558027408	0.58123681	0.826612054
TMEM132A	NIH	0.225026667	0.403317841	0.557938787	0.581320267	0.783421746
ITGAX	NIH	0.10578	0.189675663	0.557688839	0.581488658	0.783421746
SMPD1	UCL	0.104964301	0.18820026	0.557726654	0.581618811	0.826612054
BID	NIH	0.19178	0.344033824	0.557445189	0.58165283	0.783421746
NINJ1	UCL	-0.281724312	0.505184637	-0.557666032	0.581722533	0.826612054
IFNGR2	NIH	0.14264	0.255941642	0.557314547	0.581740865	0.783421746
PLB1	UCL	-0.128591028	0.230898953	-0.556914731	0.582046226	0.826612054
TGOLN2	UCL	-0.060383398	0.108518109	-0.556436141	0.582306063	0.826612054
PCDH9	NIH	0.07718	0.138770493	0.556170106	0.582512352	0.783838802
PGM2	NIH	-0.18776	0.337661324	-0.55606013	0.582586515	0.783838802
SPRR3	UCL	-0.123238981	0.221684282	-0.55592115	0.582760696	0.826612054
KLK12	UCL	0.361043772	0.649044393	0.556269765	0.582842095	0.826612054
AHNAK2	UCL	0.126275019	0.227752758	0.554439035	0.583962484	0.827401044
ANXA1	UCL	-0.061315417	0.110848966	-0.553143795	0.583964541	0.827401044

CEP112	NIH	0.099266667	0.179290315	0.553664411	0.584203238	0.784713141
UPK3BL1	NIH	-0.154973333	0.279942155	-0.553590557	0.584253113	0.784713141
REXO2	NIH	0.119273333	0.215500979	0.553470029	0.584334512	0.784713141
ATRAID	NIH	0.069973333	0.126525017	0.55303951	0.584625308	0.784713141
RAB3GAP1	NIH	0.081186667	0.146867045	0.552790225	0.584793721	0.784713141
CGA	UCL	-0.133761812	0.241982078	-0.552775699	0.584827948	0.827830824
C4BPB	NIH	0.099013333	0.179140656	0.552712799	0.584846033	0.784713141
DPP7	UCL	0.12724127	0.230323656	0.552445511	0.585252821	0.827830824
CHI3L1	UCL	-0.186342089	0.337366746	-0.552342788	0.585335613	0.827830824
PDE4D	UCL	0.150076887	0.271997662	0.551758006	0.585614127	0.827830824
B4GALT1	UCL	-0.061225595	0.110991481	-0.551624271	0.585823232	0.827830824
CCL7	UCL	0.131040679	0.238658923	0.549070942	0.586742231	0.827830824
ACHE	UCL	-0.075103864	0.136515777	-0.550147873	0.586772952	0.827830824
CXCL14	UCL	-0.084357582	0.153709952	-0.548810153	0.586877312	0.827830824
CRYBB2	UCL	0.220819832	0.401858999	0.5494958	0.586878084	0.827830824
CKAP4	UCL	0.061624285	0.112130061	0.54957863	0.587296966	0.827830824
AMIGO1	UCL	-0.08636354	0.158277216	-0.545647328	0.587475422	0.827830824
CNTN2	UCL	0.141766282	0.258312807	0.548816311	0.587666425	0.827830824
CGN	NIH	-0.1436	0.261932089	-0.548233707	0.587876217	0.78841721
EDN1	UCL	-0.080191746	0.146307311	-0.548104846	0.588104455	0.827961379
EPB41L5	UCL	0.15208588	0.277962946	0.547144438	0.588325619	0.827961379
RRAS	NIH	0.1822	0.332839709	0.547410646	0.588433864	0.788803416
FUS	UCL	-0.070017956	0.127834585	-0.547723106	0.588723842	0.828123095
SPRED2	NIH	-0.109973333	0.201108126	-0.546836846	0.588822782	0.788906075
BCL2L15	NIH	0.191726667	0.350825257	0.546501891	0.58904987	0.788906075
MRPL46	UCL	-0.052860375	0.09690106	-0.545508736	0.58936896	0.828631779
TREH	UCL	0.186034323	0.340961768	0.545616371	0.589931478	0.829023899
KLRC1	NIH	0.1257	0.230820517	0.544578973	0.590354367	0.79027009
CLU	NIH	0.054993333	0.101052731	0.544204324	0.59060869	0.79027009
CREG1	UCL	0.073930758	0.136092125	0.543240531	0.590832912	0.829807284
CBLN1	NIH	0.156953333	0.28864616	0.543756873	0.590912502	0.790315075
ITGAX	UCL	0.123467653	0.228269361	0.540885788	0.591346005	0.829807284
CXCL12	UCL	0.097582889	0.179795016	0.542745238	0.591358317	0.829807284

APOA4	UCL	-0.074673172	0.137440068	-0.543314429	0.591724644	0.829807284
TNFRSF13B	UCL	0.084445617	0.155601108	0.542705759	0.591908377	0.829807284
SPRR1B	UCL	-0.096225184	0.177923038	-0.540824761	0.592401945	0.829846728
GBA	UCL	-0.109374966	0.202648148	-0.539728429	0.592504318	0.829846728
ARID3A	NIH	0.106573333	0.19687937	0.541312853	0.592573292	0.792174077
PDXDC1	NIH	-0.11812	0.21902006	-0.53931133	0.59393507	0.793631832
GGA1	NIH	0.121713333	0.225914839	0.538757585	0.594312088	0.793772995
TOP1MT	UCL	-0.109568164	0.204100094	-0.536835443	0.594351626	0.830858138
PRG2	UCL	-0.104091929	0.193176573	-0.538843437	0.594373182	0.830858138
CALCB	UCL	0.114211279	0.211962479	0.538827813	0.59452676	0.830858138
AGR3	UCL	0.259033025	0.482451801	0.536909646	0.595112382	0.830858138
RBPMS	NIH	0.136593333	0.254100977	0.537555326	0.595131046	0.794504021
S100G	NIH	-0.164006667	0.305453825	-0.536927854	0.595558684	0.794712204
COL4A4	UCL	0.122458963	0.22963803	0.533269523	0.59595722	0.830858138
DCN	UCL	-0.040558414	0.075641482	-0.53619275	0.596001988	0.830858138
PDRG1	UCL	0.064966901	0.121852259	0.533161236	0.596031676	0.830858138
MORC3	NIH	0.09826	0.183433878	0.535669861	0.596416484	0.795110855
PCDHB15	UCL	0.089907756	0.167791348	0.535830703	0.596433639	0.830858138
PROK1	NIH	-0.176513333	0.329546071	-0.535625664	0.596446632	0.795110855
CXCL9	UCL	0.205594367	0.383880072	0.535569262	0.596707142	0.830858138
IFNL2	UCL	-0.074564055	0.139825546	-0.533264896	0.596867007	0.830858138
SIRT1	UCL	0.217689207	0.406849535	0.535060724	0.596893977	0.830858138
TSLP	NIH	-0.181246667	0.338812835	-0.534946283	0.596910145	0.795110855
GHRL	UCL	-0.210969812	0.39435239	-0.534977896	0.59691925	0.830858138
ISM2	UCL	-0.041065254	0.077209586	-0.531867301	0.596921687	0.830858138
LTA	NIH	0.111153333	0.20780381	0.53489555	0.596944765	0.795110855
HS6ST2	UCL	0.109040863	0.204616712	0.532903016	0.598615707	0.832819472
PMCH	UCL	-0.133599893	0.250933564	-0.532411412	0.599099195	0.833095598
SCGB3A1	NIH	-0.072406667	0.13619656	-0.531633593	0.599172729	0.797711108
ESPL1	NIH	-0.228606667	0.430327208	-0.531239165	0.599442398	0.797711108
SPAG1	UCL	0.146599648	0.276286939	0.530606507	0.599686804	0.833516181
ASPN	NIH	-0.065626667	0.123662614	-0.530691244	0.599817107	0.797846765

DEFB104A_DEFB104B	NIH	-0.261886667	0.494176233	-0.529945897	0.600327008	0.798162045
MAGED1	UCL	-0.064710828	0.122607436	-0.527788771	0.600732199	0.834572347
MAGED1	NIH	0.10938	0.206909454	0.528637035	0.601222917	0.798990019
FCGR2B	UCL	0.155646966	0.29487142	0.527846903	0.601576736	0.835253672
RRP15	NIH	0.161126667	0.305254541	0.527843636	0.601766304	0.799083048
OGT	NIH	0.146833333	0.278231982	0.527737079	0.6018393	0.799083048
CNST	UCL	-0.18910952	0.360144907	-0.525092862	0.601941097	0.835253672
NPTX2	UCL	-0.059233515	0.112292534	-0.5274929	0.602079879	0.835253672
SMOC2	NIH	-0.074473333	0.141239777	-0.527283	0.602150416	0.799122107
SNAPIN	NIH	0.102293333	0.194238894	0.526636717	0.602593352	0.799122107
XRCC4	NIH	-0.05902	0.112099157	-0.526498163	0.602688331	0.799122107
NPHS1	UCL	-0.07466011	0.142210659	-0.52499658	0.603172352	0.83554614
ARHGAP5	UCL	0.298669799	0.567946791	0.525876375	0.603231428	0.83554614
GPR15L	UCL	-0.137118692	0.261111573	-0.525134489	0.603374733	0.83554614
MOG	UCL	-0.074721779	0.142510605	-0.524324342	0.603434109	0.83554614
BHMT2	NIH	-0.08296	0.158195047	-0.524415914	0.604116578	0.800652918
CDHR2	NIH	0.197313333	0.377204709	0.523093505	0.60502447	0.801386787
TNFSF9	UCL	-0.089884949	0.172051937	-0.52242916	0.605133957	0.837503104
ANGPTL1	NIH	-0.07576	0.144908864	-0.522811357	0.605218261	0.801386787
EPHA10	NIH	0.207013333	0.39629437	0.522372632	0.605519653	0.80142307
L1CAM	UCL	0.071895894	0.137627304	0.522395569	0.605681802	0.837862264
CPQ	UCL	0.074323982	0.142420022	0.521864699	0.605966755	0.837862264
RELB	NIH	-0.171433333	0.32880841	-0.521377581	0.606203488	0.801703619
KYAT1	NIH	0.093533333	0.179434741	0.521266578	0.606279796	0.801703619
PDCD1LG2	NIH	-0.059506667	0.114251158	-0.52084082	0.606572521	0.801728253
NFIC	UCL	-0.049379799	0.095376474	-0.517735629	0.606682086	0.838221554
TOMM20	UCL	-0.139843586	0.269424594	-0.519045362	0.606800139	0.838221554
PARP1	NIH	0.176106667	0.338885334	0.519664468	0.607381655	0.802435113
PLXNB2	NIH	0.04548	0.087924544	0.517261707	0.609035928	0.804117388
RLN2	UCL	0.112803907	0.21795494	0.517556091	0.609199548	0.840868856
ERBB4	UCL	0.048837326	0.094542846	0.51656289	0.609291904	0.840868856
AP1G2	NIH	0.18332	0.354841911	0.51662443	0.609475041	0.804117388

XPNPEP2	NIH	-0.236713333	0.458198347	-0.516617607	0.609479743	0.804117388
SELENOP	NIH	0.0767	0.148636236	0.516024909	0.609888273	0.804293597
NUDT2	UCL	-0.098753218	0.192270583	-0.513615844	0.61025877	0.841661936
MVK	UCL	-0.195179587	0.378477947	-0.515696063	0.610442458	0.841661936
GAD1	NIH	-0.076853333	0.149341562	-0.5146145	0.610860944	0.805104968
MAP2K1	UCL	-0.104594805	0.203978016	-0.512774892	0.61093739	0.841947191
DBNL	NIH	0.184213333	0.358158515	0.514334647	0.611054027	0.805104968
FCRL3	UCL	-0.081709653	0.159159746	-0.5133814	0.611268351	0.84200631
CDA	NIH	0.096926667	0.188634513	0.513833153	0.611400102	0.805198244
CD99L2	UCL	0.064436385	0.125537942	0.513282151	0.612028426	0.842656189
CASQ2	NIH	0.085013333	0.165860045	0.512560656	0.612278647	0.805676814
PENK	NIH	0.057413333	0.112061271	0.512338766	0.612431902	0.805676814
LRP2	NIH	-0.116746667	0.227971789	-0.512110148	0.612589824	0.805676814
PTPRR	UCL	-0.075312855	0.147519174	-0.510529266	0.612893006	0.84326496
TLR3	UCL	-0.167398739	0.327106115	-0.511756678	0.613047567	0.84326496
ICAM1	NIH	-0.063273333	0.123717975	-0.511432014	0.613058365	0.805714568
GDF2	NIH	0.063186667	0.123623621	0.511121308	0.613273097	0.805714568
RYR1	NIH	0.088613333	0.173454806	0.510872748	0.613444904	0.805714568
ITGA2	UCL	0.064931225	0.127007007	0.511241279	0.613486866	0.843373716
WFDC12	UCL	-0.154543413	0.302763477	-0.510442723	0.613911089	0.843373716
AMY2B	UCL	0.103996325	0.203710282	0.510510931	0.613992223	0.843373716
FN1	UCL	0.061299854	0.120145989	0.510211409	0.61434748	0.843465328
ACP5	NIH	0.075433333	0.148057289	0.509487468	0.614402831	0.806092606
SMPD3	NIH	-0.0935	0.183544768	-0.509412506	0.614454688	0.806092606
EPS8L2	NIH	0.06146	0.120684675	0.509261015	0.614559491	0.806092606
EGLN1	UCL	-0.100934312	0.198506752	-0.508467902	0.614707022	0.843562735
TPPP2	NIH	0.220013333	0.432727133	0.508434338	0.61513154	0.806481289
USP8	NIH	0.1101	0.217121841	0.507088553	0.616063334	0.807341062
ADAMTSL2	UCL	0.087493689	0.17249729	0.507217761	0.616325243	0.845386525
NRN1	UCL	-0.049111113	0.097197142	-0.505273223	0.616863195	0.845521606
FGF12	UCL	0.105643841	0.208962447	0.505563761	0.617002253	0.845521606
CASP1	NIH	0.253393333	0.5010747	0.505699716	0.617025619	0.808240007
PER3	NIH	0.201153333	0.398757469	0.504450321	0.617891883	0.809012425

NCAN	NIH	-0.0877	0.174324906	-0.503083593	0.61884014	0.809862843
TDGF1	UCL	0.413618752	0.821854256	0.503275062	0.618910148	0.847581373
ACY3	UCL	0.147476191	0.293149338	0.5030753	0.619085266	0.847581373
SRPX	NIH	0.133353333	0.265265633	0.502716209	0.619095151	0.809862843
ENPP6	UCL	0.069634465	0.138687139	0.502097493	0.61963204	0.847932797
CCL28	NIH	-0.103326667	0.205973028	-0.501651442	0.619834508	0.810402595
CCDC28A	NIH	-0.083	0.165592173	-0.50123142	0.620126276	0.810402595
PHYKPL	UCL	0.069823483	0.139253134	0.501414084	0.620127998	0.848214384
MYCBP2	NIH	0.137313333	0.274119367	0.500925326	0.620338944	0.810402595
CD22	UCL	0.118736842	0.237009977	0.500978244	0.620546123	0.848389298
LTO1	NIH	-0.147873333	0.295616084	-0.500220865	0.620828515	0.810558756
BIRC2	NIH	0.119693333	0.239407788	0.499955889	0.621012708	0.810558756
ITIH1	UCL	0.023059134	0.046352972	0.497468305	0.621277549	0.84853605
RANGAP1	NIH	0.04902	0.098227771	0.499044206	0.62164664	0.81081503
FOLR1	NIH	0.071466667	0.143360132	0.498511446	0.622017228	0.81081503
SESTD1	NIH	0.09876	0.198141078	0.498432739	0.622071985	0.81081503
XCL1	UCL	-0.09553579	0.191563255	-0.498716677	0.62207424	0.84853605
ITGA5	UCL	-0.059795607	0.119837978	-0.498970425	0.622104677	0.84853605
VSIG10L	UCL	-0.067199507	0.135446562	-0.496132984	0.622136863	0.84853605
KIF1C	UCL	0.102609816	0.206823764	0.496121982	0.622186621	0.84853605
CEP164	NIH	0.188393333	0.378239591	0.49807936	0.622317861	0.81081503
CHM	NIH	-0.128986667	0.259324912	-0.497394043	0.622794821	0.811075178
TNFRSF1A	UCL	-0.070828709	0.142472204	-0.497140546	0.623261427	0.84853605
PON3	UCL	0.062093798	0.125067989	0.496480345	0.623467466	0.84853605
CDH5	UCL	0.062400527	0.12586552	0.495771417	0.62384231	0.84853605
CLEC4C	UCL	0.115307165	0.232423796	0.496107399	0.623855098	0.84853605
PRR5	UCL	-0.108555927	0.219865115	-0.493738747	0.624011886	0.84853605
CLMP	UCL	0.052535541	0.105956124	0.495823545	0.624144876	0.84853605
MMP3	UCL	0.163564791	0.329818759	0.495923251	0.62421474	0.84853605
RBP2	UCL	-0.154653814	0.313202132	-0.493782762	0.6246936	0.84853605
B3GAT3	UCL	0.043693141	0.088534705	0.49351428	0.624717612	0.84853605
MCEMP1	NIH	0.164366667	0.332739629	0.493979834	0.625173492	0.813455012
TNF	UCL	-0.126698889	0.257427383	-0.492173318	0.625297883	0.848929731

TRIM5	NIH	-0.14358	0.29080902	-0.493726089	0.625350441	0.813455012
SORCS2	NIH	0.08686	0.175981722	0.493573986	0.62545652	0.813455012
TMPRSS11D	UCL	-0.06977894	0.141903661	-0.491734602	0.626709607	0.850270062
PRRT3	NIH	0.095566667	0.194346929	0.491732321	0.626741581	0.814764055
VWA5A	UCL	-0.089198885	0.181940202	-0.490264846	0.626866912	0.850270062
TNFRSF10C	NIH	0.1163	0.236841257	0.491046203	0.62722064	0.814819777
GSAP	UCL	0.212705013	0.433762925	0.490371584	0.627441196	0.850654274
TWF2	NIH	0.098853333	0.201459468	0.490685964	0.627472231	0.814819777
CTSB	NIH	0.116866667	0.238272817	0.490474189	0.627620156	0.814819777
GNGT1	UCL	0.099403363	0.203247867	0.489074571	0.628250016	0.851355956
RHOC	NIH	0.110526667	0.226076334	0.488890918	0.628726574	0.815893765
ARMCX2	NIH	0.12588	0.257861599	0.488168849	0.62923146	0.815893765
NUBP1	UCL	-0.135376852	0.279042173	-0.485148358	0.629463675	0.851695661
SERPINA3	NIH	0.02584	0.052992501	0.487616162	0.629618035	0.815893765
RASGRF1	UCL	-0.139079536	0.285041362	-0.487927559	0.629632113	0.851695661
FZD10	UCL	0.16999768	0.34967476	0.486159424	0.629795514	0.851695661
ERCC1	UCL	0.061978037	0.127766629	0.485087829	0.629822985	0.851695661
INSL4	NIH	0.12734	0.261308178	0.487317316	0.629827106	0.815893765
TET2	NIH	-0.223533333	0.45872197	-0.487295896	0.629842093	0.815893765
PXN	UCL	0.146948297	0.301899336	0.48674601	0.630259733	0.851695661
SMPDL3A	UCL	0.103121853	0.211852426	0.486762672	0.630317337	0.851695661
RNASE10	UCL	-0.079454106	0.163321997	-0.486487473	0.630555182	0.851695661
IL20	NIH	0.086486667	0.177895789	0.486164777	0.630633709	0.816315737
NRXN3	NIH	0.048413333	0.099609161	0.486032938	0.630726006	0.816315737
PCDH1	UCL	0.024167636	0.049768717	0.485598938	0.631014634	0.851695661
SNX5	UCL	0.052171745	0.107649479	0.484644659	0.631123093	0.851695661
SLC1A4	NIH	0.08776	0.180817991	0.485349935	0.631204255	0.816573395
POMC	UCL	-0.132619391	0.273982413	-0.484043443	0.631671887	0.852042882
GHRL	NIH	-0.16512	0.340816013	-0.484484277	0.631810636	0.816711695
FCGR3B	NIH	-0.11334	0.233980119	-0.484400129	0.631869595	0.816711695
HADH	UCL	0.205234029	0.425629879	0.482188961	0.632054357	0.852165538
CD59	NIH	-0.040453333	0.083684279	-0.483404215	0.632567571	0.817252715
PAM	NIH	0.06782	0.140479223	0.482776018	0.633008014	0.817460681

ACE2	UCL	-0.129921143	0.268870557	-0.483210748	0.633193845	0.853308257
NECTIN1	NIH	0.043026667	0.08925375	0.482071247	0.633502309	0.817737976
SORD	UCL	-0.117143132	0.243139066	-0.481794778	0.633721535	0.853625828
PRDX1	UCL	-0.091035943	0.190206449	-0.47861649	0.634502781	0.85428449
MAGEA3	NIH	0.373526667	0.778021716	0.480097996	0.634887174	0.819164086
CLASP1	NIH	-0.12688	0.264712343	-0.479312745	0.635438653	0.819514136
PARD3	UCL	-0.126104068	0.263455143	-0.478654797	0.635501586	0.85523533
SPRED2	UCL	-0.168577152	0.352873797	-0.477726466	0.636496714	0.856180347
UBQLN3	UCL	0.227757673	0.477553809	0.476925676	0.637351386	0.856696964
RTBDN	UCL	-0.056911636	0.119377611	-0.476736264	0.637466951	0.856696964
CALCA	UCL	0.171611066	0.360424328	0.476136188	0.637922973	0.856717704
SLAMF7	UCL	0.095781072	0.20138242	0.475617843	0.638105509	0.856717704
CASQ2	UCL	0.163683405	0.344505173	0.475126117	0.638361669	0.856717704
SLC27A4	NIH	0.17798	0.375220561	0.474334348	0.638939918	0.823099361
MSR1	NIH	0.093226667	0.196583229	0.474235098	0.639009806	0.823099361
MESD	NIH	0.098986667	0.208830986	0.474003731	0.63917274	0.823099361
SERPINA5	UCL	-0.040712496	0.085873732	-0.474097202	0.639238685	0.857500998
SPON1	NIH	-0.055753333	0.117708988	-0.473654002	0.639419061	0.823099361
ADGRE5	NIH	0.05688	0.120312641	0.472768279	0.640043083	0.823099361
LYVE1	NIH	0.03704	0.078355602	0.472716681	0.640079443	0.823099361
PRKAR1A	NIH	-0.137333333	0.290614371	-0.472562085	0.640188392	0.823099361
LEP	UCL	-0.168581987	0.357094853	-0.472093018	0.64073527	0.858410882
ITGB2	UCL	-0.077547557	0.164256176	-0.472113495	0.640799445	0.858410882
CD248	NIH	-0.08752	0.185596022	-0.471561831	0.640893497	0.823643884
ITGBL1	UCL	-0.087800468	0.186118645	-0.471744613	0.641044056	0.858410882
KLK11	UCL	0.05223635	0.110819112	0.471365901	0.64120248	0.858410882
CFB	UCL	-0.072108211	0.153195284	-0.470694716	0.641450732	0.858410882
AMBP	UCL	0.036592815	0.077799133	0.470349899	0.641867541	0.858410882
FBLN2	UCL	0.069464546	0.147631274	0.470527308	0.641972695	0.858410882
IL4R	NIH	0.074	0.157449136	0.469993051	0.64200006	0.824703634
GALNT5	UCL	0.064232294	0.137008214	0.468820756	0.642620769	0.85856999
GDF15	UCL	-0.102941572	0.219370385	-0.469259202	0.642679144	0.85856999
IFIT3	NIH	-0.114113333	0.243595977	-0.468453276	0.643086979	0.825593762

CTSL	UCL	0.063219029	0.134873055	0.468729863	0.643092612	0.858729879
VAMP5	NIH	0.099673333	0.213040776	0.467860357	0.643505733	0.825593762
PLAUR	NIH	0.062446667	0.133486616	0.467812195	0.643539753	0.825593762
PTPRH	UCL	0.084754152	0.181204354	0.467726904	0.643630212	0.858810313
MN1	UCL	-0.100507946	0.215621485	-0.466131407	0.643740471	0.858810313
ITGBL1	NIH	0.089266667	0.191512852	0.466113192	0.644740375	0.826771415
TGFBR2	UCL	-0.054316273	0.116744972	-0.465255779	0.645540352	0.860142838
OFD1	UCL	-0.057606236	0.124268279	-0.463563479	0.645619442	0.860142838
CDH2	UCL	-0.074212615	0.159556584	-0.465117845	0.645622096	0.860142838
PRTFDC1	NIH	0.176953333	0.380796944	0.464692105	0.645745355	0.827039813
ICOSLG	NIH	-0.030293333	0.065216463	-0.464504386	0.64587816	0.827039813
FCRLB	NIH	-0.09154	0.197164772	-0.464281723	0.646035701	0.827039813
CBLN4	NIH	-0.04316	0.093015239	-0.464009987	0.646227985	0.827039813
CD82	NIH	0.166886667	0.360140526	0.463393188	0.646664535	0.827039813
HSD17B14	NIH	0.1856	0.400671297	0.463222601	0.646785294	0.827039813
DPEP1	NIH	0.1039	0.224396487	0.463019727	0.64692892	0.827039813
PRKCQ	NIH	0.105893333	0.229199087	0.462014639	0.647640688	0.827333218
PBK	UCL	-0.053242699	0.115738068	-0.46002754	0.647753056	0.861215692
SLC16A1	NIH	0.15812	0.342390232	0.46181224	0.647784061	0.827333218
CTSS	UCL	-0.039805459	0.086259667	-0.461460848	0.648168038	0.861215692
SAT2	UCL	0.110343214	0.239364901	0.460983267	0.648214102	0.861215692
F11R	NIH	0.06396	0.138708323	0.461111479	0.648280565	0.827333218
PFDN6	NIH	0.099426667	0.215630034	0.461098413	0.648289824	0.827333218
FUT3_FUT5	UCL	-0.120278208	0.260675622	-0.461409497	0.6484081	0.861215692
OLR1	UCL	0.065956752	0.143339029	0.460145097	0.648577549	0.861215692
SART1	UCL	0.050124838	0.1091438	0.459255022	0.648615843	0.861215692
MAMDC2	UCL	-0.082488045	0.179066955	-0.460654758	0.648754541	0.861215692
GP5	UCL	0.091908771	0.200173141	0.45914637	0.648784452	0.861215692
ISLR2	UCL	-0.104658292	0.227939982	-0.459148462	0.649625263	0.861940374
PPP3R1	UCL	-0.071717965	0.15647055	-0.458348008	0.650342232	0.862500156
GP6	NIH	0.128493333	0.28043524	0.458192534	0.650350468	0.829369634
CST6	NIH	0.099446667	0.217109435	0.458048571	0.650452629	0.829369634
PSCA	NIH	-0.54216	1.185071596	-0.457491346	0.650848124	0.829512315

ADIPOQ	NIH	0.18116	0.396833185	0.45651424	0.651541882	0.830034845
FASLG	UCL	0.065301583	0.143053191	0.456484633	0.651719278	0.863934445
DARS1	NIH	0.116073333	0.254792289	0.455560621	0.65221927	0.83043044
REST	UCL	0.098026331	0.215638778	0.454585823	0.652390216	0.864431824
GABRA4	NIH	0.1899	0.417532803	0.454814564	0.652749432	0.83043044
F13B	UCL	-0.046868588	0.10310231	-0.454583293	0.652952913	0.864785395
CD300LG	NIH	0.102513333	0.225558332	0.454487016	0.652982252	0.83043044
RFC4	NIH	-0.039673333	0.08729412	-0.454478876	0.652988038	0.83043044
PFDN2	UCL	0.08226784	0.18256983	0.45061027	0.65401033	0.865793567
CYTL1	UCL	0.047493375	0.105036504	0.45216066	0.654512162	0.865822594
IL2	UCL	0.050734688	0.112805672	0.449752984	0.654624678	0.865822594
LTBP2	NIH	-0.10078	0.223016273	-0.451895275	0.654825706	0.832210516
GPA33	NIH	0.268746667	0.595250408	0.451485061	0.655117687	0.832210516
TUBB3	NIH	0.137086667	0.303771682	0.451281917	0.655262301	0.832210516
LELP1	UCL	0.139206558	0.309088937	0.45037703	0.655690542	0.866686563
LONP1	NIH	-0.084186667	0.186946566	-0.450324757	0.655943866	0.832210516
CTSL	NIH	0.057566667	0.127843239	0.450291052	0.655967872	0.832210516
FGF20	NIH	0.102053333	0.226728366	0.450112772	0.656094855	0.832210516
M6PR	UCL	0.087288645	0.193944314	0.450070658	0.656142639	0.866686563
B2M	UCL	-0.049280054	0.109618351	-0.449560259	0.656727876	0.866686563
CD14	UCL	0.089464181	0.199320654	0.44884551	0.657306476	0.866686563
ENG	UCL	-0.029572073	0.065917702	-0.44862111	0.657319353	0.866686563
DTX2	UCL	0.177332752	0.395296299	0.448607166	0.657385598	0.866686563
DRAXIN	UCL	0.077037529	0.171864849	0.448244824	0.657477502	0.866686563
CD33	UCL	0.201660122	0.450247722	0.447887044	0.657901348	0.866686563
CENPF	NIH	-0.101846667	0.227586739	-0.447507034	0.657952035	0.833747306
LIFR	NIH	-0.039226667	0.087657742	-0.447498026	0.657958458	0.833747306
GRPEL1	UCL	0.142712973	0.31869479	0.447804538	0.658083816	0.866686563
DNLZ	NIH	0.316846667	0.708491202	0.447213269	0.658161549	0.833747306
ATRAID	UCL	-0.063032003	0.141367972	-0.445871876	0.65875071	0.866686563
SUSD1	UCL	-0.104788545	0.235546007	-0.444875067	0.658818795	0.866686563
EFNA1	UCL	-0.055147286	0.123668413	-0.445928629	0.659081002	0.866686563
F2	UCL	0.019655487	0.044223019	0.444462809	0.659132477	0.866686563

ANXA1	NIH	0.10388	0.233040008	0.44576037	0.659198178	0.834698991
CRIM1	NIH	-0.035993333	0.080881703	-0.445012062	0.659732357	0.835013909
LTBP2	UCL	0.06024677	0.135344446	0.445136626	0.659897075	0.867301776
PDZD2	NIH	-0.136686667	0.307797205	-0.444080272	0.660397771	0.835494584
PALM	UCL	-0.054817339	0.123451152	-0.444040728	0.660436814	0.867621037
EDEM2	UCL	-0.114918212	0.259166499	-0.44341461	0.660969969	0.867931366
SEZ6	UCL	0.047648837	0.108085675	0.440843219	0.661485986	0.868095531
EDNRB	UCL	0.158607085	0.358574906	0.442326226	0.661688964	0.868095531
FLT3LG	NIH	-0.06994	0.158452993	-0.441392735	0.662318591	0.837562421
TREML1	NIH	0.087413333	0.198232935	0.440962716	0.66262615	0.837589234
STC1	UCL	0.202377987	0.458985358	0.440924713	0.662811059	0.869177534
RUVBL1	UCL	0.239620351	0.544964566	0.439698956	0.663541248	0.869260294
LMNB2	UCL	0.052330103	0.119017443	0.439684316	0.663571896	0.869260294
TSHB	UCL	0.096643737	0.220054351	0.439181215	0.663766328	0.869260294
PTPRZ1	UCL	-0.058439187	0.13324925	-0.438570475	0.664107429	0.869282274
REG4	UCL	-0.112590372	0.256641927	-0.438706072	0.6643779	0.869282274
CD55	NIH	-0.036486667	0.083207676	-0.43850121	0.664387823	0.839453297
RNF31	UCL	0.104425226	0.238981327	0.436959774	0.665044616	0.869418994
SCN2A	UCL	0.189700872	0.433859726	0.437240104	0.665101738	0.869418994
PPT1	NIH	-0.072886667	0.166655732	-0.437348693	0.665213341	0.840122109
TSPYL1	UCL	-0.054199039	0.12456506	-0.435106273	0.665374714	0.869418994
ITGB5	NIH	-0.044013333	0.10072615	-0.436960347	0.665491599	0.840122109
VCAN	NIH	0.060173333	0.137847266	0.436521777	0.665805902	0.840156283
KLRB1	UCL	0.076030316	0.174026763	0.436888644	0.665807639	0.869578348
ALMS1	NIH	0.095013333	0.218015554	0.435809884	0.666316216	0.840437659
FSTL1	UCL	-0.037707231	0.086573049	-0.435553915	0.666712446	0.869578348
ANKRA2	NIH	-0.263386667	0.605884137	-0.434714577	0.667101694	0.840950325
FMR1	NIH	0.055613333	0.128011135	0.434441374	0.667297676	0.840950325
ROBO4	UCL	0.026939728	0.062062919	0.434071237	0.667854458	0.869578348
DKK3	NIH	-0.054706667	0.126189486	-0.433527929	0.66795311	0.841142223
LGALS1	UCL	0.060498974	0.139559991	0.433497978	0.667974858	0.869578348
SLC9A3R2	UCL	0.120564567	0.27812658	0.433488116	0.668063753	0.869578348
CBS	UCL	0.130431489	0.300169194	0.434526564	0.66813556	0.869578348

YES1	NIH	0.136566667	0.315250627	0.433200301	0.668188262	0.841142223
ZCCHC8	UCL	-0.177517433	0.409836305	-0.433142284	0.668237472	0.869578348
EP300	UCL	0.0862493	0.199473625	0.432384482	0.668303757	0.869578348
KLK3	UCL	0.372366684	0.859768763	0.433100968	0.668382346	0.869578348
C1GALT1C1	UCL	-0.05645576	0.131181556	-0.430363552	0.668809808	0.869578348
BLNK	NIH	-0.148493333	0.343470241	-0.432332457	0.668811313	0.841142223
IFNG	NIH	-0.138613333	0.32083183	-0.432043583	0.669018758	0.841142223
AFAP1	NIH	0.115533333	0.267500839	0.431898958	0.669122625	0.841142223
CSPG4	NIH	0.063713333	0.147544159	0.431825521	0.669175369	0.841142223
RBP7	UCL	0.066312218	0.154053047	0.430450545	0.669467304	0.869578348
A1BG	UCL	0.028661827	0.066535288	0.430776329	0.669884036	0.869578348
KLK14	UCL	0.071133434	0.165122203	0.430792666	0.669995787	0.869578348
DECR1	NIH	0.078466667	0.182214658	0.430627632	0.670035959	0.84186219
AFM	UCL	0.029534377	0.068683117	0.43000926	0.670113289	0.869578348
SLC39A5	UCL	-0.086937644	0.202131311	-0.430104786	0.67026184	0.869578348
PRUNE2	UCL	-0.052184328	0.121439249	-0.429715502	0.670263148	0.869578348
CLEC12A	UCL	-0.043117753	0.10044503	-0.429267162	0.670554087	0.869578348
PRKAB1	NIH	0.13338	0.310488658	0.429580909	0.670788321	0.842245316
CDH6	NIH	-0.064873333	0.151078238	-0.429402237	0.670916781	0.842245316
FGF20	UCL	-0.120431406	0.280241011	-0.429742263	0.670952144	0.869708699
ESPL1	UCL	0.113309	0.264676423	0.428103864	0.671731304	0.869872712
DHODH	NIH	-0.14232	0.332544314	-0.427973037	0.671944703	0.843173856
MILR1	UCL	0.099127004	0.231562375	0.428079059	0.671981526	0.869872712
SERPINF2	UCL	0.01452702	0.034060652	0.426504449	0.67207885	0.869872712
GLT8D2	UCL	-0.099271514	0.232359328	-0.427232747	0.672269058	0.869872712
MFAP4	NIH	-0.104033333	0.244120789	-0.426155157	0.673253111	0.843714284
PTPN6	NIH	0.119706667	0.281057172	0.425915716	0.673425525	0.843714284
ARAF	NIH	-0.085433333	0.20074869	-0.425573554	0.673671936	0.843714284
CLPS	NIH	-0.12992	0.305554874	-0.425193676	0.673945552	0.843714284
SELE	NIH	-0.08424	0.198127647	-0.425180439	0.673955088	0.843714284
BMP10	NIH	0.049873333	0.117357073	0.424970836	0.674106079	0.843714284
FGFR4	UCL	0.092796106	0.218405136	0.424880602	0.674346077	0.871290915
OLR1	NIH	0.168526667	0.397488091	0.42397916	0.674820642	0.843723594

TSPAN8	NIH	-0.16996	0.400980531	-0.423860978	0.67490582	0.843723594
IL17F	UCL	0.093064315	0.219929737	0.423154759	0.674937237	0.871290915
TNFSF12	UCL	0.047385276	0.11182531	0.423743748	0.675091462	0.871290915
MCEMP1	UCL	-0.181086532	0.428940324	-0.422171856	0.675101381	0.871290915
BCAT2	NIH	-0.123886667	0.292741644	-0.423194545	0.675386223	0.843723594
C8B	UCL	0.047230365	0.111780676	0.422527103	0.675400525	0.871290915
EVI2B	NIH	0.0858	0.20280699	0.423062342	0.67548154	0.843723594
LAMP3	NIH	-0.09672	0.228674443	-0.422959376	0.67555578	0.843723594
MTUS1	UCL	-0.099051363	0.234286894	-0.422778079	0.676021812	0.871290915
SERPINC1	UCL	0.02486868	0.058996779	0.421526063	0.676081748	0.871290915
PSPN	UCL	0.170824851	0.404375005	0.422441668	0.676162869	0.871290915
TIMP2	UCL	-0.030802795	0.073285467	-0.420312465	0.676246548	0.871290915
HSD17B3	NIH	-0.113853333	0.269848869	-0.421915176	0.676308858	0.843788258
VSTM2B	UCL	0.039239005	0.093267926	0.420712741	0.676345907	0.871290915
IGFBP2	NIH	0.120286667	0.285205954	0.421753701	0.676425344	0.843788258
ACAN	NIH	-0.049966667	0.118492121	-0.421687672	0.676472979	0.843788258
KIR2DS4	UCL	-0.312410785	0.741765888	-0.421171679	0.676960136	0.871698008
SLA2	NIH	-0.1542	0.36645929	-0.420783439	0.677125453	0.844242093
IGFBP7	UCL	-0.050348141	0.119707844	-0.420591826	0.677526111	0.871985117
ARNTL	NIH	0.052893333	0.125878925	0.420192129	0.677552267	0.844414308
PLXDC1	UCL	0.120819071	0.287695505	0.419954669	0.677779742	0.871985117
HSDL2	NIH	0.086593333	0.20687194	0.418584238	0.678713411	0.845501162
FNTA	UCL	-0.053591299	0.128208164	-0.418002239	0.678965596	0.872924809
CD3G	NIH	-0.1585	0.379132559	-0.418059585	0.679092466	0.845613224
OMD	UCL	0.077634148	0.185751439	0.417946417	0.679364696	0.872924809
DAG1	NIH	0.0693	0.165985004	0.417507596	0.679491362	0.845749887
MUC2	UCL	-0.191289421	0.45803141	-0.417633849	0.679513895	0.872924809
KIR2DL2	UCL	-0.204975464	0.491029735	-0.417440024	0.679704709	0.872924809
H2AP	NIH	-0.105946667	0.254453442	-0.416369555	0.680314069	0.846274552
SSC4D	UCL	0.174101963	0.417982982	0.416528832	0.680370748	0.873396441
KCTD5	UCL	-0.108951419	0.262018698	-0.415815437	0.680703967	0.873440604
MANSC4	NIH	0.141633333	0.340901812	0.415466648	0.68096708	0.846274552
RGS8	NIH	-0.12408	0.298940574	-0.415065772	0.681257088	0.846274552

SPARC	NIH	0.0639	0.154044804	0.414814381	0.681438978	0.846274552
SPRR1B	NIH	0.0991	0.238958415	0.41471651	0.681509796	0.846274552
WASL	UCL	0.146170385	0.352372951	0.414817268	0.681584305	0.873704573
GPNMB	UCL	0.051234121	0.123569857	0.414616653	0.68165204	0.873704573
XG	UCL	0.054085994	0.13057535	0.414212897	0.681806408	0.873704573
AKT2	NIH	0.12486	0.301552463	0.414057304	0.681986867	0.846274552
SOX9	NIH	0.112253333	0.271126331	0.414025938	0.68200957	0.846274552
MZT1	NIH	0.076433333	0.184744342	0.413724893	0.682227485	0.846274552
CCL21	UCL	-0.085191106	0.205862225	-0.413825827	0.682254529	0.873895701
HCG22	UCL	0.05371708	0.130750161	0.410837577	0.683598731	0.875233942
TGFA	NIH	-0.08674	0.210791219	-0.411497217	0.683840885	0.847916315
GNAS	UCL	-0.053865426	0.131075502	-0.41094961	0.684311665	0.875520376
GPR101	NIH	-0.065253333	0.158906859	-0.410638872	0.68446295	0.848328021
SMOC2	UCL	-0.061932127	0.150800708	-0.410688568	0.684464316	0.875520376
ARHGEF5	UCL	0.086737228	0.211985124	0.409166581	0.684721033	0.875520376
LY6D	NIH	-0.06962	0.169869985	-0.409842857	0.685040045	0.848683665
INPP5J	UCL	0.082270658	0.201062865	0.409178783	0.685206182	0.875644989
RPGR	UCL	0.108738483	0.265795231	0.409106222	0.685417631	0.875644989
FGF2	NIH	0.059406667	0.145304334	0.408843046	0.685765163	0.849089212
ADAM23	NIH	0.05468	0.133838884	0.408550927	0.685977081	0.849089212
ECI2	UCL	-0.135606865	0.332210047	-0.408196158	0.68613234	0.876175112
ADRA2A	NIH	-0.104593333	0.25623628	-0.408190961	0.686238255	0.849089212
CFI	UCL	-0.029832517	0.073320114	-0.406880398	0.686938401	0.876821373
GNPDA1	UCL	0.038320825	0.094294384	0.406395623	0.687407786	0.87703752
GAS2	UCL	0.125396898	0.308965375	0.405860681	0.687775611	0.877123957
CXCL11	UCL	0.152511887	0.375925124	0.405697511	0.688081411	0.877131254
TSHB	NIH	-0.121686667	0.300038644	-0.40556998	0.688141101	0.850227741
IL24	NIH	0.149313333	0.368208395	0.405513116	0.688182408	0.850227741
CENPJ	NIH	-0.139806667	0.345191616	-0.405011767	0.688546636	0.850227741
CFHR5	NIH	-0.051486667	0.127129841	-0.404992772	0.688560437	0.850227741
PVALB	NIH	0.13678	0.337793384	0.404922081	0.688611801	0.850227741
JMJD1C	UCL	-0.07970929	0.197453076	-0.403687255	0.68881085	0.877678341
RGCC	NIH	0.05198	0.128524876	0.404435325	0.688965514	0.850305539

TPK1	UCL	0.042874305	0.106014201	0.404420394	0.689177528	0.877762926
NXPE4	UCL	-0.042685965	0.106351368	-0.401367335	0.690073568	0.877898511
C5	UCL	-0.019263104	0.048088432	-0.40057667	0.690256014	0.877898511
LYAR	UCL	0.100835538	0.25047495	0.402577336	0.690366664	0.877898511
MATN3	UCL	0.0526396	0.13091885	0.402078079	0.690597094	0.877898511
PCOLCE	UCL	0.075180304	0.1870478	0.401930973	0.690785692	0.877898511
SPTBN2	NIH	0.091673333	0.22818492	0.401750183	0.690918023	0.852226935
PPP1R14D	UCL	-0.075505431	0.188620691	-0.400303012	0.691112662	0.877932338
IL18RAP	NIH	0.323286667	0.805314422	0.401441546	0.691142588	0.852226935
SNAP29	NIH	0.13124	0.327206123	0.401092739	0.691396416	0.852226935
LYSMD3	UCL	-0.184655221	0.461904635	-0.399769146	0.692423486	0.878756876
DLK1	NIH	0.086093333	0.215423959	0.399646045	0.69244957	0.85316554
BST2	UCL	0.116425827	0.29113311	0.399905827	0.692560079	0.878756876
LGMN	UCL	0.050527821	0.126728002	0.398710782	0.693239025	0.878756876
MYOC	NIH	-0.101226667	0.254002649	-0.398526028	0.693265343	0.853811001
FNDC1	UCL	0.083808558	0.210321263	0.39847877	0.693265597	0.878756876
IL36G	NIH	0.116473333	0.292643281	0.398004468	0.693645353	0.853919468
IL17RB	UCL	-0.078329877	0.197252179	-0.397105254	0.694242389	0.878756876
RTN4R	UCL	-0.064628804	0.16267611	-0.397285157	0.694314102	0.878756876
CLSPN	UCL	0.091756627	0.231151904	0.396953804	0.694349044	0.878756876
EPHB4	UCL	0.035256858	0.088840234	0.396856881	0.694808099	0.878756876
ARID4B	UCL	-0.083029805	0.209848952	-0.395664617	0.694853859	0.878756876
NPHS2	NIH	0.198313333	0.50067949	0.39608839	0.69504211	0.854836252
KAZN	NIH	0.112013333	0.282865274	0.39599535	0.695109961	0.854836252
GCHFR	UCL	-0.065732136	0.165879831	-0.396263584	0.695154742	0.878756876
PSMD5	NIH	0.116746667	0.294978487	0.395780274	0.695266818	0.854836252
KYAT1	UCL	-0.089115918	0.225543403	-0.395116491	0.695420374	0.878756876
CEP152	UCL	-0.102580398	0.260266727	-0.394135661	0.695541202	0.878756876
MTPN	UCL	-0.0484099	0.122532836	-0.395076958	0.695670001	0.878756876
CAMLG	NIH	-0.070366667	0.178072265	-0.395157925	0.695720782	0.855034995
MEPE	NIH	0.032273333	0.081812717	0.394478201	0.696216729	0.855285146
CDKN1A	NIH	0.09472	0.240545567	0.393771547	0.69673247	0.855299202
AMIGO1	NIH	0.07094	0.180205703	0.393661237	0.696812991	0.855299202

DLGAP5	UCL	-0.153246566	0.389544342	-0.393399542	0.697207449	0.879670597
FURIN	UCL	0.043468352	0.110740377	0.392524872	0.697239998	0.879670597
FGF6	UCL	-0.058280584	0.148521084	-0.39240613	0.697296193	0.879670597
S100A3	NIH	-0.045933333	0.116936268	-0.392806561	0.697436989	0.855706037
TPSD1	NIH	-0.068466667	0.174562517	-0.392218604	0.697866381	0.855873864
F12	NIH	0.082773333	0.211449231	0.391457245	0.698422562	0.85619698
LAMP1	UCL	0.063989615	0.164807254	0.388269405	0.699288837	0.879835258
IDS	UCL	-0.019882595	0.050921121	-0.390458695	0.699425661	0.879835258
NRTN	UCL	0.079276469	0.203786452	0.389017366	0.699863351	0.879835258
FEN1	UCL	-0.16326675	0.419550163	-0.38914715	0.699905845	0.879835258
MEGF9	UCL	-0.021874302	0.056177369	-0.389379261	0.7000365	0.879835258
MME	UCL	-0.170552779	0.438168797	-0.389239901	0.700168749	0.879835258
CEACAM18	UCL	0.107123364	0.275908317	0.388257104	0.700207959	0.879835258
SEZ6	NIH	-0.0645	0.165851636	-0.388901801	0.700290589	0.857298261
GUK1	UCL	0.047507435	0.122770556	0.386961145	0.700411863	0.879835258
AGBL2	NIH	-0.111113333	0.285847609	-0.38871528	0.700427011	0.857298261
STX1B	NIH	-0.09304	0.239555291	-0.38838633	0.70066763	0.857298261
ECM1	NIH	0.071033333	0.182944867	0.388277269	0.700747412	0.857298261
CTSH	NIH	-0.126513333	0.326043125	-0.38802638	0.700930961	0.857298261
PAIP2B	UCL	0.063818534	0.165075022	0.386603213	0.701169149	0.879835258
ATP5PO	NIH	0.08176	0.210943328	0.387592255	0.701248608	0.857298261
CEND1	NIH	0.074326667	0.191848939	0.387422871	0.70137256	0.857298261
NECTIN2	UCL	-0.055362721	0.142761647	-0.387798279	0.70147076	0.879835258
CHRM1	UCL	-0.060943666	0.157903482	-0.385955174	0.701659242	0.879835258
RET	NIH	-0.054706667	0.141389215	-0.386922487	0.701738781	0.857387608
GBP6	UCL	0.055292909	0.143558159	0.38516034	0.701958554	0.879835258
PTS	UCL	0.110381297	0.284904081	0.387433192	0.702052016	0.879835258
CFP	UCL	0.026290595	0.068051843	0.386331866	0.702092296	0.879835258
DDT	UCL	-0.079823116	0.206654852	-0.386262967	0.70220952	0.879835258
TNFRSF6B	UCL	-0.098816425	0.255787298	-0.386322642	0.702282907	0.879835258
DAAM1	NIH	0.121086667	0.314046991	0.385568625	0.70273001	0.858240201
CD3D	UCL	-0.070756395	0.183986244	-0.384574378	0.702913068	0.879835258
RAB3GAP1	UCL	0.082508012	0.214740489	0.384221964	0.702999567	0.879835258

KIAA2013	UCL	0.068712307	0.178598061	0.384731538	0.703145796	0.879835258
CLEC1A	UCL	-0.059675364	0.155226914	-0.384439541	0.70357241	0.879992364
HBZ	NIH	0.1551	0.403807407	0.384093994	0.703810268	0.858817085
FCN1	NIH	0.186746667	0.48664787	0.383740849	0.704069063	0.858817085
FCAMR	NIH	0.116006667	0.302319913	0.383721554	0.704083204	0.858817085
FAM3C	UCL	0.043596256	0.114040496	0.382287499	0.705044239	0.881210256
CCL5	NIH	0.112566667	0.294496763	0.382233969	0.70517375	0.85957273
BAIAP2	NIH	0.111846667	0.292734969	0.38207484	0.705290445	0.85957273
MRC1	UCL	0.041801501	0.109365646	0.382217837	0.705333575	0.881210256
SIGLEC1	UCL	-0.071990095	0.188478382	-0.381954122	0.705471246	0.881210256
CSF2RA	UCL	0.114518021	0.300102903	0.381595846	0.705752039	0.881210256
IL33	UCL	0.066236937	0.174521104	0.379535402	0.706241844	0.881445307
SCARF2	NIH	0.060173333	0.158155343	0.38046981	0.706467881	0.860416879
MMP9	NIH	-0.08026	0.211406417	-0.379647889	0.707071122	0.860416879
CLSTN1	UCL	-0.136713332	0.360777826	-0.378940506	0.707259685	0.882218017
VSTM2B	NIH	-0.099133333	0.26137582	-0.379275073	0.70734481	0.860416879
PIGR	NIH	-0.05954	0.156995161	-0.379247359	0.707365158	0.860416879
IL18	NIH	0.066833333	0.176296665	0.379095846	0.707476397	0.860416879
TFF2	UCL	0.078345736	0.206567977	0.379273388	0.707492943	0.882218017
THBD	UCL	-0.042059669	0.111015883	-0.378861723	0.707774579	0.882218017
FGA	NIH	0.014386667	0.038022905	0.378368422	0.70801056	0.860416879
PGA4	NIH	-0.130386667	0.344641638	-0.378325345	0.708042197	0.860416879
ADAM8	UCL	0.056402015	0.149116777	0.378240568	0.708251494	0.882218017
NID2	UCL	-0.115490013	0.305985972	-0.377435647	0.70837006	0.882218017
VIPR1	NIH	0.039926667	0.10569456	0.377755171	0.708461001	0.860568284
SIT1	UCL	-0.07065472	0.188048302	-0.375726444	0.709800491	0.882993714
CDK1	UCL	-0.070747497	0.189214292	-0.373901441	0.709889581	0.882993714
CEBPA	UCL	0.063619986	0.169388884	0.375585365	0.709899154	0.882993714
GRIK2	NIH	0.088693333	0.236220498	0.375468404	0.710141608	0.862251641
GCNT1	UCL	0.073435202	0.196083088	0.374510633	0.710981171	0.883919236
STX4	NIH	0.102153333	0.272940749	0.374269265	0.711023484	0.862787803
THY1	UCL	0.049966914	0.133580286	0.374059047	0.711248048	0.883919236
SIGLEC15	NIH	-0.180453333	0.482664658	-0.373868959	0.711317969	0.862787803

MRPL46	NIH	-0.035153333	0.09407716	-0.373664908	0.711468096	0.862787803
MUC13	UCL	-0.088002052	0.237001829	-0.371313808	0.712495976	0.884894529
LILRA4	UCL	-0.109530281	0.293931854	-0.37263835	0.71263829	0.884894529
CGREF1	UCL	0.069909904	0.188445373	0.370982334	0.71354417	0.885643147
ARHGEF12	NIH	0.149953333	0.404378368	0.37082432	0.713559242	0.864965099
OSM	NIH	0.100213333	0.271774634	0.36873689	0.715097388	0.866273442
BIN2	NIH	-0.130226667	0.353888282	-0.367988072	0.71564946	0.866273442
EFNB2	NIH	0.073513333	0.200080593	0.36741861	0.716069406	0.866273442
REG1B	NIH	0.076953333	0.209489564	0.367337311	0.716129366	0.866273442
NPTXR	NIH	-0.0498	0.13559319	-0.367275082	0.716175264	0.866273442
VASH1	NIH	0.041006667	0.111750215	0.36694933	0.716415541	0.866273442
CAMLG	UCL	-0.110430771	0.301209496	-0.366624467	0.71665969	0.889118961
DEFB103A_DEFB103B	NIH	0.113806667	0.310673123	0.366322859	0.716877714	0.866474096
TNFSF8	UCL	0.07244533	0.19777567	0.366300516	0.716952922	0.889118961
PDIA5	NIH	-0.079593333	0.217994872	-0.365115623	0.717768649	0.867172351
STK4	NIH	0.061893333	0.169693173	0.364736731	0.718048354	0.867172351
CAMKK1	UCL	0.101071842	0.277700436	0.363959971	0.718249869	0.890349604
RAB6A	NIH	-0.067126667	0.184697478	-0.363441165	0.719005063	0.867700822
PDLIM7	NIH	0.1758	0.483843355	0.363340734	0.719079245	0.867700822
GRK5	UCL	-0.083539615	0.229787589	-0.363551466	0.719104171	0.89072357
HYOU1	UCL	-0.039834919	0.109565888	-0.363570446	0.719161007	0.89072357
LIPF	NIH	-0.10394	0.286478967	-0.362818957	0.719464696	0.867807933
CDH1	UCL	0.04799992	0.13255072	0.362124927	0.720090456	0.891425939
PZP	UCL	0.054226114	0.149861635	0.361841201	0.720338032	0.891425939
REG3A	UCL	-0.097582076	0.270095161	-0.361287762	0.720729819	0.891533331
ERBB2	NIH	0.042746667	0.118448029	0.360889641	0.720890589	0.869169404
MMUT	UCL	0.079206526	0.220711041	0.358869795	0.721569452	0.892194378
CALCOCO2	NIH	0.064593333	0.179631579	0.359587851	0.721853277	0.869971502
TEF	UCL	0.055823322	0.155509607	0.358970244	0.722027759	0.892383569
PROC	UCL	-0.031956692	0.089169432	-0.358381692	0.722648949	0.892614983
CTHRC1	NIH	0.057853333	0.161438756	0.358360871	0.722761068	0.870706805
ATP1B3	UCL	0.052850079	0.147908689	0.357315576	0.722825749	0.892614983

SNAP23	NIH	0.12168	0.340078656	0.357799579	0.723176481	0.870848583
LAMP2	UCL	-0.024903987	0.06960556	-0.357787318	0.723364794	0.892869241
PDIA3	UCL	0.02814094	0.079216766	0.355239698	0.723983757	0.892869241
MANSC1	UCL	0.043217755	0.12115758	0.35670698	0.724086301	0.892869241
NADK	NIH	0.099413333	0.279106901	0.356183716	0.724372865	0.8719303
SIGLEC6	UCL	0.054224857	0.152258703	0.356136338	0.72451465	0.892869241
TYRP1	UCL	-0.072373509	0.203366107	-0.355877927	0.72455896	0.892869241
CSF1	UCL	-0.050645999	0.14238907	-0.355687406	0.725066019	0.893117561
SELL	NIH	-0.034033333	0.096036265	-0.354380019	0.72570916	0.87317947
PIKFYVE	NIH	-0.05546	0.157004439	-0.35323842	0.726555385	0.873838199
STX7	NIH	0.074966667	0.212994609	0.351965089	0.727499673	0.874469428
MAD1L1	UCL	0.04941289	0.140625268	0.351379883	0.72753708	0.893815714
FABP2	NIH	-0.116153333	0.330239541	-0.351724488	0.72767815	0.874469428
CCL20	UCL	-0.095166142	0.270984635	-0.351186486	0.727921296	0.893815714
ESM1	UCL	0.052383219	0.148979803	0.351612889	0.727960604	0.893815714
TLR1	UCL	0.060617951	0.172759737	0.350880082	0.728056035	0.893815714
IL20RB	NIH	-0.038613333	0.109997697	-0.351037653	0.728187725	0.874722421
PLXDC2	UCL	0.040915012	0.116802386	0.350292601	0.728319727	0.893815714
EVPL	UCL	0.043115674	0.123584211	0.348876879	0.729126629	0.893815714
IL11	UCL	0.081632844	0.233837322	0.349101006	0.729310263	0.893815714
REG3A	NIH	-0.080526667	0.230451534	-0.349429944	0.729381008	0.875297151
SWAP70	NIH	0.06304	0.180611763	0.349035959	0.72967354	0.875297151
SCG2	NIH	-0.05214	0.149391631	-0.349015535	0.729688706	0.875297151
ANGPT1	UCL	0.159684519	0.458080525	0.348594864	0.729714453	0.893815714
ELN	UCL	-0.040753696	0.116910734	-0.348588145	0.729836838	0.893815714
FAM3B	NIH	0.0505	0.144790163	0.348780602	0.729863163	0.875297151
CD300A	UCL	0.044076214	0.12637983	0.348759875	0.729964127	0.893815714
KLK13	UCL	-0.078665834	0.225847867	-0.348313383	0.730267037	0.893815714
TNFRSF10A	UCL	-0.0433218	0.124380396	-0.348300869	0.73030489	0.893815714
OPTC	UCL	0.055779335	0.160496148	0.347543137	0.730308976	0.893815714
BST2	NIH	-0.079053333	0.227201227	-0.347944131	0.730484432	0.875499603
ARF6	NIH	0.162846667	0.468290165	0.347747356	0.730630609	0.875499603
FCER1A	UCL	0.041486794	0.119481377	0.34722394	0.730883736	0.893815714

SNX2	UCL	-0.083072109	0.23913967	-0.347379041	0.730972839	0.893815714
DSG3	UCL	0.05102984	0.146905166	0.347365865	0.730983982	0.893815714
PMS1	UCL	-0.060313072	0.174016589	-0.346593807	0.731476153	0.893815714
HMGCS1	UCL	0.093141162	0.269146239	0.346061541	0.731557146	0.893815714
GLP1R	UCL	-0.059040853	0.170525436	-0.346229014	0.731685536	0.893815714
TNC	UCL	0.075941079	0.219175951	0.34648454	0.731750615	0.893815714
KIR3DL2	NIH	-0.057886667	0.167203951	-0.34620394	0.731777515	0.876514837
DCUN1D1	UCL	0.062198714	0.179613329	0.346292308	0.732054334	0.893815714
CD163	UCL	-0.087216067	0.252603903	-0.345268091	0.732567795	0.894069171
GSTM4	NIH	-0.185533333	0.53770018	-0.345049788	0.732635573	0.876968157
APPL2	NIH	0.10022	0.290586785	0.344888361	0.732755615	0.876968157
IL7R	UCL	-0.064177775	0.186503296	-0.344110675	0.73355367	0.894898738
ICAM3	UCL	0.040328892	0.11759062	0.342960107	0.734326518	0.895467839
NMI	NIH	-0.131326667	0.383508893	-0.342434475	0.734581241	0.878781369
CDH15	NIH	0.11274	0.329606253	0.342044482	0.734871531	0.878781369
ALDH2	UCL	-0.069009395	0.201880145	-0.341833492	0.735086166	0.896020377
CXCL6	NIH	0.11694	0.343988048	0.339953672	0.736428501	0.880278735
CEP164	UCL	0.062491164	0.184486105	0.338731003	0.736466117	0.897328245
CD200	NIH	-0.044406667	0.130779049	-0.339554897	0.736725587	0.880278735
TNFRSF10C	UCL	-0.069957176	0.20614562	-0.339358052	0.737000866	0.897605638
SCPEP1	NIH	0.089046667	0.263230775	0.338283648	0.737672944	0.880875181
COL9A1	NIH	0.064453333	0.190646975	0.338076873	0.737827075	0.880875181
TAP1	UCL	0.044494112	0.132520497	0.335752677	0.738312084	0.898171161
STEAP4	UCL	0.060617003	0.180216371	0.336356806	0.738352312	0.898171161
CPA4	UCL	0.06846773	0.202862525	0.337508022	0.738387034	0.898171161
PRDX1	NIH	-0.086993333	0.258160098	-0.336974358	0.738649087	0.881496769
GGACT	NIH	0.078213333	0.232531484	0.336355886	0.739110345	0.881687503
ITPRIP	NIH	0.04344	0.129353696	0.335823415	0.739507541	0.881744035
ANP32C	NIH	0.082166667	0.24491965	0.335484175	0.739760637	0.881744035
BSND	UCL	0.101833641	0.305415067	0.333427037	0.740057201	0.899795611
TNFRSF17	UCL	0.062533664	0.186762927	0.334829107	0.740338161	0.899795611
NID1	NIH	0.0445	0.13325326	0.333950553	0.740905186	0.882748542
GDF2	UCL	0.053876288	0.161450345	0.333701907	0.741118473	0.900369616

CXCL17	NIH	0.089213333	0.267949152	0.332948744	0.741653168	0.882946055
FRMD4B	NIH	-0.104126667	0.312767899	-0.332919928	0.741674687	0.882946055
GYS1	NIH	0.112866667	0.339712327	0.332241893	0.74218108	0.883189446
PEAR1	NIH	0.02874	0.087362353	0.328974655	0.744622876	0.885733602
PEAR1	UCL	0.020686501	0.062987881	0.328420338	0.745072116	0.904796758
LAT	NIH	0.036046667	0.109824576	0.328220402	0.745186959	0.885733602
SPART	NIH	0.100066667	0.304926773	0.328166221	0.745227485	0.885733602
CCL4	NIH	-0.06136	0.187370468	-0.327479568	0.745741147	0.8859841
LAP3	UCL	-0.05620743	0.171941707	-0.326898174	0.745894927	0.905419798
NMNAT1	UCL	-0.070756926	0.217294903	-0.325626255	0.746609459	0.905664086
ADAM12	UCL	-0.058303538	0.179017824	-0.325685657	0.746862357	0.905664086
CLNS1A	NIH	0.0968	0.297073596	0.325845182	0.746964254	0.886557093
LDLR	UCL	0.067284454	0.206400078	0.325990451	0.747025697	0.905664086
SIRPA	NIH	0.073826667	0.226739563	0.325601168	0.747146922	0.886557093
CCL13	NIH	-0.060046667	0.184634226	-0.325219586	0.747432602	0.886557093
CDC123	NIH	0.116073333	0.3569123	0.32521528	0.747435826	0.886557093
CES1	UCL	0.108402906	0.333708131	0.324843466	0.747917385	0.906369202
SLAMF1	UCL	0.11231594	0.347256021	0.323438425	0.748641844	0.906871161
CST5	UCL	-0.062516708	0.193548895	-0.323002142	0.749283877	0.906976089
HLA-A	NIH	-0.043293333	0.134157284	-0.322705797	0.749315523	0.888224054
NAAA	UCL	-0.101200805	0.313453637	-0.322857333	0.749349044	0.906976089
KLRK1	NIH	-0.066226667	0.205490375	-0.322285979	0.749630137	0.888224054
DLGAP5	NIH	-0.200306667	0.621889573	-0.322093625	0.749774302	0.888224054
KIFBP	NIH	0.05804	0.180406455	0.321717979	0.750055868	0.888224054
TSC1	UCL	0.1040942	0.323670095	0.321605862	0.750080438	0.907119725
IL36A	UCL	0.084649207	0.263455584	0.321303523	0.750088394	0.907119725
PSAPL1	NIH	0.06464	0.201312753	0.321092424	0.750524831	0.888304602
PSG1	NIH	0.168186667	0.524485748	0.320669661	0.750841822	0.888304602
ENPEP	UCL	-0.047332241	0.147858932	-0.320117563	0.751114053	0.907640746
LRG1	UCL	-0.04275066	0.133381537	-0.320514078	0.751140254	0.907640746
TMEM25	NIH	-0.08642	0.269967328	-0.32011281	0.751259419	0.888304602
PRELP	NIH	0.025206667	0.078769072	0.320007155	0.751338661	0.888304602
MMP3	NIH	0.052886667	0.166058982	0.318481217	0.752483437	0.889298607

SDC1	UCL	0.063742872	0.200424326	0.318039596	0.752982588	0.909490953
TBL1X	NIH	-0.074833333	0.235758184	-0.317415633	0.75328319	0.889884221
CCL13	UCL	0.11373103	0.358697688	0.317066527	0.753598777	0.909859243
LACRT	NIH	0.108253333	0.341758049	0.316754305	0.753779678	0.890111247
IL3	NIH	0.110366667	0.34911217	0.31613526	0.754244519	0.890256867
PALM2	UCL	-0.067418569	0.213164581	-0.316274725	0.754729305	0.910733894
ADGRV1	NIH	-0.116253333	0.368556486	-0.315428809	0.754775108	0.890256867
DOCK9	UCL	0.098450301	0.312709811	0.314829587	0.754946365	0.910733894
SYNGAP1	NIH	0.13762	0.437022241	0.314903881	0.755169441	0.890256867
EPCAM	NIH	-0.134086667	0.426253714	-0.314570084	0.755420229	0.890256867
AGR3	NIH	0.086366667	0.274559922	0.314563998	0.755424801	0.890256867
CFHR2	UCL	0.064177684	0.204682676	0.313547221	0.756266331	0.911475688
TEK	UCL	-0.030045757	0.096040317	-0.312845251	0.756724426	0.911475688
DENND2B	UCL	0.082313141	0.263403369	0.312498437	0.756768525	0.911475688
MAP2K6	NIH	0.133773333	0.42771917	0.312759733	0.756780851	0.891495767
TMPRSS5	UCL	0.056690695	0.18118372	0.312890669	0.756808585	0.911475688
IL17D	NIH	-0.043466667	0.139224557	-0.31220546	0.757197591	0.891627598
C1QTNF6	UCL	-0.04420125	0.142133344	-0.310984382	0.75778585	0.911507819
C1QBP	UCL	0.058403371	0.187110173	0.312133594	0.757949521	0.911507819
REXO2	UCL	-0.030220939	0.096991806	-0.311582396	0.758082793	0.911507819
UHRF2	UCL	0.04136138	0.133672279	0.309423766	0.758147609	0.911507819
MUC2	NIH	0.182826667	0.588176672	0.310836311	0.758227327	0.892421847
TNFRSF11B	UCL	0.040073646	0.128931711	0.310812956	0.758394463	0.911507819
ICAM5	NIH	-0.058926667	0.18986909	-0.310354185	0.758590041	0.892421847
MUCL3	NIH	-0.111386667	0.359205355	-0.310091888	0.758787396	0.892421847
JAM3	NIH	-0.040946667	0.132251603	-0.309611875	0.759148607	0.892487812
PPM1A	UCL	-0.03228867	0.104586895	-0.308725774	0.759188171	0.912086733
KRT17	NIH	0.055586667	0.180684149	0.307645507	0.760628881	0.893179002
NPM1	UCL	-0.084228434	0.275434744	-0.305801777	0.760889507	0.913755147
PLXNA4	NIH	-0.066413333	0.21619125	-0.307197139	0.76096654	0.893179002
DCTPP1	NIH	-0.036686667	0.119651898	-0.306611656	0.761407531	0.893179002
SCRN1	NIH	0.069553333	0.226873925	0.306572619	0.761436936	0.893179002
PITHD1	NIH	0.141826667	0.46276977	0.30647349	0.76151161	0.893179002

DNM1	NIH	0.138573333	0.452266212	0.306397713	0.761568695	0.893179002
CCL4	UCL	0.072776697	0.237801445	0.306039759	0.761712871	0.914205642
TREM2	UCL	0.086924183	0.28404794	0.30601941	0.761972588	0.914205642
BRD3	NIH	0.055706667	0.182660276	0.304974173	0.76264133	0.894078513
FABP1	UCL	-0.133836161	0.439070817	-0.3048168	0.76274619	0.914205642
GFRA2	UCL	-0.041626025	0.136640803	-0.304638325	0.76291253	0.914205642
FAP	NIH	-0.03756	0.123481719	-0.304174579	0.763244034	0.89438829
PQBP1	UCL	0.09904632	0.325942276	0.303876874	0.763440906	0.914205642
IL18R1	UCL	0.05152876	0.16946638	0.304064793	0.763539439	0.914205642
RABEPK	UCL	0.077976861	0.25696912	0.30344837	0.763549963	0.914205642
DCLRE1C	NIH	-0.08178	0.269509441	-0.303440205	0.763797711	0.89438829
CEACAM21	UCL	0.110951601	0.365580782	0.303494073	0.763848566	0.914205642
IL17RB	NIH	-0.070966667	0.234142861	-0.30309131	0.764060803	0.89438829
CPB2	UCL	0.028947384	0.095501945	0.303107797	0.764079502	0.914205642
LRP11	UCL	0.048274212	0.159419914	0.302811681	0.764419748	0.914238512
KLK8	NIH	-0.045913333	0.151749704	-0.302559623	0.764461789	0.89438829
SULT2A1	NIH	0.091333333	0.302102872	0.302325273	0.764638551	0.89438829
ACHE	NIH	-0.051806667	0.171437111	-0.302190503	0.764740209	0.89438829
IGF2BP3	UCL	0.042289565	0.140314269	0.301391761	0.765067971	0.914573936
MAP1LC3A	NIH	0.075933333	0.252423591	0.300817102	0.765776422	0.894828317
SERPINE1	UCL	0.118427242	0.394103808	0.300497584	0.765802989	0.914573936
ART5	NIH	-0.032213333	0.107134956	-0.300679952	0.765879924	0.894828317
DEFB116	NIH	0.075773333	0.252178075	0.300475501	0.766034225	0.894828317
PLAUR	UCL	-0.034660683	0.115443241	-0.30024004	0.766402588	0.914573936
TOP1MT	NIH	0.075186667	0.250952427	0.299605259	0.766691109	0.895238122
BMP4	NIH	-0.048753333	0.162977994	-0.29914059	0.767041928	0.895290359
LAMA1	UCL	0.10141569	0.33908856	0.299083194	0.767136706	0.914573936
CEP350	UCL	0.057264937	0.192131849	0.298050205	0.767351323	0.914573936
SMPD1	NIH	-0.072506667	0.242761813	-0.298674102	0.76739417	0.895344215
GSTP1	UCL	-0.12944775	0.434557118	-0.297884316	0.76760014	0.914573936
ARID3A	UCL	-0.047611027	0.16015446	-0.297281931	0.767782094	0.914573936
DDX53	NIH	0.046253333	0.155140048	0.298139223	0.767798117	0.89545833
CD58	UCL	-0.024175061	0.081219678	-0.297650287	0.768025538	0.914573936

SCN4B	NIH	-0.054066667	0.18164849	-0.297644459	0.768171827	0.895537104
STX5	UCL	-0.054756517	0.183956045	-0.297660871	0.768232739	0.914573936
CLNS1A	UCL	0.125593589	0.422087975	0.297553108	0.768243611	0.914573936
GPRC5C	NIH	-0.11104	0.373869303	-0.297002185	0.76865704	0.895560153
DCC	NIH	0.025686667	0.086543113	0.296807751	0.768803947	0.895560153
GCLM	UCL	-0.049915933	0.168393564	-0.296424231	0.768847633	0.914573936
CD1C	UCL	0.041345992	0.139304106	0.29680383	0.768906266	0.914573936
BTLA	UCL	-0.089694375	0.303399642	-0.295631116	0.769021865	0.914573936
GBP1	UCL	0.110102767	0.37127297	0.296554761	0.769080649	0.914573936
SPINK5	NIH	0.04658	0.157510379	0.295726544	0.769621023	0.895736512
PTPN9	NIH	0.064553333	0.218528627	0.295399895	0.769867927	0.895736512
COL2A1	UCL	-0.078901834	0.267250034	-0.295236012	0.769993114	0.915286649
GNLY	NIH	0.07012	0.23765628	0.295047958	0.770133973	0.895736512
IGFBP1	NIH	0.12532	0.424832712	0.2949867	0.770180283	0.895736512
PSAP	UCL	-0.032329684	0.110052345	-0.293766427	0.770405042	0.915404039
EZR	NIH	-0.039453333	0.134918372	-0.292423729	0.77211865	0.897211993
KIRREL1	NIH	0.137653333	0.471170745	0.292151698	0.772324475	0.897211993
ATP1B2	NIH	0.052286667	0.179223772	0.291739572	0.77263633	0.897211993
IFNGR1	NIH	0.02346	0.080526763	0.291331715	0.772944993	0.897211993
PLA2G1B	UCL	-0.050754344	0.174172665	-0.291402468	0.773010367	0.9161594
DCDC2C	NIH	0.08042	0.276138414	0.291230759	0.773021402	0.897211993
RYR1	UCL	0.035218398	0.121192748	0.29059823	0.773151796	0.9161594
APOA2	NIH	0.06258	0.215142694	0.290876715	0.773289379	0.897211993
BLNK	UCL	0.065679999	0.226294036	0.290241847	0.773519252	0.9161594
BDNF	UCL	-0.161526763	0.556879296	-0.290057045	0.773753001	0.9161594
IL21R	UCL	-0.036707051	0.127034164	-0.288954166	0.774120133	0.9161594
ATP6V1G2	UCL	0.212001376	0.731777921	0.289707259	0.774179136	0.9161594
CTSE	UCL	0.067786966	0.234064853	0.289607624	0.774299813	0.9161594
PRAME	NIH	-0.054166667	0.187318092	-0.289169434	0.774582024	0.89835544
PLA2G10	UCL	0.081187128	0.280612507	0.28932113	0.77458458	0.9161594
SPINK8	UCL	0.03248027	0.112915709	0.287650586	0.774676064	0.9161594
INSL5	UCL	0.102444876	0.355349766	0.288293072	0.774984212	0.9161594
IL18BP	UCL	0.046844018	0.162353378	0.288531217	0.775218033	0.9161594

PPIB	NIH	0.0629	0.218385912	0.288022242	0.775450976	0.898527585
RGL2	UCL	0.087460887	0.304018158	0.287683104	0.775500677	0.9161594
CXCL17	UCL	-0.037696564	0.130978876	-0.287806441	0.775660895	0.9161594
PPY	NIH	0.104253333	0.36246006	0.287627093	0.775750354	0.898527585
CFD	UCL	-0.032565622	0.113385393	-0.287211795	0.776054964	0.9161594
IRAG2	NIH	0.082146667	0.286061654	0.287164202	0.7761011	0.898527585
DUOX2	UCL	-0.054092455	0.189031686	-0.286155493	0.776212539	0.9161594
SERPINH1	NIH	0.099486667	0.346664116	0.286982881	0.776238506	0.898527585
GZMH	NIH	-0.097586667	0.340087125	-0.286946078	0.776266396	0.898527585
TF	UCL	0.025902477	0.09025954	0.286977721	0.776290616	0.9161594
CXCL8	UCL	-0.067692723	0.236072579	-0.286745387	0.776369085	0.9161594
CCL22	UCL	0.079192484	0.276723151	0.286179467	0.777003522	0.916328472
PRDX2	UCL	-0.043961615	0.154350988	-0.284815895	0.777139337	0.916328472
NT5E	NIH	-0.058733333	0.206324099	-0.284665406	0.777995343	0.900099645
TXNL1	UCL	0.061727573	0.217528655	0.283767546	0.778098003	0.916711711
CDCP1	UCL	0.084517522	0.29725343	0.284328163	0.778382026	0.916711711
C7	UCL	0.037976463	0.133599405	0.284256226	0.778413638	0.916711711
GBP4	NIH	0.06778	0.238636587	0.284030211	0.778477083	0.900099645
SEMA4C	NIH	0.024826667	0.087437214	0.283937073	0.778547727	0.900099645
SIGLEC9	UCL	0.037620498	0.13284686	0.28318696	0.779201658	0.916711711
TPP1	UCL	-0.046949876	0.165770033	-0.283222938	0.779209039	0.916711711
BCAM	UCL	0.02943592	0.104045886	0.282912873	0.779346083	0.916711711
PSAPL1	UCL	-0.056138292	0.19886308	-0.282296202	0.779921633	0.916764797
GFAP	NIH	-0.06194	0.219966279	-0.281588616	0.780329652	0.901803331
DEFB118	UCL	-0.075358821	0.267875329	-0.281320499	0.780631351	0.916764797
KLK15	UCL	-0.053350462	0.189784166	-0.281111239	0.780658184	0.916764797
BMP10	UCL	0.035535875	0.126503741	0.280907698	0.780828527	0.916764797
ITIH5	NIH	-0.08652	0.307984707	-0.28092304	0.780834889	0.902030825
PRG3	UCL	0.073134738	0.260450598	0.280800806	0.781012333	0.916764797
HBZ	UCL	0.104521302	0.372649672	0.2804814	0.781273044	0.916764797
SELPLG	UCL	0.03195251	0.114271196	0.279619988	0.781986449	0.917233704
SCAMP3	NIH	0.09402	0.336944038	0.279037435	0.782266775	0.903328195
ENPP7	UCL	0.118654776	0.425612791	0.278785738	0.78259262	0.917576506

CTSZ	UCL	0.037896325	0.136620836	0.277383201	0.783594539	0.917774295
MNDA	UCL	-0.083220138	0.301162521	-0.276329663	0.783666978	0.917774295
CD80	UCL	-0.048973765	0.176709427	-0.27714291	0.783703264	0.917774295
CLEC10A	UCL	0.053557001	0.193623853	0.276603324	0.784236799	0.918031303
PSMA1	NIH	-0.06254	0.227046902	-0.275449696	0.784993371	0.905107163
ADAM8	NIH	-0.040713333	0.147848642	-0.27537171	0.785052669	0.905107163
EDIL3	NIH	-0.0413	0.15003585	-0.275267545	0.785131875	0.905107163
BHLHE40	NIH	-0.149153333	0.541866671	-0.275258364	0.785138857	0.905107163
FCGR2B	NIH	-0.08826	0.321062877	-0.274899424	0.785411811	0.905107163
IL6R	UCL	0.042833972	0.155814418	0.274903775	0.785489395	0.919096419
ATP5F1D	NIH	-0.039206667	0.142819789	-0.274518448	0.785701553	0.905107163
CDH22	UCL	-0.037384125	0.136573497	-0.273728989	0.785775556	0.919096419
CACNA1C	NIH	-0.062926667	0.229524467	-0.27416104	0.785973399	0.905107163
IL4	UCL	0.092686871	0.339808437	0.272762124	0.786342279	0.919391393
ATXN10	NIH	-0.079606667	0.292032678	-0.272595065	0.787164812	0.905957316
TMED1	UCL	-0.051829579	0.191143248	-0.27115569	0.787616856	0.919702048
RNASEH2A	UCL	0.126638477	0.465189311	0.272229981	0.787625789	0.919702048
MEP1A	NIH	-0.107746667	0.39622884	-0.271930399	0.787670657	0.905957316
PCDH7	UCL	0.044134661	0.162273471	0.271977058	0.787715799	0.919702048
S100A16	NIH	0.080853333	0.297755996	0.271542251	0.787966101	0.905957316
TGFBR1	UCL	-0.029531567	0.109280261	-0.270236971	0.787971397	0.919702048
VAMP5	UCL	0.052416759	0.19311652	0.271425559	0.788275846	0.919702048
CKB	NIH	0.070606667	0.260469045	0.271075078	0.788321739	0.905957316
CRACR2A	NIH	-0.108666667	0.401059734	-0.270948833	0.788417852	0.905957316
LAIR1	UCL	-0.038247779	0.14119712	-0.270882145	0.788644208	0.919702048
CCN4	NIH	-0.054573333	0.201762527	-0.270482998	0.78877253	0.905957316
ARHGEF5	NIH	0.0673	0.248943764	0.270342181	0.788879755	0.905957316
SERPINA1	UCL	-0.005215378	0.019362849	-0.269349724	0.789014446	0.919702048
AAMDC	UCL	-0.051252125	0.190090383	-0.269619767	0.789339917	0.919702048
PTH	UCL	0.08183606	0.30371813	0.269447398	0.789532405	0.919702048
CD7	UCL	-0.053803467	0.199964793	-0.269064699	0.78975441	0.919702048
PGLYRP4	NIH	0.07524	0.280853371	0.267897799	0.79074169	0.907739184
HMBS	UCL	-0.061075996	0.228697313	-0.267060401	0.791109359	0.920913045

GASK1A	NIH	-0.07634	0.285814975	-0.267095872	0.791352809	0.908084333
KLK6	NIH	-0.038133333	0.143545945	-0.26565246	0.79245312	0.908990344
MRPL58	UCL	0.10535319	0.397142898	0.265277788	0.792713143	0.921978663
LGALS3BP	UCL	0.045669033	0.172218179	0.265181254	0.792968018	0.921978663
ATRN	UCL	0.016459164	0.062142681	0.264860868	0.793035983	0.921978663
ANXA10	NIH	-0.103633333	0.391597494	-0.264642483	0.793223284	0.909517094
LGALS4	UCL	-0.071419378	0.26993047	-0.26458435	0.793286465	0.921978663
MLLT1	UCL	-0.086502224	0.327280055	-0.264306434	0.793698419	0.92209081
IGFL4	NIH	0.05734	0.217357899	0.263804538	0.793862426	0.909893258
IGFBP3	UCL	0.035693662	0.135798371	0.262843076	0.794662196	0.922843703
TSC1	NIH	0.071713333	0.273449232	0.262254653	0.795044983	0.910545771
PLCB1	UCL	-0.073889082	0.282851019	-0.261229684	0.795482915	0.922943354
ITGA5	NIH	0.03204	0.122466511	0.261622543	0.795527423	0.910545771
IL20	UCL	-0.029445952	0.112880061	-0.260860525	0.795538724	0.922943354
HCG22	NIH	-0.062166667	0.237652434	-0.261586492	0.79555494	0.910545771
CEACAM16	UCL	0.099814749	0.381453697	0.26166937	0.795695263	0.922943354
OBP2B	NIH	-0.082733333	0.317015344	-0.260975801	0.796021116	0.910545771
CLEC14A	NIH	-0.03614	0.138516147	-0.260908211	0.796072717	0.910545771
MPHOSPH8	NIH	0.06478	0.248744741	0.260427616	0.796439644	0.910545771
TDP1	UCL	-0.036565728	0.141016509	-0.259301047	0.796693398	0.923734551
GAS6	NIH	0.029006667	0.111541191	0.260053407	0.796725379	0.910545771
CD70	NIH	-0.04756	0.183077259	-0.259781036	0.796933372	0.910545771
COMMD9	UCL	-0.029864282	0.115314409	-0.258981353	0.797169498	0.923771634
STXBP3	UCL	-0.115913989	0.447712937	-0.258902479	0.797543188	0.923771634
OTOA	UCL	-0.054086778	0.208872155	-0.258946808	0.797673488	0.923771634
FCRL1	NIH	0.0696	0.268963036	0.258771618	0.797704335	0.910545771
SCGB3A2	NIH	-0.077046667	0.298342055	-0.258249433	0.798103246	0.910545771
TBC1D5	NIH	0.070693333	0.273782845	0.258209507	0.798133749	0.910545771
BCAT1	NIH	-0.029013333	0.112382651	-0.258165589	0.798167301	0.910545771
CD82	UCL	-0.04897616	0.190224304	-0.257465313	0.798487811	0.924273853
SLAMF1	NIH	0.080053333	0.311079513	0.257340423	0.798797795	0.91068802
FZD10	NIH	0.098266667	0.382081876	0.257187459	0.798914687	0.91068802
ITGAV	UCL	0.027634271	0.1074251	0.257242223	0.799001312	0.924273853

RNF149	UCL	-0.035790736	0.139255694	-0.257014526	0.799055773	0.924273853
CEND1	UCL	0.036172132	0.141807009	0.25508	0.799595805	0.924477193
CLPP	NIH	-0.042626667	0.166396981	-0.25617452	0.799688875	0.91121541
CHL1	UCL	0.028108042	0.109754308	0.25609967	0.799864119	0.924477193
NFKB2	NIH	0.049966667	0.195729725	0.255283998	0.800369671	0.911411303
GLIPR1	NIH	-0.030873333	0.121008071	-0.255134497	0.800483979	0.911411303
CA9	UCL	-0.057140889	0.224432936	-0.254601174	0.80074101	0.924722678
UBE2Z	UCL	-0.08139501	0.320286475	-0.254131898	0.801138292	0.924722678
CXCL10	UCL	0.063394806	0.249893576	0.253687216	0.801602571	0.924722678
RGS8	UCL	0.063066518	0.248731751	0.253552342	0.801657898	0.924722678
TDO2	UCL	-0.058216169	0.229799115	-0.253335045	0.801839703	0.924722678
TNFRSF14	UCL	-0.033814596	0.133606909	-0.253090172	0.802235707	0.924722678
ACP1	UCL	0.077739214	0.307658461	0.25268024	0.802291041	0.924722678
MICB_MICA	NIH	0.257406667	1.018938605	0.252622352	0.802405427	0.912888647
ODAM	NIH	0.076526667	0.302936127	0.252616508	0.802409897	0.912888647
ENTR1	NIH	0.04602	0.182464034	0.252214088	0.802717812	0.912888647
CSF1R	UCL	0.046334409	0.184702813	0.250859249	0.803835923	0.925912605
CCN2	UCL	0.057628643	0.230051276	0.250503471	0.803956959	0.925912605
FUCA1	NIH	0.079026667	0.31554629	0.250443973	0.804072609	0.913708123
SPOCK1	NIH	0.030086667	0.120164195	0.250379631	0.804121867	0.913708123
IGLON5	NIH	-0.056253333	0.224969864	-0.250048306	0.804375527	0.913708123
NRP1	UCL	-0.026761093	0.10740194	-0.249167692	0.804983922	0.926013641
ZPR1	UCL	-0.066474756	0.267914748	-0.248119062	0.805794063	0.926013641
AP2B1	NIH	0.03848	0.155061302	0.248159918	0.805821679	0.914627134
PRTG	UCL	-0.022278799	0.089726431	-0.24829695	0.805829088	0.926013641
FCN2	UCL	-0.031411678	0.126568091	-0.248180076	0.805872648	0.926013641
GLRX5	NIH	0.061793333	0.249403878	0.247764124	0.806124873	0.914627134
PLXNB3	NIH	0.043173333	0.174650548	0.247198385	0.806558304	0.914627134
QSOX1	UCL	0.029267602	0.118503816	0.246976028	0.806658313	0.926013641
GCG	NIH	-0.11736	0.47516332	-0.246988762	0.806718918	0.914627134
NUDT15	UCL	0.044197952	0.178756258	0.247252616	0.806869258	0.926013641
COL9A1	UCL	-0.043595948	0.177040023	-0.246249111	0.806940609	0.926013641
CHP1	NIH	-0.062246667	0.252637661	-0.246387124	0.807179946	0.914627134

PTPRM	UCL	0.026474465	0.107497337	0.246280196	0.807200634	0.926013641
PSRC1	UCL	-0.081980473	0.333025282	-0.246168916	0.807242507	0.926013641
STAM	NIH	0.07246	0.294391264	0.246135022	0.80737315	0.914627134
SPON2	UCL	0.044487708	0.180857114	0.245982627	0.807558828	0.926013641
AMN	UCL	-0.054537588	0.222152827	-0.245495809	0.807704975	0.926013641
CHIT1	NIH	0.096126667	0.391456367	0.245561638	0.807812621	0.914627134
PLB1	NIH	-0.078606667	0.320219839	-0.245477191	0.80787735	0.914627134
AFP	NIH	0.057993333	0.236399931	0.245318741	0.807998808	0.914627134
VCAM1	UCL	0.030655661	0.125005021	0.245235439	0.808128367	0.926013641
HS3ST3B1	UCL	-0.03577492	0.146088744	-0.244884847	0.808298616	0.926013641
RTN4IP1	NIH	0.04792	0.195814602	0.244721279	0.80845683	0.914791577
TIGIT	UCL	0.022417088	0.091910295	0.243901817	0.808479922	0.926013641
PDCD1	UCL	-0.04243343	0.173873097	-0.244048278	0.808997027	0.926242973
NAA10	NIH	-0.063433333	0.260446275	-0.243556308	0.80935011	0.91544821
PPL	UCL	-0.029980194	0.123689717	-0.242382263	0.810124447	0.927170619
DSG3	NIH	0.036506667	0.150595432	0.242415498	0.810225117	0.915584247
GUCY2C	NIH	-0.075453333	0.311892733	-0.24192078	0.810604647	0.915584247
FZD8	NIH	0.067553333	0.279304274	0.24186287	0.810649076	0.915584247
GJA8	UCL	-0.03763797	0.156678359	-0.240224432	0.811033498	0.927469913
MASP1	UCL	-0.025331287	0.105202523	-0.240785928	0.811434088	0.927469913
TCL1A	UCL	0.163943541	0.681101709	0.240703465	0.811696247	0.927469913
TMPRSS11B	UCL	0.044469696	0.185213369	0.240099818	0.811720316	0.927469913
MMP8	NIH	0.106793333	0.444819334	0.24008249	0.812015325	0.915584247
MLN	NIH	0.089593333	0.373571325	0.239829257	0.812209703	0.915584247
PRKAG3	UCL	-0.028001843	0.11712186	-0.239082982	0.812337513	0.927469913
TLR2	UCL	0.030183974	0.1265978	0.238424162	0.812422446	0.927469913
RLN1	UCL	-0.045385199	0.189538254	-0.239451394	0.812607064	0.927469913
ANGPTL7	UCL	0.043266407	0.181405664	0.238506375	0.813250087	0.927841532
ALCAM	NIH	-0.0244	0.102322063	-0.238462745	0.813258829	0.915584247
KLF4	NIH	0.050973333	0.213870601	0.238337261	0.813355185	0.915584247
MINDY1	NIH	-0.112013333	0.469987885	-0.238332385	0.81335893	0.915584247
PPM1A	NIH	-0.0288	0.120906927	-0.238199751	0.81346078	0.915584247
AGER	NIH	-0.044753333	0.187993382	-0.238058026	0.813569615	0.915584247

SMC3	UCL	0.033566142	0.14194781	0.236468189	0.813932209	0.928257451
IL34	NIH	-0.081133333	0.341547956	-0.237545949	0.813962887	0.915584247
PDE4D	NIH	0.08136	0.342794369	0.237343455	0.814118415	0.915584247
PYDC1	NIH	0.05002	0.210805967	0.237279811	0.814167299	0.915584247
PKD2	NIH	0.0747	0.315179891	0.237007506	0.814376462	0.915584247
ITPR1	NIH	0.045206667	0.190951095	0.236744736	0.814578313	0.915584247
MPIG6B	NIH	-0.06342	0.268371059	-0.236314602	0.814908757	0.915584247
ICAM2	UCL	0.03120457	0.132050748	0.236307412	0.814965511	0.929073396
ADGRB3	NIH	0.0421	0.17826104	0.236170506	0.815019464	0.915584247
HTR1B	NIH	0.05542	0.23477131	0.236059508	0.815104745	0.915584247
BTD	UCL	0.014361095	0.061268266	0.234396957	0.816216289	0.930136535
ATP2B4	NIH	0.080026667	0.341130081	0.234592817	0.816231838	0.91649832
OMP	UCL	0.036466623	0.156739394	0.232657678	0.817559111	0.93102581
PTGDS	NIH	0.023566667	0.101232821	0.232796701	0.817612625	0.917696442
IL6ST	NIH	-0.015973333	0.068741033	-0.232369702	0.817940974	0.91771283
PLAU	UCL	0.023314212	0.100403447	0.232205292	0.818109838	0.93102581
SERPINB8	UCL	0.046965991	0.20242075	0.232021621	0.81822895	0.93102581
TFPI2	UCL	-0.032979904	0.142164985	-0.231983313	0.818270717	0.93102581
CACNA1H	NIH	0.0552	0.238574141	0.231374615	0.818706296	0.918219293
AGR2	NIH	-0.074273333	0.321610617	-0.230941795	0.819039234	0.918240613
FCN1	UCL	-0.072176605	0.312785678	-0.230754187	0.819227522	0.931751769
ARTN	NIH	0.0307	0.133550317	0.229875906	0.819859297	0.918807833
MCTS1	UCL	-0.026083334	0.113697076	-0.229410774	0.820028271	0.932299741
P4HB	NIH	0.031126667	0.135668606	0.22943161	0.820201186	0.918838939
FOSB	UCL	0.035822124	0.156950621	0.228238179	0.820823633	0.932734402
TPRKB	UCL	0.048832479	0.213986369	0.22820369	0.821048791	0.932734402
AKT3	NIH	-0.0435	0.190986	-0.227765386	0.821483678	0.919923338
NOS3	NIH	-0.05306	0.233712853	-0.227030732	0.822049302	0.920204442
SELE	UCL	0.047968276	0.21150524	0.226794742	0.822383554	0.93334151
PHLDB2	UCL	0.078957195	0.349039197	0.226212973	0.822388561	0.93334151
GC	NIH	-0.025866667	0.11420529	-0.226492719	0.822463589	0.92031599
NHLRC3	UCL	0.026733352	0.118056868	0.226444697	0.822541132	0.93334151
WDR46	UCL	0.030877181	0.137361719	0.224787383	0.823246845	0.933437534

RALY	UCL	0.026412667	0.117613967	0.22457084	0.823283818	0.933437534
SCT	NIH	0.05236	0.232621546	0.225086631	0.823546569	0.920837048
UNC5D	NIH	0.035046667	0.155714067	0.225070653	0.823558878	0.920837048
GUCY2C	UCL	0.094363784	0.419220759	0.225093299	0.823583783	0.933437534
CLEC3B	NIH	0.020213333	0.089981733	0.224638186	0.82389204	0.920857553
CCAR2	UCL	0.034120226	0.152434985	0.223834612	0.8239894	0.933535278
CFC1	UCL	-0.079153407	0.355347124	-0.222749536	0.825308755	0.93466776
SCARB1	NIH	0.085733333	0.38569159	0.222284684	0.825705705	0.922532157
GASK1A	UCL	-0.031706543	0.143039815	-0.221662362	0.826196857	0.935174856
DNPEP	UCL	-0.056458844	0.25478833	-0.221591168	0.826396391	0.935174856
CD163	NIH	0.03638	0.164591662	0.221031853	0.826671568	0.923258624
CIRBP	NIH	0.080546667	0.365419066	0.220422726	0.82714127	0.923341429
STX6	UCL	-0.126307059	0.573275321	-0.220325304	0.827234605	0.935707779
FDX1	UCL	0.054389535	0.24771139	0.219568165	0.827517089	0.935707779
CCDC80	NIH	0.045286667	0.20602686	0.219809527	0.827614179	0.923341429
RNF43	NIH	-0.040846667	0.18591369	-0.219707686	0.827692727	0.923341429
PTPRC	UCL	0.025915816	0.118063113	0.219508156	0.827827683	0.935707779
CSF3R	UCL	0.03623575	0.165396914	0.219083593	0.828288058	0.935866251
AOC3	NIH	0.042773333	0.195408925	0.218891401	0.828322376	0.923691555
TGFB2	UCL	-0.036611646	0.168424752	-0.217376871	0.828796678	0.936079092
SEPTIN3	UCL	-0.069934059	0.320923741	-0.217914882	0.829154857	0.936121919
TNFRSF19	NIH	0.031486667	0.144675725	0.217636143	0.829290857	0.924419115
FABP4	UCL	-0.044766105	0.206292816	-0.217002735	0.829719854	0.936209782
FUT1	UCL	0.047639843	0.22006534	0.216480444	0.829873262	0.936209782
BTN2A1	UCL	-0.021714817	0.10053428	-0.215994156	0.830641008	0.93640699
CEMIP2	UCL	-0.034575971	0.160592189	-0.215302943	0.830716239	0.93640699
OLFM4	UCL	0.082393935	0.382972437	0.21514325	0.831009145	0.93640699
FGL1	UCL	0.074044259	0.34498613	0.214629669	0.831741402	0.936870952
ENSA	NIH	-0.04746	0.222145887	-0.213643388	0.832373237	0.926826896
ARL13B	NIH	0.06262	0.293143651	0.213615406	0.832394849	0.926826896
SEMA4D	NIH	0.019593333	0.091726146	0.213606852	0.832401455	0.926826896
COCH	UCL	-0.04527717	0.212051743	-0.213519443	0.832523061	0.937035689
TNFRSF4	UCL	-0.028169151	0.131911404	-0.213545983	0.8325288	0.937035689

MTR	UCL	0.070121889	0.331410527	0.211586185	0.834060332	0.938159212
GRN	UCL	0.021079475	0.099724402	0.211377305	0.834168934	0.938159212
TMED10	NIH	-0.028586667	0.135342258	-0.211217598	0.834247262	0.927406306
IGF2BP3	NIH	0.03166	0.14998889	0.211082301	0.834351815	0.927406306
LILRB5	NIH	0.0662	0.313762011	0.210987939	0.834424736	0.927406306
PDP1	NIH	0.031753333	0.150536104	0.210935002	0.834465645	0.927406306
BAG6	NIH	0.029546667	0.140110415	0.210881302	0.834507144	0.927406306
ATP1B1	UCL	0.069149171	0.32975123	0.209701025	0.835394773	0.939176508
MARCO	NIH	0.02616	0.124793947	0.209625552	0.835477726	0.928132301
FKBP1B	NIH	-0.085166667	0.408197033	-0.208641072	0.836238825	0.928625119
PKD1	UCL	0.028611783	0.137404977	0.208229599	0.836599104	0.940108615
ENTPD5	NIH	-0.01944	0.093519308	-0.207871512	0.836833883	0.92870775
CLEC1A	NIH	0.0322	0.155013665	0.207723622	0.836948249	0.92870775
IGSF3	UCL	0.039617202	0.190883399	0.207546609	0.837153257	0.940108615
S100A16	UCL	0.055646949	0.269542938	0.206449292	0.837188753	0.940108615
PDE1C	NIH	0.045033333	0.217542943	0.207008936	0.837500981	0.928721403
ATF2	NIH	0.09342	0.451551643	0.206886635	0.837595577	0.928721403
CCL8	UCL	0.055550894	0.269130183	0.206409007	0.838027652	0.940689257
SAP18	NIH	-0.048533333	0.235382583	-0.206189144	0.838135106	0.928967482
VWF	UCL	-0.044212268	0.214857351	-0.205774986	0.838565381	0.94093152
SNED1	UCL	-0.032820528	0.160513093	-0.204472589	0.839172768	0.941251727
KIR2DS4	NIH	0.148873333	0.729354893	0.204116452	0.839738866	0.929721527
BGLAP	NIH	0.0765	0.374965204	0.204018931	0.839814341	0.929721527
MSLNL	UCL	0.050074254	0.245555781	0.203922113	0.839923686	0.941503278
IGSF8	UCL	-0.02232569	0.109481456	-0.203922115	0.840041241	0.941503278
TACSTD2	NIH	-0.03108	0.152622382	-0.203639857	0.840107735	0.929721527
CEP20	NIH	0.05644	0.277584148	0.203325732	0.840350877	0.929721527
TLR3	NIH	-0.050233333	0.247238157	-0.203177915	0.840465297	0.929721527
RAP1A	UCL	0.096434399	0.474569867	0.203203797	0.840632072	0.9415485
MSMB	UCL	-0.068797351	0.340337425	-0.202144537	0.841282616	0.9415485
TFAP2A	NIH	-0.14716	0.729018816	-0.201860359	0.841485332	0.929721527
NADK	UCL	-0.039104115	0.193787115	-0.201789035	0.841487108	0.9415485
CCDC50	NIH	0.043893333	0.217515164	0.20179436	0.841536435	0.929721527

CDA	UCL	-0.042706923	0.211696311	-0.201736737	0.84164788	0.9415485
GPC5	UCL	-0.047675996	0.236401787	-0.201673583	0.841692176	0.9415485
LILRA3	NIH	0.045313333	0.225120287	0.201284984	0.841930868	0.929721527
RTN4R	NIH	0.033886667	0.168829833	0.200714922	0.842372343	0.929721527
FABP6	UCL	0.027203327	0.136232002	0.19968382	0.842451201	0.942037054
FCER1A	NIH	-0.024533333	0.12231777	-0.200570475	0.842484215	0.929721527
SAMD9L	NIH	0.05126	0.255788181	0.200400189	0.842616104	0.929721527
CBLIF	NIH	0.063666667	0.317725395	0.200382682	0.842629664	0.929721527
SIGLEC7	UCL	0.027331061	0.136865929	0.199692216	0.843134038	0.942440074
C1QTNF9	NIH	0.053793333	0.269928027	0.199287691	0.843477865	0.930306469
TK1	UCL	0.026549877	0.134300038	0.197690767	0.844624303	0.943744969
GAD2	UCL	-0.038087637	0.193649239	-0.196683639	0.845103512	0.94391959
DCBLD2	NIH	-0.029033333	0.147588724	-0.196717829	0.845469281	0.932151393
RPS10	UCL	-0.05732811	0.292390995	-0.196066605	0.846164936	0.94420808
ORM1	UCL	-0.017594239	0.089817386	-0.195889015	0.846204855	0.94420808
PNMA2	UCL	-0.029169632	0.149732818	-0.194811212	0.846330883	0.94420808
DPP4	NIH	0.0166	0.085025722	0.195235038	0.846618787	0.932704704
PLCB1	NIH	-0.025453333	0.130443894	-0.195128591	0.846701321	0.932704704
REG3G	NIH	-0.038546667	0.197841036	-0.194836558	0.846927759	0.932704704
CRHR1	UCL	-0.04847044	0.249501609	-0.194269049	0.847198829	0.944632876
SLC34A3	NIH	-0.03138	0.161484741	-0.194321765	0.847326955	0.932793129
GATD3	UCL	-0.042802461	0.22069332	-0.193945432	0.847440212	0.944632876
OGT	UCL	0.06071794	0.313369573	0.19375825	0.847864564	0.944632876
SRPX	UCL	0.03638383	0.187982792	0.193548727	0.848004334	0.944632876
POMC	NIH	0.06116	0.317754008	0.192475935	0.848758644	0.933497941
HEPACAM2	NIH	0.034173333	0.177742449	0.192263207	0.848923677	0.933497941
CTSS	NIH	0.014426667	0.0750365	0.192261988	0.848924623	0.933497941
SERPINF1	UCL	0.020364221	0.106307069	0.191560367	0.849398473	0.945825423
ACE	NIH	0.027473333	0.143786483	0.191070348	0.849849218	0.934163458
PRCP	UCL	0.032729275	0.171866123	0.190434711	0.850482953	0.94635893
PXDNL	UCL	0.069777206	0.367498659	0.189870642	0.850794442	0.94635893
ASAH2	UCL	-0.032938775	0.17360906	-0.189729586	0.850889076	0.94635893
NECTIN4	UCL	-0.018726085	0.098923054	-0.189299501	0.851256736	0.94635893

ROR1	NIH	0.02962	0.156705783	0.189016636	0.851443207	0.935564004
SDC4	UCL	0.082053219	0.435297169	0.188499318	0.851742649	0.94635893
AMPD3	UCL	-0.040728982	0.216789412	-0.187873485	0.852154947	0.94635893
ADAMTS15	UCL	0.034365231	0.182747114	0.188048007	0.852305077	0.94635893
KLK10	UCL	0.02474847	0.131854097	0.187695875	0.852467692	0.94635893
DSCAM	NIH	0.035006667	0.18677537	0.187426569	0.852677778	0.936568719
IL32	NIH	0.03488	0.187063958	0.186460291	0.853428208	0.937041107
GIMAP7	UCL	0.019822112	0.106499543	0.186123919	0.853492175	0.947136533
CREBZF	NIH	-0.02026	0.109253215	-0.185440767	0.854220142	0.937558692
TNFRSF8	UCL	0.03120048	0.168386172	0.185291224	0.854413531	0.947330818
ADAMTS1	UCL	-0.026040649	0.141259462	-0.18434623	0.854651531	0.947330818
VWC2L	UCL	-0.020922329	0.113406958	-0.184488935	0.85491266	0.947330818
CD74	UCL	0.028031994	0.151945454	0.184487218	0.855024651	0.947330818
OMP	NIH	0.032026667	0.173692434	0.18438723	0.855038659	0.937954539
VSIR	NIH	0.046073333	0.250753622	0.183739453	0.855542013	0.937954539
CXCL16	NIH	0.01798	0.097867574	0.183717643	0.855558961	0.937954539
DLL1	UCL	0.022902733	0.12463021	0.183765505	0.855610222	0.947330818
TSC22D1	UCL	0.038919513	0.212213304	0.183398084	0.855683014	0.947330818
CRISP3	NIH	-0.02462	0.134445958	-0.183121906	0.856021935	0.937954539
ITGB7	UCL	0.054585056	0.298025014	0.183155955	0.856109349	0.947330818
RAC3	NIH	0.03782	0.207015262	0.182691845	0.856356187	0.937954539
CRNN	UCL	0.041551107	0.227377073	0.182740971	0.856357679	0.947330818
SLC13A1	NIH	0.04692	0.257095004	0.18250063	0.856504811	0.937954539
MYOC	UCL	0.037348444	0.204770581	0.182391652	0.85658411	0.947330818
FOXO3	UCL	0.130131029	0.724108336	0.179712099	0.858544396	0.949139664
SORT1	UCL	-0.022567342	0.127065843	-0.177603528	0.860375249	0.950506383
TP53	UCL	-0.066601001	0.375727179	-0.177258938	0.860431027	0.950506383
DOK2	NIH	0.058546667	0.330438762	0.177178568	0.860643597	0.941818391
CEACAM21	NIH	-0.073206667	0.413576468	-0.177008782	0.860775702	0.941818391
DHPS	NIH	0.026833333	0.152135812	0.176377495	0.861266918	0.941818391
SEPTIN9	NIH	0.016846667	0.095552547	0.176307876	0.861321092	0.941818391
BMP6	UCL	-0.027863808	0.158456655	-0.175844982	0.861740639	0.951593459
SLAMF8	UCL	-0.055485947	0.318134546	-0.174410316	0.862843612	0.952173138

CAPS	UCL	0.025535871	0.147129619	0.17356037	0.863465655	0.952173138
MAMDC2	NIH	0.027606667	0.159315787	0.173282681	0.863675863	0.943475406
XRCC4	UCL	-0.024460829	0.141809221	-0.172491103	0.863832823	0.952173138
HDAC8	UCL	0.055139231	0.318631909	0.173049934	0.86383749	0.952173138
CA6	UCL	-0.041750422	0.241205169	-0.17309091	0.863894342	0.952173138
SKAP2	NIH	0.061446667	0.355212744	0.172985535	0.863907227	0.943475406
GRK5	NIH	-0.021113333	0.122071819	-0.172958293	0.863928439	0.943475406
EFCAB2	NIH	-0.023286667	0.134941386	-0.172568753	0.864231763	0.943475406
SYTL4	NIH	-0.044946667	0.26087881	-0.17228945	0.864449261	0.943475406
CDHR1	UCL	0.031604026	0.183724683	0.172018399	0.864541586	0.952404659
SFTPD	UCL	0.038510181	0.223964197	0.171947934	0.86475606	0.952404659
ENTPD6	UCL	0.018337955	0.10726182	0.170964418	0.86547569	0.952838208
ENAH	UCL	-0.02607233	0.152885223	-0.170535315	0.865822448	0.952861075
TK1	NIH	0.029633333	0.174083277	0.170225043	0.866057186	0.944877758
FOS	NIH	-0.05042	0.297810585	-0.169302243	0.866776126	0.945068832
IGFBP2	UCL	0.04612799	0.272989469	0.168973516	0.867055356	0.953291487
LAMA4	UCL	0.020167631	0.119467071	0.168813303	0.86716388	0.953291487
LPO	NIH	-0.037466667	0.22226308	-0.168569007	0.867347462	0.945068832
PAPPA	NIH	0.049706667	0.295766526	0.168060488	0.867743742	0.945068832
AKR1C4	UCL	0.029365929	0.175018068	0.167787987	0.867776803	0.953291487
CLC	UCL	-0.043412878	0.258350716	-0.168038545	0.867798056	0.953291487
ZP3	NIH	-0.15462	0.921979119	-0.167704449	0.868021218	0.945068832
NELL1	NIH	-0.02648	0.158465294	-0.167102836	0.868490119	0.945068832
CREB3	UCL	-0.033265813	0.199419046	-0.166813622	0.868504969	0.953291487
BABAM1	NIH	0.01596	0.095585203	0.166971451	0.868592527	0.945068832
SLAMF6	UCL	0.036251392	0.217225659	0.166883564	0.868614157	0.953291487
CCL27	NIH	-0.038626667	0.231483004	-0.166866103	0.868674643	0.945068832
PDGFB	UCL	-0.088433842	0.531202838	-0.166478481	0.868818617	0.953291487
SEMA3G	UCL	-0.028496579	0.171292265	-0.166362321	0.86910191	0.953291487
CD274	UCL	0.024569804	0.147736812	0.166307933	0.869148755	0.953291487
SUSD1	NIH	-0.044886667	0.270444918	-0.165973415	0.869370528	0.945068832
LY96	NIH	0.03174	0.191528648	0.165719334	0.869568613	0.945068832
NPHS2	UCL	0.080183721	0.484420812	0.165524929	0.869771245	0.95361641

PTPN1	NIH	0.05458	0.33042293	0.165182241	0.869987367	0.945068832
WFDC12	NIH	-0.043566667	0.263942861	-0.165060978	0.870081918	0.945068832
LEFTY2	NIH	-0.03672	0.222738646	-0.164856888	0.870241053	0.945068832
ADAMTS4	UCL	0.028029984	0.170900413	0.164013553	0.870501294	0.954058974
COMP	NIH	0.025406667	0.154990813	0.163923695	0.870968766	0.945068832
NFATC3	UCL	0.039606131	0.241684595	0.16387528	0.871007476	0.954255941
HPCAL1	NIH	0.01686	0.103294148	0.163223186	0.871515105	0.945068832
DAPP1	NIH	-0.048366667	0.296987606	-0.162857526	0.871800316	0.945068832
GUCA2A	UCL	-0.025059448	0.154189896	-0.162523281	0.872051497	0.955041785
TEF	NIH	0.024693333	0.152098195	0.162351258	0.872195227	0.945068832
RBP2	NIH	0.041873333	0.258793696	0.161801983	0.872623724	0.945068832
EXOSC10	UCL	-0.021492635	0.133081726	-0.161499523	0.872707432	0.955402181
STC1	NIH	-0.040906667	0.253470162	-0.161386517	0.872947861	0.945068832
VAMP8	NIH	0.049833333	0.309143556	0.161198034	0.873094919	0.945068832
PTGR1	UCL	0.053253369	0.330988927	0.160891693	0.87330233	0.955695512
NTF3	NIH	0.022873333	0.142228994	0.160820467	0.873389516	0.945068832
FGF23	NIH	-0.02908	0.181142666	-0.160536447	0.873611137	0.945068832
MSLNL	NIH	0.027406667	0.170769368	0.160489361	0.873647879	0.945068832
PPP1R12B	NIH	0.050946667	0.317485694	0.160469173	0.873663632	0.945068832
CDH6	UCL	0.024627701	0.153873073	0.160052051	0.874019736	0.956122638
GM2A	UCL	0.029388204	0.185285243	0.158610601	0.874685108	0.956267068
SERPINE2	UCL	-0.060821422	0.382823345	-0.158875948	0.874806069	0.956267068
TFRC	NIH	-0.019533333	0.123012008	-0.158792086	0.874972483	0.945973008
MBL2	NIH	-0.05954	0.37548231	-0.158569388	0.875146311	0.945973008
HMOX1	NIH	-0.026713333	0.169145777	-0.157930832	0.875644774	0.946108925
PON3	NIH	-0.01912	0.121335497	-0.157579608	0.875918964	0.946108925
SFTPA2	UCL	0.047661594	0.303036709	0.157279934	0.876195746	0.957339752
SAP18	UCL	0.102217087	0.65395953	0.156304912	0.876981982	0.957339752
ITGAL	NIH	-0.10526	0.675572497	-0.155808593	0.877301785	0.946868886
REG4	NIH	-0.03644	0.234775664	-0.155211998	0.877767699	0.946868886
DLL4	UCL	0.047226547	0.30488069	0.154901734	0.877930099	0.957339752
PDGFA	UCL	-0.072965734	0.471296413	-0.1548192	0.877958016	0.957339752
NOS2	UCL	0.038389036	0.248641571	0.154395084	0.878005332	0.957339752

LRIG3	UCL	0.017453554	0.112991869	0.154467343	0.878081777	0.957339752
GKN1	NIH	-0.02816	0.182128966	-0.154615713	0.878233416	0.946868886
FUT8	UCL	-0.036534391	0.236609737	-0.15440781	0.878340122	0.957339752
ECHDC3	NIH	0.060966667	0.395454757	0.1541685	0.878582733	0.946868886
SIGLEC1	NIH	0.033126667	0.215241601	0.153904573	0.878788897	0.946868886
TBC1D23	NIH	-0.06144	0.399244454	-0.153890679	0.878799751	0.946868886
TNFRSF14	NIH	0.019133333	0.124716996	0.153414	0.879172126	0.946868886
PROCR	UCL	0.02716048	0.17744859	0.153061119	0.879197254	0.957339752
IL19	UCL	0.052004183	0.339209328	0.153310003	0.879311968	0.957339752
FSTL3	UCL	-0.024686122	0.161274197	-0.15306926	0.879524125	0.957339752
KIF20B	UCL	-0.03503942	0.229500463	-0.152676903	0.879632787	0.957339752
CDKN2D	NIH	-0.065566667	0.42969162	-0.152590052	0.879815852	0.946868886
SCIN	UCL	-0.054082408	0.355244304	-0.152240042	0.879823614	0.957339752
ACE2	NIH	-0.030426667	0.199604326	-0.152434906	0.879937072	0.946868886
MINK1	NIH	0.05504	0.361171243	0.152393085	0.879969748	0.946868886
SBSN	UCL	0.023084427	0.152345722	0.151526583	0.880104904	0.957339752
HPSE	NIH	0.034606667	0.227496398	0.152119625	0.880183419	0.946868886
DDX25	UCL	0.018573943	0.122335522	0.151827882	0.880372649	0.957339752
FRZB	UCL	-0.017179721	0.114045698	-0.150638926	0.88133583	0.958030729
LYPD8	NIH	-0.04594	0.305421426	-0.150415118	0.881515461	0.947711963
DIPK1C	NIH	0.04046	0.269941627	0.149884256	0.881930392	0.947711963
FABP6	NIH	-0.04438	0.296117233	-0.149873074	0.881939133	0.947711963
TRIAP1	UCL	0.033989483	0.227322556	0.149520945	0.882158931	0.958372738
ODAM	UCL	-0.03486986	0.233492552	-0.149340352	0.882306205	0.958372738
SSH3	NIH	0.020713333	0.138953077	0.149067108	0.882569157	0.948040685
RNASE4	UCL	-0.01881265	0.126394998	-0.148840147	0.882748562	0.958497045
KLK10	NIH	0.023146667	0.156842393	0.147579147	0.883732503	0.94894184
INHBB	UCL	0.034151316	0.231765482	0.147352901	0.883860783	0.959348336
TCP11	UCL	-0.049791829	0.339078652	-0.146844482	0.884319283	0.959489705
IL7	NIH	0.02882	0.19768259	0.145789268	0.885132251	0.949771108
TMED4	NIH	0.042073333	0.288645624	0.145761203	0.885154202	0.949771108
VEGFA	UCL	-0.034603159	0.239508153	-0.144475912	0.886146964	0.960628658
KYNU	UCL	0.030700718	0.212398517	0.144542997	0.886147745	0.960628658

LYPD3	UCL	0.023649157	0.163932376	0.144261664	0.88635494	0.960628658
NCS1	NIH	0.021113333	0.147560817	0.143082247	0.887249952	0.95167074
ADGRE1	UCL	-0.044549732	0.311495189	-0.143019004	0.887342324	0.961084893
ANXA10	UCL	-0.053040473	0.372861987	-0.142252293	0.887764119	0.961084893
CPOX	NIH	-0.017326667	0.121967125	-0.142060138	0.888049769	0.952179462
CAPG	UCL	-0.039575731	0.278661297	-0.14202091	0.888143836	0.961084893
CRTAP	UCL	0.012557078	0.088966286	0.141144233	0.888262514	0.961084893
VSIG4	UCL	0.027099548	0.191591578	0.141444361	0.888640886	0.961084893
SHMT1	UCL	0.050888556	0.361606492	0.1407291	0.889006265	0.961084893
FLT3	UCL	0.020031908	0.142618374	0.140458111	0.889305192	0.961084893
PSMC3	UCL	-0.01845518	0.131885341	-0.139933518	0.889406307	0.961084893
VCPKMT	UCL	0.045131214	0.322818493	0.139803683	0.889841366	0.961199672
PTX3	UCL	-0.022373036	0.161285337	-0.13871711	0.890171338	0.961200895
CLEC6A	UCL	-0.040322574	0.292019982	-0.138081557	0.891225193	0.961677848
PDCL2	UCL	0.025584859	0.18578424	0.13771275	0.891326159	0.961677848
ARHGAP30	NIH	0.016453333	0.119523382	0.137657863	0.891495984	0.955183713
SERPINI1	NIH	0.01348	0.097931946	0.137646607	0.891504799	0.955183713
GPKOW	UCL	-0.012037996	0.087892037	-0.136963443	0.891839377	0.961677848
EPHA10	UCL	0.082793702	0.604161378	0.137039051	0.891995764	0.961677848
DCN	NIH	0.011006667	0.080411369	0.136879483	0.892105547	0.955388312
THSD1	NIH	0.0275	0.201363986	0.136568612	0.892349014	0.955388312
SIRT5	UCL	0.016344261	0.120005179	0.136196297	0.892440585	0.961677848
DCDC2C	UCL	-0.055032371	0.403993499	-0.136220931	0.892587069	0.961677848
LRRC25	UCL	-0.025967832	0.191873217	-0.135338495	0.893350387	0.962145609
CRIP2	NIH	-0.01664	0.12301587	-0.1352671	0.893368446	0.956129786
DDX4	UCL	0.039484465	0.292983588	0.134766815	0.893876711	0.962357874
ARSA	NIH	-0.02154	0.160329527	-0.134348304	0.89408822	0.956542139
CMC1	NIH	-0.017173333	0.128499417	-0.133645224	0.894639068	0.956542139
NEDD9	NIH	0.02406	0.18019362	0.13352304	0.894734801	0.956542139
CD28	UCL	0.022115724	0.166002831	0.133224979	0.894754613	0.962948356
TPK1	NIH	0.01048	0.078993787	0.132668662	0.89540427	0.956566888
C1QTNF5	NIH	-0.017026667	0.128481027	-0.13252281	0.895518565	0.956566888
MANSC1	NIH	-0.014933333	0.112924747	-0.132241459	0.895739045	0.956566888

CMIP	NIH	0.03866	0.293620027	0.131666768	0.896189429	0.956698569
CTSF	UCL	0.020619673	0.156704291	0.131583333	0.896276449	0.96423116
SMTN	NIH	0.063193333	0.483156652	0.130792639	0.89687455	0.957059803
BEX3	NIH	-0.03424	0.262576428	-0.130400129	0.897182216	0.957059803
RAB33A	NIH	-0.045866667	0.354799199	-0.129275001	0.898064229	0.957371216
ANGPT2	UCL	0.021495003	0.166524318	0.129080263	0.898264459	0.965773957
FAM13A	NIH	-0.0287	0.223608112	-0.128349547	0.898789812	0.957371216
NPTXR	UCL	-0.018435324	0.144109736	-0.127925599	0.899173486	0.965773957
IL36A	NIH	0.037013333	0.289803412	0.127718763	0.899284417	0.957371216
PTPRC	NIH	0.011766667	0.092176962	0.12765301	0.899335977	0.957371216
LILRB2	UCL	0.019756888	0.154797761	0.127630319	0.899397938	0.965773957
TNFSF10	NIH	-0.013053333	0.102416959	-0.127452851	0.899492934	0.957371216
WASF3	NIH	0.126386667	0.991977812	0.127408764	0.899527506	0.957371216
C7orf50	UCL	-0.006836814	0.053771432	-0.127145847	0.899540752	0.965773957
FABP2	UCL	0.046772405	0.367683866	0.127208207	0.899703831	0.965773957
VEGFB	UCL	-0.017096042	0.134688682	-0.126930054	0.899757482	0.965773957
NPPC	NIH	0.021613333	0.170377838	0.126855309	0.899961527	0.957371216
DSG2	UCL	-0.016332081	0.129206103	-0.126403325	0.900351967	0.965773957
SLITRK6	NIH	0.019513333	0.15469449	0.126141101	0.900521659	0.957371216
ADRA2A	UCL	0.030202331	0.239360818	0.126179095	0.900542944	0.965773957
BCL2	UCL	-0.013099945	0.104092239	-0.125849395	0.900684162	0.965773957
AMOTL2	NIH	0.025813333	0.205273387	0.125750998	0.900827627	0.957371216
COL5A1	NIH	0.0244	0.19422885	0.125625003	0.900926451	0.957371216
FGFBP3	NIH	0.01724	0.137709698	0.125190892	0.90126696	0.957371216
STXBP1	NIH	0.02468	0.197410049	0.125018965	0.901401822	0.957371216
VSIG10L	NIH	-0.018633333	0.149562077	-0.124585949	0.901741498	0.957384349
CD207	UCL	-0.03091229	0.248699424	-0.124295786	0.902031604	0.966864092
TNFRSF13C	NIH	0.025773333	0.208354818	0.123699244	0.902437127	0.957775252
IFNAR1	UCL	-0.014040017	0.113928336	-0.123235517	0.902825475	0.967205574
AGRN	UCL	-0.017765201	0.14440121	-0.123026675	0.903011978	0.967205574
F3	NIH	0.01398	0.114106557	0.122517061	0.903364683	0.957899052
FCAR	UCL	-0.028740881	0.235520692	-0.122031235	0.903796538	0.967332935
SCARF1	NIH	-0.0176	0.144304446	-0.121964364	0.903798384	0.957899052

COL15A1	NIH	0.010006667	0.082093615	0.121893361	0.903854102	0.957899052
BPIFB2	UCL	-0.055295056	0.453610011	-0.12189999	0.903904229	0.967332935
WFDC2	NIH	0.018666667	0.153392285	0.121692344	0.904011849	0.957899052
IL12RB1	UCL	0.019081708	0.1573105	0.121299648	0.904123701	0.967332935
SLAMF6	NIH	0.027586667	0.227577522	0.121218767	0.904383501	0.957899052
CA7	NIH	0.023286667	0.192377853	0.121046504	0.904518695	0.957899052
DOC2B	UCL	-0.021870809	0.182035182	-0.120146056	0.905077495	0.967999092
RSPO3	NIH	0.015533333	0.129828993	0.119644564	0.905619059	0.958717244
CD6	UCL	0.028372459	0.238029549	0.119197213	0.906021403	0.968488285
GZMB	UCL	-0.029699791	0.250343862	-0.118635987	0.906197557	0.968488285
SUOX	UCL	0.03164054	0.267767024	0.118164438	0.906786444	0.968763442
ASAH1	UCL	0.01444091	0.123787323	0.116659037	0.907754945	0.969170874
EPHB6	UCL	-0.013917773	0.119093215	-0.116864537	0.907830945	0.969170874
DIABLO	NIH	-0.037766667	0.324565868	-0.116360561	0.908197372	0.960727559
PLSCR3	NIH	0.029566667	0.254718615	0.116075799	0.908420991	0.960727559
KIAA2013	NIH	0.0243	0.209535382	0.115970867	0.908503394	0.960727559
SLITRK6	UCL	-0.020581653	0.177985111	-0.115636937	0.908781557	0.969831505
SERPINA1	NIH	-0.00246	0.021537896	-0.114217285	0.909880638	0.961836236
IL34	UCL	0.03279397	0.287462082	0.114081029	0.909970928	0.970746359
TIMP2	NIH	-0.0255	0.224640587	-0.11351466	0.910432552	0.961971296
CDK1	NIH	0.033146667	0.29300744	0.113125683	0.910738115	0.961971296
LAMTOR5	UCL	0.038674368	0.342481576	0.112923937	0.91092882	0.971413696
CD86	NIH	0.00828	0.073492644	0.112664337	0.911100544	0.961971296
LEPR	NIH	-0.014246667	0.126772248	-0.112380011	0.911323918	0.961971296
DKK1	UCL	-0.033670638	0.300370168	-0.112097144	0.911487926	0.971498767
CD300E	UCL	0.021514305	0.192942694	0.111506192	0.912043252	0.971498767
SMNDC1	UCL	0.024494158	0.220832064	0.110917581	0.912332212	0.971498767
SCGB1A1	UCL	-0.022389359	0.201501732	-0.111112491	0.912338049	0.971498767
PTPRS	NIH	-0.016033333	0.144554803	-0.110915259	0.912474784	0.962838652
MED21	NIH	0.01296	0.117325464	0.110461954	0.912830988	0.962867162
APOA1	NIH	0.006213333	0.056694813	0.109592623	0.913514154	0.963058814
BAP18	UCL	-0.031533847	0.28882341	-0.109180371	0.913747684	0.972645477
RAB27B	NIH	-0.03974	0.363666556	-0.109275927	0.913763047	0.963058814

DDX58	NIH	0.030173333	0.278055438	0.10851553	0.914360684	0.963058814
JMJD1C	NIH	-0.033886667	0.313088314	-0.108233572	0.914582302	0.963058814
CEACAM6	NIH	0.01784	0.164977319	0.108136077	0.914658936	0.963058814
SATB1	UCL	-0.008926394	0.083329308	-0.107121898	0.915180279	0.973194321
AK1	UCL	-0.020081112	0.189876831	-0.105758621	0.916264364	0.973194321
FCER2	NIH	0.034646667	0.326920482	0.10597888	0.91635474	0.964175266
CKB	UCL	0.026659725	0.252153283	0.10572825	0.916539995	0.973194321
TOR1AIP1	NIH	0.036726667	0.347461184	0.105700056	0.916573957	0.964175266
KIRREL1	UCL	0.024999059	0.237384112	0.105310581	0.916651897	0.973194321
CDAN1	UCL	0.011301864	0.10781267	0.104828721	0.916975363	0.973194321
CA5A	UCL	0.040609804	0.386062611	0.105189685	0.917049973	0.973194321
MNAT1	NIH	-0.014393333	0.1370455	-0.105025946	0.917103984	0.964175266
FAM171B	NIH	-0.009966667	0.095115929	-0.104784411	0.917293903	0.964175266
BAX	NIH	-0.03302	0.315404715	-0.104690889	0.917367441	0.964175266
LYSMD3	NIH	0.039426667	0.378187812	0.104251553	0.917712907	0.964191902
TFRC	UCL	0.011390758	0.109873227	0.103671829	0.918180527	0.973194321
IL5RA	NIH	-0.021586667	0.208524909	-0.103520806	0.918287557	0.964273643
TXNL1	NIH	-0.029493333	0.285765818	-0.103208052	0.918533517	0.964273643
LIPF	UCL	0.021143025	0.204876866	0.103198693	0.918541929	0.973194321
DEFB116	UCL	0.012324891	0.11990938	0.102785041	0.918699511	0.973194321
IFNG	UCL	-0.046893226	0.456602874	-0.102700242	0.918749507	0.973194321
CDH3	UCL	-0.013398881	0.130243357	-0.102875731	0.918791792	0.973194321
EPGN	UCL	0.009421527	0.091743242	0.102694504	0.918851555	0.973194321
RGS10	NIH	0.0191	0.186362754	0.10248829	0.919099591	0.964273643
AMDHD2	UCL	0.010484739	0.10258716	0.102203226	0.919141763	0.973194321
TPT1	NIH	0.09298	0.908658451	0.102326677	0.919226702	0.964273643
PCDH9	UCL	0.016317994	0.15941312	0.102362931	0.919246454	0.973194321
ENOPH1	NIH	-0.027533333	0.269784647	-0.10205671	0.919439039	0.964273643
S100A11	UCL	-0.009953765	0.09864992	-0.100899877	0.919990331	0.973194321
VEGFC	UCL	0.022751152	0.224801742	0.101205407	0.920022165	0.973194321
FGF9	UCL	-0.025650415	0.254488514	-0.100792032	0.920173148	0.973194321
NMRK2	UCL	0.015156342	0.150297942	0.100841977	0.920256279	0.973194321
TSPAN7	NIH	0.031813333	0.316093078	0.10064546	0.920549127	0.965091826

ACAA1	UCL	0.049483437	0.495832348	0.099798727	0.921223461	0.973864802
GDF15	NIH	0.021006667	0.210577798	0.099757272	0.921247859	0.965240061
NOTCH1	NIH	0.00616	0.061830758	0.099626791	0.921350513	0.965240061
APOF	UCL	0.007898343	0.080029334	0.098693093	0.92203339	0.974368619
TMCO5A	NIH	0.028406667	0.290528821	0.097775727	0.922806958	0.965776063
PCDH7	NIH	-0.02106	0.215423004	-0.097761147	0.92281843	0.965776063
SELP	NIH	0.014986667	0.153367089	0.097717618	0.922852683	0.965776063
MAG	UCL	-0.013608083	0.139973664	-0.097218881	0.922974856	0.974745389
SCGB2A2	UCL	0.012312857	0.127901305	0.096268425	0.923651081	0.974745389
ARG1	UCL	0.019874405	0.205839283	0.096553024	0.92369274	0.974745389
CCL15	UCL	0.022352593	0.231335815	0.096624005	0.923738342	0.974745389
CPXM2	UCL	0.012972926	0.134918532	0.096153775	0.924057295	0.974745389
PPP1R14D	NIH	0.023893333	0.249313264	0.095836591	0.924332991	0.966917744
MMP13	NIH	-0.030166667	0.317001783	-0.095162451	0.924863585	0.966917744
RBPMS2	NIH	-0.046886667	0.493173736	-0.095071297	0.924935333	0.966917744
GSTP1	NIH	-0.028673333	0.303571627	-0.094453272	0.925421796	0.967080655
BCL2L15	UCL	0.020949294	0.224140402	0.093465048	0.926066127	0.976505351
SLK	NIH	0.028833333	0.308074346	0.093592127	0.926099674	0.96721416
SUMF2	UCL	-0.018616588	0.199831171	-0.093161583	0.926393889	0.976505351
GNPDA2	NIH	-0.014246667	0.153198291	-0.092994945	0.926569798	0.96721416
SHBG	NIH	0.021533333	0.232116214	0.092769622	0.926747189	0.96721416
ZP4	NIH	-0.011053333	0.119385381	-0.092585317	0.926892289	0.96721416
SPARCL1	UCL	-0.00586206	0.063501542	-0.092313665	0.927096355	0.976581678
BLMH	UCL	-0.01277658	0.138444486	-0.092286667	0.927134504	0.976581678
PNLIP	NIH	0.026606667	0.288604409	0.092190784	0.927202907	0.96721416
PTPN9	UCL	0.018604755	0.204707683	0.0908845	0.928124809	0.977272628
PITHD1	UCL	0.032914627	0.36423318	0.090366911	0.92862842	0.9774508
MAP3K5	NIH	-0.033366667	0.369604205	-0.090276751	0.928710003	0.968440913
GALNT7	NIH	-0.005586667	0.062693432	-0.089110877	0.929628137	0.968804591
FGF3	NIH	0.012206667	0.137164779	0.088992719	0.929721192	0.968804591
FETUB	UCL	0.016478516	0.18653767	0.08833881	0.930250807	0.978462646
IFNGR2	UCL	-0.025155175	0.286741866	-0.087727598	0.930770834	0.978462646
GHR	UCL	0.008592037	0.098042746	0.087635618	0.930778211	0.978462646

BST1	NIH	0.02388	0.272447303	0.087649978	0.930778743	0.969180444
CD5	UCL	-0.011181209	0.127784383	-0.087500594	0.930928709	0.978462646
ARID4B	NIH	0.018926667	0.216651132	0.08736011	0.931007062	0.969180444
LAMA1	NIH	0.037353333	0.428126164	0.087248425	0.931095034	0.969180444
DLG4	NIH	-0.024013333	0.276485511	-0.08685205	0.931407258	0.969180444
NLGN2	UCL	0.022530042	0.262221173	0.085919994	0.932163231	0.978479525
THBS2	UCL	-0.017097824	0.199177084	-0.085842327	0.932254292	0.978479525
GAD1	UCL	-0.011070813	0.130134982	-0.085071769	0.932507752	0.978479525
BLVRB	UCL	-0.011652105	0.137024189	-0.085036844	0.932698094	0.978479525
IL1R1	NIH	0.006633333	0.078099016	0.084934916	0.932917534	0.969879221
KLK4	UCL	-0.016392538	0.19309912	-0.084891831	0.932977968	0.978479525
ANKMY2	NIH	0.016246667	0.191482175	0.084846888	0.932986887	0.969879221
TNFRSF1B	UCL	-0.013213438	0.155684699	-0.084873067	0.933023126	0.978479525
MENT	NIH	-0.006406667	0.075606581	-0.084736892	0.933073548	0.969879221
PGF	UCL	-0.009425405	0.111529063	-0.084510755	0.933288032	0.978479525
MAP2	NIH	-0.013606667	0.161707189	-0.084143857	0.933540788	0.970020179
SIGLEC15	UCL	-0.038337844	0.457280166	-0.083838851	0.933628838	0.978485871
CD34	UCL	-0.008597579	0.10301252	-0.083461494	0.934114237	0.978643697
CACYBP	NIH	0.026213333	0.315038568	0.083206743	0.93427917	0.970442675
TIMP1	UCL	-0.01204663	0.145666532	-0.082700057	0.93473106	0.97893905
AGRP	UCL	-0.020308682	0.247130583	-0.082177938	0.935121093	0.97899676
FNTA	NIH	0.00994	0.121080253	0.082094311	0.935155768	0.971008385
PGLYRP4	UCL	0.017381871	0.215981465	0.080478529	0.936299597	0.97957872
HLA-E	NIH	-0.008233333	0.102423033	-0.080385565	0.936502424	0.971829051
VWA5A	NIH	-0.024233333	0.301985619	-0.080246647	0.936611913	0.971829051
ING1	UCL	-0.032655596	0.408371963	-0.079965323	0.93668104	0.97957872
IL31	NIH	-0.014073333	0.176298503	-0.079826732	0.93694288	0.971829051
SLC4A1	UCL	-0.013661808	0.173109088	-0.078920222	0.937422042	0.97957872
VNN2	UCL	0.016550545	0.20973707	0.07891092	0.937700043	0.97957872
UNG	UCL	-0.01225822	0.156427	-0.07836384	0.937855811	0.97957872
BTNL10	NIH	-0.017353333	0.220719057	-0.078621817	0.937892627	0.972246142
PDE1C	UCL	-0.01692424	0.21576614	-0.078437888	0.937963286	0.97957872
BCL7B	NIH	0.009753333	0.124288726	0.078473194	0.938009783	0.972246142

SYNGAP1	UCL	-0.017251943	0.220114894	-0.078376991	0.938022866	0.97957872
TGFBI	NIH	0.009453333	0.121961541	0.077510773	0.938768466	0.972687837
MYH4	UCL	-0.028921819	0.373768922	-0.077378875	0.938894389	0.979804107
BCHE	UCL	-0.00776162	0.101030302	-0.076824671	0.939333489	0.979804107
CRHBP	UCL	-0.009724166	0.126605116	-0.076807054	0.939342301	0.979804107
GNLY	UCL	-0.016675016	0.218556927	-0.076295985	0.939752443	0.979804107
BTN3A2	UCL	-0.012815265	0.168421459	-0.07609045	0.939914716	0.979804107
LILRA4	NIH	-0.02066	0.27362791	-0.075503994	0.940350612	0.973982131
ALDH1A1	UCL	0.018624397	0.248195125	0.075039335	0.940759551	0.980335176
NUDT2	NIH	0.013393333	0.18073394	0.074105247	0.941453531	0.97477932
USP47	UCL	-0.025927044	0.353081671	-0.073430727	0.941965844	0.981242395
TIGIT	NIH	-0.011506667	0.15713047	-0.073230015	0.942143717	0.975148752
FBN2	UCL	0.020254971	0.282265728	0.07175852	0.943049755	0.981762118
PRRT3	UCL	0.011098239	0.154267873	0.071941352	0.943147002	0.981762118
IFNLR1	UCL	-0.009669064	0.135121679	-0.071558202	0.943472387	0.981762118
DNAJA2	NIH	-0.02396	0.337316564	-0.071031199	0.943877849	0.976598057
CAT	UCL	-0.009947731	0.140886814	-0.070607965	0.944131389	0.98178964
CD164L2	UCL	0.013024419	0.18438627	0.070636599	0.944170605	0.98178964
TNPO1	NIH	0.019266667	0.273303075	0.070495609	0.944300293	0.97668966
CNTNAP2	UCL	0.012623733	0.180749196	0.069841156	0.944801153	0.982095935
ITIH4	UCL	0.005824448	0.084400802	0.069009396	0.945468654	0.982196681
SLC9A3R2	NIH	0.010573333	0.15333216	0.06895705	0.945513917	0.977599225
TCOF1	UCL	0.007624848	0.110692249	0.068883303	0.94557012	0.982196681
FAM3B	UCL	0.012112236	0.178020725	0.068038347	0.946284629	0.982366104
NPTN	NIH	-0.022006667	0.323802946	-0.067963145	0.946297985	0.978064172
DDI2	UCL	0.014345844	0.211997572	0.06766985	0.946405388	0.982366104
FCAMR	UCL	0.025404696	0.377226893	0.06734593	0.946803827	0.982430808
BRD2	NIH	-0.009086667	0.135575152	-0.067023098	0.947039618	0.978356484
GBP2	NIH	0.02914	0.436510238	0.066756739	0.947249765	0.978356484
COMP	UCL	0.012400645	0.186617166	0.06644965	0.947497168	0.982801357
RAB2B	NIH	0.01648	0.248571292	0.066298887	0.947611003	0.97838411
NAPRT	NIH	0.010733333	0.163902918	0.06548592	0.948252448	0.978700922
IGF1R	UCL	0.004819332	0.07465021	0.064558852	0.948934637	0.983943222

EXTL1	NIH	0.016453333	0.257943391	0.063786605	0.949593351	0.979739172
ADAM15	UCL	0.009433544	0.150700219	0.062598075	0.950549239	0.984994997
LY9	NIH	0.009193333	0.14711262	0.062491806	0.950615159	0.980102453
IZUMO1	UCL	0.012758372	0.204414259	0.062414296	0.950622952	0.984994997
CNTNAP2	NIH	-0.01024	0.16572478	-0.06178919	0.951169673	0.980102453
TNFSF11	NIH	0.0164	0.266842213	0.061459541	0.951429846	0.980102453
WARS	NIH	0.00644	0.105000045	0.061333307	0.951529476	0.980102453
CASP2	NIH	0.02326	0.380756468	0.061088916	0.951722364	0.980102453
TRIM24	NIH	0.01414	0.232592555	0.060793003	0.951955921	0.980102453
MED21	UCL	-0.014598463	0.240734182	-0.06064142	0.952057018	0.986017501
C3	UCL	0.006572692	0.109439815	0.060057594	0.95249997	0.986017501
VPS28	NIH	0.01172	0.195089247	0.060075069	0.952522586	0.980340804
CPOX	UCL	0.006924924	0.115660354	0.059872926	0.952621767	0.986017501
CORO1A	NIH	-0.02304	0.389428093	-0.059163682	0.95324198	0.980736121
C1RL	UCL	0.004539196	0.07724821	0.058761186	0.953538481	0.986502506
SPACA5_SPACA5B	UCL	0.009316624	0.159954897	0.058245316	0.953972079	0.986502506
GPR158	UCL	0.005881669	0.102201527	0.057549715	0.954403139	0.986502506
OSTN	UCL	0.008643436	0.151064817	0.057216738	0.954793171	0.986502506
FCGR2A	UCL	0.011641674	0.204101741	0.057038581	0.954933796	0.986502506
IFNAR1	NIH	-0.00568	0.099697102	-0.056972569	0.954971676	0.981881366
GABARAPL1	UCL	-0.009287126	0.163883733	-0.056668991	0.955228759	0.986502506
CDC25A	NIH	-0.0131	0.231456823	-0.056598029	0.955267365	0.981881366
NTF4	NIH	0.007066667	0.125122607	0.056477937	0.955362177	0.981881366
SUSD2	UCL	-0.007947089	0.140943754	-0.056384829	0.95545282	0.986502506
KIR2DL3	NIH	0.020013333	0.363976643	0.054985213	0.956540716	0.982442537
LUZP2	UCL	-0.006224796	0.113778491	-0.054709776	0.956686583	0.987259944
UHRF2	NIH	0.012266667	0.224614878	0.054611995	0.956835396	0.982442537
KRT18	UCL	-0.021676692	0.396814609	-0.054626748	0.95686193	0.987259944
CLEC5A	NIH	-0.007966667	0.146636332	-0.054329419	0.957058512	0.982442537
FGF19	UCL	-0.016823597	0.313507565	-0.05366249	0.957502917	0.987572698
RTKN2	NIH	0.01524	0.283702503	0.053718243	0.957541096	0.982442537
FAM171A2	NIH	-0.026326667	0.490625683	-0.053659373	0.957587581	0.982442537
NBL1	UCL	0.006469174	0.121777477	0.053122907	0.958043862	0.987782085

NAGA	UCL	0.017290179	0.328619572	0.052614575	0.95842203	0.987823552
SCN3B	UCL	0.007733515	0.14940409	0.051762408	0.958948683	0.988017977
THAP12	UCL	-0.015328497	0.298370373	-0.051374059	0.95939834	0.988132962
KCNIP4	NIH	0.01354	0.266666303	0.050775069	0.959865252	0.984328061
LAMA4	NIH	0.00628	0.124406224	0.05047979	0.960098447	0.984328061
LYZL2	UCL	0.005936899	0.11817524	0.050238094	0.960243835	0.988183892
SIRPB1	UCL	0.010225091	0.205622267	0.049727547	0.960716144	0.988183892
TJP3	UCL	0.007820956	0.157764982	0.049573461	0.960716919	0.988183892
CTBS	UCL	0.006190308	0.126363555	0.04898808	0.961296091	0.988183892
TNFSF8	NIH	-0.006333333	0.129937224	-0.048741486	0.961471336	0.985305509
CD48	UCL	0.007759864	0.159241875	0.048730044	0.961494387	0.988183892
NAA10	UCL	-0.014598786	0.300847695	-0.048525502	0.961531709	0.988183892
CCDC28A	UCL	0.005284973	0.10965592	0.048195964	0.961814291	0.988183892
EPHA4	NIH	0.005266667	0.10948245	0.048105123	0.961973957	0.985305509
OFD1	NIH	-0.018606667	0.38769428	-0.047993142	0.962062405	0.985305509
B4GAT1	NIH	0.005233333	0.110073933	0.047543802	0.96241732	0.985323998
AHSG	UCL	-0.004720564	0.099989064	-0.047210801	0.962685863	0.988731826
SOST	NIH	0.007813333	0.167419314	0.046669247	0.963108119	0.98537328
EVI5	NIH	0.013366667	0.288719763	0.046296334	0.963402687	0.98537328
SERPINA12	NIH	-0.026153333	0.566047791	-0.046203401	0.963476096	0.98537328
IL12RB2	NIH	0.009406667	0.208012922	0.04522155	0.964251698	0.985402564
TMEM132A	UCL	-0.012666285	0.280914362	-0.045089489	0.964282245	0.98967224
MATN3	NIH	0.00512	0.115568534	0.044302716	0.964977552	0.985402564
UNC5D	UCL	0.00847064	0.191867486	0.044148389	0.965059359	0.98967224
SPINT2	UCL	0.005371735	0.121662507	0.044152753	0.965093526	0.98967224
COL9A2	NIH	0.009453333	0.214538039	0.04406367	0.965166398	0.985402564
INPP1	NIH	-0.00676	0.153440699	-0.044056108	0.965172372	0.985402564
HTR1A	NIH	-0.009473333	0.215132712	-0.044034834	0.965189178	0.985402564
OMG	UCL	-0.014161938	0.325309023	-0.0435338	0.965520358	0.98967224
SERPINB5	NIH	0.032266667	0.740579369	0.043569492	0.965556803	0.985433932
LYPD8	UCL	0.034916038	0.803141798	0.043474313	0.965581165	0.98967224
RBKS	UCL	-0.009255749	0.212857571	-0.043483299	0.96563299	0.98967224
CST5	NIH	0.00954	0.222721246	0.042833812	0.966138012	0.985683182

SH3GL3	UCL	-0.013892552	0.333711903	-0.041630375	0.966995165	0.990720948
CD160	UCL	-0.008627692	0.213764009	-0.040360828	0.968113332	0.990908597
ITPR1	UCL	-0.007373529	0.18332481	-0.040221117	0.968193958	0.990908597
OXCT1	UCL	0.009312472	0.233220942	0.039929829	0.968375778	0.990908597
NFASC	UCL	0.00664465	0.166863412	0.03982089	0.968534335	0.990908597
PLA2G2A	UCL	0.015583471	0.401512381	0.038811931	0.969349982	0.99139608
DPEP2	UCL	-0.003294839	0.086587235	-0.038052245	0.96994973	0.991662491
GLYR1	NIH	-0.01312	0.352016853	-0.037270943	0.970533435	0.989754913
COL4A4	NIH	0.026073333	0.706090567	0.03692633	0.970805759	0.989754913
IFNGR1	UCL	0.003722542	0.101145895	0.03680369	0.970907352	0.992133489
IL4R	UCL	0.006139372	0.16772646	0.036603481	0.971089261	0.992133489
C19orf12	UCL	-0.015136201	0.41947645	-0.036083554	0.971499795	0.992206115
SULT1A1	NIH	-0.014746667	0.414820663	-0.035549499	0.971893808	0.990485109
GPIHBP1	NIH	0.005106667	0.1452283	0.035163027	0.97219923	0.990485109
KLK1	UCL	-0.015482275	0.446139715	-0.03470275	0.972579202	0.992929322
GTPBP2	NIH	-0.01334	0.389307624	-0.034265961	0.972908181	0.99063122
CNTN1	UCL	0.004086401	0.119275515	0.034260184	0.972920707	0.992929322
DNMBP	NIH	0.00896	0.262568126	0.034124477	0.973019998	0.99063122
CLEC4C	NIH	0.00966	0.289309323	0.033389868	0.97360058	0.990806988
PRKAG3	NIH	-0.005726667	0.173278948	-0.033048831	0.973870116	0.990806988
CST3	UCL	0.004377325	0.133863212	0.032699986	0.974150915	0.992929322
HSP90B1	NIH	-0.021706667	0.682120556	-0.031822332	0.974839496	0.991237132
VIT	NIH	-0.005653333	0.178584441	-0.03165636	0.974970677	0.991237132
APBB1IP	UCL	-0.008845711	0.279627741	-0.031633882	0.974990439	0.992929322
SEMA6C	UCL	-0.004991937	0.159222038	-0.031352048	0.975184705	0.992929322
ADGRF5	NIH	-0.00224	0.07237961	-0.030947942	0.975530605	0.991394221
KIT	UCL	-0.003939213	0.127363941	-0.030928795	0.975552098	0.992929322
CTSH	UCL	-0.008922231	0.288676516	-0.030907367	0.975575738	0.992929322
ADAM23	UCL	-0.003457075	0.111929645	-0.030886141	0.975585726	0.992929322
CCL11	UCL	0.005533572	0.180019761	0.030738692	0.97570191	0.992929322
IFIT1	UCL	-0.006831512	0.227228258	-0.030064536	0.976164166	0.992929322
KHK	UCL	0.00827495	0.277427483	0.029827433	0.976412601	0.992929322
OTOA	NIH	-0.01116	0.375542198	-0.029717033	0.976503536	0.991394221

LY6D	UCL	0.00473952	0.160092069	0.029604967	0.976591122	0.992929322
MANSC4	UCL	0.008587198	0.290700509	0.029539673	0.976623948	0.992929322
EPO	NIH	-0.008073333	0.273617864	-0.029505871	0.976670446	0.991394221
SHD	NIH	0.013	0.452678661	0.028717943	0.977293261	0.991394221
TCN1	NIH	0.00408	0.142689865	0.028593481	0.977391643	0.991394221
GP1BB	NIH	0.00746	0.26451535	0.028202522	0.977700682	0.991394221
IDO1	NIH	-0.005193333	0.18459706	-0.028133348	0.977755362	0.991394221
TANK	NIH	0.0133	0.474483856	0.028030458	0.977836693	0.991394221
METAP1	UCL	0.006614946	0.236178695	0.028008225	0.977840178	0.993015561
KIAA0319	UCL	-0.003069377	0.110483007	-0.027781437	0.978020361	0.993015561
HMMR	UCL	0.005322865	0.192954303	0.027586141	0.978176266	0.993015561
GSTM4	UCL	-0.013171425	0.48054738	-0.027409212	0.978334582	0.993015561
CCL3	UCL	0.005259456	0.192565774	0.027312515	0.978407395	0.993015561
DRAXIN	NIH	0.005406667	0.205542532	0.026304369	0.979201152	0.9924336
MYO6	NIH	-0.00348	0.13825713	-0.025170492	0.980097508	0.992463903
DNAJA4	UCL	-0.006039677	0.243688349	-0.024784432	0.980387544	0.9935042
ELOA	UCL	-0.004277577	0.173033378	-0.024721107	0.980388293	0.9935042
BOC	NIH	0.003326667	0.135203167	0.024604946	0.980544594	0.992463903
PRSS2	NIH	-0.006266667	0.257117154	-0.024372807	0.980728112	0.992463903
ADGRE2	NIH	0.002406667	0.099169642	0.024268179	0.980810825	0.992463903
PTPRF	NIH	0.002453333	0.10171153	0.024120504	0.980927571	0.992463903
CLEC5A	UCL	0.003318547	0.143388928	0.023143679	0.981688422	0.9935042
CTSO	UCL	0.003074157	0.13436071	0.022879884	0.981906154	0.9935042
BAMBI	UCL	-0.003318296	0.14643492	-0.022660554	0.982057301	0.9935042
IFIT3	UCL	-0.005263942	0.232994147	-0.022592592	0.982119116	0.9935042
FOLR3	UCL	-0.018412932	0.815457217	-0.022579887	0.982152154	0.9935042
GRP	UCL	0.004654205	0.206783151	0.022507659	0.982213288	0.9935042
RRM2	UCL	0.004668956	0.207265871	0.022526408	0.982219647	0.9935042
OSCAR	UCL	0.003202034	0.14289328	0.022408571	0.982287765	0.9935042
LILRB1	UCL	-0.00198193	0.098096944	-0.020203786	0.984030872	0.994922947
BLOC1S2	NIH	-0.009133333	0.454575915	-0.020091987	0.984112488	0.995139396
ESM1	NIH	-0.0028	0.141339739	-0.019810423	0.984335102	0.995139396
RAB44	UCL	0.004151067	0.212985484	0.019489905	0.984572969	0.995126829

TG	NIH	0.005853333	0.300406434	0.019484714	0.984592619	0.995139396
CDK5RAP3	NIH	0.003086667	0.162696012	0.018971987	0.984998002	0.995205235
RBP5	UCL	0.004276181	0.236310778	0.018095581	0.985701245	0.995594292
CNTF	NIH	-0.006146667	0.34198242	-0.017973633	0.985787355	0.995658844
PPIF	UCL	-0.00216274	0.122747437	-0.017619427	0.986019257	0.995594292
ATP1B2	UCL	0.001947893	0.110967721	0.017553688	0.986057296	0.995594292
CETN2	NIH	-0.005693333	0.337832806	-0.016852518	0.986673787	0.995910399
GRN	NIH	-0.001506667	0.089696505	-0.016797384	0.98671738	0.995910399
CHIT1	UCL	0.009786921	0.582692202	0.01679604	0.986726012	0.995653853
KRT5	UCL	0.003410744	0.204316515	0.016693434	0.986797541	0.995653853
CDSN	NIH	-0.003526667	0.2175039	-0.016214269	0.987178439	0.996032057
DDX39A	UCL	0.001574273	0.099392978	0.015838871	0.987434975	0.99595322
CEP170	NIH	0.005413333	0.370198412	0.014622789	0.988436816	0.996346092
FCRL6	NIH	-0.0039	0.278737641	-0.013991652	0.988935862	0.996346092
HRAS	NIH	0.005473333	0.398601576	0.013731339	0.989141696	0.996346092
NPTX1	UCL	-0.002708816	0.197735195	-0.013699209	0.989172071	0.99697162
CST6	UCL	0.003211668	0.238027939	0.013492817	0.989334251	0.99697162
CST1	UCL	0.006479237	0.486188481	0.013326594	0.989467899	0.99697162
GIPC3	NIH	-0.005746667	0.440858325	-0.013035178	0.989692165	0.996346092
EEF1D	NIH	-0.003686667	0.285426766	-0.012916331	0.989786139	0.996346092
CCL21	NIH	0.002206667	0.176100432	0.012530728	0.990091046	0.996346092
FKBP5	NIH	-0.00474	0.38563903	-0.012291287	0.99028038	0.996346092
ART5	UCL	0.003067671	0.254519022	0.012052818	0.990451838	0.997619133
GH2	NIH	-0.007793333	0.690618554	-0.01128457	0.991076428	0.996346092
MCAM	NIH	-0.001746667	0.154997672	-0.011268986	0.991088751	0.996346092
EDN1	NIH	-0.001406667	0.127315771	-0.011048644	0.991262985	0.996346092
COPB2	NIH	0.002926667	0.265621244	0.011018195	0.991287062	0.996346092
GAL	UCL	-0.002900337	0.271182446	-0.01069515	0.991544635	0.997741925
CXCL1	UCL	0.005915965	0.575625882	0.010277448	0.991868683	0.997741925
LTO1	UCL	0.001285322	0.126007265	0.010200383	0.991915623	0.997741925
VSIG10	NIH	0.0026	0.259076645	0.01003564	0.992064016	0.996346092
LYPLA2	NIH	0.003113333	0.316189204	0.009846425	0.992213638	0.996346092
KIR2DL2	NIH	0.003893333	0.397698122	0.00978967	0.992258518	0.996346092

CTRB1	UCL	0.002058346	0.216481584	0.009508183	0.992481591	0.997741925
PGLYRP1	UCL	0.00131858	0.142781446	0.009234951	0.992697194	0.997741925
DSC2	UCL	-0.001605606	0.177846087	-0.009028067	0.992865036	0.997741925
LRRC59	NIH	-0.001773333	0.197810379	-0.008964814	0.992910779	0.996658898
CD86	UCL	0.001355982	0.152362833	0.008899693	0.99296314	0.997741925
HADH	NIH	-0.003406667	0.408970893	-0.008329851	0.993412884	0.996820819
NEDD9	UCL	0.001113217	0.165952069	0.006708061	0.994686502	0.999130118
CXCL9	NIH	0.0013	0.198975614	0.006533464	0.994833415	0.997513271
CPA2	UCL	-0.001951761	0.318287584	-0.006132067	0.995152645	0.999190064
CINP	NIH	0.00114	0.187255801	0.006087929	0.995185734	0.997513271
C2	NIH	0.000493333	0.082650155	0.005968934	0.995279833	0.997513271
C4BPB	UCL	0.000837373	0.144770564	0.005784137	0.995429855	0.999190064
FGF19	NIH	0.001913333	0.333790803	0.005732133	0.99546709	0.997513271
TPRKB	NIH	0.001813333	0.349115456	0.005194079	0.995892573	0.997597869
CTNNA1	UCL	-0.000745513	0.157375288	-0.004737168	0.996251776	0.999471664
MTSS1	NIH	0.001353333	0.305002927	0.004437116	0.996491168	0.997855757
WASF1	NIH	-0.000966667	0.242467046	-0.003986796	0.996847275	0.997870732
CLEC14A	UCL	0.000465831	0.121531768	0.003832995	0.996970483	0.999471664
ACSL1	UCL	-0.00046961	0.130005598	-0.003612226	0.997139457	0.999471664
AIF1	UCL	-0.001449546	0.491482724	-0.002949332	0.997657258	0.999471664
RAB37	NIH	-0.000853333	0.316744	-0.002694079	0.997869542	0.998552313
CEACAM6	UCL	0.000310665	0.146658635	0.002118289	0.998323555	0.999471664
CDKL5	UCL	-0.000461657	0.228839567	-0.002017383	0.998405413	0.999471664
BCL7B	UCL	0.000259605	0.137683264	0.00188552	0.998506527	0.999471664
LY75	UCL	0.00031456	0.176171704	0.001785533	0.998588375	0.999471664
DEFB4A_DEFB4B	UCL	0.000928201	0.678707278	0.001367601	0.998919124	0.999471664
S100A14	UCL	-0.000241092	0.254674572	-0.000946667	0.999251284	0.999471664
COL28A1	NIH	0.000313333	0.355682388	0.000880936	0.999303362	0.999645121
CEACAM19	UCL	0.000123377	0.184665233	0.00066811	0.999471664	0.999471664
SAG	NIH	9.33E-05	0.545712507	0.00017103	0.99986475	0.99986475

Supplemental Table 2. SBMA vs Control - Gene Set Enrichment Analysis Results

Gene Set	Gene Set Size	Gene Set Category	Study	Normalized Enrichment Score	P	padj	Proteins Driving Enrichment
Skeletal Muscle	75	HPA Tissue	UCL	2.926661797	3.48995309296756e-14	1.8496751392728e-12	CSRP3, MYBPC1, CA3, MYL3, HSPB6, MB, ACTN2, TPM3, TTN, FABP3, NEB, CORO6, MYLPF, MYBPC2, ENO3, CHCHD10, KLHL41, CAPN3, MYOM1, NDUFS6, BAG3, MYL1, IL32, TMOD4
Muscle Contraction	80	Biological Process	UCL	2.788158408	5.58668436257884e-14	1.08437543477655e-10	CSRP3, MYBPC1, MYOM3, MYL3, HSPB6, MB, ACTN2, TPM3, NOS1, TTN, NEB, MYLPF, MYOM2, MYBPC2, SORBS1, KLHL41, MYOM1, TNNI3, DMD, MYL1, TMOD4, KCNH2, MYL6B, LMOD2, MYL4, GSN
Muscle System Process	105	Biological Process	UCL	2.597575063	2.85389107303775e-13	2.76970128638313e-10	CSRP3, MYBPC1, MYOM3, MYL3, HSPB6, MB, ACTN2, TPM3, NOS1, TTN, NEB, MYLPF, MYOM2, MYBPC2, SORBS1, KLHL41, MYOM1, TNNI3, DMD, MYL1, TMOD4, KCNH2, MYL6B, LMOD2, MYL4, AGT, GSN
Skeletal Muscle	75	HPA Tissue	NIH	2.176739464	5.52034740884817e-13	2.92578412668953e-11	TTN, CORO6, MYL3, MYBPC1, CA3, CSRP3, TPM3, HSPB6, FABP3, CHCHD10, KLHL41, MYL1, ACTN2, MB, CAPN3, GPD1, MYLPF, ENO3, NEB, COX6B1, AAMDC, CTSD, MYBPC2, MDH1, ACSL1, GAPDH, NDUFS6, GLRX, BAG3, PRDX5, PEBP1, PARK7, GNAS, HJV
Tongue	52	HPA Tissue	UCL	2.910879712	7.8043150908987e-13	2.06814349908815e-11	CSRP3, MYBPC1, CA3, MYL3, HSPB6, MB, ACTN2, TPM3, FABP3, NEB, MYLPF, MYBPC2, ENO3, CHCHD10, KLHL41, GOT1, MYL1, TMOD4
Muscle Contraction	80	Biological Process	NIH	2.104674128	1.43860840951237e-12	2.79233892286351e-09	DMD, TTN, MYL3, TNNI3, MYBPC1, CSRP3, TPM3, HSPB6, MYOM2, MYOM3, KLHL41, MYL1, ACTN2, SORBS1, MB, NOS1, MYLPF, NEB, SLMAP, MYBPC2, MYL4
Structural Constituent Of Muscle	18	Molecular Function	NIH	2.359855237	6.80210328333532e-12	1.66651530441715e-09	DMD, TTN, MYL3, MYBPC1, CSRP3, MYOM2, MYL1, ACTN2, CAPN3, MYLPF, NEB, MYBPC2

Contractile Fiber	54	Cellular Components	NIH	2.150786367	7.85220422368089e-11	1.79815476722292e-08	DMD, TTN, MYL3, TNNI3, MYBPC1, CSRP3, TPM3, MYOM2, MYOM3, KLHL41, MYL1, ACTN2, CAPN3, MYLPF, NEB, MYL4, MYH7B, BAG3, ITGB1BP2, AHNAK
RNA Binding	216	Molecular Function	UCL	-1.882578403	1.1118956481331e-10	2.72414433792611e-08	SERPINH1, PPP1CC, TRIM21, STIP1, EEF1D, CORO1A, FARSA, SF3B4, EIF4G1, USO1, HDGF, MTIF3, TWF2, DRG2, NAP1L4, RBPMS2, EIF4E, ANXA11, TRIM25, ARHGEF1, YARS1, EIF4G3, NUDT5, EIF4B, TARBP2, DARS1, TDRKH, NFX1, GIGYF2, ENO1, PTPN1, DNM1, YTHDF3, STXBP1, RBPMS, FAM172A, DUT, METAP2, HEXIM1, ANXA2, IGHMBP2, DTD1, TARS1, LRRFIP1, CRKL, PCBP2, TIA1, PDIA4, PDAP1, MARS1, EIF2AK2, TBCA, LARP1, DXO, MANF, MTDH, HSPB1, MYH9, CASC3, MTHFSD, TPD52L2, S100A4, P4HB, EIF2S2, CNP, PPIB, HNRNPUL1, TST, PARK7, TRDMT1, GTPBP2, DNAJC21, RBM17, ERI1, FKBP4, HNRNPK, LACTB2, RANBP2, CIRBP, LONP1, LBR, EIF5, TOP1, PNPT1, SNU13, DDX58, FMR1, SUGP1, GRSF1, PRKRA, PEBP1
Myogenesis	70	Hallmark	UCL	2.623623922	1.45653061168114e-10	6.55438775256514e-09	CSRP3, MYL3, MB, ACTN2, TPM3, NOS1, FABP3, MYLPF, MYOM2, ENO3, SORBS1, MYOM1, DMD, MYL1, FST, SOD3, KCNH2, MYL6B, NCAM1, MYL4, GSN
Striated Muscle Cell Development	29	Biological Process	UCL	2.81234979	2.96678299541996e-10	1.91950859803672e-07	CSRP3, ACTN2, TTN, NEB, KLHL41, CAPN3, DMD, TMOD4, WFIKKN2, LMOD2, DNER, KEL, PDGFRA
Contractile Fiber	54	Cellular Components	UCL	2.587573812	9.36428098300133e-10	2.1350560641243e-07	CSRP3, MYBPC1, MYOM3, MYL3, ACTN2, TPM3, TTN, NEB, MYLPF, MYOM2, KLHL41, CAPN3, MYOM1,

							TNNI3, DMD, BAG3, MYL1, TMOD4, MYL6B, LMOD2, MYL4
Actin Mediated Cell Contraction	28	Biological Process	UCL	2.745880501	1.01641612081771e-09	4.93215922626793e-07	MYBPC1, MYL3, ACTN2, TPM3, TTN, NEB, MYBPC2, TNNI3, DMD, MYL1, KCNH2, MYL6B, MYL4, GSN
Tongue	52	HPA Tissue	NIH	2.080754222	1.42325777750478e-09	3.77163311038767e-08	MYL3, MYBPC1, CA3, CSRP3, TPM3, HSPB6, FABP3, CHCHD10, KLHL41, MYL1, ACTN2, MB, MYLPF, ENO3, NEB, COX6B1, MYBPC2, MDH1, GOT1, GAPDH
Muscle System Process	105	Biological Process	NIH	1.862332346	3.44845096095288e-09	3.34672165760477e-06	DMD, TTN, MYL3, TNNI3, MYBPC1, CSRP3, TPM3, HSPB6, MYOM2, MYOM3, KLHL41, MYL1, ACTN2, SORBS1, MB, NOS1, MYLPF, NEB, SLMAP, MYBPC2, LEP, MYL4
Actin Filament Based Movement	34	Biological Process	UCL	2.683126621	4.12741422742806e-09	1.14447300220541e-06	MYBPC1, MYL3, ACTN2, TPM3, TTN, NEB, MYBPC2, TNNI3, DMD, MYL1, KCNH2, MYL6B, MYL4, GSN
Structural Molecule Activity	139	Molecular Function	UCL	2.193478337	4.9955106044911e-09	6.11950049050159e-07	CSRP3, MYBPC1, MYL3, HSPB6, ACTN2, TTN, NEB, MYLPF, MYOM2, MYBPC2, CAPN3, MYOM1, DMD, MYL1, POSTN, HMCN2, ADIPOQ, TNXB, MYL6B, UMOD, DPT, EDIL3, IMPG1, SEPTIN7, APOE, MATN2, SPON1, TPR, LMNB1
Actin Mediated Cell Contraction	28	Biological Process	NIH	2.212045786	5.50125687455867e-09	3.55931319783946e-06	DMD, TTN, MYL3, TNNI3, MYBPC1, TPM3, MYL1, ACTN2, NEB, MYBPC2, MYL4
Actin Filament Based Movement	34	Biological Process	NIH	2.126769784	1.40280989122444e-08	6.80713499716661e-06	DMD, TTN, MYL3, TNNI3, MYBPC1, TPM3, MYL1, ACTN2, NEB, MYBPC2, MYL4
Striated Muscle Contraction	36	Biological Process	NIH	2.098448207	3.72283260121423e-08	1.44520361579136e-05	DMD, TTN, MYL3, TNNI3, CSRP3, KLHL41, MYL1, MB, NOS1, MYL4
Supramolecular Polymer	166	Cellular Components	NIH	1.675666658	4.04364579148972e-08	4.62997443125573e-06	DMD, TTN, MYL3, TNNI3, MYBPC1, CSRP3, TPM3, MYOM2, MYOM3, KLHL41, MYL1, ACTN2, CAPN3, MYLPF, NEB, LMNB2, CRHBP, MYBPC2, TBCA, MYL4, TBCC, MYH7B,

							AMOT, CLMP, COL3A1, COL6A3, BAG3, REEP4, FKBP4, ITGB1BP2, SIRT2, MTUS1, PPL, DCTN1, KRT6C, NDRG1, AHNAK, LDLRAP1, DCXR, XIAP, EHBP1, ELN, COL2A1, DNM3, KRT18, TMOD4, DBN1, SNCA, MFAP5, KIF1C
Structural Constituent Of Muscle	18	Molecular Function	UCL	2.542791492	4.96114550504971e-08	4.05160216245726e-06	CSRP3, MYBPC1, MYL3, ACTN2, TTN, NEB, MYLPF, MYOM2, MYBPC2, CAPN3, MYOM1, DMD, MYL1, MYL6B
Striated Muscle Contraction	36	Biological Process	UCL	2.553614704	9.86894216407827e-08	1.74141970367963e-05	CSRP3, MYL3, MB, NOS1, TTN, KLHL41, TNNI3, DMD, MYL1, KCNH2, MYL4, GSN
Myogenesis	70	Hallmark	NIH	1.870916437	1.12111468908989e-07	5.04501610090451e-06	DMD, MYL3, CSRP3, TPM3, MYOM2, FABP3, MYL1, ACTN2, SORBS1, MB, NOS1, MYLPF, ENO3, MYL4, ACSL1, COL3A1, COL6A3, FST, CFD, SIRT2, ADAM12, AGRN
Heart Muscle	71	HPA Tissue	UCL	2.249299434	1.14191887919671e-06	2.01739001991419e-05	CSRP3, MYL3, HSPB6, MB, ACTN2, FABP3, CHCHD10, GOT1, TNNI3, LMOD2, MYL4, GSN
Myofibril Assembly	16	Biological Process	NIH	2.088559155	1.35235401333404e-06	0.000437487	TTN, CSRP3, KLHL41, ACTN2, CAPN3, NEB
Myofibril Assembly	16	Biological Process	UCL	2.46672185	1.57085734378459e-06	0.000145192	CSRP3, ACTN2, TTN, NEB, KLHL41, CAPN3, TMOD4, LMOD2, PDGFRA
Actin Binding	92	Molecular Function	UCL	1.962590524	5.7803325419848e-06	0.000283236	CSRP3, MYBPC1, MYOM3, MYL3, ACTN2, TPM3, TTN, NEB, CORO6, MYOM2, MYBPC2, SORBS1, MYOM1, TNNI3, DMD, TMOD4
Actin Binding	92	Molecular Function	NIH	1.697322166	8.01308681586479e-06	0.000981603	DMD, TTN, CORO6, MYL3, TNNI3, MYBPC1, CSRP3, TPM3, MYOM2, MYOM3, ACTN2, SORBS1, NEB, HIP1R, MYBPC2, MYL4, MYH7B, ABL1, IMPACT, MPRIP, TMOD4, DBN1, SNCA, INPPL1, TMSB10, AIF1, CAPG, S100A4, SCIN, NEXN, GAS2

Dilated Cardiomyopathy Signaling Pathway	25	IPA	NIH	1.978665665	9.91291004327763e-06	0.00313248	DMD, TTN, MYL3, TNNI3, MYL1, CACNB3, MYL4, MYH7B, BAG3
Heart Process	58	Biological Process	UCL	2.096461318	1.15296509500646e-05	0.000790729	CSRP3, MYL3, NOS1, TTN, TNNI3, DMD, MYL1, KCNH2, MYL4, AGT, GSN, CACNB1, CHGA, ACE, ATP2B4, SCN2B, NPR1
I Band	30	Cellular Components	NIH	1.901697953	1.40387498624556e-05	0.000803718	DMD, TTN, MYL3, CSRP3, ACTN2, CAPN3, NEB, BAG3, ITGB1BP2
Cardiac Muscle Contraction	15	KEGG	NIH	1.96740844	1.87510181519276e-05	0.003543942	MYL3, TNNI3, TPM3, COX6B1, CACNB3, MYL4
Structural Molecule Activity	139	Molecular Function	NIH	1.558681622	2.30612585220536e-05	0.001513715	DMD, TTN, MYL3, MYBPC1, CSRP3, HSPB6, MYOM2, MYL1, ACTN2, CAPN3, MYLPF, NEB, COL18A1, MYBPC2, CRELD1, CD4, MRPL24, COL3A1, COL6A3, PRG3, MATN2, CLTA, PPL, AHNAK, AGRN, THBS2, PRG2, FBLN2, ELN, COL2A1, DNM3, COL24A1, KRT18, MFAP5, VWA1, COPE, TPR, HSPG2, BGN, TFPI2, VIM, NEXN, EFEMP1, DPT, HMGCL
Cytoskeletal Protein Binding	183	Molecular Function	NIH	1.498227085	2.47137074148311e-05	0.001513715	DMD, TTN, CORO6, MYL3, TNNI3, MYBPC1, CSRP3, TPM3, MYOM2, MYOM3, FABP3, ACTN2, SORBS1, CAPN3, HSPA2, NEB, HIP1R, MYBPC2, TBCA, B4GALT1, MYL4, TBCC, MYH7B, GAPDH, FTCD, ABL1, REEP4, FKBP4, PDCD5, MTUS1, DCTN1, NDRG1, IMPACT, MPRIP, DNM3, TMOD4, DBN1, SNCA, INPPL1, TMSB10, KIF1C, EP300, AIF1, NME1, TPR, CAPG, DCTN2, S100A4, SCIN, NEXN, GAS2, PDLIM5, GABARAPL1, CAMSAP1, PHACTR2, MYH9, MAP1LC3B2, PXN, CLSTN1, APOE, SNX5, PSRC1, MYOM1, MDM1, CLIP2, AXL, ADD1, CEP350, CDH1, CCT5, VASP, MAPT,

							IQGAP2, FMNL1, SMC3, GBP1, GMFG, OPHN1
Heart Muscle	71	HPA Tissue	NIH	1.712111391	2.97637697657677e-05	0.00039437	MYL3, TNNI3, CSRP3, HSPB6, FABP3, CHCHD10, ACTN2, MB, COX6B1, TIMP3, MDH1, GOT1, MYL4, HSPA1A, GAPDH, IGFBP4, TIMP1, CFD, GNAS, SAT1, CST3, CD36, TMSB10, BGN, CD74, VIM
Supramolecular Polymer	166	Cellular Components	UCL	1.665628196	4.25390095336291e-05	0.001385556	CSRP3, MYBPC1, MYOM3, MYL3, ACTN2, TPM3, TTN, NEB, MYLPF, MYOM2, MYBPC2, KLHL41, CAPN3, MYOM1, TNNI3, DMD, BAG3, MYL1, TMOD4, AMOT
Heart Process	58	Biological Process	NIH	1.755127563	5.01114687252084e-05	0.012158295	DMD, TTN, MYL3, TNNI3, CSRP3, MYL1, NOS1, CACNB3, YAP1, MYL4
Dilated Cardiomyopathy Signaling Pathway	25	IPA	UCL	2.180657124	7.278146270795e-05	0.023071724	MYL3, TTN, TNNI3, DMD, BAG3, MYL1, MYL6B, MYL4, CACNB1
Regulation Of Striated Muscle Contraction	19	Biological Process	NIH	1.925928412	7.83650052148318e-05	0.016900719	DMD, MYL3, TNNI3, NOS1
Actin Filament Binding	43	Molecular Function	NIH	1.755981844	0.000140396	0.005732851	TTN, CORO6, TNNI3, TPM3, MYOM2, MYOM3, ACTN2, NEB, HIP1R, MYL4, MYH7B, ABL1, MPRIP, TMOD4, DBN1, AIF1, CAPG, SCIN, NEXN, GAS2
Protein Modification By Small Protein Conjugation Or Removal	124	Biological Process	NIH	1.508036166	0.000159843	0.029529947	KLHL41, CAPN3, PSMD1, UBE2B, USP28, THOP1, OTUD7B, HSPA1A, DCUN1D1, UFD1, UBAC1, AMFR, NUP50, HGS, ABL1, TRIM58, ZBTB16, PARK7, COMMD1, ATXN3, XIAP, PSMD9, SEL1L, ZNRF4, STAMBP, NHLRC3, RNF149, TRAF2, UBXN1, CDC26, BECN1, RNF41, PSME1, EP300, MAPK9, PSME2, TOMM20, SMAD1, NEDD4L, TPR, UBE2Z, OTUD6B, TGFBR1, DTX3, UBE2L6, CLSPN, TRAF3, BLMH, DNAJB2, NFX1

Calcium Signaling	32	IPA	UCL	2.08427823	0.00016287	0.025814858	MYL3, TPM3, TNNI3, MYL1, CHP1, MYL6B, MYL4, ATF2, CACNB1, ATP2B4
Cytoskeletal Protein Binding	183	Molecular Function	UCL	1.569185983	0.000181644	0.003423286	CSRP3, MYBPC1, MYOM3, MYL3, ACTN2, TPM3, TTN, FABP3, NEB, CORO6, MYOM2, MYBPC2, SORBS1, HSPA2, CAPN3, MYOM1, TNNI3, DMD, TMOD4, CHP1
Striated Muscle Cell Development	29	Biological Process	NIH	1.826751592	0.000182565	0.029529947	DMD, TTN, CSRP3, KLHL41, ACTN2, CAPN3, NEB
I Band	30	Cellular Components	UCL	1.980901884	0.000208369	0.004750802	CSRP3, MYL3, ACTN2, TTN, NEB, CAPN3, DMD, BAG3
Actin Filament Binding	43	Molecular Function	UCL	1.969935667	0.000224397	0.003539583	MYOM3, ACTN2, TPM3, TTN, NEB, CORO6, MYOM2, MYOM1, TNNI3, TMOD4, MYL4, MPRIP, GSN
Regulation Of Striated Muscle Contraction	19	Biological Process	UCL	2.116499073	0.000255256	0.007839858	MYL3, NOS1, TNNI3, DMD, CHGA
Hypertrophic Cardiomyopathy	28	KEGG	UCL	2.09652998	0.000475595	0.010665314	MYL3, TPM3, TTN, TNNI3, DMD, AGT, CACNB1, ACE
Hypertrophic Cardiomyopathy	28	KEGG	NIH	1.748178824	0.000548726	0.02592728	DMD, TTN, MYL3, TNNI3, TPM3, CACNB3
Calcium Signaling	32	IPA	NIH	1.744533684	0.000665114	0.035029335	MYL3, TNNI3, TPM3, MYL1, CACNB3, MYL4, MYH7B
RNA Binding	217	Molecular Function	NIH	1.362715018	0.000792241	0.027728445	RBFOX3, NUDT16, IMMT, NGRN, ELAVL4, JUN, PCBP2, TBCA, HSPA1A, RIDA, LARP1, NUDT5, MRPL24, MTHFSD, PCSK9, FKBP4, TARS1, PEBP1, CSTB, AHNAK, YARS1, PARK7, HDGF, EIF5, TPD52L2, ATXN2L, MKI67, EIF2S2, MARS1, TXN, DENR, GPKOW, CKAP4, DDX25, LGALS3, EIF4G1, KRT18, METAP2, KIF1C, HEXIM1, DXO, DDX39A, EP300, LRRFIP1, SRP14, SMAD1, NME1, TPR, PDIA4, NAP1L4, STIP1, USO1, S100A4, C1QBP, VIM, STAU1, HNRNPUL1, SF3B4, SNRPB2, RNASET2, NFX1, MCTS1, DDX1, PDIA3, TCOF1,

							EIF4E, BAG4, YTHDF3, RBM17, MYH9, DTD1, TRIM21, HNRNPK, DUT, CSDE1, ADD1, ENO1, APEX1, CCAR2, FUS, SUGP1, SAFB2, RPS10, CWC15, CCT5, MAPT, RALY, DNAJC21, SART1, GIGYF2, LGALS1, TARBP2, SLIRP, EDF1, TSNAX, ZCCHC8, ATXN2, PPIE, HSPB1, CNP, ARHGEF1, LACTB2, ASS1, NPM1, RNASEH2A, DDX4, RPGR, PRKRA, FAM172A, TDRKH, EIF4G3, EIF2AK2, RBM25, ANG, POLR2A, PSIP1, CASC3, TRDMT1, PDAP1, CARHSP1, SMNDC1, DYNC1H1, LSM1, C7orf50, MTDH, EIF4B, EIF1AX, IGHMBP2, EXOSC10, LBR, ANXA11, MANF, TST, ACTN4, PPP1CC, DRG2, SHMT1, SSBP1, SRPK2, EPPK1, GRSF1, RANBP2, MTIF3, PNPT1, SKIV2L, RPL14, TRIM25, TP53, SOX2
Mitochondrion	246	Cellular Components	UCL	-1.445449696	0.001103006	0.013971415	PPP1CC, MTIF3, PLSCR3, DYNLT1, FXN, HARS1, AP3B1, NFU1, LYN, MAVS, GIT1, ABL1, METAP1D, PECR, WASF1, TP73, MECR, ACOT13, SRC, TDRKH, BID, RTN4IP1, PTPN1, HMGCL, CMC1, DNM1, CASP8, STXBP1, CIAPIN1, DECR1, BECN1, STK11, NIT1, HCLS1, ATXN3, SPART, DUT, PLA2G4A, RAB11FIP3, GLRX5, AK2, CLPP, YWHAQ, KIFBP, BCL2L1, PRDX5, SNCA, FHIT, HS1BP3, AIFM1, DTYMK, NRGN, DIABLO, RNF5, SIRT2, TIMM8A, GLOD4, NDUFB7, SOD1, GADD45GIP1, BSG, CASP2, CNP, NFKB1, TST, ARL2BP, PARK7, PRDX3, ARAF, MTHFD2, MSRA, FKBP4, ACADSB, LACTB2, RANBP2, CYB5A, SNAP23, LONP1

Mitochondrion	246	Cellular Components	NIH	1.315530757	0.001555491	0.04754108	MYOM2, CHCHD10, PTGES2, NOS1, TRIAP1, IMMT, NGRN, NDUFA5, COX6B1, COQ7, MAP2K1, FIS1, HIP1R, THOP1, HSPA1A, ERN1, ACSL1, EFHD1, RIDA, TNFRSF1A, NDUFS6, MRPL24, ARG1, IVD, ABL1, PTS, AK2, TIMM8A, OXCT1, FKBP4, SIRT2, PRDX5, MTUS1, FXN, STK11, CASP8, PARK7, BOLA1, DTYMK, ATXN3, PRDX3, GLOD4, SUOX, FOXO3, DNM3, LGALS3, METAP1D, BECN1, SNCA, MAPK9, TOMM20, NME1, SOD1, ALDH2, GSR, GIT1, GRPEL1,<br><br>VASN, CA5A, C1QBP, NFU1, HK2, RNASET2, YWHAQ, HMGCL, TRAF3, CAT, SORD, DDX1, MRPL52, DYNLT1, UROS, GABARAPL1, PRDX6, BCL2, PECR, BTD, DUT, HAGH, MRPS16, GADD45GIP1, ARL2BP, MECR, SIRT5, NMT1, APEX1, PLA2G2A, SDHB, CCAR2, BNIP3L, TIGAR, NIT1, BCL2L1, UNG, CWC15, MSRA, MAPT, PLA2G4A, FOXO1, ACP6, TXNRD1, HARS1, MAVS, SLIRP, C19orf12, LETM1, ALDH5A1, PACS2, CNP, LACTB2, FGR, ASS1, NFKB1, SIRT1,<br><br>AGXT, AKR1B10, ATP5IF1, BDNF
Cardiac Muscle Contraction	15	KEGG	UCL	1.952554413	0.001589335	0.022485477	MYL3, TPM3, TNNI3, MYL4, CACNB1
Envelope	165	Cellular Components	NIH	1.354950871	0.001868427	0.04754108	CHCHD10, TRIAP1, IMMT, MAD1L1, NGRN, LMNB2, NDUFA5, COX6B1, COQ7, FIS1, THOP1, ERN1, ACSL1, EFHD1, GAPDH, NDUFS6, MRPL24, NUP50, ARG1, IVD, ABL1, AK2, TIMM8A, DCTN1, CASP8, PARK7, DTYMK, ATXN3, GCHFR, SUOX,

							FOXO3, DNM3, LGALS3, SORT1, SHISA5, CLGN, BECN1, NUCB2, GHRHR, TOMM20, SMAD1, NME1, TPR, SOD1, GRPEL1, HK2, RNASET2, BCHE, CAT, DNAJB2, SORD, MRPL52, IL15RA, CPTP, BCL2, RANBP1, MRPS16,
Protein Modification By Small Protein Conjugation Or Removal	123	Biological Process	UCL	-1.548179109	0.002022619	0.038115563	ARL2BP, SIRT5, SCGB1A1, PLA2G2A, SDHB, INSR, BNIP3L, TIGAR, BCL2L1 TRIM21, TRIM58, WWP2, TNIP1, NUB1, NEDD4L, AXIN1, TRIM5, SPRY2, TRIM25, MINDY1, ABRAXAS2, ABL1, HGS, TRIM24, USP25, NFX1, BRAP, UFD1, BECN1, ATXN3, OTUD7B, IKBKG, ZNRF4, DDA1, RNF41, STAMBP, RAD23B, UBXN1, RNF5, XIAP, MYCBP2, PSME1, ZBTB16, MED18, COMMD1, PARK7, OTUD6B, ELOB, GATA3, YOD1, SEL1L, RANBP2, TRAF2, TADA3, DDX58, AMFR, USP8, CDC26, DNAJB2, HIF1A, CDC25A, TRIM40, PSMD5, UBE2L6, BIRC2, PTEN, CDC27, HSPA1A
Envelope	164	Cellular Components	UCL	-1.396699249	0.007014311	0.044467342	DCTN1, PPP1CC, IRAG2, PLSCR3, LYN, MAVS, ANXA11, ABL1, CETN3, IST1, WASF1, SRC, BID, RTN4IP1, PTPN1, DNM1, BRAP, CASP8, CIAPIN1, ITPRIP, ATP1B4, BECN1, ATXN3, RANBP1, SPART, ANXA4, ERBIN, PLA2G4A, DNAJB14, AK2, CACYBP, BCL2L1, NUCB2, AIFM1, DTYMK, NRGN, DIABLO, RNF5, CETN2, MTDH, TIMM8A, CASC3, NDUFB7, SOD1, CNP, ARL2BP, PARK7

Supplemental Table 3. AR113Q vs WT - Differential Abundance Results

Gene	Age	Log2FoldChange	se	t	P
Eda2r	20 Weeks	0.992329238	0.102637835	9.668259677	9.19326562642196e-15
Eda2r	14 Weeks	0.7656205	0.085835619	8.919612936	1.71333846203195e-13
Eda2r	22 Weeks	1.019337011	0.122135053	8.345982445	3.46422463411184e-12
Eda2r	16 Weeks	0.839389259	0.103622153	8.100480771	7.0690535841972e-12
Eda2r	28 Weeks	0.720027764	0.094589518	7.612130613	2.74253479497188e-10
Eda2r	12 Weeks	0.543293167	0.081856374	6.637151645	3.82927954331737e-09
Eda2r	30 Weeks	0.942197333	0.144855989	6.504372633	3.57859402606644e-08
Eda2r	24 Weeks	1.039592761	0.170976756	6.080316336	6.42841528290901e-08
Eda2r	26 Weeks	0.79155466	0.131297776	6.02869817	1.04589839734416e-07
Wfikkn2	30 Weeks	0.385036556	0.065656375	5.864419926	3.55288518988241e-07
Cdh6	30 Weeks	0.593666333	0.109405839	5.426276481	1.67980272169012e-06
Wfikkn2	20 Weeks	0.287757303	0.060720488	4.73904795	1.01422300543406e-05
Wfikkn2	26 Weeks	0.305632415	0.065411891	4.672428997	1.68693083670632e-05
Fst	12 Weeks	-0.455708667	0.105133177	-4.334584765	4.31307689169981e-05
Cntn4	30 Weeks	0.354087556	0.080605811	4.392829119	5.81809433824554e-05
Wfikkn2	14 Weeks	0.221205	0.05482543	4.034715286	0.000127818
Vsig2	30 Weeks	0.609119667	0.147742685	4.122841458	0.000140918
Wisp1	30 Weeks	0.378274778	0.092350758	4.096065749	0.000153654
Wfikkn2	24 Weeks	0.29175102	0.076076616	3.834963164	0.000280144
Wfikkn2	22 Weeks	0.269098908	0.070640753	3.809400354	0.000290612
Cdh6	20 Weeks	0.349030771	0.092194287	3.785817791	0.000309138
Fas	14 Weeks	0.1853825	0.051885305	3.572928766	0.000612946
Wfikkn2	28 Weeks	0.207598374	0.057669157	3.599816353	0.000660485
Mia	12 Weeks	0.182567	0.052548449	3.474260471	0.000839374
Tnfrsf12a	24 Weeks	0.63688107	0.184362561	3.454503278	0.000960747
Mia	14 Weeks	0.208334667	0.061014987	3.414483514	0.001022322
Erbb4	12 Weeks	-0.193990167	0.05811886	-3.337817824	0.001296115
Cdh6	24 Weeks	0.38883393	0.116113558	3.348738391	0.001335643

Cdh6	26 Weeks	0.384596803	0.115277891	3.33625814	0.0014496
Cxcl9	26 Weeks	0.615578741	0.187514897	3.282825795	0.001703726
Cyr61	28 Weeks	-0.237460163	0.072450389	-3.277555373	0.001771772
Ntf3	12 Weeks	-0.230434	0.071425815	-3.226200493	0.00183434
Gdnf	30 Weeks	0.383908222	0.117441027	3.268944691	0.001956712
Ghrl	24 Weeks	0.338259726	0.106424516	3.178400434	0.002241667
Yes1	26 Weeks	0.496561395	0.1557158	3.188895374	0.002254995
Cxcl9	22 Weeks	0.54180023	0.172480553	3.141225027	0.002441527
Notch3	30 Weeks	0.164299444	0.051572142	3.185817759	0.002487267
Erbb4	16 Weeks	-0.225707677	0.072234228	-3.124663791	0.002520335
Cxcl9	30 Weeks	0.754256778	0.237729629	3.172750409	0.002582102
Cpe	14 Weeks	0.1575525	0.050627486	3.111995311	0.002607309
Ghrl	30 Weeks	0.462882778	0.149457058	3.097095473	0.00320155
Tnfrsf12a	22 Weeks	0.434103276	0.144421062	3.005816948	0.003644203
Cdh6	28 Weeks	0.333139106	0.110308615	3.020064272	0.003754013
Erbb4	28 Weeks	-0.305620488	0.101300187	-3.016978529	0.00378713
Ntf3	14 Weeks	-0.170319	0.057298895	-2.972465717	0.003941219
Il1a	26 Weeks	0.784333027	0.266559683	2.942429317	0.004599117
Clstn2	28 Weeks	0.279125122	0.096358403	2.896738786	0.005310781
S100a4	30 Weeks	0.328131222	0.113281929	2.896589298	0.005583278
Dll1	14 Weeks	0.190811333	0.067466234	2.828249368	0.005963265
Cxcl9	14 Weeks	0.469969833	0.166252333	2.82684655	0.005986942
Nadk	16 Weeks	-0.490586869	0.173831244	-2.822201915	0.006083989
S100a4	20 Weeks	0.233804023	0.083240967	2.808761488	0.006356892
Cxcl9	28 Weeks	0.570982439	0.205446705	2.779224122	0.007332265
Nadk	26 Weeks	0.535306224	0.19299833	2.773631387	0.007344784
Ahr	16 Weeks	-0.224963165	0.082392134	-2.73039615	0.007858542
Ca13	14 Weeks	-0.3261335	0.120297668	-2.711054217	0.008266613
Erbb4	14 Weeks	-0.178011333	0.066026641	-2.696053159	0.0086139
Il23r	14 Weeks	0.279995167	0.104962459	2.667574374	0.009310016
Foxo1	16 Weeks	-0.171015051	0.064197736	-2.663879783	0.009427011

Cdh6	14 Weeks	0.2414175	0.091062574	2.651116594	0.00973526
Itgb6	30 Weeks	0.292754333	0.109204678	2.680785655	0.009922363
Vsig2	28 Weeks	0.355640325	0.133860087	2.656806322	0.010172556
Tnfrsf11b	16 Weeks	-0.257768316	0.098731539	-2.610800157	0.010877374
Clmp	30 Weeks	0.193876556	0.074609502	2.598550455	0.012269842
Cdh6	12 Weeks	0.197242167	0.077024781	2.560762446	0.012374303
Csf2	16 Weeks	-0.145412071	0.057045883	-2.549037068	0.012817584
Kitlg	20 Weeks	-0.368417545	0.144869631	-2.54309715	0.013076503
Tnfrsf12a	30 Weeks	0.339496778	0.13252342	2.561787016	0.013474922
Vsig2	20 Weeks	0.309811559	0.123626935	2.506019907	0.014407498
Cntn4	28 Weeks	0.191804228	0.076101934	2.520359429	0.014497313
Wisp1	24 Weeks	0.283179826	0.113919159	2.485796307	0.015427213
Tnfrsf12a	20 Weeks	0.255209427	0.103017606	2.477337971	0.015519441
Cyr61	22 Weeks	-0.213063851	0.086017465	-2.476983612	0.015598949
Notch3	14 Weeks	0.097819	0.039554885	2.472994141	0.015602522
Tgfb1	12 Weeks	0.177525167	0.072103142	2.462100296	0.016018466
Clmp	14 Weeks	0.110316667	0.044996546	2.451669647	0.016484935
Il23r	30 Weeks	0.262269667	0.107082958	2.449219486	0.017864171
Tgfbr3	28 Weeks	-0.140362642	0.058295019	-2.407798219	0.019250007
Foxo1	26 Weeks	0.238152687	0.099202521	2.400671718	0.019430193
Dctn2	24 Weeks	0.343686219	0.143952352	2.387499852	0.019791227
Dctn2	30 Weeks	0.448775444	0.186921638	2.400874771	0.020117487
Cxcl9	20 Weeks	0.444897652	0.187687573	2.370416136	0.020374443
Riox2	26 Weeks	0.462463401	0.194769037	2.374419515	0.020738249
Tnfrsf12a	16 Weeks	0.266533872	0.112973719	2.359255543	0.020881174
Hgf	26 Weeks	0.434625034	0.183666839	2.366377269	0.021154417
Clstn2	24 Weeks	0.208664726	0.089199444	2.339305247	0.022309504
Fli1	22 Weeks	0.196665977	0.084232971	2.334786173	0.022346757
Tnfrsf12a	14 Weeks	0.265627	0.114295101	2.324045374	0.022762029
Flrt2	24 Weeks	-0.192833035	0.083154337	-2.31897749	0.023454472

Ntf3	16 Weeks	-0.176716498	0.077109372	-2.291764191	0.024689481
Il23r	12 Weeks	0.248600833	0.109050911	2.279676809	0.025359776
Yes1	30 Weeks	0.554523444	0.24328111	2.279352655	0.026948579
Cdh6	22 Weeks	0.214841839	0.095201306	2.25671105	0.027062135
Erbb4	20 Weeks	-0.1552394	0.068885883	-2.2535735	0.027185133
Hgf	22 Weeks	0.369015747	0.163989962	2.250233745	0.027490167
Dctn2	22 Weeks	0.288369368	0.128923142	2.236754106	0.028400082
Mia	16 Weeks	0.165671785	0.074191035	2.233043179	0.028489221
Fas	12 Weeks	0.130522	0.058710503	2.223145663	0.029100711
Wisp1	22 Weeks	0.194372011	0.087759537	2.214824934	0.029937014
Plin1	26 Weeks	0.592695	0.266821301	2.221318152	0.030051235
Cxcl9	12 Weeks	0.428864667	0.194603322	2.203789037	0.030488589
Qdpr	30 Weeks	0.744914556	0.335258461	2.221911277	0.030843914
Parp1	22 Weeks	0.457714598	0.208196265	2.198476508	0.031129843
Foxo1	14 Weeks	-0.151446333	0.069292519	-2.185608698	0.031883993
Mia	20 Weeks	0.180226183	0.082940598	2.172954944	0.032985212
Il23r	16 Weeks	0.23676904	0.109115095	2.169901791	0.033139214
Epo	24 Weeks	-0.548883035	0.252972748	-2.169731876	0.033577873
Ccl2	12 Weeks	0.603457833	0.280176752	2.153846913	0.03434021
Fst	16 Weeks	-0.194413283	0.091050293	-2.135229631	0.035964315
Clstn2	26 Weeks	0.217411769	0.101381334	2.144495057	0.035985771
Mia	24 Weeks	0.164502512	0.077006847	2.136206322	0.036319391
Prdx5	30 Weeks	0.718998111	0.334718888	2.148065549	0.036578178
Qdpr	26 Weeks	0.349862823	0.163883186	2.13483049	0.036800546
Igsf3	30 Weeks	0.243283111	0.113527561	2.142943169	0.037008624
Ccl5	22 Weeks	0.320179195	0.150823727	2.122870205	0.037203583
Il17f	14 Weeks	-0.1356005	0.064155584	-2.113619592	0.037786135
Lgmn	14 Weeks	0.133444333	0.063206287	2.111250954	0.03799547
Tgfb1	14 Weeks	0.154234167	0.073480019	2.098994636	0.039094788
Wisp1	26 Weeks	0.156973946	0.074779906	2.099146077	0.039949973
Il17f	28 Weeks	0.113477764	0.054004293	2.101273017	0.039972411

Dlk1	28 Weeks	-0.174245488	0.083099999	-2.096816974	0.040380702
Fstl3	24 Weeks	0.107565672	0.05179447	2.076779073	0.041660098
Fas	20 Weeks	0.139248898	0.067542068	2.061661739	0.042752511
Flrt2	12 Weeks	-0.123563	0.060162697	-2.053814191	0.043344502
S100a4	12 Weeks	0.142156833	0.069755733	2.037923293	0.044947822
Fli1	16 Weeks	-0.174037862	0.085580577	-2.033614018	0.045481388
Notch3	20 Weeks	0.107381559	0.052935335	2.028542156	0.046103789
Parp1	26 Weeks	0.408923844	0.200952436	2.034928524	0.04621031
Riox2	22 Weeks	0.307728276	0.152289251	2.020682836	0.047032598
Fas	30 Weeks	0.278536889	0.13765225	2.023482285	0.04838509
Wisp1	20 Weeks	0.132041631	0.066104222	1.997476505	0.049449964
Ghrl	20 Weeks	0.23305793	0.116684326	1.997337079	0.049465439
Rgma	14 Weeks	0.087973333	0.044524643	1.975834682	0.051756117
Igsf3	20 Weeks	0.16896336	0.085514229	1.975850818	0.05190023
Cpe	30 Weeks	0.145809222	0.073810933	1.975442057	0.053750766
Clstn2	14 Weeks	0.1523835	0.077938111	1.955185967	0.054189945
Ddah1	30 Weeks	0.394561556	0.200220241	1.9706377	0.054314519
Ghrl	12 Weeks	0.235703333	0.120749765	1.951998281	0.054527408
Tgfa	20 Weeks	0.199190556	0.102134263	1.950281418	0.054930336
Tgfbr3	16 Weeks	-0.105962003	0.054378159	-1.948613307	0.055032944
Ccl2	30 Weeks	0.698739556	0.356830347	1.958184221	0.055799545
Csf2	22 Weeks	0.175525115	0.090318775	1.943395658	0.055877708
Tnr	16 Weeks	-0.110812205	0.057349456	-1.932227677	0.057058229
Cant1	30 Weeks	0.136152778	0.069908835	1.94757613	0.057091865
Cyr61	12 Weeks	-0.159753833	0.082765385	-1.930201043	0.057217044
Map2k6	20 Weeks	-0.329494812	0.170644013	-1.930889961	0.05732752
Itgb6	26 Weeks	0.22687551	0.117523602	1.930467638	0.058201066
Prdx5	14 Weeks	-0.124364167	0.065025359	-1.912548699	0.059524714
Dlk1	26 Weeks	-0.180898776	0.094493955	-1.914395212	0.06026327
Il17f	22 Weeks	0.133573276	0.07050724	1.894461857	0.062179989
Ahr	14 Weeks	-0.161151333	0.085250803	-1.89032042	0.062477845

Cntn1	30 Weeks	0.151565667	0.080011794	1.89429157	0.063977676
Gdnf	14 Weeks	-0.1319465	0.070512912	-1.87123885	0.065110575
Gfra1	14 Weeks	0.273851167	0.14646332	1.869759383	0.065318541
Qdpr	22 Weeks	0.293656782	0.157329876	1.866503611	0.066043187
Fas	26 Weeks	0.162843537	0.087244575	1.86651763	0.066777552
Fstl3	14 Weeks	0.084370333	0.045380814	1.859163079	0.066824431
Tgfbr3	12 Weeks	-0.087050333	0.047081881	-1.848913682	0.068259343
Kitlg	26 Weeks	0.114813367	0.061966713	1.852823256	0.068746687
Fstl3	20 Weeks	0.080617616	0.044138393	1.82647375	0.071812158
Il5	14 Weeks	0.203681667	0.111743473	1.822761199	0.07222139
Casp3	28 Weeks	-0.518652805	0.284878396	-1.820611222	0.073826092
Dll1	20 Weeks	0.130643656	0.07243963	1.803483208	0.075382706
Gfra1	26 Weeks	0.303019286	0.168152562	1.802049765	0.07647907
Hgf	30 Weeks	0.483677889	0.268007597	1.804717083	0.077143033
Eno2	28 Weeks	-0.125401098	0.070385084	-1.781643084	0.080043019
Ppp1r2	14 Weeks	-0.157450333	0.089526591	-1.758699086	0.082601692
Wfikkn2	16 Weeks	0.112414108	0.064132795	1.752833449	0.083663258
Gdnf	28 Weeks	0.152705285	0.086831382	1.758641647	0.083913371
Cpe	26 Weeks	0.149910102	0.085345771	1.756502986	0.084021372
Notch3	28 Weeks	0.077001504	0.043808541	1.757682459	0.084078089
Matn2	22 Weeks	0.28016477	0.159951977	1.751555527	0.084108635
Cpe	22 Weeks	0.113738678	0.065060808	1.748190384	0.084693729
Il1b	24 Weeks	0.54124806	0.309473765	1.748930348	0.084882836
Ahr	26 Weeks	0.235621939	0.134782733	1.748161162	0.085467517
Axin1	26 Weeks	0.238612585	0.136931717	1.742566223	0.086448965
Cyr61	24 Weeks	-0.200986791	0.115709795	-1.73699029	0.086984418
Casp3	24 Weeks	-0.320664303	0.184940025	-1.733882665	0.087538383
Ccl5	30 Weeks	0.240378444	0.138007992	1.741771921	0.087697771
Prdx5	24 Weeks	0.66491194	0.383884276	1.732063494	0.087864016
Lgmn	26 Weeks	0.128017517	0.07392447	1.731733987	0.088375635
Vsig2	22 Weeks	0.245690402	0.142369888	1.725718867	0.088687539

Ppp1r2	26 Weeks	0.204987347	0.11852296	1.729515931	0.088774492
Ddah1	14 Weeks	-0.1004255	0.058272533	-1.723376259	0.088834045
Gcg	28 Weeks	-0.381980691	0.220939501	-1.7288927	0.089148372
Vsig2	14 Weeks	0.167334667	0.097531289	1.715702401	0.090237893
Dctn2	26 Weeks	0.306798197	0.1782266	1.721393985	0.090247708
Dll1	12 Weeks	0.123564167	0.072080351	1.714255886	0.090452994
Wisp1	14 Weeks	0.103611167	0.060652803	1.708266741	0.091615477
Cyr61	16 Weeks	-0.138072458	0.080964788	-1.705339587	0.092215502
Ahr	30 Weeks	0.298846556	0.175667373	1.70120695	0.095116008
Ca13	16 Weeks	-0.185317626	0.109760417	-1.688383035	0.095437286
Il23r	20 Weeks	0.173798324	0.103401549	1.680809675	0.097016509
Il6	12 Weeks	0.194269333	0.117349946	1.655470151	0.101847434
Dlk1	22 Weeks	-0.164169828	0.099152259	-1.655734613	0.102128078
Ghrl	22 Weeks	0.188091494	0.11425286	1.646273833	0.104065922
Apbb1ip	16 Weeks	-0.178374697	0.108447829	-1.644797313	0.104141904
Il1b	22 Weeks	0.338142241	0.205752643	1.643440569	0.104652027
Apbb1ip	30 Weeks	0.280529222	0.169752289	1.652579904	0.104683129
Itgb6	20 Weeks	0.13240888	0.081226273	1.630123796	0.107324892
Rgma	30 Weeks	0.098770778	0.060330957	1.637149189	0.107878741
Ccl2	22 Weeks	0.394744655	0.24267224	1.626657648	0.108178909
Notch3	26 Weeks	0.108428231	0.066548737	1.629305621	0.108402243
Clstn2	30 Weeks	0.161267556	0.099436446	1.621815358	0.111132658
Lpl	30 Weeks	-0.307341111	0.190835144	-1.610505822	0.113583384
Fas	22 Weeks	0.192104483	0.120158305	1.598761587	0.114253112
Erbb4	22 Weeks	-0.154156897	0.096490204	-1.597642977	0.114502287
Lpl	12 Weeks	-0.205512667	0.128890746	-1.594471849	0.114874698
Parp1	30 Weeks	0.682371222	0.42587215	1.602291255	0.115390795
Tgfbr3	24 Weeks	-0.099595398	0.062441616	-1.595016345	0.115415289
Igsf3	26 Weeks	0.151796224	0.095308517	1.592682677	0.116400594
Il17f	20 Weeks	0.088714203	0.055890546	1.587284591	0.116712255
Casp3	14 Weeks	-0.321865	0.203204939	-1.583942799	0.11730557

Tpp1	30 Weeks	-0.187883111	0.118213359	-1.589355998	0.118284035
Pak4	16 Weeks	-0.173729731	0.110192833	-1.57659737	0.1190419
Fas	16 Weeks	0.097365707	0.061864329	1.573858616	0.119674137
Cxcl9	16 Weeks	0.243162912	0.155118759	1.567591911	0.12113089
Flrt2	16 Weeks	-0.10978202	0.070620241	-1.554540439	0.124210223
Axin1	16 Weeks	-0.151621229	0.097596705	-1.553548645	0.124446748
Gfra1	24 Weeks	0.249592761	0.160495239	1.555141216	0.124623773
Lgmn	30 Weeks	0.125469778	0.080462754	1.559352269	0.125220811
S100a4	26 Weeks	0.134886803	0.08691897	1.551868401	0.125866949
Pak4	22 Weeks	0.1869	0.12085042	1.546539933	0.126358646
Dlk1	24 Weeks	-0.165111891	0.106864859	-1.545053181	0.127043989
Vsig2	26 Weeks	0.231505238	0.149983889	1.543534043	0.127873607
Riox2	30 Weeks	0.582600333	0.376947091	1.545575883	0.128513512
Plin1	20 Weeks	-0.466844131	0.303875902	-1.536298627	0.128730105
Kitlg	30 Weeks	0.091514556	0.059254809	1.544424105	0.128791911
Cpe	20 Weeks	0.115518396	0.075293229	1.534246807	0.129233554
Foxo1	30 Weeks	0.254263	0.165040427	1.540610411	0.129717174
Cyr61	14 Weeks	-0.119132167	0.077861415	-1.530053967	0.130101475
Igsf3	24 Weeks	0.146482662	0.095615219	1.53200153	0.130230529
Itgb6	24 Weeks	0.189831517	0.124277129	1.52748554	0.131347755
Fstl3	30 Weeks	0.113666111	0.074110549	1.533737263	0.131398128
Epcam	22 Weeks	-0.145220287	0.095420929	-1.521891353	0.132416316
Ghrl	26 Weeks	0.204371054	0.134062048	1.524451237	0.132564172
Lpl	16 Weeks	-0.183800791	0.12089778	-1.520299142	0.132585468
Lgmn	24 Weeks	0.099977886	0.065798833	1.519447713	0.133355044
Cxcl1	22 Weeks	0.414238678	0.273600333	1.514028414	0.134396321
Tnr	28 Weeks	-0.11077752	0.07323427	-1.512645919	0.135799818
Gfra1	22 Weeks	0.24182092	0.160383195	1.507769683	0.135989008
Matn2	14 Weeks	0.154242	0.102975772	1.497847472	0.138261294
Lpl	14 Weeks	-0.1604795	0.107354055	-1.494862025	0.139037614
Tnr	22 Weeks	-0.118591897	0.07950939	-1.491545802	0.140186863

Mia	30	Weeks	0.184997333	0.123494141	1.498025184	0.140413868
Wisp1	28	Weeks	0.114537764	0.076912358	1.489198452	0.141852961
Riox2	16	Weeks	-0.245694259	0.166252009	-1.477842346	0.143582384
Il17a	12	Weeks	0.532739833	0.36096197	1.475889088	0.143999346
Pdgfb	20	Weeks	-0.496312357	0.337081281	-1.472381838	0.145158365
Adam23	30	Weeks	0.167770444	0.113503671	1.478105889	0.145651646
Ccl5	12	Weeks	0.1157825	0.079725729	1.452260153	0.150440142
Cntn4	20	Weeks	0.125595529	0.086524047	1.451567899	0.150848274
Fstl3	22	Weeks	0.079970517	0.055293205	1.446299186	0.152432663
Il5	26	Weeks	0.226512143	0.157317123	1.439844169	0.155024067
Tgfa	14	Weeks	0.129046833	0.09007459	1.432666341	0.156000727
Tnfrsf12a	26	Weeks	0.188833605	0.131664836	1.434199225	0.156622146
Il6	16	Weeks	-0.201211448	0.14090049	-1.428039371	0.157377099
Itgb1bp2	12	Weeks	-0.163838	0.114891138	-1.426028182	0.157850304
Clstn2	20	Weeks	0.13331457	0.093835124	1.420732075	0.159594755
Parp1	16	Weeks	-0.195343788	0.137958086	-1.41596476	0.160871188
Plxna4	30	Weeks	-0.413169444	0.290686999	-1.421355087	0.161420986
Matn2	20	Weeks	0.173267661	0.122824995	1.410687308	0.162526911
Il17f	16	Weeks	-0.088188805	0.062776005	-1.404817093	0.164149827
Tnr	12	Weeks	-0.089759333	0.063973416	-1.403072385	0.164563483
Csf2	20	Weeks	0.085150448	0.060676833	1.403343652	0.164696704
Gfra1	28	Weeks	0.237548374	0.169330625	1.402867165	0.165987013
Map2k6	16	Weeks	-0.211763603	0.151767836	-1.395312794	0.166985473
Il1b	30	Weeks	0.329319111	0.23664752	1.391601782	0.170203472
Map2k6	26	Weeks	0.318151939	0.229249859	1.38779557	0.170247927
Igsf3	28	Weeks	0.156938821	0.113293595	1.385240018	0.171283819
Prdx5	16	Weeks	-0.126442896	0.091810675	-1.377213433	0.1724891
Apbb1ip	22	Weeks	0.177016724	0.128547193	1.377056312	0.172761286
Fas	24	Weeks	0.247754378	0.179828041	1.377729395	0.172871438
Yes1	22	Weeks	0.181347989	0.131951004	1.374358532	0.173593331
Tnf	24	Weeks	0.2991199	0.218855908	1.366743547	0.176274592

Cntn4	26	Weeks	0.147637687	0.108502767	1.360681305	0.178620907
Dlk1	16	Weeks	-0.116940539	0.086321347	-1.3547117	0.17952302
Cxcl9	24	Weeks	0.266558881	0.196995787	1.353119702	0.180565578
Il10	24	Weeks	0.381318582	0.281919532	1.352579507	0.180737339
Acvrl1	12	Weeks	0.139698333	0.103558895	1.348974737	0.181248321
Cyr61	26	Weeks	-0.146567347	0.108579549	-1.349861443	0.182048437
Plxna4	24	Weeks	-0.264775572	0.19819869	-1.335909799	0.18609885
Ppp1r2	12	Weeks	-0.128113667	0.096419639	-1.328709256	0.187818036
Cdh6	16	Weeks	0.141306229	0.106467699	1.327221587	0.188408824
Ccl2	26	Weeks	0.339713571	0.256398319	1.324944613	0.19013143
Gdnf	24	Weeks	0.118704328	0.089818384	1.321603926	0.190795188
Gfra1	20	Weeks	0.205640636	0.156587497	1.313263447	0.1931518
Fli1	20	Weeks	-0.114356577	0.087263507	-1.310474228	0.194088353
Dctn2	14	Weeks	0.136991333	0.104758466	1.307687472	0.194870292
Il23r	26	Weeks	0.131122041	0.100072719	1.310267592	0.195017797
Tgfbr3	14	Weeks	-0.066077167	0.050586646	-1.306217593	0.195367098
Pak4	20	Weeks	-0.170534704	0.130672286	-1.305056405	0.195917241
Lgmn	22	Weeks	0.090458678	0.070289407	1.286946099	0.202235256
Itgb1bp2	16	Weeks	-0.16063069	0.125646655	-1.278431889	0.204987633
Tnf	28	Weeks	0.142153293	0.111065396	1.279906231	0.205673943
Cxcl1	28	Weeks	0.289078699	0.226216271	1.277886411	0.206380418
Ntf3	20	Weeks	-0.097293683	0.076353443	-1.274254037	0.206560621
Itgb6	22	Weeks	0.145257989	0.113978886	1.274428917	0.206609361
Pdgfb	16	Weeks	-0.400622273	0.314538445	-1.273683007	0.206657372
Tgfa	26	Weeks	0.135321769	0.106343476	1.27249714	0.20802565
Gdnf	12	Weeks	0.100033167	0.079062519	1.265241336	0.20955157
Prdx5	22	Weeks	0.34285477	0.272183999	1.259643373	0.21186601
Tgfb1	20	Weeks	0.065795645	0.052448238	1.254487243	0.213612911
Il1b	14	Weeks	-0.082498167	0.065830034	-1.253199508	0.213925244
Ppp1r2	30	Weeks	0.161802556	0.128656225	1.257634871	0.214366075
Lgmn	20	Weeks	0.076783665	0.061311261	1.252358271	0.214382925

Ntf3	26 Weeks	-0.130088946	0.104062785	-1.250100561	0.216037463
S100a4	14 Weeks	0.0836505	0.067124783	1.246193965	0.216471356
Tnf	20 Weeks	0.122118701	0.097965476	1.24654833	0.216494673
Ntf3	28 Weeks	-0.101623252	0.081660086	-1.244466632	0.218333325
Tgfb1	22 Weeks	0.082916034	0.066906775	1.239277112	0.219267398
Cntn1	20 Weeks	0.095844642	0.077685214	1.233756546	0.221197882
Pak4	12 Weeks	0.141189833	0.114703161	1.230914929	0.222053534
Wisp1	12 Weeks	0.069996833	0.057037634	1.227204374	0.223436902
Matn2	24 Weeks	0.189792488	0.15455213	1.228015993	0.223737616
Dll1	30 Weeks	0.109870111	0.089509571	1.22746774	0.225394656
Epo	30 Weeks	-0.442052889	0.361724076	-1.222072067	0.227410478
Cyr61	20 Weeks	-0.080673835	0.066283069	-1.217110742	0.227429418
Vsig2	12 Weeks	0.1302885	0.107176192	1.215647785	0.227785669
Itgb6	16 Weeks	-0.104056801	0.085739158	-1.213643851	0.228641998
Tgfbr3	22 Weeks	-0.082981782	0.068544278	-1.210630331	0.229996261
Rgma	20 Weeks	0.057955125	0.04799382	1.207553923	0.231064318
Tpp1	16 Weeks	-0.091501835	0.075797493	-1.207188145	0.23110346
Snap29	30 Weeks	0.398938556	0.329178695	1.211920946	0.231238758
Lpl	22 Weeks	-0.155371379	0.128721588	-1.207034357	0.231369516
Erbb4	26 Weeks	-0.13536602	0.112223297	-1.206220311	0.232390104
Ccl3	22 Weeks	0.179031149	0.149447893	1.197950311	0.234865049
Casp3	12 Weeks	-0.2610935	0.219205852	-1.191088185	0.237230925
Ddah1	16 Weeks	-0.0763167	0.064205568	-1.188630553	0.238285966
Prdx5	28 Weeks	0.32835061	0.275574718	1.191512098	0.238305558
Pla2g4a	14 Weeks	0.143523667	0.121008796	1.186059788	0.239245675
Il23r	28 Weeks	0.128220081	0.108031533	1.186876442	0.240115008
Matn2	26 Weeks	0.166769184	0.140753013	1.184835622	0.240678151
Ghrl	14 Weeks	0.1388025	0.117443305	1.181868134	0.240895147
Casp3	16 Weeks	-0.259272189	0.219525755	-1.181055904	0.241263374
Vegfd	14 Weeks	0.064642	0.054738019	1.180934214	0.241263766
Erbb4	24 Weeks	-0.133783831	0.113310688	-1.180681485	0.241903833

Matn2	30 Weeks	0.202591	0.171568442	1.180817388	0.243261985
Il6	28 Weeks	0.237446138	0.20296583	1.169882328	0.246833293
Dlk1	12 Weeks	-0.072518167	0.062286878	-1.164260745	0.247867126
Epo	26 Weeks	-0.381299932	0.32879366	-1.159693686	0.250692735
Il6	20 Weeks	0.270669722	0.234596033	1.153769391	0.25230755
Hgf	28 Weeks	0.242325894	0.210646217	1.150392813	0.254703157
Pdgfb	12 Weeks	-0.372676	0.325780789	-1.143947135	0.256144114
Snap29	16 Weeks	-0.164025606	0.143518258	-1.142890168	0.256671753
Fst	20 Weeks	-0.100490412	0.087922246	-1.142946378	0.256745171
Wfikkn2	12 Weeks	0.0689845	0.06039666	1.142190647	0.256868901
Il17a	14 Weeks	0.432714333	0.379701136	1.139618222	0.257978123
Il5	28 Weeks	0.249243333	0.218215992	1.142186378	0.258069944
Nadk	30 Weeks	0.438663778	0.384168662	1.141852058	0.258954487
Matn2	28 Weeks	0.142187724	0.124753251	1.139751653	0.259074882
Il1a	12 Weeks	0.294515	0.259302262	1.135798036	0.259518956
Gfra1	16 Weeks	0.187305135	0.16540691	1.132390022	0.261030578
Clmp	20 Weeks	0.079332464	0.070150336	1.130892154	0.261752367
Map2k6	30 Weeks	0.292808667	0.258476049	1.132827074	0.262690018
Epo	12 Weeks	0.345194333	0.306587698	1.125923629	0.263650214
Ccl2	16 Weeks	-0.322866532	0.286840702	-1.125595251	0.2638789
Yes1	24 Weeks	0.184596766	0.164578974	1.121630316	0.266023512
Snap29	26 Weeks	0.269839082	0.241384191	1.117882164	0.268000679
Vegfd	12 Weeks	-0.061674167	0.055468463	-1.111878063	0.269605933
Snap29	20 Weeks	-0.17340328	0.156254846	-1.109746572	0.270701548
Acvrl1	16 Weeks	0.111296279	0.101063338	1.101252756	0.274262255
Il6	24 Weeks	0.511214303	0.465268679	1.098750737	0.275809504
Crim1	16 Weeks	-0.091214714	0.083513511	-1.092215053	0.278188842
Snap29	22 Weeks	0.189712989	0.173746603	1.091894664	0.27851968
Flrt2	28 Weeks	-0.067678618	0.062076094	-1.090252517	0.280110805
Cxcl1	30 Weeks	0.347994889	0.319428369	1.089430128	0.281187198
Dctn2	20 Weeks	0.100007742	0.092519872	1.080932556	0.283237308

Tnf	14 Weeks	0.104653667	0.097027341	1.078599754	0.284133471
Plxna4	12 Weeks	-0.2639635	0.245638963	-1.074599474	0.285867085
Pdgfb	26 Weeks	0.444603537	0.415720584	1.069476842	0.289068649
Vegfd	16 Weeks	-0.067678232	0.063579663	-1.064463525	0.290489088
Lgmn	16 Weeks	-0.06203867	0.058473534	-1.060970085	0.292063531
Cyr61	30 Weeks	-0.098286889	0.092466372	-1.062947387	0.292912773
Eno2	30 Weeks	0.067308778	0.063422022	1.061284013	0.293660382
Ddah1	22 Weeks	0.068909713	0.065508324	1.051922991	0.296352575
Cant1	20 Weeks	0.053980654	0.051608833	1.045957666	0.298985175
Il1a	30 Weeks	0.196794556	0.187796016	1.047916561	0.299716305
Cntn4	14 Weeks	0.061608333	0.059046407	1.043388357	0.300033219
Dll1	24 Weeks	0.091608582	0.087725823	1.044260166	0.300118169
Snap29	28 Weeks	-0.331383902	0.318415934	-1.040726505	0.30232145
Ccl20	22 Weeks	-0.318177586	0.30677123	-1.037181961	0.303121887
Clmp	12 Weeks	0.0461125	0.044525675	1.035638423	0.303571002
Tnfrsf11b	12 Weeks	-0.112279833	0.108675242	-1.033168469	0.304717867
Itgb1bp2	22 Weeks	0.133532816	0.129302183	1.03271896	0.305191907
Ccl20	20 Weeks	0.247782437	0.240320883	1.0310483	0.305876297
Ppp1r2	22 Weeks	0.082926034	0.080531477	1.029734434	0.306581514
Foxo1	12 Weeks	-0.080470167	0.078363939	-1.026877512	0.307652151
Gcg	12 Weeks	-0.229979667	0.223989019	-1.026745276	0.307714033
Cntn1	26 Weeks	0.109440918	0.10642046	1.028382304	0.307830635
Il1b	12 Weeks	0.0612255	0.060087326	1.018942005	0.311380607
Crim1	12 Weeks	-0.082174333	0.081047366	-1.013905049	0.313762889
Igsf3	22 Weeks	0.098258218	0.096860258	1.014432755	0.31377318
Apbb1ip	28 Weeks	-0.178267276	0.175965303	-1.013081972	0.31522871
Il23r	22 Weeks	0.093682471	0.092647878	1.011166943	0.315322644
Dll1	22 Weeks	0.079321264	0.078506609	1.010376904	0.315698248
Tnfrsf11b	28 Weeks	-0.13208248	0.130834225	-1.009540735	0.316908579

Epcam	26 Weeks	-0.132382449	0.131519719	-1.0065597	0.318122599
Plxna4	20 Weeks	-0.109374498	0.108848598	-1.00483148	0.318252478
Adam23	16 Weeks	-0.073308333	0.073490579	-0.99752015	0.321677566
Rgma	26 Weeks	0.056253503	0.056713771	0.991884385	0.325172257
Tnfsf12	26 Weeks	0.098618197	0.099627471	0.989869528	0.326148216
Gcg	16 Weeks	-0.216960168	0.219865931	-0.986783935	0.326879782
Ccl5	26 Weeks	0.103393878	0.104825312	0.986344568	0.327860328
Cpe	16 Weeks	0.058520236	0.059685006	0.980484711	0.329957861
Acvrl1	30 Weeks	0.139690667	0.142661503	0.979175626	0.332207392
Pla2g4a	16 Weeks	-0.11390101	0.116846577	-0.974791156	0.332756399
Pdgfb	24 Weeks	-0.406481368	0.416899614	-0.975010181	0.333062428
Gdnf	22 Weeks	0.071945747	0.073892615	0.973652742	0.333488843
Clstn2	22 Weeks	0.085280057	0.088011832	0.96896128	0.335808263
Foxo1	20 Weeks	-0.072272321	0.074695583	-0.967558166	0.336416758
Tgfb1	30 Weeks	-0.110308333	0.113816314	-0.969178578	0.337121125
Crim1	30 Weeks	0.119086667	0.123498667	0.964274914	0.339548876
Dll1	26 Weeks	0.081647279	0.085014985	0.960386912	0.340651958
Axin1	28 Weeks	-0.19667626	0.205897208	-0.955215769	0.343433059
Igsf3	12 Weeks	0.064821667	0.068111912	0.951693544	0.344192221
Tnfsf12	16 Weeks	-0.086384865	0.090884949	-0.950485929	0.344878319
Fstl3	26 Weeks	0.04292915	0.045331314	0.947008727	0.347370845
Cntn4	12 Weeks	0.061554	0.065130707	0.945084163	0.347534367
Cpe	28 Weeks	0.064728455	0.068558491	0.94413477	0.349017272
Parp1	24 Weeks	0.27702903	0.294466392	0.940783185	0.350195636
Tnf	22 Weeks	0.143190517	0.152290818	0.940243927	0.350236303
Gdnf	26 Weeks	0.087002075	0.092563625	0.939916456	0.350967585
Il1a	24 Weeks	0.23707	0.253263887	0.936059233	0.352604476
Igsf3	14 Weeks	0.074866167	0.080393408	0.931247576	0.354636162
Nadk	28 Weeks	-0.219346301	0.237264182	-0.924481306	0.359066216
Mia	22 Weeks	0.068192126	0.074716125	0.912682852	0.364455354
Nadk	14 Weeks	-0.146737667	0.16120722	-0.91024252	0.365535245

Cxcl1	16	Weeks	-0.177442508	0.195136879	-0.909323289	0.366054256
Il6	14	Weeks	0.148099	0.162948302	0.908871085	0.366254195
Tpp1	28	Weeks	-0.08285687	0.091355713	-0.906969774	0.368175597
Axin1	30	Weeks	0.190164333	0.210049049	0.905332987	0.369630983
Casp3	20	Weeks	-0.167736263	0.186178751	-0.90094204	0.370541993
Il23r	24	Weeks	0.083435299	0.093701175	0.890440258	0.37641589
Gfra1	12	Weeks	0.142714833	0.160578534	0.88875412	0.376867922
Plin1	14	Weeks	0.174906	0.196821615	0.888652397	0.376957643
Dctn2	28	Weeks	0.133084146	0.150552482	0.883971786	0.380361118
Tgfbr3	26	Weeks	-0.056093163	0.063736529	-0.880078721	0.382270806
Axin1	20	Weeks	-0.08439224	0.096565987	-0.873933384	0.384981914
Tgfb1	24	Weeks	-0.054123284	0.061961587	-0.873497369	0.385512099
Nadk	22	Weeks	0.137340977	0.157793492	0.870384293	0.38698311
Kitlg	14	Weeks	-0.043440333	0.050001785	-0.868775643	0.387669746
Ca13	26	Weeks	0.168992789	0.194366063	0.869456256	0.38800598
Cxcl1	26	Weeks	0.253071054	0.291434527	0.868363323	0.388599099
Mia	26	Weeks	0.085115782	0.098232832	0.866469804	0.389628023
Prdx5	26	Weeks	0.292245408	0.34025175	0.858909346	0.393753218
Yes1	20	Weeks	0.171202814	0.200394255	0.85432995	0.395679528
Rgma	24	Weeks	-0.0421151	0.049353887	-0.853328937	0.396517397
S100a4	24	Weeks	0.092884179	0.109288972	0.849895259	0.39841019
Il1a	22	Weeks	0.211419368	0.248899822	0.849415505	0.398465678
Itgb1bp2	30	Weeks	0.097027778	0.113987669	0.851212929	0.398709461
Vsig2	24	Weeks	0.144332687	0.170733331	0.845369124	0.400913677
Tnfsf12	30	Weeks	0.109465333	0.129264812	0.846830095	0.401124951
Vegfd	28	Weeks	-0.072161382	0.085424599	-0.844737729	0.401728395
Crim1	28	Weeks	-0.084862154	0.100610504	-0.843472117	0.402429754
Cntn4	16	Weeks	0.065278754	0.077613185	0.841078147	0.402942731
Cant1	22	Weeks	0.048333621	0.057539219	0.840011754	0.403682556
Gcg	30	Weeks	-0.330371556	0.392945071	-0.840757602	0.404486577
Ntf3	22	Weeks	-0.111015402	0.13252785	-0.837676022	0.404984786

Kitlg	24 Weeks	-0.056491617	0.067580823	-0.835911938	0.406175709
Il1b	28 Weeks	0.211204593	0.253208274	0.834114108	0.407638978
Tnfrsf12a	12 Weeks	0.087231667	0.104954282	0.831139664	0.408431797
Plin1	22 Weeks	0.239095172	0.287584186	0.831391934	0.40850103
Il6	22 Weeks	0.329227069	0.396686836	0.829942008	0.40931496
Ccl2	24 Weeks	0.229950522	0.27728515	0.829292599	0.409883712
Prdx5	20 Weeks	-0.085829642	0.103593579	-0.828522797	0.410038817
Axin1	22 Weeks	0.085397471	0.103138295	0.827989942	0.410412326
Tgfa	28 Weeks	0.102057846	0.123141879	0.82878259	0.410625174
Vegfd	26 Weeks	0.063299286	0.076611007	0.826242701	0.411886522
Ccl3	24 Weeks	0.205715025	0.249308466	0.825142556	0.412218932
Crim1	24 Weeks	0.081225821	0.098770277	0.822371096	0.413782909
Vegfd	20 Weeks	0.053340672	0.065595142	0.813180223	0.41872343
Gdnf	20 Weeks	-0.071681174	0.088306439	-0.811732129	0.419548786
Pla2g4a	12 Weeks	-0.147397667	0.182864618	-0.806048039	0.422665825
Kitlg	22 Weeks	0.049672931	0.061614183	0.806193129	0.422786332
Cntn1	28 Weeks	0.06318126	0.078777002	0.802026714	0.425811461
Foxo1	28 Weeks	-0.110785407	0.139869856	-0.79206063	0.431552991
Il5	20 Weeks	-0.086846631	0.110088029	-0.788883514	0.432700289
Kitlg	12 Weeks	-0.0730815	0.092918834	-0.786509006	0.433952423
Hgf	14 Weeks	0.093779833	0.11984515	0.782508375	0.436315762
Hgf	24 Weeks	0.165451468	0.212620486	0.778153935	0.439217673
Ntf3	30 Weeks	-0.127318667	0.164620006	-0.773409441	0.442921387
Map2k6	28 Weeks	-0.286583211	0.372266939	-0.769832562	0.444523392
Plxna4	28 Weeks	-0.176095325	0.228813724	-0.769601239	0.444659562
Cxcl1	20 Weeks	0.166245511	0.216547454	0.767709375	0.445102663
Lgmn	12 Weeks	0.048847833	0.064121654	0.761799328	0.44847711
Il17f	30 Weeks	0.044287222	0.058054916	0.762850505	0.449136782
Fli1	30 Weeks	0.172301444	0.226477347	0.760788869	0.450356262
Vsig2	16 Weeks	-0.088208569	0.116312832	-0.758373499	0.450572825
Il5	12 Weeks	0.069227667	0.091521405	0.75640957	0.451682272

Il1a	16 Weeks	-0.198013451	0.264253317	-0.749331942	0.455970318
Il10	22 Weeks	0.15562092	0.207990861	0.748210374	0.456770357
Tnr	24 Weeks	-0.086565771	0.116127908	-0.745434691	0.458616157
Ahr	20 Weeks	-0.07106302	0.095748051	-0.742187639	0.460322987
Pdgfb	30 Weeks	0.541116778	0.728141347	0.743147989	0.460869743
Ddah1	28 Weeks	0.082987683	0.111920305	0.741489071	0.461388863
Tgfb1	16 Weeks	0.054776077	0.074151309	0.738706821	0.462360292
Flrt2	30 Weeks	0.077763	0.105070383	0.740103899	0.462698136
Dlk1	30 Weeks	0.079851222	0.108067603	0.738900653	0.463421998
S100a4	16 Weeks	-0.070544545	0.095969021	-0.735076219	0.464555373
Tnf	30 Weeks	0.101691222	0.138068619	0.736526683	0.464852062
Pak4	30 Weeks	0.113085	0.153618822	0.736140264	0.465085078
Axin1	14 Weeks	-0.061709167	0.084327315	-0.731781468	0.466523351
Tpp1	20 Weeks	-0.065519176	0.089625354	-0.731033943	0.467066886
Clstn2	12 Weeks	0.055578667	0.076396742	0.72750048	0.469097522
Yes1	14 Weeks	0.083694667	0.116504331	0.71838245	0.474695722
Flrt2	22 Weeks	-0.050830575	0.070749583	-0.71845759	0.474800264
Csf2	26 Weeks	0.051305476	0.071798285	0.714578018	0.477595618
Lpl	20 Weeks	-0.076909552	0.107974349	-0.712294661	0.478522172
Fst	26 Weeks	0.106250816	0.149127037	0.712485265	0.478880835
Tnfsf12	28 Weeks	0.065945285	0.093458134	0.705613109	0.483253264
Cntn1	22 Weeks	-0.074168218	0.105319152	-0.70422347	0.483565163
Tpp1	24 Weeks	-0.061102488	0.086814373	-0.703829161	0.483978153
Snap29	12 Weeks	-0.164926	0.234553758	-0.703147975	0.48405705
Csf2	24 Weeks	0.052647612	0.074950059	0.702435898	0.484840723
Tpp1	22 Weeks	-0.062007011	0.08856825	-0.70010429	0.486118245
Tnf	26 Weeks	0.113188503	0.161979615	0.698782394	0.48734379
Fst	28 Weeks	0.067216545	0.096373345	0.697459914	0.488301524
Ccl3	30 Weeks	0.186727889	0.268165852	0.696314938	0.489456065
Fst	30 Weeks	0.093292889	0.134392725	0.694181096	0.490781576
Cxcl1	14 Weeks	0.1241425	0.181613462	0.683553403	0.496309062

Hgf	16 Weeks	-0.093790017	0.137242315	-0.683389936	0.496438672
Ccl3	20 Weeks	-0.062833369	0.092109956	-0.682156107	0.497269965
Epo	22 Weeks	-0.206679483	0.302950049	-0.682222973	0.497286943
Itgb6	12 Weeks	0.060718333	0.089161674	0.680991399	0.49789367
Axin1	12 Weeks	-0.087008	0.128074987	-0.679352011	0.498925916
Ccl5	24 Weeks	0.110268632	0.163115154	0.676017092	0.50135669
Tnni3	22 Weeks	0.187189253	0.277264001	0.675130028	0.501755566
Nadk	20 Weeks	-0.118735529	0.176748915	-0.671775149	0.50381875
Riox2	24 Weeks	0.144520299	0.215435155	0.670829693	0.504635074
Eno2	20 Weeks	0.068280959	0.10192227	0.669931694	0.504986524
Tgfb1	28 Weeks	-0.05982061	0.089439687	-0.668837424	0.506252675
Clmp	24 Weeks	0.055088657	0.082551659	0.667323432	0.506857525
Sez6l2	16 Weeks	-0.066764276	0.100193986	-0.666350133	0.507205094
Ccl20	24 Weeks	-0.17744709	0.267043194	-0.664488344	0.50865839
Epo	28 Weeks	-0.22540622	0.338945545	-0.665021927	0.50867227
Gfra1	30 Weeks	0.217353778	0.327435339	0.663806719	0.509863453
Plxna4	22 Weeks	-0.088953046	0.134486712	-0.66142628	0.510450162
Il17a	16 Weeks	0.209606919	0.317454059	0.660274813	0.511073759
Apbb1ip	12 Weeks	-0.069794833	0.106152407	-0.657496474	0.51279737
Ccl20	16 Weeks	0.125789444	0.192294085	0.654151398	0.514988901
Plxna4	14 Weeks	0.091610667	0.141064033	0.649426114	0.517995575
Ddah1	24 Weeks	-0.058436517	0.090174048	-0.648041414	0.519172872
Pla2g4a	20 Weeks	-0.094490323	0.146159648	-0.646487071	0.519964066
Tnni3	28 Weeks	-0.111135122	0.171926891	-0.646409188	0.520564144
Yes1	12 Weeks	0.074963167	0.11627537	0.644703747	0.521010833
Il1a	28 Weeks	0.28849187	0.447040072	0.64533783	0.521253093
Cant1	14 Weeks	0.032582667	0.050740759	0.642139921	0.52268958
Tnni3	12 Weeks	0.091747	0.143004969	0.641565119	0.523036462
Ghrl	16 Weeks	0.08617197	0.135142738	0.637636702	0.525626631
Riox2	14 Weeks	0.103759667	0.163236506	0.635640085	0.526895711
Cxcl1	12 Weeks	0.1119135	0.176658502	0.633501918	0.528259176

Tnr	20	Weeks	0.043580466	0.069859826	0.623827287	0.53465935
Ca13	12	Weeks	0.072362833	0.116535796	0.620949405	0.536443278
Pdgfb	28	Weeks	-0.369744431	0.59644259	-0.619916212	0.537740932
Ccl20	28	Weeks	0.174798171	0.282075812	0.619685076	0.537892065
Ahr	24	Weeks	-0.082247363	0.133338512	-0.616831267	0.539436499
Vegfd	22	Weeks	0.04574977	0.07435654	0.615275674	0.540312602
Pak4	14	Weeks	0.056738833	0.092246981	0.615075233	0.540318589
Sez6l2	20	Weeks	0.064391228	0.105117204	0.612566023	0.542041232
Notch3	22	Weeks	0.03484408	0.057270378	0.608413659	0.544827811
Itgb1bp2	24	Weeks	-0.081590771	0.134157062	-0.608173509	0.54512862
Tnfsf12	20	Weeks	0.051806353	0.085614914	0.605108977	0.546957802
Apbb1ip	14	Weeks	-0.049757333	0.082385658	-0.603956251	0.547648019
Fas	28	Weeks	0.044567195	0.073767491	0.604157665	0.548094838
Ccl5	14	Weeks	0.043783	0.072718751	0.602086795	0.548885236
Gcg	14	Weeks	-0.131637667	0.219534705	-0.599621216	0.550519118
Flrt2	14	Weeks	-0.041877	0.070335868	-0.595386126	0.553331299
Ppp1r2	28	Weeks	-0.093893415	0.157491112	-0.596182307	0.553373184
Tnfsf12	22	Weeks	0.053666207	0.090283811	0.594416719	0.554096734
Il10	30	Weeks	0.125875444	0.212145425	0.593345081	0.555624907
Matn2	12	Weeks	0.059872667	0.10208988	0.586470145	0.5592531
Il1a	14	Weeks	0.14564	0.248820545	0.585321441	0.560043108
Gcg	26	Weeks	0.129129524	0.224079898	0.576265542	0.566555987
Yes1	28	Weeks	0.101126585	0.177271786	0.570460689	0.57056877
Fli1	12	Weeks	-0.092542667	0.163190697	-0.567082978	0.572286233
Hgf	20	Weeks	0.086905394	0.15362088	0.565713424	0.573300034
Acvrl1	20	Weeks	0.053449498	0.095307681	0.560809974	0.576621269
Yes1	16	Weeks	-0.068366044	0.122079521	-0.560012384	0.57711796
Epcam	16	Weeks	-0.064626296	0.116060507	-0.556832793	0.579277781
Tnfrsf11b	14	Weeks	-0.0614415	0.110567203	-0.555693718	0.580031316
Tgfbr3	30	Weeks	-0.037414333	0.067717738	-0.552504184	0.583064589

Tnfsf12	14 Weeks	0.038836667	0.070577401	0.550270566	0.583726627
Ca13	22 Weeks	-0.076127931	0.138416337	-0.549992382	0.584026775
Gdnf	16 Weeks	-0.035691313	0.065341451	-0.546227738	0.586509381
Nadk	12 Weeks	0.093355833	0.171811275	0.543362673	0.588429681
Notch3	24 Weeks	0.033654403	0.062018557	0.542650529	0.589171042
Foxo1	22 Weeks	-0.040416034	0.074666891	-0.54128455	0.589982424
Eno2	16 Weeks	-0.03178367	0.058738415	-0.541105339	0.590017572
Ccl5	28 Weeks	-0.059305447	0.109688324	-0.540672378	0.590803847
Adam23	12 Weeks	-0.037529167	0.069542967	-0.539654376	0.590972718
Il17a	28 Weeks	-0.216746057	0.401694587	-0.539579232	0.59155282
Tnni3	30 Weeks	0.438783	0.813050185	0.539675174	0.591816534
Riox2	12 Weeks	0.104864833	0.195310256	0.536914114	0.59285521
Casp3	30 Weeks	0.153347556	0.286446225	0.535345004	0.594784543
Tnni3	24 Weeks	0.286626493	0.53794727	0.532815219	0.595924354
Igsf3	16 Weeks	0.042363754	0.079564194	0.532447476	0.595969415
Tnr	26 Weeks	-0.049063367	0.092982282	-0.527663615	0.59964699
Ddah1	12 Weeks	-0.031283667	0.059473977	-0.526005962	0.600376467
Vegfd	24 Weeks	-0.049955249	0.095843307	-0.521217918	0.603933557
Plin1	12 Weeks	0.1087725	0.209528945	0.519128753	0.605140849
Mia	28 Weeks	0.04682561	0.090119104	0.519596931	0.605321931
Sez6l2	12 Weeks	-0.041273	0.080488878	-0.512778921	0.609555151
Acvrl1	24 Weeks	0.072786343	0.143887304	0.505856608	0.614617657
Pla2g4a	30 Weeks	0.119883667	0.239347631	0.500876762	0.618655767
Pla2g4a	22 Weeks	0.057463161	0.115774148	0.496338449	0.621167567
Pdgfb	14 Weeks	-0.10702	0.217781803	-0.491409285	0.624534848
Fli1	28 Weeks	0.138191138	0.280908079	0.491944336	0.62461553
Dll1	16 Weeks	0.035437727	0.072361324	0.489732984	0.625733525
Clmp	26 Weeks	0.034595272	0.070995753	0.487286499	0.627801922
Ccl3	26 Weeks	0.069028435	0.141778207	0.486876204	0.628090986
Notch3	16 Weeks	0.021588502	0.044400463	0.48622244	0.628208551
Tnni3	20 Weeks	-0.057834884	0.121074157	-0.477681486	0.634284911

Ca13	30 Weeks	0.076545333	0.159958746	0.478531718	0.634357748
Dctn2	12 Weeks	0.038646833	0.080943381	0.477455142	0.63437326
Ca13	20 Weeks	-0.071086828	0.149154233	-0.476599467	0.635051708
Tnf	16 Weeks	-0.035530253	0.075135856	-0.472880122	0.637653996
Snap29	24 Weeks	-0.104564876	0.222372944	-0.470223013	0.639722769
Ddah1	20 Weeks	0.030898844	0.065836652	0.469325869	0.640216643
Il17f	26 Weeks	0.035331633	0.075770446	0.466298332	0.642662931
Clstn2	16 Weeks	0.044656818	0.096371538	0.463381814	0.644415022
Crim1	26 Weeks	0.055036769	0.119063615	0.462246745	0.645548928
Fli1	26 Weeks	0.084331531	0.183001882	0.460823297	0.646564171
Plin1	28 Weeks	-0.158091463	0.343430953	-0.460329688	0.64700082
S100a4	28 Weeks	0.044500325	0.096753522	0.459934941	0.647282467
Foxo1	24 Weeks	-0.04749097	0.103336593	-0.459575536	0.647308695
Plin1	16 Weeks	-0.094729478	0.206799466	-0.458074093	0.648206284
Ccl20	26 Weeks	0.100753469	0.220226882	0.457498507	0.64893813
Tnfrsf11b	24 Weeks	0.062923159	0.137693002	0.456981534	0.649162552
Itgb1bp2	28 Weeks	-0.050519065	0.111783638	-0.451936131	0.65300063
Crim1	22 Weeks	0.044475172	0.09938835	0.447488789	0.655865606
Tnr	30 Weeks	-0.041429667	0.093121995	-0.444896682	0.658314482
Tnni3	16 Weeks	0.074836768	0.168926172	0.443014642	0.659013608
Fst	14 Weeks	-0.036019667	0.081509681	-0.441906606	0.659795429
Qdpr	24 Weeks	0.084089677	0.190271353	0.441946069	0.65995147
Cxcl1	24 Weeks	0.130385522	0.296489357	0.439764596	0.661522911
Il6	26 Weeks	0.200462109	0.457728419	0.437949886	0.662969533
Cant1	26 Weeks	0.026785	0.061635028	0.434574313	0.665404914
Ccl2	28 Weeks	0.091353374	0.213685266	0.427513678	0.670588317
Tnni3	26 Weeks	0.195852041	0.458520579	0.427139042	0.670782
Pla2g4a	26 Weeks	0.096065442	0.225305083	0.426379383	0.671332355
Crim1	20 Weeks	0.039544185	0.093806703	0.421549667	0.674575787
Itgb1bp2	26 Weeks	-0.048487993	0.116119758	-0.417568844	0.677728514

Fstl3	16 Weeks	0.020717862	0.049797437	0.41604274	0.678551235
Eno2	14 Weeks	0.026374333	0.063642516	0.414413744	0.679723354
Flrt2	26 Weeks	-0.030390986	0.075155487	-0.404374818	0.687351493
Pla2g4a	28 Weeks	-0.107242602	0.266157974	-0.402928381	0.688482321
Adam23	20 Weeks	-0.031638073	0.078667622	-0.402174014	0.688715279
Itgb6	14 Weeks	0.033141333	0.084548519	0.391980059	0.696155758
Epo	16 Weeks	-0.097105859	0.248405846	-0.390916156	0.696952932
Tgfbr3	20 Weeks	-0.022827563	0.058435455	-0.390645758	0.697181457
Parp1	14 Weeks	0.054758667	0.140510003	0.389713654	0.697824152
Il17a	20 Weeks	-0.139775672	0.359720517	-0.388567416	0.698711899
Il5	30 Weeks	0.083604222	0.215166495	0.388555952	0.699254309
Cntn1	12 Weeks	0.022739333	0.059299653	0.383464865	0.702418314
Erbb4	30 Weeks	-0.048207556	0.125974036	-0.382678505	0.703580146
Epcam	28 Weeks	-0.039718862	0.10450155	-0.380079166	0.705274961
Cant1	12 Weeks	0.019047167	0.050733363	0.375436704	0.708354753
Fst	22 Weeks	-0.036417184	0.097466205	-0.373639086	0.709770802
Ghrl	28 Weeks	0.043213618	0.117422117	0.368019407	0.714198481
Ahr	28 Weeks	-0.066458455	0.181090026	-0.366991252	0.714961136
Map2k6	12 Weeks	-0.075253167	0.205666647	-0.365898738	0.715431143
Ccl3	28 Weeks	-0.080543089	0.220062196	-0.366001481	0.715695595
Qdpr	16 Weeks	-0.059798519	0.165178184	-0.362024312	0.718338539
Casp3	26 Weeks	-0.081008844	0.223625609	-0.362252086	0.718415673
Ppp1r2	20 Weeks	-0.032858584	0.091079696	-0.360767391	0.719301179
Tnfrsf11b	20 Weeks	-0.044801263	0.124233194	-0.360622325	0.719409181
Ccl3	16 Weeks	0.051931818	0.144463098	0.359481549	0.720231797
Acvrl1	28 Weeks	-0.036046016	0.101914504	-0.353688777	0.724854598
Pak4	28 Weeks	0.053451951	0.15196849	0.35173049	0.726315074
Adam23	14 Weeks	0.026976	0.077085916	0.349947193	0.727333724
Fli1	24 Weeks	0.062501318	0.17884429	0.34947338	0.727830014
Il10	14 Weeks	0.042083167	0.120913421	0.3480438	0.728756996

Cntn1	14	Weeks	0.022632667	0.065270644	0.346751086	0.729724175
Tnni3	14	Weeks	0.042734833	0.12389903	0.344916608	0.731097441
Tpp1	12	Weeks	0.022820333	0.066341078	0.343984966	0.731783194
Dlk1	14	Weeks	0.022192667	0.064718441	0.342911019	0.732599804
Rgma	16	Weeks	-0.016685741	0.04869317	-0.342671072	0.732791878
Tnfsf12	12	Weeks	0.024921833	0.073602421	0.338600727	0.735820329
Sez6l2	22	Weeks	0.038456264	0.116465968	0.330193146	0.742212872
Il17a	26	Weeks	-0.132903776	0.4060114	-0.32734001	0.74453147
Tnfsf12	24	Weeks	0.036126368	0.110649736	0.326493034	0.745069073
Ntf3	24	Weeks	0.054064453	0.166558616	0.324597154	0.746497284
Eno2	24	Weeks	0.05727505	0.180230756	0.317787325	0.751634601
Epcam	30	Weeks	0.045693778	0.144789501	0.315587647	0.753629035
Cntn4	24	Weeks	0.032040746	0.101701908	0.315045674	0.753706091
Tgfa	24	Weeks	0.036298632	0.115405289	0.314531786	0.754094568
Cant1	16	Weeks	-0.017006616	0.054489078	-0.312110552	0.755811201
Ahr	12	Weeks	-0.023229167	0.074439481	-0.312054389	0.755831844
Clmp	22	Weeks	-0.018556322	0.060025826	-0.309138969	0.758108636
Epcam	20	Weeks	0.031714391	0.103249133	0.307163747	0.759581489
Ccl20	12	Weeks	0.0741585	0.241684391	0.306840255	0.759782623
Qdpr	20	Weeks	0.046419176	0.151342533	0.306715996	0.759920949
Tpp1	26	Weeks	-0.032424762	0.106304503	-0.305017764	0.761390947
Il10	20	Weeks	-0.024460735	0.08236437	-0.296981991	0.767312285
Epcam	12	Weeks	0.029385333	0.099310495	0.295893533	0.768097719
Adam23	24	Weeks	0.027882811	0.095124959	0.293117717	0.770338131
Tgfa	12	Weeks	-0.0323065	0.110310639	-0.292868398	0.770400454
Gcg	24	Weeks	-0.069710572	0.240783499	-0.28951557	0.773080901
Ccl20	14	Weeks	-0.0734805	0.257369389	-0.285505981	0.776023133
Cant1	28	Weeks	0.014907886	0.052464527	0.284151733	0.777306093
Tpp1	14	Weeks	0.022251667	0.081736761	0.272235729	0.786169446
Il1a	20	Weeks	0.069359211	0.254746347	0.272267737	0.786174418
Il17a	30	Weeks	0.129557222	0.476125864	0.272107088	0.786660196

Rgma	28 Weeks	0.012912886	0.047512239	0.271780207	0.786756121
Ccl2	20 Weeks	-0.074085296	0.272952044	-0.271422389	0.786821937
Cntn4	22 Weeks	0.026676149	0.098594287	0.27056486	0.787499823
Ccl2	14 Weeks	0.0760255	0.281811932	0.269773886	0.788055885
Rgma	12 Weeks	-0.010092	0.037527226	-0.268924751	0.788697642
Sez6l2	30 Weeks	0.035135333	0.13223995	0.265693788	0.791567531
Cntn1	16 Weeks	-0.020823081	0.078518771	-0.265198763	0.791574339
Il10	16 Weeks	-0.036395724	0.139928524	-0.260102249	0.795488278
Prdx5	12 Weeks	0.018907333	0.073300061	0.257944305	0.797129245
Il5	24 Weeks	-0.040124428	0.157064911	-0.255463983	0.799147384
Plxna4	26 Weeks	-0.051737347	0.206957979	-0.249989622	0.803435372
Cntn1	24 Weeks	-0.028255274	0.114457888	-0.246861742	0.805770064
Tgfa	22 Weeks	-0.024472586	0.100709924	-0.243000743	0.808695775
Qdpr	28 Weeks	0.049543455	0.204441941	0.242335086	0.809375403
Apbb1ip	20 Weeks	-0.026855	0.111191248	-0.241520807	0.809819498
Rgma	22 Weeks	-0.011023506	0.046191361	-0.238648647	0.812055986
Nadk	24 Weeks	0.041446891	0.174452477	0.237582701	0.81292979
Plxna4	16 Weeks	0.028835657	0.121989255	0.236378659	0.813774768
Ddah1	26 Weeks	0.030974898	0.13259284	0.233609129	0.816070647
Eno2	22 Weeks	0.013449598	0.057705067	0.233074813	0.81636465
Parp1	20 Weeks	0.041597572	0.181740612	0.228884294	0.819590227
Tnfrsf12a	28 Weeks	0.020966057	0.091933852	0.228055894	0.820404884
Crim1	14 Weeks	-0.018871	0.083703091	-0.225451651	0.822225236
Kitlg	28 Weeks	0.014991707	0.068192578	0.219843679	0.826764834
Tnr	14 Weeks	-0.011625833	0.053330944	-0.217994141	0.828010359
Il1b	16 Weeks	-0.030446785	0.142232063	-0.214064143	0.831070319
Map2k6	22 Weeks	0.041106897	0.199640945	0.205904137	0.837446891
Il5	16 Weeks	0.020007088	0.097888432	0.204386639	0.838597723
Il1b	20 Weeks	-0.02400147	0.119067226	-0.201579145	0.840798846
Csf2	28 Weeks	-0.013098293	0.065029261	-0.201421521	0.841073739
Gcg	20 Weeks	-0.041205188	0.20533144	-0.200676468	0.841502088

Tnfrsf11 b	30 Weeks	-0.032502889	0.165749502	-0.19609645	0.845329503
Il10	28 Weeks	-0.033055976	0.169142398	-0.195432819	0.845737175
Itgb1bp2	14 Weeks	-0.021789	0.11217943	-0.194233471	0.846504602
Adam23	28 Weeks	-0.016107561	0.084200975	-0.191298984	0.848959464
Ccl5	20 Weeks	-0.015774453	0.082951809	-0.190164068	0.849701266
Pdgfb	22 Weeks	0.05668431	0.298374487	0.189977069	0.849861692
Map2k6	24 Weeks	-0.04670806	0.248186015	-0.18819779	0.851290455
Map2k6	14 Weeks	0.031934333	0.173313388	0.184257741	0.854295669
Lpl	26 Weeks	-0.021487789	0.116771073	-0.184016371	0.854610972
Il5	22 Weeks	0.03069954	0.167591774	0.183180471	0.855171276
Ppp1r2	16 Weeks	0.017367946	0.094993068	0.182833828	0.855415262
Ca13	28 Weeks	-0.039046951	0.223604117	-0.174625368	0.861982158
Riox2	28 Weeks	0.053722846	0.312074142	0.172147699	0.863920687
Csf2	12 Weeks	0.009941167	0.058159654	0.170928918	0.864722289
Parp1	28 Weeks	0.057463008	0.341307708	0.168361296	0.866884792
Pak4	24 Weeks	0.021674627	0.130444018	0.166160375	0.86853115
Clmp	16 Weeks	0.008875387	0.055739372	0.159230127	0.873909966
Cpe	24 Weeks	0.014606766	0.091751381	0.159199415	0.873990696
Itgb1bp2	20 Weeks	-0.017348199	0.109295365	-0.158727672	0.874315826
Tnf	12 Weeks	0.013684333	0.088915166	0.153903254	0.878083519
Il17f	12 Weeks	0.009617667	0.062893006	0.152921083	0.878855416
Axin1	24 Weeks	-0.019827687	0.131525341	-0.150751836	0.880624435
Fli1	14 Weeks	-0.011970167	0.079491086	-0.150585019	0.880696863
Il17a	24 Weeks	-0.064410498	0.441441392	-0.145909511	0.884430927
Sez6l2	14 Weeks	0.012270167	0.086653076	0.141601052	0.887764961
Snap29	14 Weeks	-0.019748167	0.142748337	-0.138342534	0.890330868
Hgf	12 Weeks	0.0173455	0.127828422	0.13569361	0.892413086
Adam23	22 Weeks	-0.01202454	0.089362025	-0.134559844	0.893335338
Cpe	12 Weeks	-0.007528167	0.05609045	-0.134214768	0.893578458
Sez6l2	26 Weeks	0.016635748	0.12486885	0.133225767	0.894453369

Notch3	12 Weeks	-0.005087167	0.039047956	-0.130279975	0.896680329
Ccl20	30 Weeks	-0.050262444	0.3928159	-0.127954201	0.898698476
Sez6l2	28 Weeks	-0.015687724	0.123517168	-0.127008446	0.899373017
Dctn2	16 Weeks	0.015644091	0.126146214	0.12401554	0.901630385
Itgb6	28 Weeks	-0.012968455	0.10644111	-0.121836904	0.90344934
Wisp1	16 Weeks	0.009279377	0.076700142	0.120982529	0.904024165
Fstl3	28 Weeks	-0.005754309	0.047683785	-0.120676431	0.904364413
Tnfrsf11b	26 Weeks	0.017855068	0.152663306	0.116957169	0.907278224
Il17a	22 Weeks	0.044451379	0.382127105	0.116326162	0.907717906
Apbb1ip	24 Weeks	0.019588831	0.177364065	0.110444192	0.912387273
Sez6l2	24 Weeks	-0.012986244	0.122852744	-0.105705769	0.916131646
Fstl3	12 Weeks	-0.0045665	0.043248096	-0.105588462	0.91617995
Csf2	30 Weeks	0.008054	0.076181562	0.105721119	0.916226633
Ahr	22 Weeks	-0.009902184	0.094370219	-0.104929118	0.91672382
Ppp1r2	24 Weeks	-0.011584179	0.114133302	-0.101496924	0.919459137
Fst	24 Weeks	0.011078234	0.109833534	0.100863857	0.919959763
Pak4	26 Weeks	0.013996565	0.139936516	0.100020817	0.920655801
Lpl	24 Weeks	-0.010913632	0.110519637	-0.098748351	0.921632928
Ccl3	14 Weeks	-0.015256833	0.170066657	-0.089710903	0.928749893
Eno2	12 Weeks	-0.004724167	0.053129587	-0.088917812	0.929375139
Acvrl1	14 Weeks	0.007011833	0.08122901	0.086321787	0.931434695
Plin1	24 Weeks	-0.026677214	0.323129752	-0.082558829	0.934448494
Pla2g4a	24 Weeks	0.015276915	0.187947932	0.0812827	0.935459457
Flrt2	20 Weeks	0.005704462	0.072887891	0.078263512	0.937829683
Epo	20 Weeks	0.020765654	0.266538666	0.077908599	0.938111037
Dlk1	20 Weeks	0.007232222	0.094454336	0.076568451	0.939173497
Apbb1ip	26 Weeks	0.011126395	0.145581642	0.076427182	0.939329364
Il17f	24 Weeks	-0.005390572	0.071681714	-0.075201496	0.940278485
Acvrl1	22 Weeks	-0.00981592	0.13507152	-0.072672015	0.942268623
Qdpr	12 Weeks	-0.011223167	0.155162339	-0.07233177	0.942523036

Il10	12 Weeks	0.007693167	0.107963842	0.071256882	0.943375701
Il10	26 Weeks	0.011483027	0.164413283	0.069842454	0.944547517
Qdpr	14 Weeks	-0.010498	0.157056843	-0.066842041	0.946880709
Riox2	20 Weeks	-0.010825762	0.163676672	-0.06614114	0.947443826
Tgfa	30 Weeks	-0.014021889	0.223187405	-0.062825628	0.950155844
Cant1	24 Weeks	0.003945398	0.064804656	0.060881397	0.951634909
Gcg	22 Weeks	0.014131264	0.244689708	0.057751773	0.954106334
Ccl5	16 Weeks	-0.004762256	0.085184146	-0.055905425	0.955563862
Il1b	26 Weeks	-0.01665898	0.315065046	-0.052874731	0.958004498
Tgfa	16 Weeks	0.005956077	0.114781426	0.051890603	0.95875201
Acvrl1	26 Weeks	0.005750136	0.113517944	0.050653983	0.95976675
Lgmn	28 Weeks	-0.00377935	0.075308931	-0.050184614	0.960147701
Vegfd	30 Weeks	0.004226667	0.085009367	0.049720011	0.960543599
Kitlg	16 Weeks	-0.002457862	0.054210144	-0.045339521	0.963955609
Csf2	14 Weeks	0.002504167	0.062359171	0.040157151	0.968071779
Epcam	14 Weeks	-0.004507	0.112284944	-0.040138952	0.968086241
Plin1	30 Weeks	0.012453	0.312493272	0.039850458	0.968371047
Clmp	28 Weeks	-0.00203561	0.0585775	-0.034750711	0.972397846
Matn2	16 Weeks	-0.003401296	0.102072751	-0.033322275	0.973504951
Ca13	24 Weeks	-0.004533408	0.20922319	-0.021667808	0.982777345
Tnfrsf11b	22 Weeks	-0.003071782	0.148760617	-0.020649159	0.983582644
Tgfb1	26 Weeks	0.001678878	0.082643634	0.020314663	0.983858663
Casp3	22 Weeks	0.00336931	0.182373568	0.018474773	0.985311203
Epcam	24 Weeks	-0.001863781	0.132863406	-0.014027799	0.988849491
Ccl3	12 Weeks	0.002545333	0.220148017	0.011561918	0.990804651
Epo	14 Weeks	-0.002625167	0.239647824	-0.010954269	0.991288265
S100a4	22 Weeks	-0.000719138	0.100982151	-0.007121436	0.994337659
Il6	30 Weeks	-0.000812111	0.201003022	-0.004040293	0.996792399
Eno2	26 Weeks	0.000277143	0.114035601	0.002430319	0.998068819
Adam23	26 Weeks	0.000219116	0.097836555	0.002239609	0.99822036

Parp1	12	Weeks	-0.000117333	0.233431869	-0.000502645	0.999600231
Dll1	28	Weeks	2.13821138215581e-05	0.092542617	0.000231052	0.99981644
Lpl	28	Weeks	-1.59E-06	0.11416716	-1.39E-05	0.999988968
